# Updated Global Warming Potentials and Radiative Efficiencies of Halocarbons and Other Weak Atmospheric Absorbers

**DOI:** 10.1029/2019RG000691

**Published:** 2020-09-07

**Authors:** Ø. Hodnebrog, B. Aamaas, J. S. Fuglestvedt, G. Marston, G. Myhre, C. J. Nielsen, M. Sandstad, K. P. Shine, T. J. Wallington

**Affiliations:** ^1^ Center for International Climate Research (CICERO) Oslo Norway; ^2^ Vice‐Chancellor's Office Northumbria University Newcastle UK; ^3^ Department of Chemistry University of Oslo Oslo Norway; ^4^ Department of Meteorology University of Reading Reading UK; ^5^ Research and Advanced Eng. Ford Motor Company Dearborn MI USA

**Keywords:** radiative efficiencies, global warming potentials, halocarbons

## Abstract

Human activity has led to increased atmospheric concentrations of many gases, including halocarbons, and may lead to emissions of many more gases. Many of these gases are, on a per molecule basis, powerful greenhouse gases, although at present‐day concentrations their climate effect is in the so‐called weak limit (i.e., their effect scales linearly with concentration). We published a comprehensive review of the radiative efficiencies (RE) and global warming potentials (GWP) for around 200 such compounds in 2013 (Hodnebrog et al., 2013, https://doi.org/10.1002/rog.20013). Here we present updated RE and GWP values for compounds where experimental infrared absorption spectra are available. Updated numbers are based on a revised “Pinnock curve”, which gives RE as a function of wave number, and now also accounts for stratospheric temperature adjustment (Shine & Myhre, 2020, https://doi.org/10.1029/2019MS001951). Further updates include the implementation of around 500 absorption spectra additional to those in the 2013 review and new atmospheric lifetimes from the literature (mainly from WMO (2019)). In total, values for 60 of the compounds previously assessed are based on additional absorption spectra, and 42 compounds have REs which differ by >10% from our previous assessment. New RE calculations are presented for more than 400 compounds in addition to the previously assessed compounds, and GWP calculations are presented for a total of around 250 compounds. Present‐day radiative forcing due to halocarbons and other weak absorbers is 0.38 [0.33–0.43] W m^−2^, compared to 0.36 [0.32–0.40] W m^−2^ in IPCC AR5 (Myhre et al., 2013, https://doi.org/10.1017/CBO9781107415324.018), which is about 18% of the current CO_2_ forcing.

## Key Points

- Radiative efficiencies are reassessed for more than 600 compounds and global warming potentials calculated for around 250 of these
- Forty‐two compounds have >10% different radiative efficiency compared to a comprehensive review in 2013
- Present‐day radiative forcing due to halocarbons and other weak absorbers is 0.38 [0.33–0.43] W m^−2^, which is ~18% of the CO_2_ forcing

## 1 Introduction

**Figure 1:**
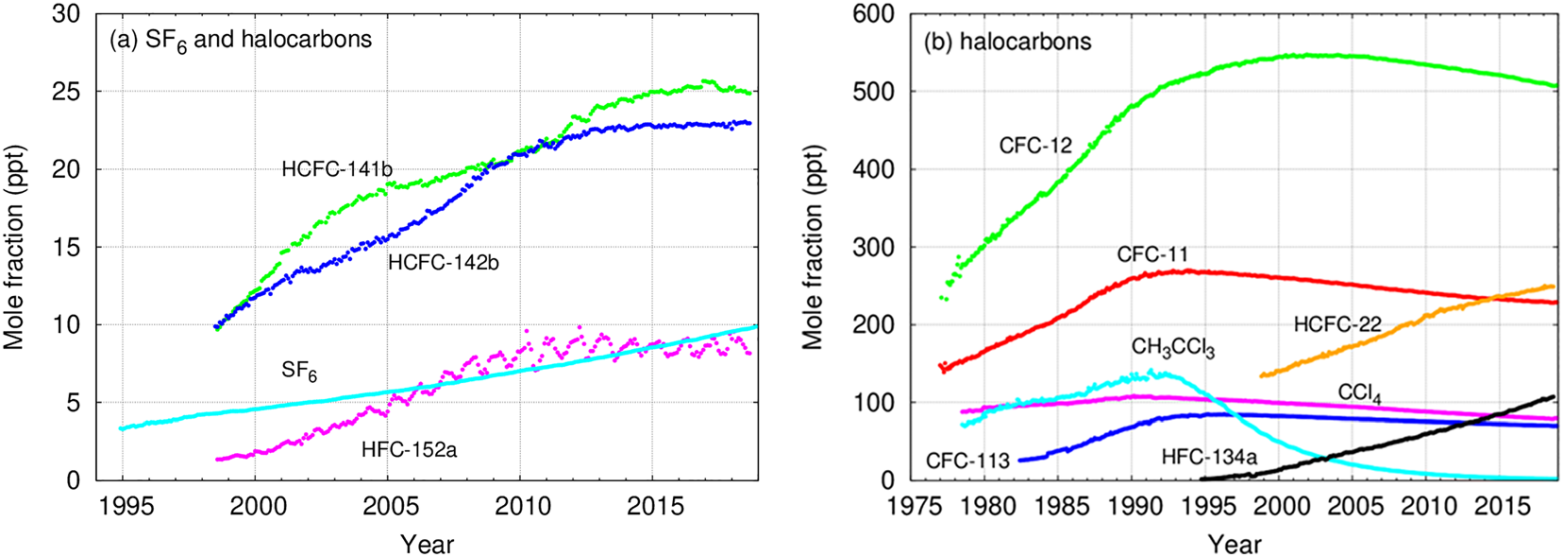
Atmospheric abundances of important halocarbons (and SF_6_), separated into (a) lower and (b) higher mole fractions and based on observations from a number of stations (from WMO/GAW, 2019). The plots are based on the data submitted to the World Data Center for Greenhouse Gases supported by the Japan Meteorological Agency by laboratories participating in the GAW program.

Anthropogenic forcing of climate change is one of the most important challenges facing humanity. The largest contributor to radiative forcing of climate change is the increased levels of greenhouse gases such as CO_2_, N_2_O, CH_4_, and halocarbons and related compounds. While many halocarbons, such as chlorofluorocarbons (CFCs), are known for depleting stratospheric ozone (Molina & Rowland, 1974; WMO, 2019), they are also powerful greenhouse gases. Despite the phase‐out of several halocarbons through the Montreal Protocol from 1987 and its amendments and adjustments, halocarbons still make an important contribution to radiative forcing of climate change because many have long atmospheric lifetimes. Furthermore, the concentrations of some replacement compounds, such as hydrochlorofluorocarbons (HCFCs) and hydrofluorocarbons (HFCs), are rising. More specifically, Figure 1 (WMO/GAW, 2019) shows that HCFC‐22 has recently become the second most abundant compound (of the greenhouse gases with only anthropogenic sources) after CFC‐12. HFC‐134a has, in only 20 years, increased from very low abundance to become the fourth most abundant halocarbon. Emissions of HFCs, perfluorocarbons, SF_6_, and NF_3_ are included in the United Nations Framework Convention on Climate Change (UNFCCC). Controls on emissions of HFCs, in addition to CFCs and HCFCs, are included in the 2016 Kigali Agreement to the Montreal Protocol (see discussion in Kochanov et al., 2019).

Differences in the intensity and wavelength of infrared (IR) absorption bands lead to distinct radiative forcing efficiencies of various gases. Radiative efficiency (RE) is a measure of the radiative forcing for a unit change in the atmospheric concentration of a gas, and for halocarbons and related compounds is usually reported in units of W m^−2^ ppb^−1^. To provide policy makers with guidance on the relative effectiveness of actions limiting the emissions of different gases, metrics have been developed to place the impact of emissions of different gases on a common scale. The most widely used metric is the global warming potential (GWP) with a 100‐year time horizon (hereafter GWP(100)), which is based on the time‐integrated radiative forcing due to a pulse emission of a unit mass of gas, normalized by the reference gas CO_2_ and was introduced in the first assessment report of the Intergovernmental Panel on Climate Change (IPCC, 1990) (see section 2.5).

In 2013 we reviewed the literature data and provided a comprehensive and self‐consistent set of new calculations of REs and GWPs for halocarbons and related compounds (Hodnebrog et al., 2013, hereafter referred to as H2013). Unlike the major greenhouse gases, current atmospheric concentrations of these compounds are low enough for the forcing to scale almost linearly with abundance, and we will therefore refer to these compounds as weak atmospheric absorbers. Adopting a common method for calculating REs and GWPs provides a more consistent approach to comparing metrics between different compounds than if these metrics are taken from studies that used different methodologies. Our results were incorporated by the IPCC into the fifth assessment report (AR5) (Myhre et al., 2013) and, as a result, they are now used in national and international agreements. The UNFCCC adopted AR5 values for reporting emissions under the Paris Agreement and the U.S. Environmental Protection Agency (EPA) uses GWP values from AR5 in its reports. To ensure that climate policy decisions are based on the latest scientific data, it is important to periodically review and update the assessments. Additional infrared absorption spectra and refinements in estimations of the atmospheric lifetimes of halocarbons and other compounds have become available since our last review. Specifically, we have considered and included absorption spectra given as supporting information to published papers, and from the HITRAN2016 (Kochanov et al., 2019) and PNNL (Sharpe et al., 2004) databases. Atmospheric lifetimes have recently been updated in WMO (2019) and these estimates have been used here. The provision of GWP(100) values in this paper, and in H2013, should not be seen as an endorsement of that metric, as the choice of metric depends on the policy context (Myhre et al., 2013); the RE and lifetime values presented here can be used to derive values for alternative emission metrics.

We have updated and extended our previous assessment of REs and GWPs for halocarbons and other weak atmospheric absorbers. Updates are based on new absorption spectra for 60 compounds considered in our previous review, the latest estimates of atmospheric lifetimes, and an update to the RE calculation method. The review has been extended to include around 440 additional compounds to bring the total number of compounds considered to more than 600. Included are several isomeric species which have identical empirical formulae but are structurally and spectrally distinct. Therefore, there is no need to consider isomeric compounds together within the context of this review. The radiative forcing contributions of the 40 most abundant halocarbons and related compounds in the atmosphere are estimated. The present work is the most comprehensive review of the radiative efficiencies and GWPs of halogenated compounds performed to date.

## 2 Data and Method

### 2.1 Absorption Cross Sections

In addition to the experimental spectra included in H2013 we have included, either in the main or supporting information, all IR absorption spectra available from the HITRAN2016 (Gordon et al., 2017; Kochanov et al., 2019) and PNNL (Sharpe et al., 2004) databases. The vast majority of spectra from PNNL are also available in HITRAN2016 and we have only included data from one of the databases to avoid overlap. The main sources of experimental infrared absorption cross sections in H2013 were the Ford Motor Company (e.g., Sihra et al., 2001), the Spectroscopy and Warming potentials of Atmospheric Greenhouse Gases project (Ballard et al., 2000b; Highwood & Shine, 2000), HITRAN‐2008 (Rothman et al., 2009) and GEISA‐2009 (Jacquinet‐Husson et al., 2011) databases, and data provided by authors of published papers (e.g., Imasu et al., 1995). Several of the spectra used in H2013 were provided in the supporting information and later included in the HITRAN2016 and GEISA‐2015 (Jacquinet‐Husson et al., 2016) databases. Many publications now make available their measured absorption cross sections as supporting information. Since spectra provided as supporting information are typically not in a standardized data format and need to be converted, we could only carry out RE calculations for a limited number of these supporting information spectra, and we have prioritized the 40 most atmospherically abundant compounds. For other studies the reported integrated absorption cross section and RE value, if available, are listed (Tables S1‐S20).

As in H2013, each of the available spectra has been evaluated and if several spectra from the same laboratory group exist, we only use the latest published spectrum. For example, spectra from Sihra et al. (2001) supersede those from Pinnock et al. (1995) and Christidis et al. (1997) due to improvements in the methodology of the Ford laboratory measurements. When more than one spectrum was available from a source, the spectrum that was recorded nearest room temperature and atmospheric pressure was used (see section 2.2 for a discussion of the temperature dependence of cross sections). The choices of spectra to be used in RE calculations have been explained for each group of compounds in the supporting information (Texts S1–S20).

In contrast to H2013, we only consider experimental absorption cross sections that are measured in a laboratory. As a result, 44 of the compounds included in H2013 have been omitted here because experimental spectra are not available, while nine of the compounds that only had calculated spectra in H2013 have been updated with RE values based on experimental spectra. Calculated IR spectra have been published for a vast number of compounds (e.g., Davila et al., 2017; Papanastasiou et al., 2018), with some studies including thousands of compounds (Betowski et al., 2016; Kazakov et al., 2012; McLinden et al., 2014) but these have a considerably larger uncertainty than experimental spectra (see Table 1 of H2013).

### 2.2 Temperature Dependence of Cross Sections

**Figure 2:**
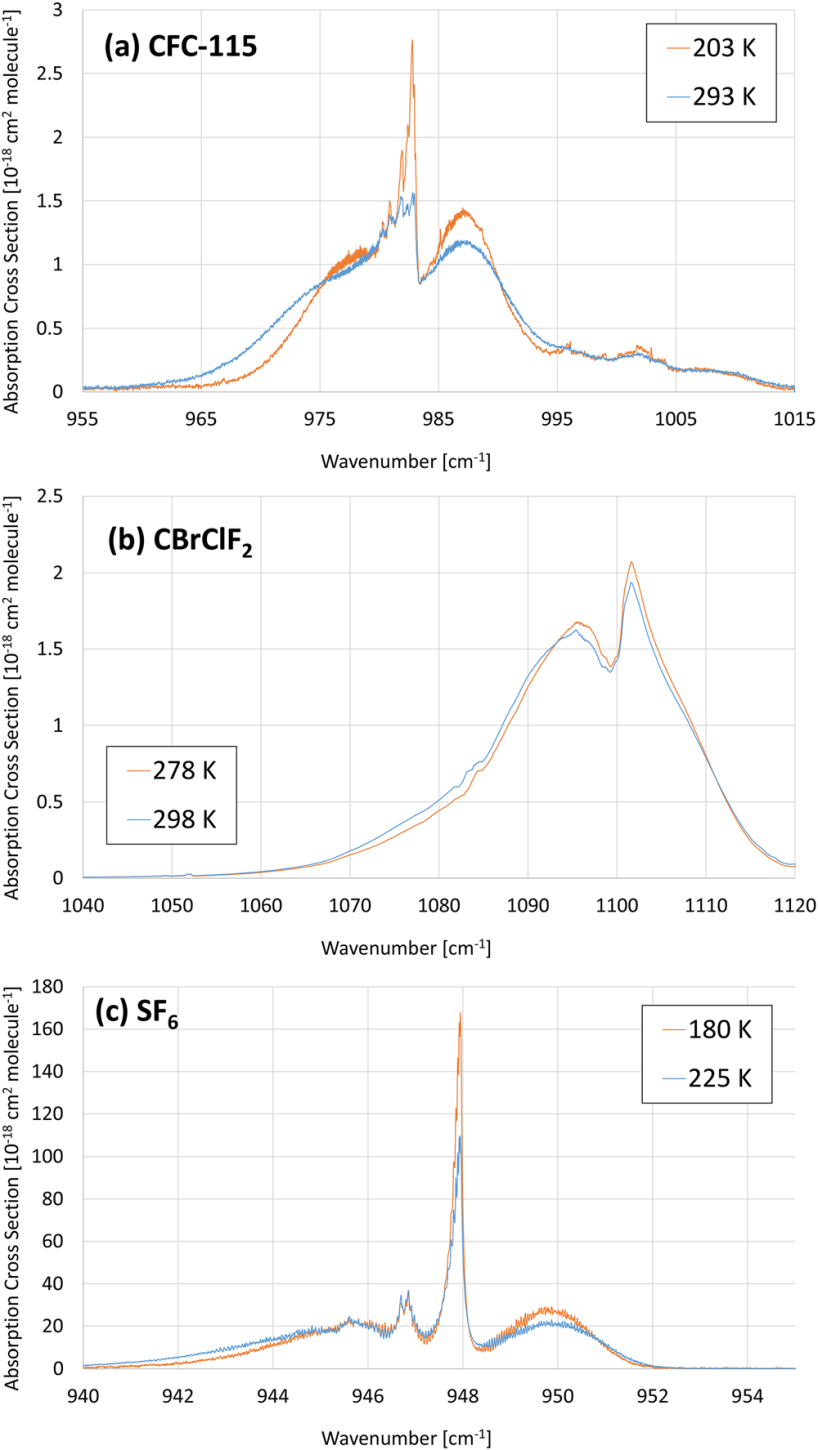
Effect of temperature on band shape. (a) CFC‐115: *T* = 203 K, *p* = 0 Torr; *T* = 298 K, *p* = 0 Torr (Massie et al., 1991; McDaniel et al., 1991). (b) CBrClF_2_: *T* = 273 K, *p* = 760 Torr; *T* = 293 K, *p* = 760 Torr (Sharpe et al., 2004). (c) SF_6_: *T* = 180 K, *p* = 75 Torr; *T* = 225 K, *p* = 78 Torr (referred to as Varanasi, private communication, 2000, in HITRAN).

Although absorption cross sections are temperature dependent, integrated absorption cross sections show little dependence on temperature. The origin of the temperature dependence of absorption cross sections is the strong dependence of rotational states on temperature. Consequently, spectral bands are generally broader and have a lower peak intensity when observed at higher temperatures. This effect is illustrated in Figure 2 for a range of compound types (CFC, halon and sulfur‐containing species), temperature range and pressure. The effect is noticeable even for the 20 K temperature difference illustrated in Figure 2 for CBrClF_2_. These small changes in band structure have a negligible effect on calculated REs, and hence GWPs.

However, when molecules exist in two or more distinct conformational forms, the possibility of significant temperature dependence of the integrated cross section exists (Godin et al., 2019). For example, the absorption spectra for CFC‐114 reported by McDaniel et al. (1991) indicate that there are bands within the spectrum that show relatively strong positive temperature dependence, bands that show a weak negative temperature dependence, and bands that are not temperature dependent. These observations can be rationalized in terms of the temperature dependence of the populations of the two different conformers of CFC‐114. However, the integrated cross sections of most molecules show little temperature dependence, and for consistency, we have used spectra obtained at ambient temperatures, where the experimental uncertainties are typically smallest.

### 2.3 Radiative Efficiency

**Figure 3:**
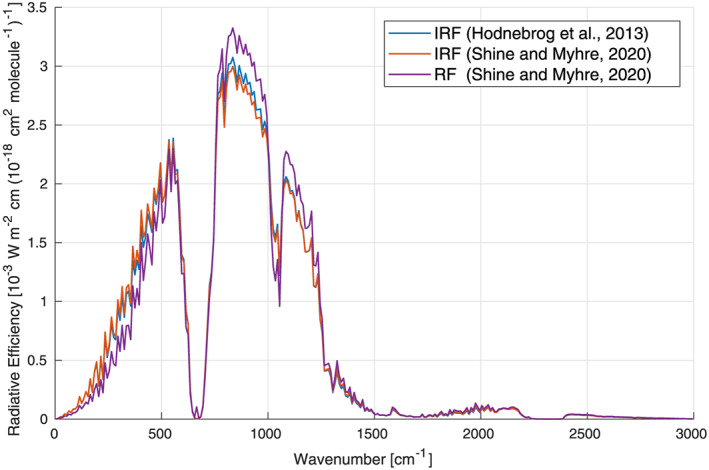
Instantaneous radiative forcing (IRF) efficiency (for a 0–1 ppb increase in mixing ratio) per unit cross section compared between the previous (Hodnebrog et al., 2013) and updated (Shine & Myhre, 2020) results from the Oslo line‐by‐line (OLBL) radiative transfer model run at 0.02 cm^−1^ spectral resolution. Also shown is the new radiative forcing (RF) efficiency where the effect of stratospheric temperature adjustment per unit cross section, based on 10 cm^−1^ narrow band model (NBM) simulations (Shine & Myhre, 2020), have been used to modify the OLBL curve. The curves have been averaged to 10 cm^−1^ spectral resolution in the plot, to improve readability, but RE calculations in this paper have been made using a 1 cm^−1^ version of the RF efficiency curve (as provided in the supporting information of Shine and Myhre, 2020).

In H2013, a common method was used to calculate the RE for most gases. This employed the “Pinnock curve” (Pinnock et al., 1995) where the RE as a function of wave number was calculated for a weak absorber absorbing equally at all wave numbers. Multiplying this curve by the absorption cross section of a given gas yields its RE. In H2013 the Pinnock curve was updated (Figure 3, blue line), most notably by increasing its spectral resolution from 10 to 1 cm^−1^ using the Oslo Line‐By‐Line (OLBL) radiative transfer model run at 0.02 cm^−1^ resolution (note that there was a typo in the caption of Figure 6 in H2013, wrongly stating a resolution of 0.2 cm^−1^); the updated calculations also used more refined atmospheric profiles of temperature, cloudiness and greenhouse gas concentrations. For instance, the atmospheric representation was expanded from one global mean profile to two profiles, one for the tropics and one for the extratropics, and the inclusion of refined cloud profiles led to weaker RE in the 800–1,200 cm^−1^ region (see sections 2.3 and 3.3.1 of H2013 for details). The Pinnock et al. (1995) method, and the H2013 update, yield the instantaneous RE (i.e., the radiative efficiency in the absence of stratospheric temperature adjustment). Since the RE, which includes this adjustment, provides a more accurate representation of a gas's impact on surface temperature, H2013 incorporated a correction to account for this. For most gases, the instantaneous RE was simply increased by 10%. For several gases (CFC‐11, CFC‐12, HFC‐41, and PFC‐14) the correction was explicitly calculated using OLBL, either because of the absolute importance of that gas or because, in the case of HFC‐41, it was known that the RE is *less* than its instantaneous value. However, this approach was somewhat ad hoc and may not have been applicable to all gases.

Shine and Myhre (2020) have incorporated stratospheric temperature adjustment into the Pinnock curve for the first time, by calculating the impact of absorption by a gas at a given wave number on stratospheric temperatures (Figure 3, red vs. purple line). The calculation of this adjustment is computationally intensive, as the RE due to absorption by a gas at a given wave number occurs not only at that wave number (as in the case of instantaneous RE) but now depends on the emission by gases (mostly CO_2_, H_2_O, and O_3_) at all other wave numbers. Because of this, Shine and Myhre (2020) calculated the effect of adjustment using a narrow‐band (10 cm^−1^) radiation code, and applied this to updated instantaneous RE calculations using OLBL (which included an improved representation of the water vapor continuum and some changes to the representation of clouds). The new method reproduced detailed calculations for a range of gases (including HFC‐41 and CFC‐11) to better than 1.5%. Although more complicated in its derivation, it is no more complicated than the original Pinnock method in its application. This new method (which also requires the use of the lifetime correction described in section 2.4) is applied to all gases here and hence improves the relative consistency of derived REs.

### 2.4 Atmospheric Lifetimes and Lifetime Correction

The atmospheric lifetime of a compound is required for calculations of GWPs and Global Temperature‐change Potentials (GTPs) (see section 2.5). The RE value obtained from the method described in section 2.3 assumes the compound is well‐mixed in the atmosphere. Most of the compounds included in this study have a nonuniform vertical and horizontal distribution in the atmosphere, and the lifetime can be used to correct for that. Here we use the method presented in H2013 (their section 3.3.4), where two approximations are given depending on the primary loss mechanism of the compound. One approximation is used for compounds primarily being lost through photolysis in the stratosphere: the fractional correction *f* to the RE of *f*(*τ*) = 1 − 0.1826*τ*^−0.3339^ is applicable for lifetimes *τ* of 10 < *τ* < 10^4^ years. Another approximation is used for compounds primarily lost through reaction with OH in the troposphere: $f\left(\tau \right)=\frac{a\tau^{b}}{1+c\tau^{d}}$, where *a* = 2.962, *b* = 0.9312, *c* = 2.994, *d* = 0.9302, and is applicable for 10^−4^ < *τ* < 10^4^ years. The lifetime corrections for very short‐lived compounds should be treated as particularly approximate, as the correction depends on where the emissions take place. Excepted from these approximations are CFC‐11, CFC‐12, and Halon‐1211 because explicit LBL calculations were made in H2013 (see their section 3.3.3) to derive factors to account for non‐uniform mixing. The derived factors were 0.927, 0.970, and 0.937, respectively, and are used here in the RE calculations for these compounds. These factors are less than one, despite being quite long‐lived compounds, because of stratospheric loss due to photolysis.

The recent WMO (2019) report gives the most up‐to‐date and complete overview of atmospheric lifetimes of halocarbons and related compounds, and we rely on these estimates. Explanations and sources for the lifetime estimates in WMO (2019) are given for each compound in their Chapter 1.2 and Table A‐1. For some compounds that do not have a lifetime estimate in WMO (2019), lifetime estimates have been taken from previous literature and sometimes as an average across different estimates if more studies exist (see Tables S1–S20 for references to lifetime estimates). For several compounds, we are not aware of any estimates of lifetimes; for these we only present REs assuming a constant horizontal and vertical distribution in the atmosphere, and no estimates of GWPs can be given.

### 2.5 Description of Metrics

The most widely used emission metric in climate policy is the GWP. It was introduced by IPCC (1990) where values for three time horizons (20, 100, and 500 years) were given. The GWP values were updated in following assessment reports. GWP has been widely adopted in climate policies, and the Kyoto Protocol adopted GWPs for a time horizon of 100 years as its metric for implementing a multigas approach. At UNFCCC COP24 it was decided to use GWP(100) for reporting national emissions to the Paris Agreement, while parties may in addition use other metrics (e.g., global temperature change potential) to report supporting information on aggregate emissions and removals of greenhouse gases, expressed in CO_2_ equivalents (UNFCCC, 2019).

The GWP is based on the time‐integrated radiative forcing due to a pulse emission of a unit mass of a gas. It can be given as an absolute GWP for gas *i* (AGWP_*i*_) (usually in W m^−2^ kg^−1^ year) or as a dimensionless value by dividing the AGWP_*i*_ by the AGWP of a reference gas, normally CO_2_. Thus, the GWP for gas *i* over a time horizon of *H* years is defined as

$${\mathrm{GWP}}_{i}\left(H\right)=\frac{\int_{0}^{H}{\mathrm{RF}}_{i}\left(t\right)dt}{\int_{0}^{H}{\mathrm{RF}}_{{\mathrm{CO}}_{2}}\left(t\right)dt}=\frac{{\text{AGWP}}_{i}\left(H\right)}{{\text{AGWP}}_{{\mathrm{CO}}_{2}}\left(H\right)}.$$

IPCC has usually presented GWPs for a time horizon (*H*) of 20, 100, and 500 years (although IPCC AR5 (Myhre et al., 2013) only gave GWPs for 20 and 100 years). We use updated lifetimes and RE values presented in section 3 to calculate GWPs for 20, 100, and 500 years as in H2013.

The models used to calculate the impulse response function for CO_2_ (Joos et al., 2013) include climate‐carbon cycle feedbacks, but usually no feedbacks are included for the non‐CO_2_ gases when metrics are calculated. IPCC AR5 (Myhre et al., 2013) included this feedback tentatively in the metric values (see their Table 8.7 and supporting information Table 8.SM.16), which increased the GWP(100) values by 10–20%. Gasser et al. (2017) found that accounting for climate‐carbon feedback increases the emission metrics of non‐CO_2_ species but, in most cases, less than indicated in AR5. They also found that when the feedback is removed for both the reference and target gas, the *relative* metric values are generally only modestly different compared to when the feedback is included in both (*absolute* metric values change more markedly); in the case of GWP(100) the differences are less than 1%. As pointed out by Gasser et al. (2017), including or excluding the climate–carbon feedback ultimately depends on the user's goal, but consistency should be ensured in either case. To resolve the consistency issue, we have excluded the climate‐carbon feedback also for CO_2_ by using the impulse response function for CO_2_ based on the Gasser et al. (2017) simple Earth system model (see their Appendix C); their model shows very good agreement with Joos et al. (2013) when the climate‐carbon feedback is included. Our documentation of input data and presentation of calculations allow for the inclusion of the climate‐carbon feedback to our results in further studies or applications, both for CO_2_ and the non‐CO_2_ compounds.

Changes to the parameters in AGWP_CO2_ impact all GWP values, and the GWP(100) values presented in section 3 are about 14% higher than if the old AGWP_CO2_ from AR5 or H2013 had been used. This is due to two changes: (i) The impulse response function for CO_2_ is updated as explained above and (ii) the RE of CO_2_ is updated using 409.8 ppm for 2019 (Butler & Montzka, 2020) and the simplified expression for CO_2_ RF presented in Etminan et al. (2016), which is an update of the formula from Myhre et al. (1998) used in IPCC assessment reports since TAR (IPCC, 2001). Among other improvements, Etminan et al. (2016) made more extensive use of line‐by‐line calculations compared to Myhre et al. (1998). Using the new formula, a 1 ppm change in the CO_2_ concentration at current (year 2019) levels of CO_2_ (409.8 ppm) and N_2_O (331.9 ppb) (Butler & Montzka, 2020) gives a radiative efficiency for CO_2_ of 0.012895 W m^−2^ ppm^−1^. The new AGWP_CO2_ values for 20, 100, and 500 year time horizons are 2.290 × 10^−14^, 8.064 × 10^−14^, and 2.694 × 10^−13^ W m^−2^ yr (kgCO_2_)^−1^, respectively. The AGWP_CO2_(100) value in AR5 (Myhre et al., 2013) and H2013 was about 14% higher, mainly because we updated the impulse response function (accounts for about 8% of the 14% change) and because of a higher atmospheric concentration of CO_2_ which lowers its RE (accounts for ~5%), and slightly because of the new formula from Etminan et al. (2016) (accounts for ~1%). Accounting for all these changes, but including the climate‐carbon feedback for CO_2_, as has been done in much of the prior literature, would give AGWP_CO2_ values which are 3%, 8%, and 13% higher for 20, 100, and 500 year time horizons, respectively.

It is worth highlighting that the impact of increasing CO_2_ mixing ratios on GWP values is the net result of two opposing effects. First, many CO_2_ absorption features are saturated, or close to saturation, and hence the RE of CO_2_ decreases as its mixing ratio increases. Second, the fraction of CO_2_ remaining in the atmosphere (measured by the impulse response function) increases with CO_2_ mixing ratio (see Figure 8.31 in Myhre et al., 2013). The first effect *decreases* AGWP_CO2_ while the second effect *increases* AGWP_CO2_. Hence, GWP calculations for optically thin gases which are defined as AGWP_*X*_/AGWP_CO2_ will change with CO_2_ mixing ratio.

An alternative, the GTP was introduced by Shine et al. (2005). It uses the change in global mean temperature following a pulse emission for a chosen point in time as the impact parameter. While GWP is a metric integrated over time, the GTP is based on the temperature change per unit emissions for a selected year, *t* after the pulse emission. As for the GWP, the impact of CO_2_ is normally used as reference:

$$\mathrm{GTP}{\left(t\right)}_{i}=\text{AGTP}{\left(t\right)}_{i}/\text{AGTP}{\left(t\right)}_{{\mathrm{CO}}_{2}}=\Delta T{\left(t\right)}_{i}/\Delta T{\left(t\right)}_{\mathrm{CO}2},$$

where AGTP (K kg^−1^) is the absolute GTP. The GTP uses the same input as for GWP but in addition includes a temperature response function that represents the thermal inertia of the climate system. AR5 presented values for both GWP and GTP. Here we follow the method used by AR5 (Myhre et al., 2013) and H2013 for calculating GTPs, except that the impulse response function and RE for CO_2_ are updated as explained above and the climate response parameters are updated from Boucher and Reddy (2008) to Geoffroy et al. (2013) (as given in Appendix C of Gasser et al., 2017), which are based on an ensemble of models from the Coupled Model Intercomparison Project phase 5 (CMIP5) (Taylor et al., 2011) and involve a lower climate sensitivity (0.88 compared to 1.1 K (W m^−2^)^−1^ in Boucher and Reddy, 2008). The new AGTP_CO2_ values for 20, 50, and 100 year time horizons are 5.413 × 10^−16^, 4.559 × 10^−16^, and 4.146 × 10^−16^ K (kgCO_2_)^−1^, respectively. Including the climate‐carbon feedback for CO_2_, but keeping all other parameters the same, would give AGTP_CO2_ values which are 5%, 8%, and 11% higher, respectively.

There continues to be a vigorous debate about the applicability of different emission metrics (e.g., Myhre et al., 2013); metric choice depends on the particular policy context in which they are applied, and the degree to which continuity of choice is important in that context (e.g., Allen et al., 2018; Cain et al., 2019; Rogelj & Schleussner, 2019). A specific development has been the suggested use of metrics that compare one‐off pulse emissions of long‐lived gases (such as CO_2_) with step‐changes in emissions of short‐lived species (e.g., gases with lifetimes less than a few decades), on the basis that this leads to a more informed comparison of their ultimate impact on temperature; such approaches can either adopt GWP values, but adapt their usage (Allen et al., 2016) or more directly compute the pulse‐step equivalence (W. J. Collins et al., 2019). In the context of this review, the important point is that all such metrics require the same set of inputs (RE and lifetimes).

It is important to note that the RE and GWP(100) calculations presented here only include the direct effect, while indirect effects can be important for several compounds. Some compounds, and particularly CFCs and halons, influence radiative forcing indirectly through depletion of stratospheric ozone as shown in other work (e.g., Daniel et al., 1995; WMO, 2019). The removal of organic compounds by reaction with OH in the troposphere acts as a source of ozone and prolongs the lifetime of methane, and this has been shown to be important for several hydrocarbons (W. J. Collins et al., 2002; Hodnebrog et al., 2018).

### 2.6 Uncertainties

An overview of estimated contributions to uncertainties associated with the radiative forcing of halocarbons was given in Table 1 of H2013. A total RE uncertainty of ~13% was estimated for compounds with lifetimes longer than about 5 years, and ~23% for compounds with lifetimes shorter than that. The much higher uncertainty for shorter‐lived compounds is caused by the difficulty of estimating nonuniform horizontal and vertical distributions in the atmosphere, which in turn are dependent on the location of emissions (see section 2.4).

Table 1 gives updated estimates of contributions to the total radiative forcing uncertainties. As in H2013, the uncertainty estimates are based on published literature and subjective judgment and we estimate the total uncertainty to be valid for a 5% to 95% (90%) confidence range. The total RF uncertainty, calculated using the root‐sum‐square (RSS) method, is ~14% and 24% for compounds with lifetimes longer and shorter than ~5 years, respectively. These total RF uncertainties are slightly higher than in H2013 and explanations are given below.

**Table 1:** Estimated Contributions to the Total Radiative Forcing Uncertainty

Source of uncertainty	Estimated contribution to total RF uncertainty	References used as basis for uncertainty estimates
Experimental absorption cross‐sections	~5%	Ballard et al. (2000a),Bravo et al. (2010), and Forster et al. (2005)
‐neglected far infrared bands	~3%	
‐neglected shortwave bands	~5%	
Radiation scheme	~5%	W. D. Collins et al. (2006), Forster et al. (2005), and Oreopoulos et al. (2012)
Clouds	~5%	Forster et al. (2005) and Gohar et al. (2004)
Spectral overlap and water vapor distribution	~3%	Forster et al. (2005), Jain et al. (2000), and Pinnock et al. (1995)
Surface emissivity and temperature, and atmospheric temperature	~5%	Forster et al. (2005)
Tropopause level	~5%	Forster et al. (2005),Freckleton et al. (1998), and Myhre and Stordal (1997)
Temporal and spatial averaging	~1%	Freckleton et al., 1998, and Myhre and Stordal (1997)
Stratospheric temperature adjustment	~2%	Forster et al. (2005), Gohar et al. (2004), and Shine and Myhre (2020)
Nonuniform vertical profile	~5% for lifetimes > ~5 years,	Hodnebrog et al. (2013) and Sihra et al. (2001)
	~20% for lifetimes < ~5 years	
Total (RSS)	~14% for lifetimes > ~5 years	
	~24% for lifetimes < ~5 years	

One issue with the use of laboratory data is that it does not always cover the entire spectral range for which radiative forcing is important (see, e.g., Figure 3). For example, the PNNL measurements mostly cover the 600–6,500 cm^−1^ wave number range, and so their use would neglect any absorption (and hence forcing) at lower wave numbers, although in general it extends to much higher wave numbers than those in other data sets.

The uncertainty due to lack of spectral data at low wave numbers cannot be assessed for every gas in our analysis, but there is some evidence to indicate its typical size. Highwood and Shine (2000) computed the contribution of wave numbers less than 700 cm^−1^ to the RE for HFC‐134a and found it contributed around 2% to the forcing. Bravo et al. (2010) presented an analysis of the RE due to a set of seven perfluorocarbons. They compared the RE calculated using ab initio methods for the wave number interval 0–2,500 cm^−1^ with calculations for the wave number interval 700–1,400 cm^−1^, chosen because it coincided with the wave number range for their associated laboratory measurements. Most of the additional absorption was at wave numbers below 700 cm^−1^. They found that the integrated absorption cross sections and REs for the narrow range were within 2% for the lighter PFCs, but this difference increased to 10% for heavier PFCs. Since many of the measured data sets (e.g., the PNNL data) use a broader wavelength range than 700–1,400 cm^−1^, it is unlikely that our estimates are systematically in error by such a large amount. Nevertheless, we introduce an additional generic uncertainty to our estimates, which was not included in the analysis of H2013, of ~3% due to neglected bands (Table 1); clearly this could be systematically investigated in future work, perhaps by including ab initio calculations outside the range of measured cross sections.

Another source of uncertainty not considered in H2013 is the contribution to RE from absorption of shortwave (SW), or solar, radiation in the near‐infrared (3,000 to 14,000 cm^−1^). There has been renewed interest in the SW forcing due to methane (e.g., W. D. Collins et al., 2018; Etminan et al., 2016). Etminan et al. (2016) find the direct effect of methane's near‐IR bands enhances its forcing by 6% but there is an additional 9% impact via the effect of this absorption on stratospheric temperatures (and hence on longwave forcing). This contrasts with the impact of the near‐IR bands of CO_2_ which cause a decrease of a few percent, because much of the additional forcing is at higher altitudes. The contribution of these near‐IR bands to RE is further complicated by the fact that it depends strongly on the overlap between these bands and those of water vapor (Etminan et al., 2016), many of which are saturated for typical atmospheric paths, making generic statements difficult.

The potential impact of SW absorption is difficult to constrain for the diverse range of gases discussed here, without much more detailed study, not least because many of the experimental data sets do not extend to such high wave numbers (the PNNL data are a notable exception). For the heavier halogenated gases, the strongest fundamental and combination bands will generally be at lower wave numbers, at which SW absorption is less important (see, e.g., Bera et al., 2009). The lighter, more hydrogenated, gases, will have more significant absorption bands in the solar near‐infrared but, on the other hand, these gases are likely to be much shorter‐lived, so that their impact on stratospheric temperatures is likely to be lower. We introduce an additional uncertainty of ~5% due to the potential effect of this shortwave absorption (Table 1).

Since H2013, surface emissivity has been included as a source of uncertainty together with surface temperature and atmospheric temperature, and consequently the estimated contribution to RF uncertainty has been increased from ~3% to ~5% (Table 1). The stratospheric temperature adjustment is now based on a much more sophisticated method compared to the generic 10% increase used in H2013 (see section 2.3), and we have lowered the uncertainty contribution for this term from ~4% to ~2%. The remaining sources of uncertainties and their estimated contributions given in Table 1 are unchanged, and we refer to H2013 for detailed explanations of each term.

Uncertainties in the atmospheric lifetime of the compounds are also important for metric calculations, and since H2013, SPARC (2013) have provided recommended lifetime values and uncertainties for a range of halocarbons. Their estimates are derived using atmospheric chemistry transport and inverse modeling, and analysis of atmospheric observations and laboratory measurements. Possible uncertainty ranges for most of the compounds in SPARC (2013) have been evaluated in Velders and Daniel (2014; their Table 1) and range from ±3% to ±33% (1 standard deviation), depending on the compound; they are typically in the range from ±15% to ±20% (or ±25% to ±33% when converted from 1 standard deviation to 5–95% (90%) confidence range). However, Velders and Daniel (2014) point out that the possible uncertainty range is likely an overestimation of the true uncertainty and the most likely range, given for some of the compounds, is substantially lower (±12% to ±20% when converted from 1 standard deviation to 5–95% (90%) confidence range).

GWP uncertainties are affected by uncertainties in the compound's lifetime, RE and the AGWP_CO2_, and uncertainties in GWP and/or GTP have been investigated in previous studies (Boucher, 2012; Hodnebrog et al., 2013; Olivié & Peters, 2013; Reisinger et al., 2010; Velders & Daniel, 2014; Wuebbles et al., 1995). H2013 (see their section 3.6.4) estimated GWP(100) uncertainties of ±38% and ±34% (5–95% (90%) confidence) for CFC‐11 and HFC‐134a, respectively. GWP(100) uncertainties for six HFCs in WMO (2015; their Tables 5 and 6) were approximately in the range 30–50%, which is similar to the GWP(100) uncertainties for several ozone‐depleting substances given in Velders and Daniel (2014) (their Table 4). We estimate that the uncertainties given in H2013, WMO (2015) and Velders and Daniel (2014) (approximately in the range 30–50%) are similar for the GWP(100) values calculated here and are probably also representative for most other halocarbons with similar or longer lifetimes.

## 3 Results and Discussion

### 3.1 Updated Spectra, REs, and GWPs for the Most Abundant Halocarbons and Related Compounds

This section broadly follows the structure of section 4.1 in H2013, where absorption cross sections and radiative efficiency estimates in the literature were reviewed and new RE and GWP calculations were presented. However, we limit this section to only include studies and spectra that were not included in H2013, and only to the 40 most abundant halocarbons presented in Table 7 of Meinshausen et al. (2017) (see section 3.3 for other compounds). Also, only experimental spectra are used as a basis for our calculations here, unlike H2013 which included RE and GWP calculations for some compounds where only calculated spectra existed. In cases where spectra have been measured at different temperatures, we have used the spectra closest to room temperature (see section 2.2 for a discussion of temperature dependence of cross sections). All REs are given for all‐sky and with stratospheric temperature adjustment included (see section 2.3). The lifetime correction method from H2013, to account for a nonhomogeneous vertical and horizontal distribution in the atmosphere, has been applied to the calculated REs (see section 2.4).

Table 2 lists absorption cross sections that are new since H2013 and Tables S1–S6 in the supporting information list all (to the best of our knowledge) absorption cross sections and reported RE values from the literature. Tables S1–S6 also include calculations using the Pinnock curve from H2013 for easier identification of possible changes in RE that are due to the updated Pinnock curve from Shine and Myhre (2020). We have followed the International Union of Pure and Applied Chemistry, IUPAC, naming scheme and included the unique Chemical Abstract Service Registry Number, CASRN, for each compound listed in the tables. Table 3 presents updated atmospheric lifetimes, REs, and GWP(100) values and discussions of the results are given below for each group of compounds. RE values with more significant figures, needed to reproduce the GWP(100) values, are given in the supporting information.

**Table 2:** Integrated Infrared Absorption Cross‐Section Updates (*S*) Since the H2013 Review for the 40 Most Abundant Halocarbons and Related Compounds in the Atmosphere

Name	CASRN	Identifier	Formula^a^	*T* (K)	Wn. range (cm^−1^)	*S* ^b^	Reference	Database^c^	New^d^
***Chlorofluorocarbons***									
Trichlorofluoromethane	75‐69‐4	CFC‐11	CCl_3_F	298	570–3,000	10.1	**Sharpe et al.** **(** **2004** **)**	H16	S
Dichlorodifluoromethane	75‐71‐8	CFC‐12	CCl_2_F_2_	294	800–1,270	13.5	**Harrison** **(** **2015a** **)**	H16	S
				296	600–3,000	13.9	**Sharpe et al.** **(** **2004** **)**	P	S
1,1,2‐Trichloro‐1,2,2‐trifluoroethane	76‐13‐1	CFC‐113	CCl_2_FCClF_2_	298	620–3,000	14.6	**Sharpe et al.** **(** **2004** **)**	H16	S
1,2‐Dichloro‐1,1,2,2‐tetrafluoroethane	76‐14‐2	CFC‐114	CClF_2_CClF_2_	298	600–3,000	17.4	**Sharpe et al.** **(** **2004** **)**	H16	S
1‐Chloro‐1,1,2,2,2‐pentafluoroethane	76‐15‐3	CFC‐115	CClF_2_CF_3_	296	946–1,368	11.9	**Totterdill et al.** **(** **2016** **)**		B
				296	525–3,000	20.1	**Sharpe et al.** **(** **2004** **)**	P	S
***Hydrochlorofluorocarbons***									
Chlorodifluoromethane	75‐45‐6	HCFC‐22	CHClF_2_	295	730–1,380	10.5	**Harrison** **(** **2016** **)**	H16	S
				296	550–3,000	10.8	**Sharpe et al.** **(** **2004** **)**	P	S
1,1‐Dichloro‐1‐fluoroethane	1717‐00‐6	HCFC‐141b	CH_3_CCl_2_F	295	705–1,280		Harrison (2019)		L
				283	570–1,470	8.0	**Le Bris et al.** **(** **2012** **)**		S
				298	550–3,000	8.4	**Sharpe et al.** **(** **2004** **)**	H16	S
1‐Chloro‐1,1‐difluoroethane	75‐68‐3	HCFC‐142b	CH_3_CClF_2_	283	650–1,500	10.7	**Le Bris and Strong** **(** **2010** **)**	H16	S
				298	600–3,000	11.2	**Sharpe et al.** **(** **2004** **)**	H16	S
***Hydrofluorocarbons***									
Trifluoromethane	75‐46‐7	HFC‐23	CHF_3_	294	950–1,500	12.3	**Harrison** **(** **2013** **)**	H16	S
				296	600–3,000	12.7	**Sharpe et al.** **(** **2004** **)**	P	S
Difluoromethane	75‐10‐5	HFC‐32	CH_2_F_2_	298	510–3,000	7.0	**Sharpe et al.** **(** **2004** **)**	H16	S
1,1,1,2,2‐Pentafluoroethane	354‐33‐6	HFC‐125	CHF_2_CF_3_	298	510–3,000	17.4	**Sharpe et al.** **(** **2004** **)**	H16	S
1,1,1,2‐Tetrafluoroethane	811‐97‐2	HFC‐134a	CH_2_FCF_3_	296	750–1,600	13.2	Harrison (2015b)	H16	S
				296	600–3,000	14.2	**Sharpe et al.** **(** **2004** **)**	P	S
1,1,1‐Trifluoroethane	420‐46‐2	HFC‐143a	CH_3_CF_3_	296	570–1,500	13.8	**Le Bris and Graham** **(** **2015** **)**	H16	B
				298	500–3,000	13.9	**Sharpe et al.** **(** **2004** **)**	H16	S
1,1‐Difluoroethane	75‐37‐6	HFC‐152a	CH_3_CHF_2_	298	525–3,000	8.0	**Sharpe et al.** **(** **2004** **)**	H16	S
1,1,1,2,3,3,3‐Heptafluoropropane	431‐89‐0	HFC‐227ea	CF_3_CHFCF_3_	298	500–3,000	25.3	**Sharpe et al.** **(** **2004** **)**	H16	S
1,1,1,2,2,3,4,5,5,5‐Decafluoropentane	138495‐42‐8	HFC‐43‐10mee	CF_3_CHFCHFCF_2_CF_3_	305	550–1,600	30.1	**Le Bris et al.** **(** **2018** **)**		B
				298	500–3,000	30.4	**Sharpe et al.** **(** **2004** **)**	H16	S
***Chlorocarbons and Hydrochlorocarbons***									
1,1,1‐Trichloroethane	71‐55‐6	Methyl chloroform	CH_3_CCl_3_	298	500–3,000	5.3	**Sharpe et al.** **(** **2004** **)**	H16	S
Tetrachloromethane	56‐23‐5	Carbon tetrachloride	CCl_4_	296	700–860	6.7	Harrison et al. (2017)	H16	S
				295–8	730–825	6.3	**Wallington et al.** **(** **2016** **)**		B
				298	730–825	6.4	Sharpe et al. (2004)	P	L
Chloromethane	74‐87‐3	Methyl chloride	CH_3_Cl	295–8	660–1,620	0.8	**Wallington et al.** **(** **2016** **)**		B
				296	600–3000	1.3	Sharpe et al. (2004)	P	S
Dichloromethane	75‐09‐2	Methylene chloride	CH_2_Cl_2_	295–8	650–1,290	2.6	**Wallington et al.** **(** **2016** **)**		B
				298	600–3,000	2.8	Sharpe et al. (2004)	H16	S
Trichloromethane	67‐66‐3	Chloroform	CHCl_3_	295–8	720–1,245	4.4	**Wallington et al.** **(** **2016** **)**		B
				298	580–3,000	5.0	Sharpe et al. (2004)	H16	S
***Bromocarbons, hydrobromocarbons and halons***									
Bromomethane	74‐83‐9	Methyl bromide	CH_3_Br	296	550–3,000	1.1	**Sharpe et al.** **(** **2004** **)**	P	S
Bromochlorodifluoromethane	353‐59‐3	Halon‐1211	CBrClF_2_	298	600–3,000	13.2	**Sharpe et al.** **(** **2004** **)**	H16	S
Bromotrifluoromethane	75‐63‐8	Halon‐1301	CBrF_3_	298	510–3,000	16.1	**Sharpe et al.** **(** **2004** **)**	H16	S
1,2‐Dibromo‐1,1,2,2‐tetrafluoroethane	124‐73‐2	Halon‐2402	CBrF_2_CBrF_2_	298	550–3,000	16.1	**Sharpe et al.** **(** **2004** **)**	H16	S
***Fully fluorinated species***									
Nitrogen trifluoride	7783‐54‐2		NF_3_	296	600–1,970	7.3	**Totterdill et al.** **(** **2016** **)**		B
				298	600–3,000	7.2	**Sharpe et al.** **(** **2004** **)**	H16	S
Sulfur hexafluoride	2551‐62‐4		SF_6_	295	650–2,000	24.0	Kovács et al. (2017)		L
				298	560–3,000	21.2	**Sharpe et al.** **(** **2004** **)**	H16	S
Sulfuryl fluoride	2699‐79‐8		SO_2_F_2_	298	500–3,000	14.0	**Sharpe et al.** **(** **2004** **)**	H16	S
Tetrafluoromethane	75‐73‐0	PFC‐14	CF_4_	298	570–3,000	19.8	**Sharpe et al.** **(** **2004** **)**	H16	S
Hexafluoroethane	76‐16‐4	PFC‐116	C_2_F_6_	298	500–3,000	23.1	**Sharpe et al.** **(** **2004** **)**	H16	S
Octafluoropropane	76‐19‐7	PFC‐218	C_3_F_8_	298	600–3,000	27.5	**Sharpe et al.** **(** **2004** **)**	H16	S
Octafluorocyclobutane	115‐25‐3	PFC‐C 318	cyc (‐CF_2_CF_2_CF_2_CF_2_‐)	298	550–3,000	21.7	**Sharpe et al.** **(** **2004** **)**	H16	S
Decafluorobutane	355‐25‐9	PFC‐31‐10	n‐C_4_F_10_	298	500–3,000	32.4	**Sharpe et al.** **(** **2004** **)**	H16	S
Dodecafluoropentane	678‐26‐2	PFC‐41‐12	n‐C_5_F_12_	278	500–3,000	37.3	**Sharpe et al.** **(** **2004** **)**	H16	S

*Note*. Spectra used in the present RE calculations are indicated in bold (see Tables S1–S6 in the supporting information for a complete list of spectra used in RE calculations).

^a^ cyc, cyclic compound.

^b^ Integrated absorption cross section given in units of 10^−17^ cm^2^ molecule^−1^ cm^−1^.

^c^ Absorption cross section downloaded from database: H16, HITRAN 2016; P, PNNL.

^d^ New data since H2013: L, literature; S, spectrum; B, both.

**Table 3:** Lifetimes (*τ*), Radiative Efficiencies and Direct Effect GWPs (Relative to CO_2_) for the 40 Most Abundant Halocarbons and Related Compounds in the Atmosphere

			*τ* (yr)		RE (W m^−2^ ppb^−1^)		GWP(100)	
Identifier/name	Formula	CASRN	H2013^a^	WMO (2019)	H2013	This work	H2013	This work
***Chlorofluorocarbons***								
**CFC‐11**	CCl_3_F	75‐69‐4	45.0	52.0	**0.26**	0.26	4,660	**5,870**
**CFC‐12**	CCl_2_F_2_	75‐71‐8	100.0	102.0	**0.32**	0.32	10,200	**11,800**
**CFC‐113**	CCl_2_FCClF_2_	76‐13‐1	85.0	93.0	**0.30**	0.30	5,820	**6,900**
**CFC‐114**	CClF_2_CClF_2_	76‐14‐2	190.0	189.0	**0.31**	0.31	8,590	**9,990**
**CFC‐115**	CClF_2_CF_3_	76‐15‐3	1020.0	540.0	0.20	**0.25**	7,670	**10,200**
***Hydrochlorofluorocarbons***								
**HCFC‐22**	CHClF_2_	75‐45‐6	11.9	11.9	**0.21**	0.21	1,770	**2,060**
**HCFC‐141b**	CH_3_CCl_2_F	1717‐00‐6	9.2	9.4	**0.16**	0.16	782	**903**
**HCFC‐142b**	CH_3_CClF_2_	75‐68‐3	17.2	18.0	**0.19**	0.19	1,980	**2,410**
***Hydrofluorocarbons***								
**HFC‐23**	CHF_3_	75‐46‐7	222.0	228.0	0.17	**0.19**	12,400	**15,500**
**HFC‐32**	CH_2_F_2_	75‐10‐5	5.2	5.4	**0.11**	0.11	677	**809**
**HFC‐125**	CHF_2_CF_3_	354‐33‐6	28.2	30.0	**0.23**	0.23	3,170	**3,940**
**HFC‐134a**	CH_2_FCF_3_	811‐97‐2	13.4	14.0	0.16	**0.17**	1,300	**1,600**
**HFC‐143a**	CH_3_CF_3_	420‐46‐2	47.1	51.0	0.16	**0.17**	4,800	**6,130**
**HFC‐152a**	CH_3_CHF_2_	75‐37‐6	1.5	1.6	**0.10**	0.10	138	**172**
**HFC‐227ea**	CF_3_CHFCF_3_	431‐89‐0	38.9	36.0	0.26	**0.27**	3,350	**3,800**
HFC‐236fa	CF_3_CH_2_CF_3_	690‐39‐1	242.0	213.0	0.24	**0.25**	8,060	**9,210**
HFC‐245fa	CHF_2_CH_2_CF_3_	460‐73‐1	7.7	7.9	**0.24**	0.24	858	**1,010**
HFC‐365mfc	CH_3_CF_2_CH_2_CF_3_	406‐58‐6	8.7	8.9	0.22	**0.23**	804	**959**
**HFC‐43‐10mee**	CF_3_CHFCHFCF_2_CF_3_	138495‐42‐8	16.1	17.0	0.42	**0.36**	1,650	**1,680**
***Chlorocarbons and hydrochlorocarbons***								
**1,1,1‐Trichloroethane**	CH_3_CCl_3_	71‐55‐6	5.0	5.0	0.07	**0.06**	160	**169**
**Tetrachloromethane**	CCl_4_	56‐23‐5	26.0	32.0	**0.17**	0.17	1,730	**2,310**
**Chloromethane**	CH_3_Cl	74‐87‐3	1.0	0.9	0.010	**0.005**	12	**6**
**Dichloromethane**	CH_2_Cl_2_	75‐09‐2	0.4	0.5	**0.03**	0.03	9	**12**
**Trichloromethane**	CHCl_3_	67‐66‐3	0.4	0.5	0.08	**0.07**	16	**22**
***Bromocarbons, hydrobromocarbons and halons***								
**Bromomethane**	CH_3_Br	74‐83‐9	0.8	0.8	0.005	**0.004**	2	**3**
**Halon‐1211**	CBrClF_2_	353‐59‐3	16.0	16.0	0.29	**0.30**	1,750	**2,030**
**Halon‐1301**	CBrF_3_	75‐63‐8	65.0	72.0	**0.30**	0.30	6,290	**7,600**
**Halon‐2402**	CBrF_2_CBrF_2_	124‐73‐2	20.0	28.0	**0.31**	0.31	1,470	**2,280**
***Fully fluorinated species***								
**Nitrogen trifluoride**	NF_3_	7783‐54‐2	500.0	569.0	**0.20**	0.20	16,100	**18,500**
**Sulfur hexafluoride**	SF_6_	2551‐62‐4	3200.0	3200.0	**0.57**	0.57	23,500	**26,700**
**Sulfuryl fluoride**	SO_2_F_2_	2699‐79‐8	36.0	36.0	0.20	**0.21**	4,100	**4,880**
**PFC‐14**	CF_4_	75‐73‐0	50000.0	50000.0	**0.10**	0.10	6,630	**7,830**
**PFC‐116**	C_2_F_6_	76–16‐4	10000.0	10000.0	0.25	**0.26**	11,100	**13,200**
**PFC‐218**	C_3_F_8_	76‐19‐7	2600.0	2600.0	0.28	**0.27**	8,900	**9,850**
**PFC‐C‐318**	c‐C_4_F_8_	115‐25‐3	3200.0	3200.0	0.32	**0.31**	9,550	**10,800**
**PFC‐31‐10**	n‐C_4_F_10_	355‐25‐9	2600.0	2600.0	0.36	**0.37**	9,200	**10,600**
**PFC‐41‐12**	n‐C_5_F_12_	678‐26‐2	4100.0	4100.0	**0.41**	0.41	8,550	**9,780**
PFC‐51‐14	n‐C_6_F_14_	355‐42‐0	3100.0	3100.0	0.44	**0.45**	7,910	**9,140**
PFC‐61‐16	n‐C_7_F_16_	335‐57‐9	3000.0	3000.0	**0.50**	0.50	7,820	**8,920**
PFC‐71‐18	n‐C_8_F_18_	307‐34‐6	3000.0	3000.0	0.55	**0.56**	7,620	**8,760**

*Note*. Compounds where the radiative efficiencies are based on new spectra since the H2013 review are marked in bold. Recommended RE and GWP(100) values are indicated in bold. Lifetimes are taken from WMO (2019). Note that RE values with more significant digits have been used to calculate GWP(100) and that these are available in the supporting information.

^a^ Lifetimes in H2013 were from WMO (2011) except for PFC‐71‐18.

#### 3.1.1 Chlorofluorocarbons

Since H2013, new spectra have been included for the five most‐abundant CFCs, but the RE remains unchanged for four of the compounds (Tables 2 and 3). CFC‐115 now has a much larger RE than in H2013 (0.25 compared to 0.20 W m^−2^ ppb^−1^) due to the addition of spectra from the PNNL database (Sharpe et al., 2004). In H2013, and in two out of four previous studies (Jain et al., 2000; Myhre & Stordal, 1997), the CFC‐115 spectrum used is that from McDaniel et al. (1991), which has an integrated absorption cross‐section of 1.21 × 10^−16^ cm^2^ molecule^−1^ cm^−1^ and gives an RE of 0.20 W m^−2^ ppb^−1^ in our calculations (Table S1). Recently, Totterdill et al. (2016) measured the IR absorption spectrum of CFC‐115 and performed detailed LBL radiative transfer calculations to determine its RE. Their integrated absorption spectrum of 1.19 × 10^−16^ cm^2^ molecule^−1^ cm^−1^ is in relatively good agreement with McDaniel et al. (1991) and their resulting RE of 0.21 W m^−2^ ppb^−1^ agrees well with H2013. The PNNL spectrum for CFC‐115 has a much higher integrated absorption cross section of 2.01 × 10^−16^ cm^2^ molecule^−1^ cm^−1^ and our calculations give a RE of 0.32 W m^−2^ ppb^−1^. A comparison between the McDaniel et al. (1991) and PNNL absorption spectra shows that the locations and relative strength of the main absorption bands are similar, but that the overall magnitude of the bands are higher in the PNNL spectrum (not shown). Due to the large difference between the two spectra, we have also inspected the PNNL spectra measured at different temperatures (278 and 323 K), and these have similar integrated absorption cross sections and yield similar RE values as the 296 K PNNL spectrum (Table S1), and so give no indication of error in the 296 K PNNL spectra. A fourth source for CFC‐115 spectra is Fisher et al. (1990) who report an integrated absorption cross section of 1.74 × 10^−16^ cm^2^ molecule^−1^ cm^−1^, which is higher than McDaniel et al. (1991) and lower than (but nearer to) PNNL. Reasons for the large difference between the spectra remain unknown. We have calculated our new RE value of 0.25 W m^−2^ ppb^−1^ by averaging the RE values based on the three available spectra (McDaniel et al., 1991; Sharpe et al., 2004; Totterdill et al., 2016).

**Figure 4:**
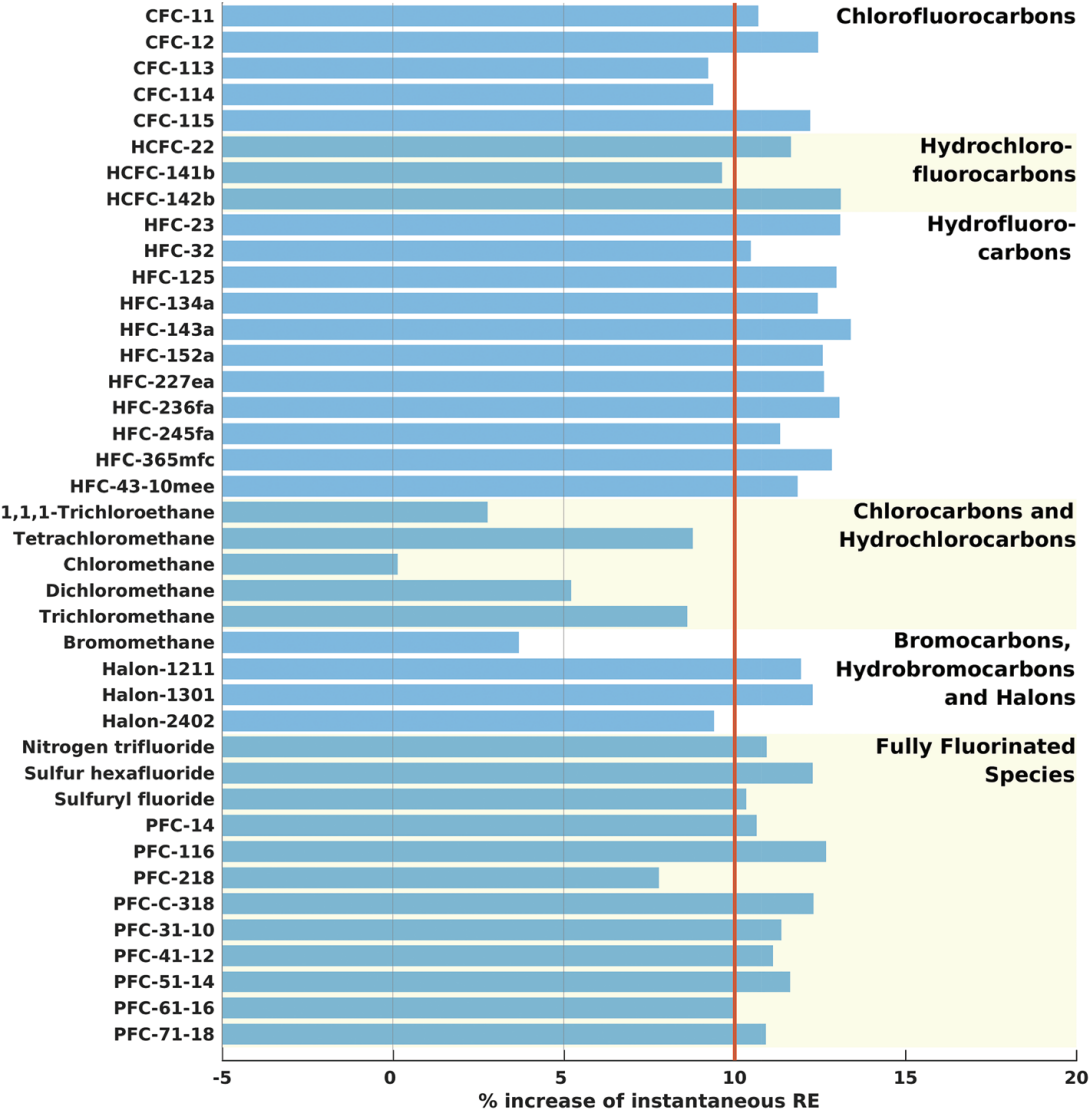
Stratospheric temperature adjustment represented as the % increase of the instantaneous RE for 40 abundant compounds. The red line shows the 10% assumption used in H2013 for nearly all compounds (note that the 10% assumption was not used for CFC‐11, CFC‐12, and PFC‐14).

**Figure 5:**
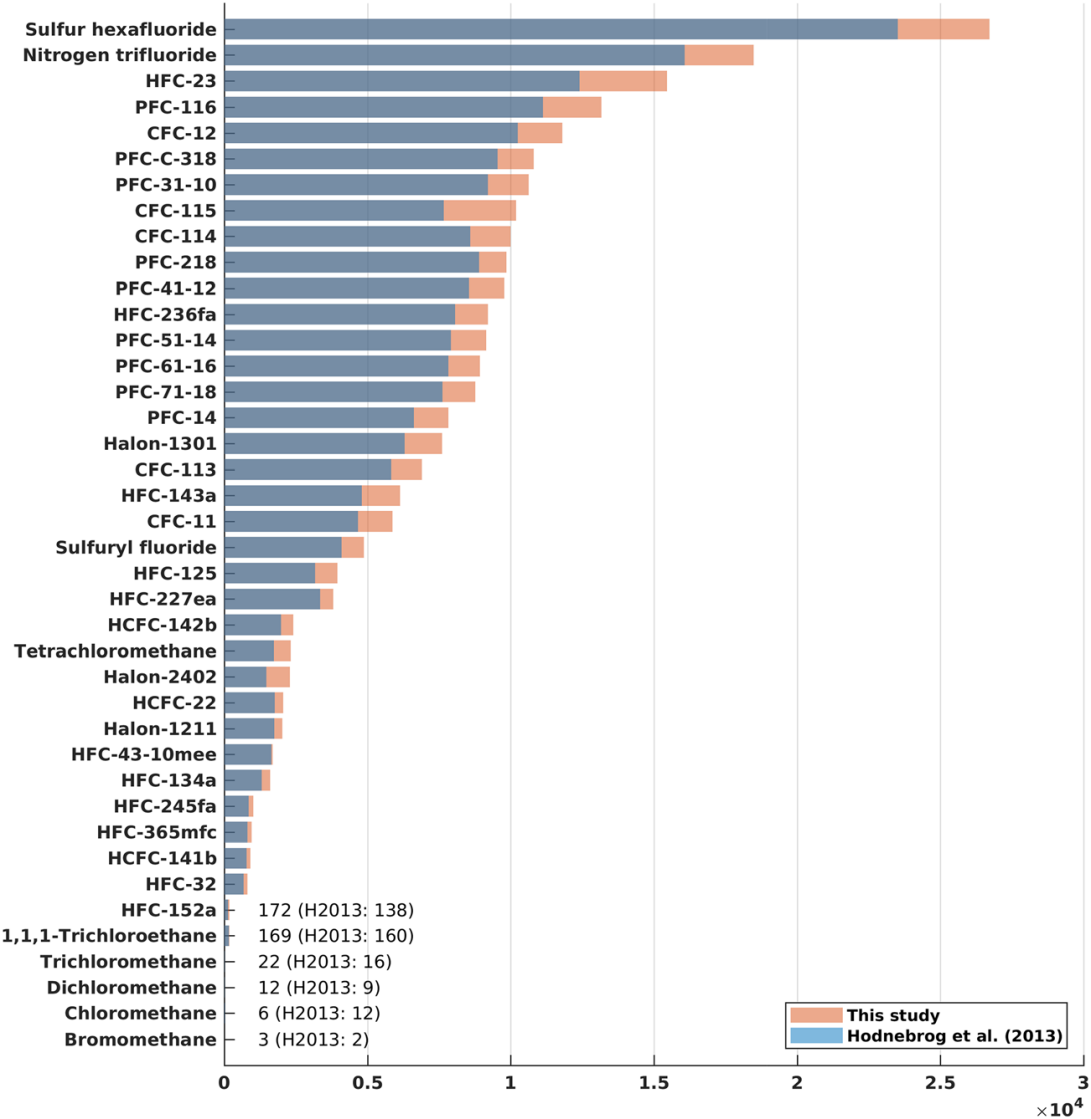
GWP(100) ranking calculated in this study and from H2013 for 40 most abundant compounds. Note that only one compound (chloromethane) shows a decrease in GWP(100).

The stratospheric temperature adjustment for the CFCs ranges from 9% to 12% increase of the instantaneous RE, and the generic 10% increase used in H2013 was a relatively good approximation for these compounds (Figure 4). (Note that the 10% assumption was not used for CFC‐11 and CFC‐12 in H2013.) The atmospheric lifetimes of the five CFCs have been updated based on WMO (2019) since H2013, most notably for CFC‐11 (52 vs. 45 years in H2013) and CFC‐115 (540 vs. 1,020 years in H2013). A combination of updated lifetimes, REs, and the AGWP_CO2_ leads to higher GWP(100) values for all five CFCs (Table 3 and Figure 5).

#### 3.1.2 Hydrochlorofluorocarbons

Six new spectra have been included for the three most‐abundant HCFCs in this category, but their REs are unchanged when rounded to two decimals (Tables 2 and 3). The updated AGWP_CO2_, and slightly longer lifetimes for two of the compounds (HCFC‐141b and HCFC‐142b), contribute to higher GWP(100) (Tables 3 and S2 and Figure 5).

#### 3.1.3 Hydrofluorocarbons

Since H2013, spectra have been added to eight of the 11 most‐abundant HFC compounds (Table 2) and in most cases this led to little or no change in the RE (Table 3). For HFC‐23, the two new spectra (Harrison, 2013; Sharpe et al., 2004) each have higher integrated absorption cross sections than the two spectra used in H2013 (Table S3); this leads to a higher RE for this compound (0.19 compared to 0.17 W m^−2^ ppb^−1^ in H2013). Another contributing factor is the stratospheric temperature adjustment. The RE is now 13% higher than the instantaneous RE for HFC‐23 (Figure 4), while in H2013 a generic 10% increase was used. In fact, all 11 HFC compounds have stratospheric temperature adjustments larger than 10% and most of them around 13%.

For HFC‐43‐10mee, the H2013 RE value of 0.42 W m^−2^ ppb^−1^ was not calculated using new spectra but was based on the RE given in the fourth assessment report (AR4) (Forster et al., 2007), which was again based on personal communication with D. A. Fisher in IPCC (1994). Recently, Le Bris et al. (2018) measured the absorption cross section and calculated a much lower RE of 0.36 W m^−2^ ppb^−1^ for HFC‐43‐10mee when using the method in H2013 (Table S3). They also showed that the RE calculated with their spectrum agreed very well with that calculated from the PNNL spectrum. Here, we have used the spectra from both Le Bris et al. (2018) and the PNNL database and calculated a RE of 0.36 W m^−2^ ppb^−1^ (Table 3), in excellent agreement with Le Bris et al. (2018).

Updated GWP(100) values are higher for all HFCs (Table 3 and Figure 5), and this is due to a combination of updated AGWP_CO2_, higher RE values for several compounds (HFC‐43‐10mee is a notable exception), and longer lifetimes for all compounds except HFC‐227ea and HFC‐236fa.

#### 3.1.4 Chlorocarbons and Hydrochlorocarbons

Nine new spectra have been added for the five most‐abundant compounds since H2013 (Table 2). Wallington et al. (2016) made new measurements of the absorption spectra of the chloromethanes CH_3_Cl, CH_2_Cl_2_, CHCl_3_, and CCl_4_, and provided recommended spectra for these compounds by combining existing and new experimental data. We have used their recommended spectra to calculate REs for all four chloromethanes (see Text S4 for an explanation of the choice of spectra). The resulting RE for CCl_4_ of 0.17 W m^−2^ ppb^−1^ is unchanged since H2013 (Tables 3 and S4), where the spectrum from Nemtchinov and Varanasi (2003) was used. The RE of CHCl_3_ is lower than in H2013 (0.07 vs. 0.08 W m^−2^ ppb^−1^), where the spectrum from Vander Auwera (2000) was used. For CH_3_Cl and CH_2_Cl_2_, new RE calculations were not carried out for H2013 but retained from IPCC AR4 (Forster et al., 2007). Our calculations using the Wallington et al. (2016) spectrum show that the RE value of 0.03 W m^−2^ ppb^−1^ for CH_2_Cl_2_ is unchanged since H2013 when rounded to two decimal places. The RE of CH_3_Cl is now 0.005 W m^−2^ ppb^−1^, which is lower than the 0.01 W m^−2^ ppb^−1^ value in H2013 (which originated from AR4), but in excellent agreement with the original instantaneous RE value of 0.005 W m^−2^ ppb^−1^ from Grossman et al. (1997).

For CH_3_CCl_3_, we added the spectrum from the HITRAN 2016 database, which was again adopted from the PNNL database, and calculate a lower RE value compared to H2013 (0.06 vs. 0.07 W m^−2^ ppb^−1^) (Tables 2 and 3). The addition of the new spectrum did not change the RE, but the updated Pinnock curve and particularly the method to account for stratospheric temperature adjustment (see section 2.3) led to the lower value (Table S4). For all five compounds in this group, the stratospheric temperature adjustment is lower than the generic 10% increase used in H2013, and ranges from 0% change to a 9% increase of the instantaneous RE (Figure 4). GWP(100) values are lower for CH_3_Cl, and higher for the remaining four compounds (Table 3 and Figure 5). Lifetime updates for four of the compounds contribute to the changes in GWP 100‐year values.

#### 3.1.5 Bromocarbons, Hydrobromocarbons, and Halons

Since H2013, absorption spectra from the HITRAN 2016 and PNNL databases have been included in the RE calculations for each of the four most‐abundant compounds (Table 2). Changes in RE since H2013 are negligible (<5%) for the three halons, while CH_3_Br shows a lower RE (0.004 vs. 0.005 W m^−2^ ppb^−1^) (Table 3), mainly because the stratospheric temperature adjustment is lower (~4%) compared to the generic 10% increase used in H2013 (Figure 4). Since H2013, lifetimes are longer for Halon‐1301 and Halon‐2402 while GWP(100) values are higher for all four compounds (Tables 3 and S5 and Figure 5).

#### 3.1.6 Fully Fluorinated Species

For 9 of the 12 most‐abundant compounds, spectra have been added from the HITRAN 2016 database (where spectra were again adopted from the PNNL database) since H2013 (Table 2). Still, the calculated RE values for all these compounds are relatively similar to those reported in H2013 (Table 3). Sulfuryl fluoride shows the largest change of around 5%, mainly due to a slightly higher integrated absorption cross section in the new PNNL spectrum compared to that of Andersen et al. (2009), which was used in H2013 (Table S6). This is in turn partly because the PNNL spectrum also includes a weak absorption band around 550 cm^−1^ (not shown).

For NF_3_, two new spectra have been added since H2013 and the calculated RE value is now based on three different spectra (Robson et al., 2006; Sharpe et al., 2004; Totterdill et al., 2016) (Tables 2, 3, and S6). The RE value of 0.20 W m^−2^ ppb^−1^ is the same as in H2013, but the RE of 0.25 W m^−2^ ppb^−1^ presented in Totterdill et al. (2016) is substantially higher (>20%). Totterdill et al. (2016) attribute the differences to a higher integrated absorption cross section compared to Robson et al. (2006) (which was used to calculate the RE value in H2013 and AR5), but our RE calculation differs by less than 5% when using spectra from each of the two studies separately (Table S6) so this is only part of the reason. Other potential reasons include differences between the radiative transfer models, treatment of clouds, and stratospheric temperature adjustment.

The RE of SF_6_ has had a relatively wide range in reported literature values from 0.49 W m^−2^ ppb^−1^ (Jain et al., 2000) to 0.68 W m^−2^ ppb^−1^ (H. Zhang et al., 2011) (Table S6). Since H2013, Kovács et al. (2017) have made new measurements of the SF_6_ absorption spectrum and used a LBL model to calculate a RE value of 0.59 W m^−2^ ppb^−1^. Their spectrum is not included here, but their RE value is close to our calculated RE value of 0.57 W m^−2^ ppb^−1^ using spectra from the HITRAN and PNNL databases; this value was also presented in H2013 and used in AR5.

The stratospheric temperature adjustment for the fully fluorinated species ranges from 8% to 13% increase of the instantaneous RE (Figure 4). For most of these compounds, the generic 10% increase used in H2013 was a relatively good approximation for stratospheric temperature adjustment (note that the 10% assumption was not used for PFC‐14 in H2013).

GWP(100) values are higher than in H2013 for all compounds in this category (Tables 3 and S6 and Figure 5), mainly due to the updated AGWP_CO2_. The only lifetime change since H2013 is for NF_3_, which has a longer lifetime of 569 years compared to the value of 500 years that was used earlier. While we have adopted atmospheric lifetimes from WMO (2019), we note that two recent studies have calculated substantially shorter lifetimes for SF_6_ than the widely used estimate of 3,200 years (Ravishankara et al., 1993). If the shorter SF_6_ lifetimes of 1,278 [1,120–1,475] years (Kovács et al., 2017) or 850 [580–1,400] years (Ray et al., 2017) would have been used instead of 3,200 years, our GWP(100) value of 26,700 would not have been significantly affected (by less than 5%), but a shorter lifetime could be important for metric calculations using time horizons of several hundred years.

### 3.2 Present‐Day Radiative Forcing From Halocarbons and Related Compounds

**Figure 6:**
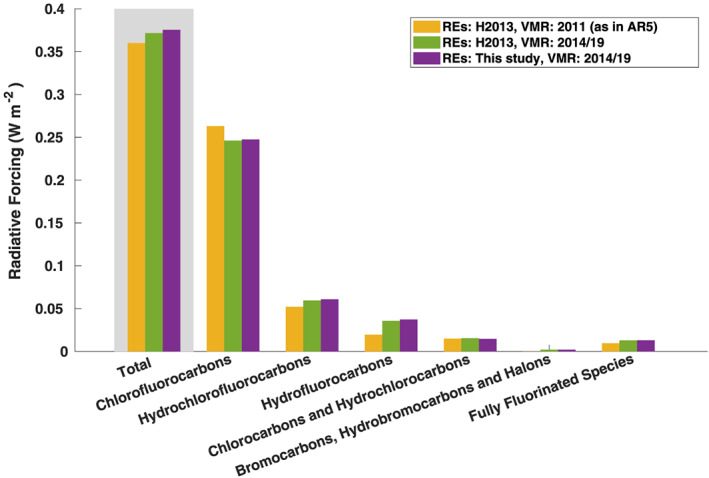
Preindustrial to present‐day radiative forcing for the different groups of compounds. Note that in AR5 (Myhre et al., 2013) (yellow bars), Halon‐1211 and Halon‐1301 were included in the CFC category.

Figure 6 shows preindustrial to present‐day radiative forcing for the halocarbons and related compounds discussed in section 3.1. RF for each group of compounds is compared against that reported in AR5 (Myhre et al., 2013—see their Table 8.2), when atmospheric concentrations from 2011 were used. We have used the atmospheric concentrations from Meinshausen et al. (2017) for 2014, but updated with 2019 observations from Butler and Montzka (2020) when available (see Table 4 for details). In the RF calculation, we use the preindustrial concentrations recommended by Meinshausen et al. (2017); these are nonzero for CH_3_Cl, CHCl_3_, CH_2_Cl_2_, CH_3_Br, and PFC‐14/CF_4_, and assumed to be zero for the remaining compounds (see Table 4 footnote).

**Table 4:** Concentrations (ppt) and Radiative Forcing (mW m^−2^) for the 40 Most Abundant Halocarbons and Related Compounds in the Atmosphere

		Radiative forcing (mW m^−2^)^a^		
Identifier/name	Concentrations (ppt)	H2013 REs	Updated REs	% difference
***Chlorofluorocarbons***		**246.16**	**247.47**	**1**
CFC‐11	226.50	58.89	58.76	0
CFC‐12	501.60	159.51	160.50	1
CFC‐113	69.70	21.05	21.01	0
CFC‐114	*16.31*	5.01	5.13	2
CFC‐115	*8.43*	1.70	2.08	22
***Hydrochlorofluorocarbons***		**59.44**	**60.95**	**3**
HCFC‐22	246.80	51.33	52.78	3
HCFC‐141b	24.39	3.95	3.92	−1
HCFC‐142b	22.00	4.16	4.25	2
***Hydrofluorocarbons***		**35.61**	**37.26**	**5**
HFC‐23	30.00	5.25	5.73	9
HFC‐32	*8.34*	0.92	0.93	1
HFC‐125	29.10	6.58	6.80	3
HFC‐134a	107.77	17.35	18.01	4
HFC‐143a	23.83	3.77	4.00	6
HFC‐152a	6.92	0.68	0.70	4
HFC‐227ea	*1.01*	0.26	0.28	6
HFC‐236fa	*0.13*	0.03	0.03	3
HFC‐245fa	*2.05*	0.50	0.50	1
HFC‐365mfc	*0.77*	0.17	0.18	2
HFC‐43‐10mee	*0.25*	0.11	0.09	−15
***Chlorocarbons and hydrochlorocarbons***		**15.50**	**14.67**	**−5**
1,1,1‐Trichloroethane	1.60	0.11	0.10	−6
Tetrachloromethane	78.50	13.35	13.04	−2
Chloromethane	*539.54*	0.83	0.38	−53
Dichloromethane	*36.35*	0.91	0.85	−7
Trichloromethane	*9.90*	0.30	0.29	−6
***Bromocarbons, hydrobromocarbons and halons***		**2.07**	**2.09**	**1**
Bromomethane	*6.69*	0.01	0.01	−14
Halon‐1211	3.25	0.96	0.98	2
Halon‐1301	3.28	0.98	0.98	0
Halon‐2402	0.40	0.13	0.12	0
***Fully fluorinated species***		**12.83**	**13.06**	**2**
Nitrogen trifluoride	*1.24*	0.25	0.25	0
Sulfur hexafluoride	9.96	5.65	5.64	0
Sulfuryl fluoride	*2.04*	0.41	0.43	5
PFC‐14	*81.09*	4.47	4.64	4
PFC‐116	*4.40*	1.10	1.15	4
PFC‐218	*0.60*	0.17	0.16	−3
PFC‐C‐318	*1.34*	0.42	0.42	0
PFC‐31‐10	*0.18*	0.07	0.07	2
PFC‐41‐12	*0.13*	0.05	0.05	1
PFC‐51‐14	*0.28*	0.12	0.13	2
PFC‐61‐16	*0.13*	0.07	0.07	0
PFC‐71‐18	*0.09*	0.05	0.05	1
**Total**		**371.60**	**375.49**	**1.0**

*Note*. Concentrations in italics are from 2014 (Meinshausen et al., 2017) and the remaining from 2019 (Butler & Montzka, 2020). The REs used to calculate RF are both from H2013 and from this study. Note that RE values with more significant digits than given in Table 3 have been used to calculate RF for each compound and that these are available in the supporting information.

^a^ Preindustrial values are zero except for chloromethane (457 ppt), dichloromethane (6.9 ppt), trichloromethane (6 ppt), bromomethane (5.3 ppt), and PFC‐14/CF_4_ (34.05 ppt), see Meinshausen et al. (2017).

When using the same RE values as in AR5 (from H2013), we see that the change from 2011 to 2014/2019 concentrations has led to a decrease in radiative forcing of CFCs (Figure 6). At the same time, concentrations of the CFC replacement compounds HCFCs and HFCs have increased and this leads to stronger RF for these compound groups, most notably for HFCs with a 83% increase in the RF. In total, RF due to increasing concentrations of HCFCs and HFCs more than outweighs the decrease in RF due to declining concentrations of CFCs. For the present‐day (2014/2019) RF, nearly all compound groups show slightly higher RF when using new REs compared to using AR5 REs. The total present‐day (2014/19) RF due to halocarbons is 0.38 [0.33 to 0.43] W m^−2^ compared to 0.36 [0.32 to 0.40] W m^−2^ in AR5, and while updated RE values push present‐day RF upward (by ~4 mW m^−2^; green vs. purple bars in Figure 6), the main reason for the RF increase can be attributed to increased concentrations (yellow vs. green bars in Figure 6).

Table 4 shows that the main contributors to the ~4 mW m^−2^ increase in RF are the updated RE values for CFC‐12, HCFC‐22, and HFC‐134a. Chloromethane has the largest relative change in RF (and RE) with a 53% decrease. While its atmospheric concentration is the highest among the compounds, its high abundance is mainly due to natural sources (WMO, 2019) and its influence on anthropogenic RF is therefore much smaller than would otherwise be expected. Here we have assumed a pre‐industrial value of 457 ppt from Meinshausen et al. (2017) who used a simple budget equation for its derivation, and it should be noted that this number is associated with uncertainties due to a lack of observations. Table 4 further shows that CFC‐115 and HFC‐43‐10mee, respectively, have the second and third largest relative RF change due to new REs. While the new REs of methyl chloride and HFC‐43‐10mee are lower compared to H2013, the RE of CFC‐115 is higher (see section 3.1).

The RF of 0.38 W m^−2^ due to halocarbons and other weak atmospheric absorbers can be put into context by comparison with the RF due to increased CO_2_ concentrations. When using the simplified formula from Etminan et al. (2016), and assuming preindustrial (1750) and 2019 CO_2_ concentrations of 278 ppm (Myhre et al., 2013) and 409.8 ppm (Butler & Montzka, 2020), respectively (and of 270 ppb and 331.9 ppb, respectively, for N_2_O), the present‐day RF due to CO_2_ is 2.09 W m^−2^. Thus, the RF due to halocarbons and other weak absorbers is 18% of the RF due to increased CO_2_ concentrations.

### 3.3 Updated Spectra, REs, and GWPs for Other Weak Atmospheric Absorbers

This section has a similar structure to section 3.1 but presents and discusses lifetimes, REs, and GWP(100) values for compounds other than the 40 most abundant halocarbons and related compounds. Table 5 shows results for the compound groups included in our previous review (H2013), and brief discussions of these results are given in sections 3.3.1, 3.3.7 below. Tables S7–S13 in the supporting information provide information on how the RE numbers were derived and list previously published absorption cross sections and reported RE values from the literature. In addition to the compound groups included in H2013, we have made RE calculations for a number of other compounds, mainly based on absorption spectra from the HITRAN 2016 (Kochanov et al., 2019) and PNNL (Sharpe et al., 2004) databases. These results are presented in Tables S14–S20 and a brief discussion of these results is given in section 3.3.8 below.

**Table 5:** Lifetimes (τ), Radiative Efficiencies and Direct GWPs (Relative to CO_2_) for Less Abundant Compounds

				RE (W m^−2^ ppb^−1^)		GWP(100)	
Identifier/name	Formula^a^	CASRN	*τ* (yr)	H2013	This work	H2013	This work
***Chlorofluorocarbons***							
**CFC‐13**	CClF_3_	75‐72‐9	640.0	0.26	**0.28**	13,900	**17,200**
**CFC‐112**	CCl_2_FCCl_2_F	76‐12‐0	63.6		**0.28**		**4,880**
**CFC‐112a**	CCl_3_CClF_2_	76‐11‐9	52.0		**0.25**		**3,740**
**CFC‐113a**	CCl_3_CF_3_	354‐58‐5	55.0		**0.24**		**4,140**
**CFC‐114a**	CCl_2_FCF_3_	374‐07‐2	105.0		**0.30**		**7,850**
**E‐R316c**	trans cyc (‐CClFCF_2_CF_2_CClF‐) ^b^	3832‐15‐3	75.0		**0.27**		**4,470**
**Z‐R316c**	cis cyc (‐CClFCF_2_CF_2_CClF‐) ^b^	3934‐26‐7	114.0		**0.30**		**5,990**
CFC 1112	CClF=CClF	598‐88‐9	*7.1 days*		***0.01***		**<1**
CFC 1112a	CCl_2_ = CF_2_	79‐35‐6	*2.3 days*		***0.01***		**<1**
**1,1,2‐trichloro‐2‐fluoroethene**	CCl_2_ = CClF	359‐29‐5			**(0.13)**		
**Chlorotrifluoroethylene**	CF_2_ = CClF	79‐38‐9			**(0.11)**		
***Hydrochlorofluorocarbons***							
**HCFC‐21**	CHCl_2_F	75‐43‐4	1.7	0.14	**0.15**	148	**168**
HCFC‐31	CH_2_ClF	593‐70‐4	1.2		***0.07***		**83**
**HCFC‐121**	CHCl_2_CCl_2_F	354‐14‐3	1.1		**0.15**		**61**
HCFC‐122	CHCl_2_CClF_2_	354‐21‐2	0.9	0.17	**0.16**	**59**	59
HCFC‐122a	CHClFCCl_2_F	354‐15‐4	3.1	0.21	**0.20**	258	**257**
**HCFC‐123**	CHCl_2_CF_3_	306‐83‐2	1.3	0.15	**0.16**	79	**95**
**HCFC‐123a**	CHClFCClF_2_	354‐23‐4	4.0	**0.23**	0.23	370	**415**
**HCFC‐124**	CHClFCF_3_	2837‐89‐0	5.9	0.20	**0.21**	527	**627**
**HCFC‐124a**	CHF_2_CClF_2_	354‐25‐6	17.0		**0.25**		**2,170**
**HCFC‐132**	CHClFCHClF	431‐06‐1	1.7		**0.14**		**128**
**HCFC‐132a**	CHCl_2_CHF_2_	471–43‐2	1.1		**0.13**		**74**
HCFC‐132c	CH_2_FCCl_2_F	1842‐05‐3	4.1	**0.17**	0.17	338	**359**
**HCFC‐133a**	CH_2_ClCF_3_	75–88‐7	4.6		**0.15**		**407**
**HCFC‐141**	CH_2_ClCHClF	430‐57‐9	1.1		**0.07**		**49**
HCFC‐225ca	CHCl_2_CF_2_CF_3_	422‐56‐0	1.9	**0.22**	0.22	127	**143**
HCFC‐225cb	CHClFCF_2_CClF_2_	507‐55‐1	5.9	**0.29**	0.29	525	**596**
**HCFO‐1233zd(E)**	(E)‐CF_3_CH=CHCl	102687‐65‐0	42.5 days	0.04	**0.07**	1	**4**
**HCFO‐1233zd(Z)**	(Z)‐CF_3_CH=CHCl	99728‐16‐2	13.0 days		**0.02**		**<1**
(E/Z)‐1‐chloro‐2‐fluoro‐ethene	(E/Z)‐CHCl = CHF	460‐16‐2	*1.8 days*		***0.001***		**<1**
***Hydrofluorocarbons***							
**HFC‐41**	CH_3_F	593‐53‐3	2.8	**0.02**	0.02	116	**142**
**HFC‐134**	CHF_2_CHF_2_	359‐35‐3	10.0	**0.19**	0.19	1,120	**1,330**
HFC‐143	CH_2_FCHF_2_	430‐66‐0	3.6	**0.13**	0.13	328	**382**
HFC‐152	CH_2_FCH_2_F	624‐72‐6	0.5	0.04	**0.05**	16	**23**
HFC‐161	CH_3_CH_2_F	353‐36‐6	80.0 days	**0.02**	0.02	4	**5**
HFC‐227ca	CF_3_CF_2_CHF_2_	2252‐84‐8	30.0	0.27	**0.26**	2,640	**3,140**
HFC‐236cb	CH_2_FCF_2_CF_3_	677‐56‐5	13.4	**0.23**	0.23	1,210	**1,420**
HFC‐236ea	CHF_2_CHFCF_3_	431‐63‐0	11.4	**0.30**	*0.30*	1,340	**1,570**
HFC‐245ca	CH_2_FCF_2_CHF_2_	679‐86‐7	6.6	**0.24**	*0.24*	716	**827**
HFC‐245cb	CF_3_CF_2_CH_3_	1814‐88‐6	39.9	0.24	**0.25**	4,620	**4,790**
HFC‐245ea	CHF_2_CHFCHF_2_	24270‐66‐4	3.2	**0.16**	*0.16*	235	**267**
HFC‐245eb	CH_2_FCHFCF_3_	431‐31‐2	3.2	**0.20**	*0.20*	290	**341**
HFC‐263fb	CH_3_CH_2_CF_3_	421‐07‐8	1.1	**0.10**	*0.10*	76	**78**
HFC‐272ca	CH_3_CF_2_CH_3_	420‐45–1	9.0	0.07	**0.08**	144	**629**
HFC‐329p	CHF_2_CF_2_CF_2_CF_3_	375‐17‐7	32.0	**0.31**	0.31	2,360	**3,040**
HFO‐1123	CHF=CF_2_	359‐11‐5	1.4 days		***0.002***		**<1**
HFO‐1132a	CH_2_ = CF_2_	75‐38‐7	4.6 days	**0.004**	0.004	**<1**	<1
**HFO‐1141**	CH_2_ = CHF	75‐02‐5	2.5 days	**0.002**	0.002	**<1**	<1
HFO‐1225ye(Z)	(Z)‐CF_3_CF=CHF	5528‐43‐8	10.0 days	**0.02**	0.02	**<1**	<1
HFO‐1225ye(E)	(E)‐CF_3_CF=CHF	5595‐10‐8	5.7 days	0.01	**0.02**	**<1**	<1
HFO‐1234ze(Z)	(Z)‐CF_3_CH=CHF	29118‐25‐0	10.0 days	**0.02**	0.02	**<1**	<1
HFO‐1234ze(E)	(E)‐CF_3_CH=CHF	29188‐24‐9	19.0 days	0.04	**0.05**	**1**	1
HFO‐1234yf	CF_3_CF=CH_2_	754‐12‐1	12.0 days	0.02	**0.03**	**<1**	<1
HFO‐1336mzz(E)	(E)‐CF_3_CH=CHCF_3_	N/A	0.3		***0.13***		**19**
HFO‐1336mzz(Z)	(Z)‐CF_3_CH=CHCF_3_	692‐49‐9	27.0 days	**0.07**	*0.07*	**2**	2
HFO‐1243zf	CF_3_CH=CH_2_	677‐21‐4	9.0 days	0.01	**0.02**	**<1**	<1
HFC‐1345zfc	CF_3_CF_2_CH=CH_2_	374‐27‐6	9.0 days	0.01	**0.02**	**<1**	<1
3,3,4,4,5,5,6,6,6‐Nonafluorohex‐1‐ene	n‐C_4_F_9_CH=CH_2_	19430‐93‐4	9.0 days	**0.03**	0.03	**<1**	<1
3,3,4,4,5,5,6,6,7,7,8,8,8‐Tridecafluorooct‐1‐ene	n‐C_6_F_13_CH=CH_2_	25291‐17‐2	9.0 days	**0.03**	0.03	**<1**	<1
3,3,4,4,5,5,6,6,7,7,8,8,9,9,10,10,10‐Heptadecafluorodec‐1‐ene	n‐C_8_F_17_CH=CH_2_	21652‐58‐4	9.0 days	0.03	**0.04**	**<1**	<1
**1‐Propene, 3,3,3‐trifluoro‐2‐(trifluoromethyl)‐**	(CF_3_)_2_C=CH_2_	382‐10‐5	*10.3 days*		**0.03**		**<1**
1,1,2,2,3,3‐hexafluorocyclopentane	cyc (‐CF2CF2CF2CH2CH2‐)	123768‐18‐3	*1.6*		***0.20***		**126**
1,1,2,2,3,3,4‐heptafluorocyclopentane	cyc (‐CF_2_CF_2_CF_2_CHFCH_2_‐)	15290‐77‐4	*2.8*		***0.24***		**243**
1,3,3,4,4,5,5‐heptafluorocyclopentene	cyc (‐CF_2_CF_2_CF_2_CF=CH‐)	1892‐03‐1	*0.6*		***0.21***		**47**
(4R,5R)‐1,1,2,2,3,3,4,5‐octafluorocyclopentane	trans‐cyc (‐CF_2_CF_2_CF_2_CHFCHF‐) ^b^	158,389‐18‐5	*3.2*		***0.26***		**271**
HFO‐1438ezy(E)	(E)‐(CF_3_)_2_CFCH=CHF	14149‐41‐8	0.3		***0.08***		**9**
HFO‐1447fz	CF_3_(CF_2_)_2_CH=CH_2_	355‐08‐8	9.0 days		***0.03***		**<1**
1,3,3,4,4‐pentafluorocyclobutene	cyc (‐CH=CFCF_2_CF_2_‐)	374‐31‐2	0.7		***0.27***		**97**
3,3,4,4‐tetrafluorocyclobutene	cyc (‐CH=CHCF_2_CF_2_‐)	2714‐38‐7	84.0 days		***0.21***		**27**
**3‐Fluoro‐1‐propene**	CH_2_ = CHCH_2_F	818‐92‐8			**(0.06)**		
**1‐Fluorohexane**	n‐C_6_H_13_F	373‐14‐8			**(0.04)**		
**Fluorobenzene**	C_6_H_5_‐F	462‐06‐6			**(0.07)**		
***Chlorocarbons and hydrochlorocarbons***							
**Chloroethane**	CH_3_CH_2_Cl	75‐00‐3	48.0 days		**0.004**		**<1**
**1,1‐Dichloroethane**	CH_3_CHCl_2_	75‐34‐3			**(0.03)**		
**1,2‐Dichloroethane**	CH_2_ClCH_2_Cl	107‐06‐2	82.0 days	**0.01**	0.01	**1**	1
**1,1,2‐Trichloroethane**	CH_2_ClCHCl_2_	79‐00‐5			**(0.05)**		
**1,1,1,2‐Tetrachloroethane**	CH_2_ClCCl_3_	630‐20‐6			**(0.10)**		
**1,1,2,2‐Tetrachloroethane**	CHCl_2_CHCl_2_	79‐34‐5			**(0.10)**		
**1,1,2‐Trichloroethene**	CHCl = CCl_2_	79‐01‐6	5.6 days		**0.01**		**<1**
**1,1,2,2‐Tetrachloroethene**	CCl_2_ = CCl_2_	127‐18‐4	0.3		**0.05**		**7**
**2‐Chloropropane**	CH_3_CHClCH_3_	75‐29‐6	22.0 days		**0.004**		**<1**
**Chloromethyl benzene**	C_6_H_5_‐CH_2_Cl	100‐44‐7			**(0.02)**		
**3‐Chloro‐1‐propene**	CH_2_ = CHCH_2_Cl	107‐5‐1			**(0.05)**		
**1‐Chloro‐4‐methylbenzene**	p‐Cl‐C_6_H_4_‐CH_3_	106‐43‐4			**(0.05)**		
**3,4‐Dichloro‐1‐butene**	CH_2_ClCHClCH=CH_2_	760‐23‐6			**(0.06)**		
**1‐Chloro‐3‐methylbenzene**	m‐Cl‐C_6_H_4_‐CH_3_	108‐41‐8			**(0.05)**		
**2,3‐Dichloropropene**	CH_2_ClCCl = CH_2_	78‐88‐6			**(0.05)**		
**1‐Chloro‐2‐methylbenzene**	o‐Cl‐C_6_H_4_‐CH_3_	95‐49‐8			**(0.04)**		
**1,2‐Dichloropropene**	CHCl = CClCH_3_	563‐54‐2			**(0.03)**		
**1‐Chloropentane**	CH_3_(CH_2_)_3_CH_2_Cl	543‐59‐9			**(0.02)**		
**1‐Chlorobutane**	CH_3_(CH_2_)_2_CH_2_Cl	109‐69‐3	*4.5 days*		**0.001**		**<1**
**1‐Chloro‐2‐methylpropane**	(CH_3_)_2_CHCH_2_Cl	513‐36‐0			**(0.02)**		
**Chloroethene**	CH_2_ = CHCl	75‐01‐4			**(0.04)**		
**1,2‐Dichloroethene (E)**	(E)‐CHCl = CHCl	156‐60‐5			**(0.09)**		
**Hexachloro‐1,3‐butadiene**	CCl_2_ = CClCCl = CCl_2_	87‐68‐3			**(0.14)**		
**1,3‐Dichloropropene (E)**	(E)‐CHCl = CHCH_2_Cl	10061‐02‐6			**(0.06)**		
**1,3‐Dichloropropene (Z)**	(Z)‐CHCl = CHCH_2_Cl	10061‐01‐5			**(0.06)**		
**1,3‐Dichloropropane**	CH_2_ClCH_2_CH_2_Cl	142‐28‐9			**(0.03)**		
**Chlorobenzene**	C_6_H_5_‐Cl	108‐90‐7			**(0.04)**		
**1,4‐Dichlorobenzene**	p‐Cl‐C_6_H_4_‐Cl	106‐46‐7			**(0.08)**		
**1,3‐Dichlorobenzene**	m‐Cl‐C_6_H_4_‐Cl	541‐73‐1			**(0.08)**		
**1,2‐Dichlorobenzene**	o‐Cl‐C_6_H_4_‐Cl	95‐50‐1			**(0.05)**		
**1,2‐Dichloroethylene (Z)**	(Z)‐CHCl = CHCl	156‐59‐2			**(0.04)**		
**Hexachloro‐1,3‐cyclopentadiene**	C_5_Cl_6_	77‐47‐4			**(0.11)**		
**3‐Chloro‐1‐propyne**	CH_2_ClC ≡ CH	624‐65‐7			**(0.02)**		
***Bromocarbons, hydrobromocarbons, and halons***							
**Dibromomethane**	CH_2_Br_2_	74‐95‐3	0.4	**0.01**	0.01	1	**2**
Halon‐1201	CHBrF_2_	1511‐62‐2	4.9	**0.15**	0.15	376	**398**
**Halon‐1202**	CBr_2_F_2_	75‐61‐6	2.5	**0.27**	0.27	231	**226**
Halon‐2301	CH_2_BrCF_3_	421‐06‐7	3.2	**0.14**	0.14	173	**186**
Halon‐2311 (Halothane)	CHBrClCF_3_	151‐67‐7	1.0	**0.13**	0.13	41	**47**
Halon‐2401	CHBrFCF_3_	124‐72‐1	2.9	**0.19**	0.19	184	**211**
**Tribromomethane**	CHBr_3_	75‐25‐2	57.0 days		**0.01**		**<1**
**Halon‐1011**	CH_2_BrCl	74‐97‐5	0.5		**0.02**		**5**
**Bromoethane**	CH_3_CH_2_Br	74‐96‐4	50.0 days		**0.01**		**<1**
**1,2‐Dibromoethane**	CH_2_BrCH_2_Br	106‐93‐4	89.0 days		**0.01**		**1**
**1‐Bromopropane**	CH_3_CH_2_CH_2_Br	106‐94‐5	15.0 days		**0.002**		**<1**
**2‐Bromopropane**	CH_3_CHBrCH_3_	75‐26‐3	20.0 days		**0.004**		**<1**
**Bromomethyl benzene**	C_6_H_5_‐CH_2_Br	100‐39‐0			**(0.03)**		
**3‐Bromo‐1‐propene**	CH_2_ = CHCH_2_Br	106‐95–6			**(0.04)**		
**Bromine Nitrate**	BrONO_2_	40423‐14‐1			**(0.10)**		
**Bromoethene**	CH_2_ = CHBr	593‐60‐2			**(0.04)**		
***Fully fluorinated species***							
Pentadecafluorotriethylamine	N(C_2_F_5_)_3_	359‐70‐6	>1000.0		***0.61***		**10,900**
Perfluorotripropylamine, PTPA	N (CF_2_CF_2_CF_3_)_3_	338‐83‐0	>1000.0		***0.75***		**9,580**
**Heptacosafluorotributylamine, PFTBA**	N (CF_2_CF_2_CF_2_CF_3_)_3_	311‐89‐7	>1000.0		**0.91**		**9,000**
Perfluorotripentylamine	N (CF_2_CF_2_CF_2_CF_2_CF_3_)_3_	338‐84‐1	>1000.0		***0.95***		**7,700**
Heptafluoroisobutyronitrile	(CF_3_)_2_CFCN	42532‐60‐5	*34.5*		***0.25***		**2,900**
**(Trifluoromethyl)sulfur pentafluoride**	SF_5_CF_3_	373‐80‐8	800.0	0.59	**0.58**	17,400	**19,600**
Hexafluorocyclobutene	cyc (‐CF=CFCF_2_CF_2_‐)	697‐11‐0	*1.0*		***0.30***		**132**
**Pentafluoro‐2‐(trifluoromethyl)‐1‐propene, PFIB**	(CF_3_)_2_C=CF_2_	382‐21‐8			**(0.34)**		
Octafluorocyclopentene	cyc (‐CF_2_CF_2_CFCF_2_CF_2_‐)	559‐40‐0	1.1	0.08	***0.25***	2	**82**
**Hexafluorobenzene**	C_6_F_6_	392‐56‐3			**(0.15)**		
Perfluorodecalin (mixed), PFC‐91‐18	C_10_F_18_ ^b^	306‐94‐5	2000.0	0.55	**0.54**	7,190	**7,940**
**Perfluorodecalin (cis)**	Z‐C_10_F_18_ ^b^	60433‐11‐6	2000.0	**0.56**	0.56	7,240	**8,270**
**Perfluorodecalin (trans)**	E‐C_10_F_18_ ^b^	60433‐12‐7	2000.0	0.48	**0.51**	6,290	**7,560**
PFC‐1114	CF_2_ = CF_2_	116‐14‐3	1.2 days	**0.002**	0.002	**<1**	<1
**PFC‐1216**	CF_3_CF=CF_2_	116‐15‐4	5.5 days	**0.01**	0.01	**<1**	<1
Hexafluorobuta‐1,3‐diene	CF_2_ = CFCF=CF_2_	685‐63‐2	1.1 days	**0.003**	0.003	**<1**	<1
Octafluoro‐1‐butene	CF_3_CF_2_CF=CF_2_	357‐26‐6	6.0 days	**0.02**	0.02	**<1**	<1
Octafluoro‐2‐buene	CF_3_CF=CFCF_3_	360‐89‐4	31.0 days	**0.07**	0.07	**2**	2
***Halogenated alcohols and ethers***							
HFE‐125	CHF_2_OCF_3_	3822‐68‐2	135.0	0.41	**0.42**	12,400	**15,100**
HFE‐134	CHF_2_OCHF_2_	1691‐17‐4	26.9	**0.45**	0.45	5,560	**6,980**
**HFE‐143a**	CH_3_OCF_3_	421‐14‐7	4.9	0.18	**0.19**	523	**647**
HFE‐227ea	CF_3_CHFOCF_3_	2356‐62‐9	54.8	0.44	**0.46**	6,450	**7,930**
HCFE‐235ca2 (enflurane)	CHF_2_OCF_2_CHFCl	13838‐16‐9	4.4	**0.41**	0.41	583	**686**
HCFE‐235da2 (isoflurane)	CHF_2_OCHClCF_3_	26675‐46‐7	3.5	0.42	**0.43**	491	**565**
HFE‐236ea2 (desflurane)	CHF_2_OCHFCF_3_	57041‐67‐5	14.1	0.45	**0.46**	1,790	**2,720**
HFE‐236fa	CF_3_CH_2_OCF_3_	20193‐67‐3	7.5	0.36	**0.37**	979	**1,160**
HFE‐245cb2	CF_3_CF_2_OCH_3_	22410‐44‐2	5.0	0.33	**0.34**	654	**784**
HFE‐245fa1	CHF_2_CH_2_OCF_3_	84011‐15‐4	6.7	**0.31**	0.31	828	**980**
**HFE‐245fa2**	CHF_2_OCH_2_CF_3_	1885‐48‐9	5.5	**0.36**	0.36	812	**922**
**2,2,3,3,3‐Pentafluoropropan‐1‐ol**	CF_3_CF_2_CH_2_OH	422‐05‐9	0.5	0.14	**0.16**	19	**36**
HFE‐254cb1	CH_3_OCF_2_CHF_2_	425‐88‐7	2.5	**0.26**	0.26	301	**344**
HFE‐263mf	CF_3_CH_2_OCH_3_	460‐43‐5	28.0 days	0.04	**0.05**	1	**2**
HFE‐263 m1	CF_3_OCH_2_CH_3_	690‐22‐2	0.4	**0.13**	0.13	29	**31**
3,3,3‐Trifluoropropan‐1‐ol	CF_3_CH_2_CH_2_OH	2240‐88‐2	15.0 days	0.02	**0.03**	<1	**<1**
HFE‐329mcc2	CHF_2_CF_2_OCF_2_CF_3_	134769‐21‐4	25.0	0.53	**0.55**	3,070	**3,970**
HFE‐338mmz1	(CF_3_)_2_CHOCHF_2_	26103‐08‐2	22.3	0.44	**0.45**	2,620	**3,200**
HFE‐338mcf2	CF_3_CH_2_OCF_2_CF_3_	156053‐88‐2	7.5	0.44	**0.45**	929	**1,090**
Sevoflurane (HFE‐347mmz1)	(CF_3_)_2_CHOCH_2_F	28523‐86‐6	1.9	0.32	**0.31**	216	**205**
HFE‐347mcc3 (HFE‐7000)	CH_3_OCF_2_CF_2_CF_3_	375‐03‐1	5.1	**0.34**	0.34	530	**605**
HFE‐347mcf2	CHF_2_CH_2_OCF_2_CF_3_	171182‐95‐9	6.7	0.42	**0.43**	854	**1,010**
HFE‐347pcf2	CHF_2_CF_2_OCH_2_CF_3_	406‐78‐0	6.1	**0.48**	*0.48*	889	**1,030**
HFE‐347mmy1	(CF_3_)_2_CFOCH_3_	22052‐84‐2	3.7	**0.32**	0.32	363	**412**
HFE‐356mec3	CH_3_OCF_2_CHFCF_3_	382‐34‐3	2.5	0.30	**0.29**	387	**277**
HFE‐356mff2	CF_3_CH_2_OCH_2_CF_3_	333‐36‐8	0.4	0.17	**0.19**	17	**26**
HFE‐356pcf2	CHF_2_CH_2_OCF_2_CHF_2_	50807–77‐7	6.0	0.37	**0.38**	719	**872**
HFE‐356pcf3	CHF_2_OCH_2_CF_2_CHF_2_	35042–99‐0	3.5	**0.38**	0.38	446	**508**
HFE‐356pcc3	CH_3_OCF_2_CF_2_CHF_2_	160620–20‐2	2.5	0.32	**0.30**	413	**291**
HFE‐356mmz1	(CF_3_)_2_CHOCH_3_	13171–18‐1	65.0 days	0.15	**0.12**	14	**9**
HFE‐365mcf3	CF_3_CF_2_CH_2_OCH_3_	378–16‐5	25.0 days	0.05	**0.06**	1	**2**
HFE‐374pc2	CHF_2_CF_2_OCH_2_CH_3_	512–51‐6	76.0 days	0.30	**0.13**	627	**13**
4,4,4‐Trifluorobutan‐1‐ol	CF_3_(CH_2_)_2_CH_2_OH	461–18‐7	5.4 days	**0.01**	0.01	**<1**	<1
1,1,1,3,3,3‐Hexafluoro‐2‐(trifluoromethyl)‐2‐propanol	(CF_3_)_3_COH	2378–02‐01		**(0.38)**	(0.38)		
2,2,3,3,4,4,5,5‐Octafluorocyclopentanol	cyc (−(CF_2_)_4_CH(OH)‐)	16621–87‐7	0.3	**0.16**	0.16	13	**14**
HFE‐43‐10pccc124 (H‐Galden 1,040x, HG‐11)	CHF_2_OCF_2_OCF_2_CF_2_OCHF_2_	188690–77‐9	14.1	1.02	**1.03**	2,820	**3,380**
HFE‐449 s1 (HFE‐7100)	n/i‐C_4_F_9_OCH_3_	219484–64‐7	4.8	**0.36**	0.36	421	**483**
n‐HFE‐7100	CF_3_CF_2_CF_2_CF_2_OCH_3_	163702–07‐6	4.8	**0.42**	0.42	486	**571**
i‐HFE‐7100	(CF_3_)_2_CFCF_2_OCH_3_	163702–08‐7	4.8	0.35	**0.34**	407	**458**
HFE‐569sf2 (HFE‐7200)	C_4_F_9_OC_2_H_5_	N/A	0.8	**0.30**	0.30	57	**64**
i‐HFE‐7200	(CF_3_)_2_CFCF_2_OCH_2_CH_3_	163702‐06‐5	0.6	0.24	**0.22**	44	**36**
HFE‐7300	(CF_3_)_2_CFCF(OC_2_H_5_)CF_2_CF_2_CF_3_	132182‐92‐4	*5.2*		***0.48***		**425**
HFE‐7500	n‐C_3_F_7_CF(OC_2_H_5_)CF (CF_3_)_2_	297730‐93‐9	*0.3*		***0.27***		**14**
HFE‐236ca12 (HG‐10)	CHF_2_OCF_2_OCHF_2_	78522‐47‐1	26.5	**0.65**	0.65	5,350	**6,370**
HFE‐338pcc13 (HG‐01)	CHF_2_OCF_2_CF_2_OCHF_2_	188690‐78‐0	13.4	0.86	**0.87**	2,910	**3,480**
**1,1,1,3,3,3‐Hexafluoropropan‐2‐ol**	(CF_3_)_2_CHOH	920‐66‐1	1.9	0.26	**0.27**	182	**216**
HG‐02	CHF_2_(OCF_2_CF_2_)_2_OCHF_2_	205367‐61‐9	26.9	**1.15**	*1.15*	5,140	**6,030**
HG‐03	CHF_2_(OCF_2_CF_2_)_3_OCHF_2_	173350‐37‐3	26.9	**1.43**	*1.43*	4,800	**5,630**
(2,2,2‐Trifluoroethoxy)ethene	CF_3_CH_2_OCH=CH_2_	406‐90‐6	3.6 days	**0.01**	*0.01*	**<1**	<1
2‐Ethoxy‐3,3,4,4,5‐pentafluorotetrahydro‐2,5‐bis[1,2,2,2‐tetrafluoro‐1‐(trifluoromethyl)ethyl]‐furan	C_12_H_5_F_19_O_2_ ^b^	920979‐28‐8	0.8	**0.49**	*0.49*	56	**51**
**Difluoro (methoxy)methane**	CH_3_OCHF_2_	359‐15‐9	1.1	0.17	**0.15**	144	**143**
HG'‐01	CH_3_OCF_2_CF_2_OCH_3_	73287–23‐7	1.7	**0.29**	0.29	222	**212**
HG'‐02	CH_3_O(CF_2_CF_2_O)_2_CH_3_	485399–46‐0	1.7	**0.56**	0.56	250	**240**
HG'‐03	CH_3_O(CF_2_CF_2_O)_3_CH_3_	485399–48‐2	1.7	0.77	**0.76**	239	**230**
HFE‐329me3	CF_3_CFHCF_2_OCF_3_	428454–68‐6	33.6	0.48	**0.49**	4,550	**4,620**
HFE‐338mec3	CF_3_CFHCF_2_OCF_2_H	56860–85–6		**(0.52)**	(0.52)		
3,3,4,4,5,5,6,6,7,7,7‐Undecafluoroheptan‐1‐ol	CF_3_(CF_2_)_4_CH_2_CH_2_OH	185689–57‐0	17.0 days	0.06	**0.05**	**1**	<1
3,3,4,4,5,5,6,6,7,7,8,8,9,9,9‐Pentadecafluorononan‐1‐ol	CF_3_(CF_2_)_6_CH_2_CH_2_OH	755–02‐2	17.0 days	0.07	**0.06**	1	**<1**
3,3,4,4,5,5,6,6,7,7,8,8,9,9,10,10,11,11,11‐Nonadecafluoroundecan‐1‐ol	CF_3_(CF_2_)_8_CH_2_CH_2_OH	87017–97‐8	17.0 days	**0.05**	0.05	**<1**	<1
2‐Chloro‐1,1,2‐trifluoro‐1‐methoxyethane	CH_3_OCF_2_CHClF	425–87‐6	1.4	**0.21**	0.21	122	**142**
PFPMIE (perfluoropolymethylisopropyl)	CF_3_OCF(CF_3_)CF_2_OCF_2_OCF_3_	1309353–34‐1	800.0	0.65	**0.64**	9,710	**10,900**
HFE‐216	CF_3_OCF=CF_2_	1187‐93‐5	1.6 days	0.03	**0.01**	**<1**	<1
Perfluoroethyl formate	CF_3_CF_2_OCHO	313064‐40‐3	3.6	0.44	***0.41***	580	**626**
2,2,2‐Trifluoroethyl formate	CF_3_CH_2_OCHO	32042‐38‐9	0.5	0.16	***0.19***	33	**57**
1,1,1,3,3,3‐Hexafluoropropan‐2‐yl formate	(CF_3_)_2_CHOCHO	856766‐70‐6	3.1	0.33	***0.26***	334	**282**
**Vinyl 2,2,2‐trifluoroacetate**	CF_3_C(O)OCH=CH_2_	433‐28‐3	*1.4 days*	0.387	**0.004**		**<1**
**Ethyl 2,2,2‐trifluoroacetate**	CF_3_C(O)OCH_2_CH_3_	383‐63‐1	22.0 days	0.05	**0.06**	1	**2**
**Allyl 2,2,2‐trifluoroacetate**	CF_3_C(O)OCH_2_CH=CH_2_	383‐67‐5	*1.3 days*	0.354	**0.005**		**<1**
Methyl 2,2,2‐trifluoroacetate	CF_3_C(O)OCH_3_	431‐47‐0	1.0	0.18	***0.16***	52	**86**
2,2,3,3,4,4,4‐Heptafluorobutan‐1‐ol	CF_3_CF_2_CF_2_CH_2_OH	375‐01‐9	0.6	**0.20**	0.20	33	**38**
1,1,2‐Trifluoro‐2‐(trifluoromethoxy)ethane	CHF_2_CHFOCF_3_	84011‐06‐3	9.0	0.34	**0.35**	1,240	**1,320**
1‐Ethoxy‐1,1,2,3,3,3‐hexafluoropropane	CF_3_CHFCF_2_OCH_2_CH_3_	380‐34‐7	0.4	**0.19**	0.19	23	**28**
1,1,1,2,2,3,3‐Heptafluoro‐3‐(1,2,2,2‐tetrafluoroethoxy)propane	CF_3_CF_2_CF_2_OCHFCF_3_	3330‐15‐2	59.4	0.58	**0.59**	6,490	**7,000**
2,2,3,3‐Tetrafluoropropan‐1‐ol	CHF_2_CF_2_CH_2_OH	76‐37‐9	93.0 days	**0.11**	0.11	13	**15**
2,2,3,4,4,4‐Hexafluorobutan‐1‐ol	CF_3_CHFCF_2_CH_2_OH	382‐31‐0	0.4	0.19	**0.23**	17	**32**
1,1,2,2‐Tetrafluoro‐3‐methoxypropane	CHF_2_CF_2_CH_2_OCH_3_	60598‐17‐6	26.0 days	0.03	**0.05**	1	**2**
perfluoro‐2‐methylpentan‐3‐one	CF_3_CF_2_C(O)CF (CF_3_)_2_	756‐13‐8	7.0 days	**0.03**	0.03	**<1**	<1
3,3,3‐Trifluoropropanal	CF_3_CH_2_CHO	460‐40‐2	3.0 days	0.004	**0.005**	**<1**	<1
4,4,4‐Trifluorobutanal	CF_3_CH_2_CH_2_CHO	406‐87‐1		**(0.16)**	(0.16)		
**2‐Fluoroethanol**	CH_2_FCH_2_OH	371‐62‐0	16.0 days	0.02	**0.01**	**1**	<1
2,2‐Difluoroethanol	CHF_2_CH_2_OH	359‐13‐7	61.0 days	0.04	**0.05**	3	**6**
**2,2,2‐Trifluoroethanol**	CF_3_CH_2_OH	75‐89‐8	0.5	0.10	**0.12**	20	**37**
HG‐04	CHF_2_O(CF_2_CF_2_O)_4_CHF_2_	173350‐38‐4	26.9	**1.46**	*1.46*	3,930	**4,610**
Methyl‐perfluoroheptene‐ethers	CH_3_OC_7_F_13_		*0.3*		***0.27***		**16**
**1,1,1‐Trifluoropropan‐2‐one**	CF_3_C(O)CH_3_	421‐50‐1	*5.1 days*		**0.01**		**<1**
1,1,1‐Trifluorobutan‐2‐one	CF_3_C(O)CH_2_CH_3_	381‐88‐4	*6.6 days*		***0.01***		**<1**
**2,2,2‐Trifluoroethanal**	CF_3_CHO	75‐90‐1			**(0.17)**		
**2,2,3,3,3‐Pentafluoropropanal**	CF_3_CF_2_CHO	422‐06‐0			**(0.20)**		
**2,2,3,3,4,4,4‐Heptafluorobutanal**	CF_3_CF_2_CF_2_CHO	375‐02‐0			**(0.25)**		
**2,2,3,3,4,4,5,5,5‐Nonafluoropetanal**	CF_3_CF_2_CF_2_CF_2_CHO	375‐53‐1			**(0.29)**		
**Acryloyl chloride**	CH_2_ = CHC(O)Cl	814‐68‐6			**(0.15)**		
**Acetyl chloride**	CH_3_COCl	75‐36‐5			**(0.11)**		
**1‐chloro‐2‐ethoxyethane**	C_4_H_9_ClO	628‐34‐2			**(0.10)**		
**2‐Chloroethanol**	CH_2_ClCH_2_OH	107‐07‐3			**(0.06)**		
**2‐(Chloromethyl)oxirane**	C_3_H_5_ClO ^b^	106‐89‐8			**(0.05)**		
**1‐Chloropropan‐2‐one**	CH_3_C(O)CH_2_Cl	78‐95‐5			**(0.04)**		
**1‐chloro‐2‐(2‐chloroethoxy)ethane**	CH_2_ClCH_2_OCH_2_CH_2_Cl	111‐44‐4			**(0.11)**		
2‐chloroethyl vinyl ether	ClCH_2_CH_2_OCH=CH_2_	110‐75‐8	*0.1 days*		***0.001***		**<1**
**(Chlorometoxy)ethane**	CH_3_CH_2_OCH_2_Cl	3188‐13‐4			**(0.11)**		
**Chloro (methoxy)methane**	CH_3_OCH_2_Cl	107‐30‐2			**(0.09)**		
**Ethyl carbonochloridate**	CH_3_CH_2_OC(O)Cl	541‐41‐3			**(0.26)**		
**1‐Fluoropropan‐2‐one**	CH_3_C(O)CH_2_F	430‐51‐3			**(0.05)**		
**1,1,1,3,3,3‐hexafluoropropan‐2‐one**	CF_3_C(O)CF_3_	684‐16‐2			**(0.29)**		
**Trifluoroacetic acid**	CF_3_C(O)OH	76‐05‐1			**(0.36)**		
**Trifluoroacetic anhydride**	CF_3_C(O)OC(O)CF_3_	407‐25‐0			**(0.51)**		
**Methacryloyl chloride**	CH_2_ = C (CH_3_)C(O)Cl	920‐46‐7			**(0.12)**		

*Note*. Compounds where the radiative efficiencies are based on new spectra since the H2013 review are marked in bold. Recommended RE and GWP(100) values are indicated in bold. Lifetimes are from WMO (2019) except those in italics (see text and Tables S7–S13 for details). RE values in parentheses are based on a constant horizontal and vertical distribution because of missing information about the lifetime of the compound. RE and GWP values in italic are based on previous publications (see text and Tables S7–S13 for details). Note that RE values with more significant digits have been used to calculate GWP(100) and that these are available in the supporting information.

^a^ cyc, cyclic compound.

^b^ Structure displayed in Table S21.

#### 3.3.1 Chlorofluorocarbons

The CFC‐13 spectrum from the PNNL database was added and led to a higher RE (0.28 W m^−2^ ppb^−1^) compared to H2013 (0.26 W m^−2^ ppb^−1^) (Tables 5 and S7). Since H2013, several CFC compounds have been added. CFC‐112, CFC‐112a, and CFC‐113a were detected in the atmosphere recently (Laube et al., 2014) and the atmospheric impact of these compounds have been quantified (Davis et al., 2016; Etminan et al., 2014). We used the spectra from Etminan et al. (2014), Davis et al. (2016), and PNNL and confirmed the high RE and GWP(100) values for these compounds. Calculations have further been made for three additional potent greenhouse gases (CFC‐114a, E‐R316c, and Z‐R316c) using spectra from Davis et al. (2016) and Papadimitriou et al. (2013).

#### 3.3.2 Hydrochlorofluorocarbons

Absorption spectra from the PNNL database were added for four HCFCs in H2013 (HCFC‐21, HCFC‐123, HCFC‐123a, and HCFC‐124); the new RE values are in good agreement (difference of 5% or less) with the H2013 values (Tables 5 and S8). The spectrum from Gierczak et al. (2014) has been added for HCFO‐1233zd(E), but the increased RE value since H2013 is mainly due to the longer lifetime and is therefore less influenced by the lifetime‐correction factor.

HCFC‐133a has been identified in the atmosphere recently (Laube et al., 2014). RE and GWP(100) values are given for this compound, based on absorption spectra from three sources (Etminan et al., 2014; McGillen et al., 2015; Sharpe et al., 2004). Calculations have been made for six additional HCFCs since H2013 and most notable is the relatively long‐lived HCFC‐124a (lifetime 17 years) with RE and GWP(100) values of 0.25 W m^−2^ ppb^−1^ and 2,170, respectively. In addition to the compounds listed here, Papanastasiou et al. (2018) present GWP(100) values for a large range of HCFCs using theoretically determined absorption spectra.

#### 3.3.3 Hydrofluorocarbons

RE calculations for three HFCs in H2013 (HFC‐41, HFC‐134, and HFO‐1141) are now based on additional absorption spectra from PNNL. The RE value for one of these (HFO‐1141) in H2013 was only based on a reported RE in the literature (Tables 5 and S9) rather than our calculations. For seven additional compounds, RE values in H2013 were based on reported RE in the literature and not our own calculations. These RE values have been retained here but the GWP(100) values are updated to include the effect of changes in lifetime and the AGWP of CO_2_. For most of the HFCs, changes in RE values since H2013 are minor and mostly reflect changes in the method to account for stratospheric temperature adjustment, which, for the HFCs, is generally higher than the 10% assumption used in H2013 (Figure 4). Additional factors include the revised Pinnock et al. curve (see section 2.3) and, particularly for the short‐lived compounds, changes in lifetime which influence the factor to account for nonuniform distribution in the atmosphere (see section 2.4). Since H2013, RE values have been added for 14 compounds, of which four compounds are based on calculations using absorption spectra from PNNL.

#### 3.3.4 Chlorocarbons and Hydrochlorocarbons

Among the 33 compounds in this category, only one (1,2‐dichloroethane) was included in H2013 (Table 5). For all these compounds, RE calculations are based on additional absorption spectra from the PNNL database, and for 1,2‐dichloroethane the RE value of 0.01 W m^−2^ ppb^−1^ is the same as in H2013 (when rounded to two decimals) (Table S10). Atmospheric lifetimes for most of the compounds in Table 5 are not available and the RE values listed are most likely upper limits since a uniform distribution in the atmosphere is assumed.

#### 3.3.5 Bromocarbons, Hydrobromocarbons, and Halons

Six compounds in this category were also included in H2013 and for two of these (dibromomethane and halon‐1202), new spectra from PNNL have been included in the RE calculations (Tables 5 and S11). The RE values remain unchanged for all six compounds (when rounded to two decimals). RE calculations have been made for 10 compounds in addition to those presented in H2013 and these are based on nine absorption spectra from PNNL and one from HITRAN2016.

#### 3.3.6 Fully Fluorinated Species

Among the 10 compounds that were included in H2013, three of the four compounds with a very long lifetime (800 years or more) have new RE values that are less than 5% different from H2013 (Tables 5 and S12). Octafluorocyclopentene was estimated to have a RE of 0.08 W m^−2^ ppb^−1^ in H2013, based on literature instantaneous RE values which were increased by 10% to crudely account for stratospheric temperature adjustment, and further adjusted by applying a lifetime correction. Since H2013 the lifetime of octafluorocyclopentene has been revised upward from 31 days to 1.1 years (WMO, 2019). We do not have the absorption spectrum and our recommended RE of 0.25 W m^−2^ ppb^−1^ is from N Zhang et al. (2017) who use a lifetime of 0.715 years. For the five remaining compounds with lifetimes of 31 days or less the new RE values are the same (after rounding) as in H2013 (see Table S12 for RE values with more significant figures).

Eight new compounds have been added since H2013 and one of the new compounds, heptacosafluorotributylamine/PFTBA, was recently observed in the Arctic (Schlabach et al., 2018). Its absorption spectrum has been measured in three recent studies (Bernard et al., 2018; Godin et al., 2016; Hong et al., 2013) and using the spectra from Hong et al. (2013) and Godin et al. (2016) we calculate a RE value of 0.91 W m^−2^ ppb^−1^. Bernard et al. (2018) measured spectra for three other perfluoroamines and report large RE values also for these compounds (in the range 0.61–0.95 W m^−2^ ppb^−1^). Lifetimes are estimated to be more than 1,000 years (WMO, 2019) and therefore these compounds are potent greenhouse gases. Two of the compounds added are based on RE values from the literature and RE for the remaining two compounds have been calculated using absorption spectra from PNNL. Heptafluoroisobutyronitrile is a potential replacement for sulfur hexafluoride and its atmospheric chemistry has been studied by Blazquez et al. (2017) and Andersen et al. (2017). The RE value of 0.25 W m^−2^ ppb^−1^ in Table 5 is an average of the REs from the two studies.

#### 3.3.7 Halogenated Alcohols and Ethers

Most of the 106 compounds in this category were also assessed in H2013. REs for 25 compounds were added in the present review (Tables 5 and S13) based on new absorption spectra or RE values. REs for 30 of the 106 compounds are based on additional absorption spectra from HITRAN2016 (13 spectra), PNNL (14 spectra), and Orkin et al. (2014) (3 spectra), and 10 of these compounds were also included in H2013. New absorption spectra have contributed to RE values that are significantly (>5%) different from H2013 for: HFE‐143a, 2,2,3,3,3‐pentafluoropropan‐1‐ol, difluoro (methoxy)methane, vinyl 2,2,2‐trifluoroacetate, ethyl 2,2,2‐trifluoroacetate, allyl 2,2,2‐trifluoroacetate, 2‐fluoroethanol, and 2,2,2‐trifluoroethanol. For some of these compounds a change in estimated lifetime is the main contributor to the change in RE (through the fractional correction factor).

We note that in H2013, three compounds were accidently listed twice with the same CASRN, but with different lifetime, RE, and GWP(100). The compounds are HG‐02, HG‐03, and 2,2,3,3,4,4,4‐Heptafluoro‐1‐butanol (CF_3_CF_2_CF_2_CH_2_OH), with CAS numbers 205367‐61‐9, 173350‐37‐3, and 375‐01‐9, respectively. This has been corrected in Tables 5 and S13. Six compounds were given slightly erroneous GWP(100) values in H2013: HG‐20, HG‐30, HG'‐02, HG'‐03, (CF_3_)_2_CHOCHO, and HG‐04 due to an error in their assigned molecular weights. Their lifetimes and REs were not affected. Their GWP(100) values have been corrected in Tables 5 and S13 (HG‐20 and HG‐30 are not included because of missing experimental spectra).

#### 3.3.8 Other Compounds

In addition to the seven compound groups listed in Tables 3 and 5, RE values for the following eight compound groups not considered in H2013 are presented in the supporting information: hydrocarbons (Table S14), alcohols, ethers, and other oxygenated hydrocarbons (Table S15), iodocarbons and hydroiodocarbons (Table S16), nitriles, amines and other nitrogenated hydrocarbons (Table S17), sulfur‐containing compounds (Table S18), silicon‐containing compounds (Table S19), and other compounds (Table S20). In contrast to the compounds presented in Tables S1–S13, the previous literature has not been reviewed for these compounds. Rather, RE values presented in Tables S14–S20 are for the most part purely from calculations based on available absorption spectra from the HITRAN2016 and PNNL databases. It is also important to note that the RE values assume, with a few exceptions, constant horizontal and vertical atmospheric distribution and are thus regarded as upper estimates.

## 4 Summary and Conclusions

We present a comprehensive assessment of the radiative efficiencies and GWPs for a large number of halocarbons and other weak atmospheric absorbers. The present work is an update of our review in H2013 where a consistent method for calculating RE was used for all compounds. A major advantage of using a common method for calculating REs is that the RE and GWP values for different compounds can be directly compared. This method has now been updated, and best estimate RE values have been calculated based on approximately 700 experimental absorption cross sections, versus 200 in H2013. The majority of the new spectra have been obtained from the HITRAN2016 and PNNL databases which were not included in our previous review.

**Figure 7:**
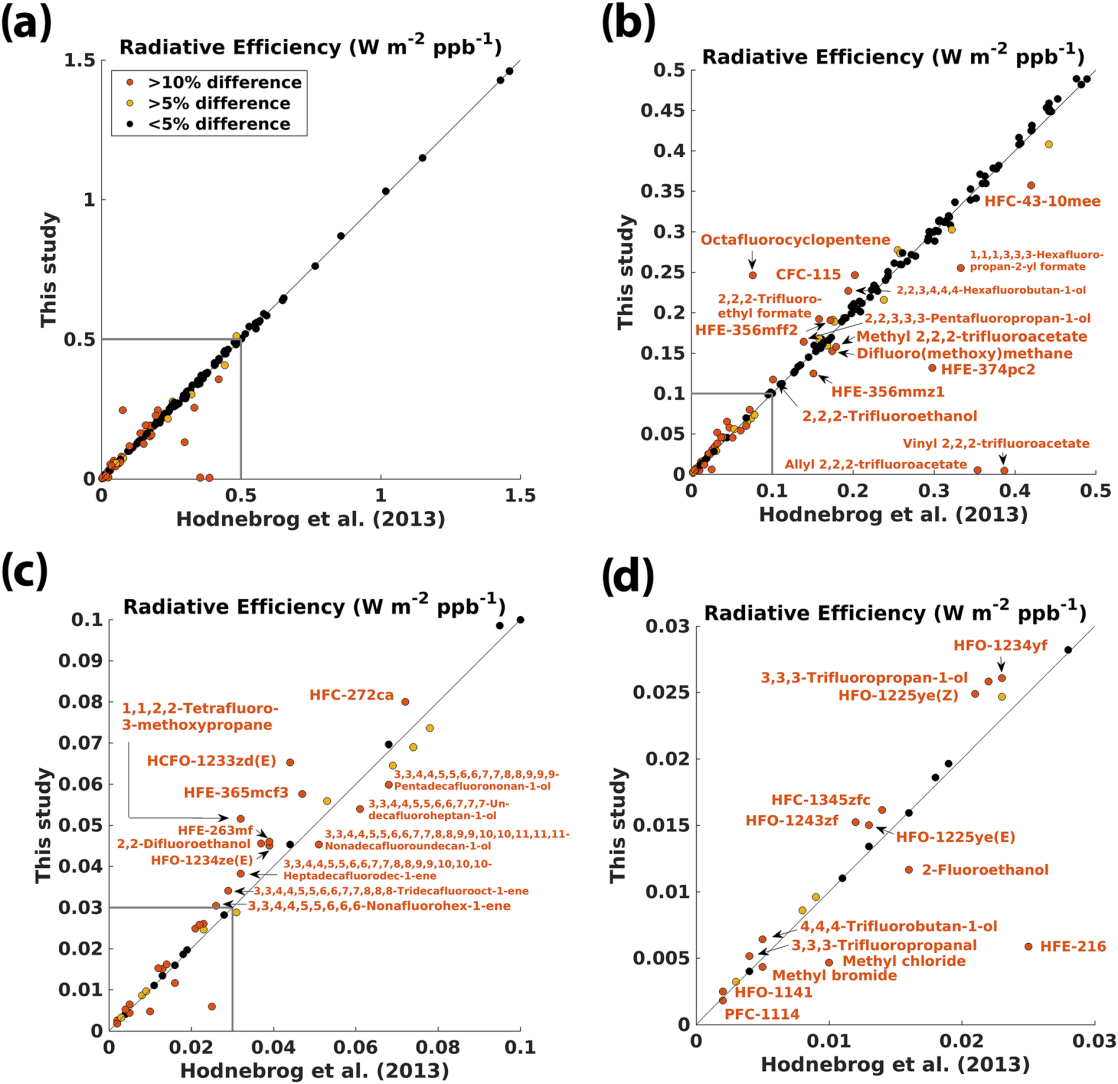
Comparison of radiative efficiencies (W m^−2^ ppb^−1^) calculated in this study (lifetime‐corrected adjusted cloudy‐sky) and from H2013 for (a) all compounds and (b)–(d) zoomed in using different scales for the RE. Black dots represent compounds where the RE in this study is less than 5% different from H2013, while yellow and red dots represent compounds where the REs are significantly different (>5% and >10%, respectively). Red dots have been labeled and represent compounds where the RE calculated here is more than 10% different from H2013.

Best estimate REs based on experimental spectra are now provided for more than 600 compounds compared to 168 compounds in H2013 (221 compounds when including REs based on calculated spectra). Most of the REs are based on our calculations, while some are based on published values. Figure 7 shows a comparison of our updated RE values with those presented in H2013 (and used in IPCC AR5; Myhre et al., 2013) for the 177 compounds included in both studies. For compounds with RE > 0.5 W m^−2^ ppb^−1^, changes are less than 5%. For compounds with RE < 0.5 W m^−2^ ppb^−1^, 61 compounds have RE values which differ by more than 5% from H2013, and 42 differ by more than 10%.

We have adopted recommended atmospheric lifetimes from the literature and, when available, calculated GWP values. In the main part of the paper (Tables 3 and 5) we have chosen to show only GWPs for a 100‐year time horizon in addition to the lifetimes and RE values. However, many metrics exist, and the choice of metric and time horizon depends on the context in which they are used (see section 2.5). Table 16 of H2013 presented GWP values for 20, 100, and 500 year time horizons in addition to GTPs for 20, 50, and 100 year time horizons for a selection of compounds. Table 6 shows updated numbers for these compounds, and (A)GWP and (A)GTP values for the same time horizons are given for all compounds in the supporting information.

**Table 6:** GWP and GTP for Selected Gases

				GWP			GTP		
Identifier/name	Formula	Lifetime (yr)	RE (W m^−2^ ppb^−1^)	20‐yr	100‐yr	500‐yr	20‐yr	50‐yr	100‐yr
CFC‐11	CCl_3_F	52.0	0.26	7,720	5,870	2,060	7,930	6,020	3,410
CFC‐12	CCl_2_F_2_	102.0	0.32	11,800	11,800	5,610	12,900	12,600	1,000
CFC‐113	CCl_2_FCClF_2_	93.0	0.30	7,130	6,900	3,120	7,700	7,330	5,620
HCFC‐22	CHClF_2_	11.9	0.21	5,900	2,060	616	4,000	814	419
HCFC‐141b	CH_3_CCl_2_F	9.4	0.16	2,800	903	270	1,640	275	180
HCFC‐142b	CH_3_CClF_2_	18.0	0.19	5,720	2,410	725	4,680	1,510	564
HFC‐23	CHF_3_	228.0	0.19	12,900	15,500	11,600	14,400	16,400	16,300
HFC‐134a	CH_2_FCF_3_	14.0	0.17	4,300	1,600	480	3,160	767	337
HFC‐152a	CH_3_CHF_2_	1.6	0.10	607	172	52	76	37	33
1,1,1‐Trichloroethane	CH_3_CCl_3_	5.0	0.06	585	169	51	190	37	33
Tetrachloromethane	CCl_4_	32.0	0.17	3,960	2,310	724	3,770	2,110	880
Sulfur hexafluoride	SF_6_	3,200.0	0.57	19,100	26,700	37,600	21,900	27,700	32,900
PFC‐14	CF_4_	50,000.0	0.10	5,520	7,830	11,700	6,350	8,120	9,740

In principle, and as noted by H2013, it would be desirable to calculate the Effective Radiative Forcing (ERF) (Myhre et al., 2013) which includes rapid adjustments beyond stratospheric temperature; ERF better represents the ultimate impact of a gas on surface temperature. It remains impracticable to calculate ERF for the gases discussed here (see Shine and Myhre, 2020 for discussion) because ERF requires computationally expensive calculations using general circulation models. The radiation codes in these models do not have sufficient spectral resolution to properly represent differences between the many halocarbons presented here, and the model's unforced variability would be much larger than the RF at their current, or likely future, concentrations. Although excellent progress has been made in understanding the generic nature of rapid adjustments and intermodel differences for many climate forcing agents (e.g., Smith et al., 2018), this has not yet extended to the halocarbons in a way that would allow a reliable generic correction to be made to the RFs calculated here.

One interesting potential consequence of revisions in the GWP(100) of halocarbons is the impact on existing legislation. For example, the European Union's legislation on the usage of fluorinated greenhouse gases (EUR‐Lex, 2014), in part, puts restrictions on usages of gases based on their GWP(100); it places dates on prohibition of marketing certain equipment which uses products with GWP(100) values exceeding 150, 1,500, and 2,500. Within that legislation, the GWP(100) values are clearly defined as those mostly originating from AR4 (Forster et al., 2007), but it does not appear to account for the uncertainties inherent in those GWP(100) values. Some of the updates presented here would push gases that were on one side of these GWP(100) boundaries to the other side. For example, HFC‐152a has now breached the 150 boundary (172 compared to 138), and HFC‐134a has breached the 1,500 boundary (1,600 compared to 1,300). Hence, future updates to legislation would either have to stick to using outdated values, amend the boundaries between allowed and prohibited fluorinated gases, or else decide that some gases that were previously accepted for certain usages, are no longer so. Similarly, Japan's “Act on Rational Use and Proper Management of Fluorocarbons” has target values for GWP(100) (at values of 100, 150, 750, and 1,500) for different products (MEGJ, 2016) as does Canada's “Ozone‐depleting Substances and Halocarbon Alternatives Regulations” (MJGC, 2019), with various products limited at different values of GWP(100) (150, 750, 1,400, 1,500, and 2,200). Table 7 gives an overview of the six (out of the 40 most abundant) compounds that enter a new policy category due to our updated GWP(100) values. All six compounds show higher GWP(100) than in H2013, and updates to lifetimes and the AGWP_CO2_(100) explain most of the increase for these compounds. A considerable part of the GWP(100) increase due to updated AGWP_CO2_(100) (around 5% out of the 14%) arises solely because of increasing CO_2_ concentrations and thereby reduced radiative efficiency of CO_2_ since H2013 (see section 2.5). Hence, it can be anticipated that continued accumulation of CO_2_ in the atmosphere will lead to changes in GWP values for weak atmospheric absorbers also in future updates.

**Table 7:** List of compounds (among the 40 most abundant presented in Table 3) that enter new policy categories due to updated GWP(100) values

Identifier/name	Formula	H2013 GWP(100)	New GWP(100)	Policy category change	Reasons for GWP(100) change (%)				
					AGWP_CO2_	τ	IRF curve	STA	Spectra
HCFC‐142b	CH_3_CClF_2_	1,980	2,410	Exceeds the 2,200 threshold in Canada.	+14	+5	−1	+3	+1
HFC‐32	CH_2_F_2_	677	809	Exceeds the 750 threshold in Japan and Canada	+14	+4	−1	0	+2
HFC‐134a	CH_2_FCF_3_	1,300	1,600	Exceeds the 1,500 threshold in EU, Japan and Canada	+14	+5	0	+2	+2
HFC‐152a	CH_3_CHF_2_	138	172	Exceeds the 150 threshold in EU, Japan and Canada	+14	+8	−1	+2	+1
Carbon tetrachloride	CCl_4_	1,730	2,310	Exceeds the 2,200 threshold in Canada	+14	+21	−2	−1	0
Halon‐2402	CBrF_2_CBrF_2_	1,470	2,280	Exceeds the 1,500 threshold in EU and Japan, and the 2,200 threshold in Canada	+14	+38	−1	−1	+1

*Note*. See section 4 for a discussion of, and references to, the policies that are referred to here, and section 3.1 for discussion of changes in lifetime and RE estimates. The rightmost column shows the contribution of change in GWP(100) due to the different factors: new AGWP_CO2_, new lifetime estimate (*τ*), new instantaneous RF “Pinnock curve” (IRF curve), new method to account for stratospheric temperature adjustment (STA), and addition of absorption spectra.

Finally, we have combined our new updated RE values with present‐day atmospheric concentrations of halocarbons to determine their radiative forcing. We find that the most abundant halocarbons cause a present‐day RF of 0.38 [0.33 to 0.43] W m^−2^, compared to 0.36 [0.32 to 0.40] W m^−2^ in AR5 (Myhre et al., 2013) and this is almost 20% of the preindustrial (1750) to present‐day (2019) RF due to CO_2_. Most of the increase in halocarbon forcing since AR5 can be attributed to increasing concentrations of CFC replacement compounds (HCFCs and HFCs) which more than outweigh the forcing due to decreasing concentrations of CFCs. However, the stronger halocarbon RF is also a consequence of updated RE values, which are slightly higher compared to AR5 for some of the most abundant compounds.

## Supporting information

Supporting Information S1

Table S1
