# Supplementary material for: Updated Global Warming Potentials and Radiative Efficiencies of Halocarbons and Other Weak Atmospheric Absorbers
Source: Rev Geophys. 2020 Sep 7;58(3):e2019RG000691. doi: 10.1029/2019RG000691 (PMC7518032; doi:10.1029/2019RG000691)
Supplement: Supplementary file 1 — Supporting Information S1 [file ROG-58-e2019RG000691-s002.pdf]

Reviews of Geophysics

Supporting Information for

Updated global warming potentials and radiative efficiencies of halocarbons and other weak atmospheric absorbers

Ø. Hodnebrog[1], B. Aamaas[1], J. S. Fuglestvedt[1], G. Marston[2], G. Myhre[1], C. J. Nielsen[3], M. Sandstad[1], K. P. Shine[4], T. J. Wallington[5]

[1]Center for International Climate Research (CICERO), P.O. Box 1129 Blindern, N-0318 Oslo, Norway

[2]Northumbria University, Newcastle, UK

[3]Department of Chemistry, University of Oslo, P.O. Box 1033 Blindern, N-0315 Oslo, Norway

[4]Department of Meteorology, University of Reading, Earley Gate, P.O. Box 243, Reading, RG6 6BB, Berks, UK

[5]Ford Motor Company, Research & Advanced Eng. Dept., Mail Drop RIC-2122, Dearborn, MI 48121-2053, USA

Contents of this file

Text S1 to S20
Tables S1 to S21

Additional Supporting Information (Files uploaded separately)

Spreadsheet in .csv format with 20-, 100-, and 500-year (absolute) GWP and 20-, 50-, and 100-year (absolute) GTP for all compounds in Tables S1-S20

Introduction

Additional text is given in Text S1 to S20, explaining why some absorption spectra have not been included in our calculations of best estimate RE for each compound.

Tables are given for each group of compounds, both for abundant compounds and separately for remaining compounds. The tables provide additional information to that given in Tables 2, 3 and 5 in the manuscript. For each compound, the 3rd column ("RE – This study") shows results using different versions of the Pinnock curve: the instantaneous RE curve from Hodnebrog et al. (2013) (hereafter H2013) ("H2013 inst. RE"), the new instantaneous RE curve from Shine and Myhre (2020) ("New inst. RE"), the new RE curve with stratospheric temperature adjustment included ("New RE – const. profile"), and the new RE curve with stratospheric temperature adjustment included and after applying lifetime correction factor ("New RE – lifetime corr.").

The last two rows for each compound give the RE and 100-yr GWP values used in H2013 and those calculated in this study, if relevant. The new recommended RE and 100-year GWP values are marked in bold. References to absorption spectra that have been used in our calculations of new best estimates are also marked in bold.

Table S21 provides structures of selected compounds, as referenced from Table 5 in the main manuscript.

Abbreviations used in Tables S1-S20:

Instantaneous/Adjusted:	I, Instantaneous RE; A, RE (with stratospheric temperature Adjustment included)
Vertical correction:	F, Freckleton et al. (1998); S, Sihra et al. (2001); H, Hodnebrog et al. (2013); M, Model based vertical profile; O, Other method; NC, Not Considered
RE calculation:	LBL, Line-By-Line code; NBM, Narrow Band Model; BBM, Broad Band Model; *k*-dist, correlated k-distribution model; Pinnock, original Pinnock et al. (1995) method; H2013, Hodnebrog et al. (2013) method
Database:	H08, HITRAN 2008; G09, GEISA 2009; P, PNNL; H16, HITRAN 2016

Table of Contents

Text S1. Most Abundant Chlorofluorocarbons 4
Text S2. Most Abundant Hydrochlorofluorocarbons 4
Text S3. Most Abundant Hydrofluorocarbons 4
Text S4. Most Abundant Chlorocarbons and Hydrochlorocarbons 5
Text S5. Most Abundant Bromocarbons, Hydrobromocarbons and Halons 6
Text S6. Most Abundant Fully Fluorinated Species 6
Text S7. Other Chlorofluorocarbons 6
Text S8. Other Hydrochlorofluorocarbons 6
Text S9. Other Hydrofluorocarbons 7
Text S10. Other Chlorocarbons and Hydrochlorocarbons 7
Text S11. Other Bromocarbons, Hydrobromocarbons and Halons 7
Text S12. Other Fully Fluorinated Species 7
Text S13. Halogenated Alcohols and Ethers 8
Text S14. Hydrocarbons 8
Text S15. Alcohols, ethers and other oxygenated hydrocarbons 8
Text S16. Iodocarbons and hydroiodocarbons 8
Text S17. Nitriles, amines and other nitrogenated hydrocarbons 8
Text S18. Sulfur containing compounds 9
Text S19. Silicon containing compounds 9
Text S20. Other compounds 9
Table S1. Most Abundant Chlorofluorocarbons 10
Table S2. Most Abundant Hydrochlorofluorocarbons 12
Table S3. Most Abundant Hydrofluorocarbons 13
Table S4. Most Abundant Chlorocarbons and Hydrochlorocarbons 16
Table S5. Most Abundant Bromocarbons, Hydrobromocarbons and Halons 17
Table S6. Most Abundant Fully Fluorinated Species 18
Table S7. Other Chlorofluorocarbons 20
Table S8. Other Hydrochlorofluorocarbons 21
Table S9. Other Hydrofluorocarbons 23
Table S10. Other Chlorocarbons and Hydrochlorocarbons 27
Table S11. Other Bromocarbons, Hydrobromocarbons and Halons 28
Table S12. Other Fully Fluorinated Species 29
Table S13. Halogenated Alcohols and Ethers 31
Table S14. Hydrocarbons 40
Table S15. Alcohols, ethers and other oxygenated hydrocarbons 42
Table S16. Iodocarbons and hydroiodocarbons 46
Table S17. Nitriles, amines and other nitrogenated hydrocarbons 47
Table S18. Sulfur containing compounds 48
Table S19. Silicon containing compounds 49
Table S20. Other compounds 50
Table S21. Structures of selected compounds 51

Text S1. Most Abundant Chlorofluorocarbons

RE calculations have been made for all available spectra – see Table S1 for overview of spectra and RE calculations. Some spectra have been excluded from the calculation of mean RE for the following reasons. **CFC-11:** Christidis et al. (1997) spectrum from the Ford laboratory is superseded by Sihra et al. (2001). Varanasi (2000, priv. comm.) spectrum in HITRAN2008 looks identical to Li and Varanasi (1994) in GEISA2009. **CFC-12:** The GEISA2009 spectrum from Hurley (2003, priv. comm.) and the Sihra et al. (2001) spectrum are from the Ford laboratory, and only one is used to avoid double-counting of absorption cross sections from the same laboratory group. Varanasi (2000, priv. comm.) spectrum in HITRAN2008 looks identical to Varanasi and Nemtchinov (1994) in GEISA2009 after visual inspection. **CFC-113, CFC-114 and CFC-115:** All available spectra included in mean RE value.

Text S2. Most Abundant Hydrochlorofluorocarbons

RE calculations have been made for all available spectra – see Table S2 for overview of spectra and RE calculations. Some spectra have been excluded from the calculation of mean RE calculation for the following reasons. **HCFC-22:** Hodnebrog et al. (2013) based their RE calculations on the recommended spectrum from Ballard et al. (2000b), who made a composite based on measurements from five different spectroscopy groups. The Ballard et al. (2000b) spectrum is also used here, but when calculating our mean RE, we have also included the spectrum from Harrison (2016) because it is the main choice on HITRAN2016 and because there were several improvements in the methods used to record the spectrum at the Molecular Spectroscopy Facility/Rutherford Appleton Laboratory (M.S.F./R.A.L.) (see Harrison (2016) for details on the improvements), and we included the spectrum from PNNL (Sharpe et al., 2004) because of the wider spectral range in that measurement. **HCFC-141b:** Pinnock et al. (1995) spectrum from Ford laboratory is superseded by Sihra et al. (2001). The spectrum from Harrison (2019) is not yet available from the HITRAN database. **HCFC-142b:** Pinnock et al. (1995) spectrum from the Ford laboratory is superseded by Sihra et al. (2001).

Text S3. Most Abundant Hydrofluorocarbons

RE calculations have been made for all available spectra – see Table S3 for overview of spectra and RE calculations. Some spectra have been excluded from the calculation of mean RE calculation for the following reasons. **HFC-23:** Sihra et al. (2001) spectrum from Ford laboratory is superseded by Gohar et al. (2004) (they also included lower wavenumbers). **HFC-32:** Pinnock et al. (1995) and Sihra et al. (2001) spectra from Ford laboratory are superseded by Gohar et al. (2004) (they also included lower wavenumbers). The Smith et al. (1996) and the GEISA2009 spectra are from M.S.F./R.A.L. and have not been included to avoid double-counting of absorption cross sections from the same laboratory groups (the Highwood and Shine (2000) spectrum is also from M.S.F./R.A.L.). **HFC-125:** Pinnock et al. (1995) and Sihra et al. (2001) spectra from Ford laboratory are superseded by Young et al. (2009b). Both the Di Lonardo and Masciarelli (2000) and Highwood and Shine (2000) spectra are from the University of Bologna, thus only one is included to avoid double-counting of absorption cross sections from the same laboratory groups. **HFC-134a:** Hodnebrog et al. (2013) based their RE calculations on the recommended spectrum from Forster et al. (2005), who made a

recommended spectrum based on measurements from six different spectroscopy groups. The Forster et al. (2005) spectrum is also used here, but when calculating our mean RE, we have also included the spectrum from PNNL (Sharpe et al., 2004) because they were not among the six laboratory groups included in the Forster et al. (2005) recommended spectrum. The spectrum from Harrison (2015b) gives essentially the same RE value as that derived using the PNNL spectrum (Table S3) because the absorption band intensities measured in Harrison (2015b) were calibrated using the PNNL spectrum. We did not include the Harrison (2015b) spectrum to avoid double-counting in the calculation of mean RE. **HFC-143a:** Pinnock et al. (1995) spectrum from the Ford laboratory is superseded by Sihra et al. (2001). The Di Lonardo and Masciarelli (2000) and Highwood and Shine (2000) spectra are from the University of Bologna, thus only one is included to avoid double-counting of absorption cross sections from the same laboratory group (see discussion in Section 4.1.3.8 of Hodnebrog et al. (2013) for explanation of differences between the two spectra). **HFC-152a:** Pinnock et al. (1995) spectrum from the Ford laboratory is superseded by Sihra et al. (2001). The Vander Auwera (2000) and Highwood and Shine (2000) spectra are from the Universitie Libre de Bruxelles, thus only one is included to avoid double-counting of absorption cross sections from the same laboratory group. **HFC-227ea:** Sihra et al. (2001) spectrum from Ford laboratory is superseded by Gohar et al. (2004) (they also included lower wavenumbers). **HFC-236fa, HFC-245fa, HFC-365mfc and HFC-43-10-mee:** All available spectra included in mean RE value.

Text S4. Most Abundant Chlorocarbons and Hydrochlorocarbons

RE calculations have been made for all available spectra – see Table S4 for overview of spectra and RE calculations. Some spectra have been excluded from the calculation of mean RE calculation for the following reasons. **CH_3CCl_3:** All available spectra included in mean RE value. **CCl_4:** Wallington et al. (2016) provided a recommended spectrum based on a scaling of the PNNL (Sharpe et al., 2004) spectrum to match the integrated absorption cross section derived from new and old laboratory measurements. This recommended spectrum is used here and the original PNNL spectrum is not included in the calculation of mean RE to avoid double-counting. The Nemtchinov and Varanasi (2003) spectrum and the identical Varanasi (2000, priv. comm.) spectrum in HITRAN2008, have also not been included because their spectra were accounted for when deriving the recommended spectrum in Wallington et al. (2016). The Harrison et al. (2017) spectrum has also not been included in the calculation of mean RE here because it was calibrated to a data set (Chu et al., 1999) that was accounted for when deriving the recommended spectrum in Wallington et al. (2016). **CH_3Cl, CH_2Cl_2 and $CHCl_3$:** Wallington et al. (2016) provided recommended spectra based on scaling the PNNL (Sharpe et al., 2004) spectra to match the integrated absorption cross section derived from literature laboratory measurements. These recommended spectra are used here and the original PNNL spectra are not included in the calculation of mean RE to avoid double-counting. For $CHCl_3$, the McPheat and Duxbury (2000) spectrum was not included because it was accounted for when deriving the recommended spectrum in Wallington et al. (2016).

Text S5. Most Abundant Bromocarbons, Hydrobromocarbons and Halons

RE calculations have been made for all available spectra – see Table S5 for overview of spectra and RE calculations. None of the available spectra were excluded when calculating the mean RE value for each compound.

Text S6. Most Abundant Fully Fluorinated Species

RE calculations have been made for all available spectra – see Table S6 for overview of spectra and RE calculations. Some spectra have been excluded from the calculation of mean RE calculation for the following reasons. **NF_3:** All available spectra included in mean RE value. **SF_6:** The two HITRAN2008 spectra are from the same laboratory and therefore the spectrum closest to room temperature has been used, i.e. the 216 K spectrum from Varanasi et al. (1994) was excluded to avoid double-counting of absorption cross sections from the same laboratory groups. **SO_2F_2:** All available spectra included in mean RE value. **PFC-14:** The GEISA2009 spectrum (Hurley, 2003, priv. comm.) and Sihra et al. (2001) spectrum from the Ford laboratory are superseded by Hurley et al. (2005) (only the Ford data in Hurley et al. (2005) were available and not the M.S.F./R.A.L. data). **PFC-116:** The Highwood and Shine (2000) and GEISA2009 spectra from M.S.F./R.A.L. are superseded by Bravo et al. (2010b). As noted in Hodnebrog et al. (2013), the Zou et al. (2004) spectrum does not include the absorption band located at 715 cm^{-1} but this had negligible impact on the RE, thus we keep this spectrum in our calculation of mean RE. One of the GEISA2009 spectra is listed with the reference Highwood et al. (1999) but that paper did not study PFC-116, thus the actual source of this spectrum is unknown and we have therefore not included it in the calculation of mean RE. **PFC-218:** All available spectra included in mean RE value. **PFC-C-318:** Two M.S.F./R.A.L. spectra are available, and we have used the published spectrum from Highwood and Shine (2000) and not the one from GEISA2009. **PFC-31-10:** Sihra et al. (2001) spectrum from the Ford laboratory is superseded by Bravo et al. (2010b). **PFC-41-12, PFC-51-14, PFC-61-16 and PFC-71-18:** All available spectra included in mean RE value.

Text S7. Other Chlorofluorocarbons

RE calculations have been made for all available spectra – see Table S7 for overview of spectra and RE calculations. Some spectra have been excluded from the calculation of mean RE calculation for the following reasons (only compounds where spectra have been excluded are mentioned). **CFC-13:** The spectrum from Massie et al. (1991) is the same as from McDaniel et al. (1991).

Text S8. Other Hydrochlorofluorocarbons

RE calculations have been made for all available spectra – see Table S8 for overview of spectra and RE calculations. Some spectra have been excluded from the calculation of mean RE calculation for the following reasons (only compounds where spectra have been excluded are

mentioned). **HCFC-21:** The Massie et al. (1985) spectrum has been excluded because it only contains one of the absorption bands (785-840 cm^{-1}). **HCFC-123 and HCFC-124:** Pinnock et al. (1995) spectrum from the Ford laboratory is superseded by Sihra et al. (2001).

Text S9. Other Hydrofluorocarbons

RE calculations have been made for all available spectra – see Table S9 for overview of spectra and RE calculations. Some spectra were excluded from the calculation of mean RE calculation for the following reasons (only compounds where spectra have been excluded are mentioned). **HFC-134:** M.S.F./R.A.L. spectrum in GEISA2009 looks identical to Smith et al. (1998) in HITRAN2008. The GEISA2009 spectrum from Hurley (2003, priv. comm.) and the Sihra et al. (2001) spectrum are from the Ford laboratory, and only one is used to avoid double-counting of absorption cross sections from the same laboratory group.

Text S10. Other Chlorocarbons and Hydrochlorocarbons

RE calculations have been made for all available spectra – see Table S10 for overview of spectra and RE calculations. None of the available spectra have been excluded when calculating the mean RE value for each compound.

Text S11. Other Bromocarbons, Hydrobromocarbons and Halons

RE calculations have been made for all available spectra – see Table S11 for overview of spectra and RE calculations. None of the available spectra were excluded when calculating the mean RE value for each compound.

Text S12. Other Fully Fluorinated Species

RE calculations have been made for all available spectra – see Table S12 for overview of spectra and RE calculations. Some spectra have been excluded from the calculation of mean RE calculation for the following reasons (only compounds where spectra have been excluded are mentioned). **SF_5CF_3:** The spectrum from the PNNL database (Sharpe et al., 2004) was not included to avoid double-counting of absorption cross sections from the same laboratory group (the Rinsland et al. (2003) spectrum is also measured at PNNL). The GEISA2009 spectrum from Hurley (2003, priv. comm.) and the Nielsen et al. (2002) spectrum are from the Ford laboratory, and only one is used to avoid double-counting of absorption cross sections from the same laboratory group.

Text S13. Halogenated Alcohols and Ethers

RE calculations have been made for all available spectra – see Table S13 for overview of spectra and RE calculations. Some spectra have been excluded from the calculation of mean RE calculation for the following reasons (only compounds where spectra have been excluded are mentioned). **HCFE-235da2 (isoflurane):** Sihra et al. (2001) spectrum from the Ford laboratory is superseded by M P S Andersen et al. (2010c). **HFE-43-10pccc124 (HGalden 1040x, HG-11):** Sihra et al. (2001) spectrum from the Ford laboratory is superseded by Wallington et al. (2009). The spectrum from Cavalli et al. (1998) has not been used because it is an overestimate (see Wallington et al. (2009) for details).

Text S14. Hydrocarbons

RE calculations have been made for all available spectra – see Table S14 for overview of spectra and RE calculations. Some spectra have been excluded from the calculation of mean RE calculation for the following reasons (only compounds where spectra have been excluded are mentioned). **Ethane:** Harrison et al. (2010) spectrum does not contain all absorption bands. **Propane:** Harrison and Bernath (2010) spectrum does not contain all absorption bands.

Text S15. Alcohols, ethers and other oxygenated hydrocarbons

RE calculations have been made for all available spectra – see Table S15 for overview of spectra and RE calculations. Some spectra have been excluded from the calculation of mean RE calculation for the following reasons (only compounds where spectra have been excluded are mentioned). **Acetaldeyde:** Tereszchuk and Bernath (2011) spectrum does not contain all absorption bands. **2-Pentylfuran:** The HITRAN2016 and the PNNL spectra are from the same laboratory and we have chosen the one closest to room temperature.

Text S16. Iodocarbons and hydroiodocarbons

RE calculations have been made for all available spectra (from PNNL (Sharpe et al., 2004)) – see Table S16 for overview of spectra and RE calculations. None of the available spectra have been excluded when calculating the mean RE value for each compound.

Text S17. Nitriles, amines and other nitrogenated hydrocarbons

RE calculations have been made for all available spectra – see Table S17 for overview of spectra and RE calculations. Some spectra have been excluded from the calculation of mean RE calculation for the following reasons (only compounds where spectra have been excluded are mentioned). **Peroxyacetyl nitrate:** Allen et al. (2005) spectrum does not contain all absorption bands in HITRAN2016.

Text S18. Sulfur containing compounds

RE calculations have been made for all available spectra (from PNNL (Sharpe et al., 2004)) – see Table S18 for overview of spectra and RE calculations. None of the available spectra have been excluded when calculating the mean RE value for each compound.

Text S19. Silicon containing compounds

RE calculations have been made for all available spectra (from PNNL (Sharpe et al., 2004)) – see Table S19 for overview of spectra and RE calculations. None of the available spectra have been excluded when calculating the mean RE value for each compound.

Text S20. Other compounds

RE calculations have been made for all available spectra – see Table S20 for overview of spectra and RE calculations. None of the available spectra have been excluded when calculating the mean RE value for each compound.

Table S1. Most Abundant Chlorofluorocarbons

Please refer to page 2 of this document for a description of the table.

				Lifetime (yr)			RE (W m^{-2} ppb^{-1}) – This study				RE (W m^{-2} ppb^{-1}) – Literature					GWP 100-yr		Absorption cross-sections						
Name	CASRN	Identifier	Formula	H2013	New	Reference	H2013 inst. RE	New inst. RE	New RE – const. profile	New RE – lifetime corr.	Value	Reference	Instantaneous/Adjusted	Vertical correction	RE calculation	H2013	New – lifetime corr.	*T* (K)	*p* (hPa)	Waveno. range (cm^{-1})	Int. abs. cross-section (10^{-17} cm^2 molecule^{-1} cm^{-1})	Reference	Database	Notes
Trichlorofluoromethane	75-69-4	CFC-11	CCl_3F	45.0	52.0	*WMO* (2019)	0.269	0.263	0.291	0.269		**(Sharpe et al., 2004)**					6,090	298	1013	570-3000	10.1		H16	
							0.250	0.244	0.270	0.251		**(Orkin et al., 2003)**	I	NC			5,670	295		820-1120	9.3			
							0.249	0.244	0.270	0.250	0.242	**(Sihra et al., 2001)**	A	O	LBL/NBM		5,650	296	933	650-1500	9.3			
											0.240	(Jain et al., 2000)	A	M	NBM							(McDaniel et al., 1991)		
											0.242	(Naik et al., 2000)	A	M										
											0.260	(Ninomiya et al., 2000)	I		Pinnock									
							0.257	0.251	0.278	0.258		(Varanasi-priv.com., 2000)					5,830	296	1013	810-1120	9.5		H08	
											0.245	(Good et al., 1998)			NBM							(McDaniel et al., 1991; Varanasi and Chudamani, 1988a)		(1)
							0.266	0.260	0.287	0.266	0.290	**(Heathfield et al., 1998)**	I		Pinnock		6,020	298		800-1120	9.4		G09	
											0.250	(Myhre et al., 1998)	A	O	LBL/NBM/BBM							(Christidis et al., 1997)		
											0.252	(Hansen et al., 1997)										(Li and Varanasi, 1994)		
											0.250	(Myhre and Stordal, 1997)	A		BBM							(HITRAN, 1992)		
											0.269	(Pinnock et al., 1995)	A									(HITRAN, 1992)		
							0.248	0.242	0.268	0.248	0.285	(Christidis et al., 1997)	A	NC	NBM		5,620	296	921	810-1120	9.0		G09	
							0.262	0.256	0.283	0.262		**(Imasu et al., 1995)**					5,940	296		700-1500	9.8			
							0.257	0.251	0.278	0.258		**(Li and Varanasi, 1994)**					5,830	296	1013	810-1120	9.5		G09	
												(McDaniel et al., 1991)						293		800-1120	9.2			
											0.220	(Fisher et al., 1990)								810-1120	8.9			
												(Varanasi and Chudamani, 1988a)								800-1120	9.8			
												(Massie et al., 1985)								800-1120	8.4			
												(Kagann et al., 1983)								800-1120	10.3			
												(Nanes et al., 1980)								800-1120	8.9			
												(Varanasi and Ko, 1977)								800-1120	8.9			
											0.260	H2013				4,660								
							0.259	0.253	0.280	**0.259**		**This study (avg.)**					**5,870**							
Dichlorodifluoromethane	75-71-8	CFC-12	CCl_2F_2	100.0	102.0	*WMO* (2019)	0.306	0.299	0.337	0.327		**(Harrison, 2015a)**					12,000	294	467	800-1270	13.5		H16	
							0.308	0.302	0.339	0.329	0.328	**(Myhre et al., 2006)**	A	O	LBL		12,100	295		850-1190	13.5			
							0.306	0.300	0.337	0.327		**(Sharpe et al., 2004)**					12,100	296		600-3000	13.9		P	
							0.274	0.269	0.302	0.293		(Hurley-priv.com., 2003)					10,800	296	933	640-1200	12.2		G09	
							0.290	0.284	0.319	0.310	0.299	**(Orkin et al., 2003)**	I	NC			11,400	295		850-1190	12.9			(2)
							0.273	0.268	0.301	0.292	0.321	**(Sihra et al., 2001)**	A	O	LBL/NBM		10,800	296	933	500-1500	12.2			
											0.302	(Jain et al., 2000)	A	M	NBM							(McDaniel et al., 1991)		
							0.308	0.302	0.339	0.329		(Varanasi-priv.com., 2000)					12,100	296	1013	850-1200	13.6		H08	
											0.310	(Good et al., 1998)			NBM							(Clerbaux et al., 1993; McDaniel et al., 1991; Varanasi and Chudamani, 1988a)		(3)
												(Minschwaner et al., 1998)										(Massie et al., 1991)		
											0.330	(Myhre et al., 1998)										(HITRAN, 1992)		
											0.320	(Myhre and Stordal, 1997)	A		BBM							(HITRAN, 1992)		
											0.300	(Hansen et al., 1997)										(Morcillo et al., 1966)		
							0.308	0.302	0.339	0.329		**(Varanasi and Nemtchinov, 1994)**					12,100	296	1013	850-1200	13.6		G09	
							0.306	0.299	0.337	0.327		**(Clerbaux et al., 1993)**					12,000	287		850-1190	13.5		G09	
												(McDaniel et al., 1991)						293		850-1190	12.6			
											0.333	(Fisher et al., 1990)						296		850-1190	12.1			(2)
												(Varanasi and Chudamani, 1988a)						300		850-1190	13.4			
												(Vanthanh et al., 1986)						298		800-1200	12.7			
												(Massie et al., 1985)						296		850-1190	13.3			
												(Kagann et al., 1983)						296		850-1190	13.6			
												(Varanasi and Ko, 1977)						300		850-1190	12.9			
												(Morcillo et al., 1966)												
											0.318	H2013				10,200								
							0.300	0.293	0.330	**0.320**		**This study (avg.)**					**11,800**							

Table S1 (cont.)

Name	CASRN	Identifier	Formula	Lifetime (yr) H2013	Lifetime (yr) New	Lifetime (yr) Reference	RE ($W\ m^{-2}\ ppb^{-1}$) – This study: H2013 inst. RE	New inst. RE	New RE – const. profile	New RE – lifetime corr.	RE ($W\ m^{-2}\ ppb^{-1}$) – Literature: Value	Reference	Instantaneous/Adjusted	Vertical correction	RE calculation	GWP 100-yr: H2013	New – lifetime corr.	Absorption cross-sections: T (K)	p (hPa)	Waveno. range (cm^{-1})	Int. abs. cross-section ($10^{-17}\ cm^2\ molecule^{-1}\ cm^{-1}$)	Reference	Database	Notes
1,1,2-Trichloro-1,2,2-trifluoroethane	76-13-1	CFC-113	CCl_2FCClF_2	85.0	93.0	*WMO* (2019)	0.294	0.288	0.315	0.302		**(Le Bris et al., 2011)**					6,910	283		600-1250	13.7			
							0.307	0.301	0.329	0.316		**(Sharpe et al., 2004)**					7,230	298	1013	620-3000	14.6		H16	
											0.284	(Jain et al., 2000)	A	M	NBM							(McDaniel et al., 1991)		
											0.304	(Myhre and Stordal, 1997)	A		BBM							(HITRAN, 1992)		
												(Olliff and Fischer, 1994)						295		450-3500	12.7			
							0.279	0.273	0.298	0.286		**(McDaniel et al., 1991)**					6,550	293		780-1232	12.7		H08	
											0.333	(Fisher et al., 1990)								618-1397	12.7			(2)
												(Rogers and Stephens, 1988)								780-1235	19.4			
												(Varanasi and Chudamani, 1988b)								780-1235	14.1			
											0.302	H2013				5,820								
							0.293	0.288	0.314	**0.301**		**This study (avg.)**					**6,900**							
1,2-Dichloro-1,1,2,2-tetrafluoroethane	76-14-2	CFC-114	$CClF_2CClF_2$	190.0	189.0	*WMO* (2019)	0.315	0.310	0.339	0.328		**(Sharpe et al., 2004)**					10,400	298	1013	600-3000	17.4		H16	
											0.290	(Jain et al., 2000)	A	M	NBM							(McDaniel et al., 1991)		
											0.307	(Myhre and Stordal, 1997)	A		BBM							(HITRAN, 1992)		
												(Olliff and Fischer, 1994)						295		450-3500	14.8			
							0.288	0.284	0.311	0.301		**(McDaniel et al., 1991)**					9,560	293		815-1285	15.2		H08	
											0.385	(Fisher et al., 1990)								555-1397	15.4			(2)
												(Rogers and Stephens, 1988)								820-1310	23.9			
												(Varanasi and Chudamani, 1988b)								820-1310	15.8			
												(Massie et al., 1985)								1025-1310	12.0			
											0.307	H2013				8,590								
							0.301	0.297	0.325	**0.314**		**This study (avg.)**					**9,990**							
1-Chloro-1,1,2,2,2-pentafluoroethane	76-15-3	CFC-115	$CClF_2CF_3$	1020.0	540.0	*WMO* (2019)	0.195	0.194	0.218	0.213	0.210	**(Totterdill et al., 2016)**	A	M	LBL		8,810	296		946-1368	11.9			
							0.294	0.293	0.328	0.321		(Sharpe et al., 2004)					13,300	323		525-3000	20.1		P	
							0.294	0.293	0.328	0.321		(Sharpe et al., 2004)					13,300	278		525-3000	20.1		P	
							0.294	0.293	0.328	0.321		**(Sharpe et al., 2004)**					13,300	296		525-3000	20.1		P	
											0.214	(Jain et al., 2000)	A	M	NBM							(McDaniel et al., 1991)		
											0.197	(Myhre and Stordal, 1997)	A		BBM							(HITRAN, 1992)		
												(Olliff and Fischer, 1994)						295		450-3500	17.1			
							0.187	0.186	0.209	0.205		**(McDaniel et al., 1991)**					8,460	293		955-1260	12.1		H08	
											0.302	(Fisher et al., 1990)								618-1397	17.4			(2)
											0.202	H2013				7,670								
							0.241	0.240	0.269	**0.263**		**This study (avg.)**					**10,200**							

(1) Average of results using two different spectra; (2) Scaled to the CFC-11 RE of 0.26 $Wm^{-2}\ ppb^{-1}$; (3) Average of results using three different spectra

Table S2. Most Abundant Hydrochlorofluorocarbons

Please refer to page 2 of this document for a description of the table.

				Lifetime (yr)			RE (W m^{-2} ppb^{-1}) – This study				RE (W m^{-2} ppb^{-1}) – Literature					GWP 100-yr		Absorption cross-sections						
Name	CASRN	Identifier	Formula	H2013	New	Reference	H2013 inst. RE	New inst. RE	New RE – const. profile	New RE – lifetime corr.	Value	Reference	Instantaneous/Adjusted	Vertical correction	RE calculation	H2013	New – lifetime corr.	*T* (K)	*p* (hPa)	Waveno. range (cm^{-1})	Int. abs. cross-section (10^{-17} cm^2 molecule^{-1} cm^{-1})	Reference	Database	Notes
Chlorodifluoromethane	75-45-6	HCFC-22	$CHClF_2$	11.9	11.9	*WMO* (2019)	0.204	0.201	0.224	0.215		**(Harrison, 2016)**					2,070	295	1015	730-1380	10.5		H16	
							0.206	0.203	0.226	0.217		**(Sharpe et al., 2004)**					2,090	296		550-3000	10.8		P	
							0.189	0.187	0.208	0.200	0.205	(Orkin et al., 2003)	I	NC			1,930	295		750-1380	10.0			(1)
							0.197	0.195	0.217	0.209	0.208	(Sihra et al., 2001)	A	O	LBL/NBM		2,010	296	933	700-1400	10.1			
							0.131	0.129	0.146	0.140		(Varanasi, priv.com., 2001; Varanasi et al., 1994)					1,340	296	1015	1070-1195	6.9		G09	
							0.197	0.195	0.218	0.209		**(Ballard et al., 2000b)**					2,010	293	800	775-1375	10.1		G09	
							0.199	0.196	0.219	0.210	0.200	(Highwood and Shine, 2000)	A	O	NBM		2,020	273		750-1400	10.2			
											0.205	(Jain et al., 2000)	A	M	NBM							(McDaniel et al., 1991)		
											0.196	(Naik et al., 2000)	A	M										
											0.216	(Good et al., 1998)			NBM							(Clerbaux et al., 1993; McDaniel et al., 1991; Varanasi and Chudamani, 1988b)		(2)
											0.177	(Myhre and Stordal, 1997)	A		BBM							(HITRAN, 1992)		
							0.199	0.196	0.219	0.210	0.227	(Pinnock et al., 1995)	A		NBM		2,020	296	933	765-1390	10.2		G09	
												(Anastasi et al., 1994)						296	500	735-1380	9.7			
							0.200	0.198	0.221	0.212		(Clerbaux et al., 1993)					2,040	287		765-1380	10.3		H08	
												(Cappellani and Restelli, 1992)						293	1000	755-1390	10.3			
												(McDaniel et al., 1991)						293		775-1375	9.0			
											0.229	(Fisher et al., 1990)								775-1397	9.5			(1)
												(Varanasi and Chudamani, 1988b)								775-1170	9.4			
											0.208	H2013				1,770								
							0.202	0.200	0.223	**0.214**		**This study (avg.)**					**2,060**							
1,1-Dichloro-1-fluoroethane	1717-00-6	HCFC-141b	CH_3CCl_2F	9.2	9.4	*WMO* (2019)						(Harrison, 2019)						295		705-1280				
							0.160	0.158	0.172	0.164		**(Le Bris et al., 2012)**					923	283		570-1470	8.0			
							0.161	0.158	0.173	0.165		**(Sharpe et al., 2004)**					925	298	1013	550-3000	8.4		H16	
							0.157	0.155	0.169	0.161	0.177	**(Orkin et al., 2003)**	I	NC			905	295		540-1540	8.0			(1)
							0.142	0.140	0.154	0.146	0.130	**(Sihra et al., 2001)**	A	O	LBL/NBM		822	296	933	700-1500	7.1			
											0.131	(Jain et al., 2000)	A	M	NBM							(Pinnock et al., 1995)		
											0.118	(Naik et al., 2000)	A	M										
							0.164	0.161	0.177	0.169	0.157	**(Imasu et al., 1995)**	I	O			949	296		700-1500	8.3			(1)
							0.143	0.141	0.155	0.147	0.157	(Pinnock et al., 1995)	A		NBM		828	296	933	700-1470	7.2		G09	
												(Olliff and Fischer, 1994)						295		540-1480	7.6			
							0.155	0.152	0.167	0.159		**(Clerbaux et al., 1993)**					895	287		710-1470	7.8		H08	
											0.146	(Fisher et al., 1990)								555-1420	7.1			(1)
											0.162	H2013				782								
							0.156	0.153	0.168	**0.160**		**This study (avg.)**					**903**							
1-Chloro-1,1-difluoroethane	75-68-3	HCFC-142b	CH_3CClF_2	17.2	18.0	*WMO* (2019)	0.178	0.175	0.199	0.193		**(Le Bris and Strong, 2010)**					2,400	283		650-1500	10.8		H16	
							0.181	0.179	0.203	0.197		**(Sharpe et al., 2004)**					2,450	298	1013	600-3000	11.2		H16	
							0.170	0.168	0.190	0.184	0.163	**(Sihra et al., 2001)**	A	O	LBL/NBM		2,300	296	933	700-1500	9.6			
											0.164	(Jain et al., 2000)	A	M	NBM							(Pinnock et al., 1995)		
											0.176	(Naik et al., 2000)	A	M										
							0.168	0.166	0.188	0.182	0.210	(Pinnock et al., 1995)	A		NBM		2,280	296	933	650-1425	10.1		G09	
												(Olliff and Fischer, 1994)						295		450-3500	10.1			
							0.184	0.182	0.206	0.200		**(Clerbaux et al., 1993)**					2,500	287		650-1469	11.1		H08	
												(Cappellani and Restelli, 1992)						293	1000	647-1485	10.7			
											0.208	(Fisher et al., 1990)								649-1397	9.6			(1)
											0.189	H2013				1,980								
							0.178	0.176	0.199	**0.193**		**This study (avg.)**					**2,410**							

(1) Scaled to the CFC-11 RE of 0.26 Wm^{-2} ppb^{-1}; (2) Average of results using three different spectra

Table S3. Most Abundant Hydrofluorocarbons

Please refer to page 2 of this document for a description of the table.

				Lifetime (yr)			RE (W m^{-2} ppb^{-1}) – This study				RE (W m^{-2} ppb^{-1}) – Literature					GWP 100-yr		Absorption cross-sections						
Name	CASRN	Identifier	Formula	H2013	New	Reference	H2013 inst. RE	New inst. RE	New RE – const. profile	New RE – lifetime corr.	Value	Reference	Instantaneous/Adjusted	Vertical correction	RE calculation	H2013	New – lifetime corr.	*T* (K)	*p* (hPa)	Waveno. range (cm^{-1})	Int. abs. cross-section (10^{-17} cm^2 molecule^{-1} cm^{-1})	Reference	Database	Notes
Trifluoromethane	75-46-7	HFC-23	CHF_3	222.0	228.0	*WMO* (2019)	0.181	0.180	0.204	0.203		**(Harrison, 2013)**					16,400	294	474	950-1500	12.3		H16	
							0.174	0.173	0.195	0.194	0.187	**(Gohar et al., 2004)**	A	S			15,700	296		400-1500	11.8			(1)
							0.182	0.181	0.205	0.203		**(Sharpe et al., 2004)**					16,500	296		600-3000	12.7		P	
							0.172	0.171	0.194	0.193	0.171	(Sihra et al., 2001)	A	O	LBL/NBM		15,600	296	933	700-1500	11.7			
							0.147	0.147	0.166	0.164	0.160	**(Highwood and Shine, 2000)**	A	O	NBM		13,300	253		655-1415	10.1			
											0.248	(Jain et al., 2000)	A	M	NBM							(Pinnock et al., 1995)		
											0.271	(Naik et al., 2000)	A	M										
											0.231	(Pinnock et al., 1995)	A		NBM			296		700-1400	12.7			
											0.175	H2013				12,400								
							0.171	0.170	0.193	**0.191**		**This study (avg.)**					**15,500**							
Difluoromethane	75-10-5	HFC-32	CH_2F_2	5.2	5.4	*WMO* (2019)					0.127	(H Zhang et al., 2011a)		S								(HITRAN, 2004)		
							0.109	0.107	0.119	0.110	0.111	**(Gohar et al., 2004)**	A	S			798	296		400-1550	5.8			(1)
							0.116	0.115	0.127	0.117		**(Sharpe et al., 2004)**					852	298	1013	510-3000	7.0		H16	
							0.110	0.109	0.120	0.111	0.114	**(Orkin et al., 2003)**	I	NC			809	295		450-1480	5.9			(2)
							0.108	0.106	0.118	0.109	0.105	(Sihra et al., 2001)	A	O	LBL/NBM		791	296	933	700-1500	5.7			
							0.106	0.104	0.116	0.107	0.090	**(Highwood and Shine, 2000)**	A	O	NBM		778	253		450-1475	5.6			
											0.155	(Jain et al., 2000)	A	M	NBM							(Pinnock et al., 1995)		
											0.141	(Naik et al., 2000)	A	M										
							0.098	0.097	0.107	0.099		(Smith et al., 1996)					719	297	1000	995-1475	5.2		H08	
							0.109	0.107	0.119	0.110	0.136	(Pinnock et al., 1995)	A		NBM		798	296	933	700-1400	6.3		G09	
							0.098	0.097	0.107	0.099		(M.S.F./R.A.L.)					720	297	1000	995-1475	5.2		G09	
											0.110	H2013				677								
							0.110	0.109	0.120	**0.111**		**This study (avg.)**					**809**							
1,1,1,2,2-Pentafluoroethane	354-33-6	HFC-125	CHF_2CF_3	28.2	30.0	*WMO* (2019)					0.295	(H Zhang et al., 2011a)		S								(HITRAN, 2004)		
							0.197	0.197	0.223	0.219	0.210	**(Young et al., 2009b)**			Pinnock		3,690	296	933	700-1480	15.5			
							0.220	0.220	0.248	0.242		**(Sharpe et al., 2004)**					4,090	298	1013	510-3000	17.4		H16	
							0.215	0.214	0.241	0.236	0.255	**(Orkin et al., 2003)**	I	NC			3,980	295		460-1480	16.6			(2)
							0.212	0.211	0.239	0.234	0.223	(Sihra et al., 2001)	A	O	LBL/NBM		3,950	296	933	700-1500	16.5			
							0.224	0.224	0.252	0.247		(Di Lonardo and Masciarelli, 2000)					4,160	293	800	550-1480	17.2		G09	
							0.211	0.211	0.238	0.233	0.230	**(Highwood and Shine, 2000)**	A	O	NBM		3,930	253		550-1480	16.6			
											0.249	(Jain et al., 2000)	A	M	NBM							(Pinnock et al., 1995)		
											0.229	(Naik et al., 2000)	A	M										
							0.220	0.220	0.249	0.244	0.231	**(Imasu et al., 1995)**	I	O			4,110	296		700-1500	17.0			(2)
							0.210	0.210	0.237	0.232	0.251	(Pinnock et al., 1995)	A		NBM		3,920	296	933	700-1400	16.1		G09	
												(Olliff and Fischer, 1994)						295		460-1480	16.0			
							0.206	0.206	0.233	0.228		**(Clerbaux et al., 1993)**					3,850	287		700-1465	16.1		G09	
											0.281	(Fisher et al., 1990)								500-1307	14.5			(2)
											0.226	H2013				3,170								
							0.212	0.211	0.239	**0.234**		**This study (avg.)**					**3,940**							

Table S3 (cont.)

Name	CASRN	Identifier	Formula	Lifetime (yr) H2013	Lifetime (yr) New	Lifetime (yr) Reference	RE ($W\ m^{-2}\ ppb^{-1}$) – This study: H2013 inst. RE	New inst. RE	New RE – const. profile	New RE – lifetime corr.	RE ($W\ m^{-2}\ ppb^{-1}$) – Literature: Value	Reference	Instantaneous/Adjusted	Vertical correction	RE calculation	GWP 100-yr: H2013	New – lifetime corr.	Absorption cross-sections: T (K)	p (hPa)	Waveno. range (cm^{-1})	Int. abs. cross-section ($10^{-17}\ cm^2\ molecule^{-1}\ cm^{-1}$)	Reference	Database	Notes
1,1,1,2-Tetrafluoroethane	811-97-2	HFC-134a	CH_2FCF_3	13.4	14.0	*WMO* (2019)						(Harrison, 2015b)					1,640	296	1014	750-1600	13.2		H16	
											0.210	(H Zhang et al., 2011a)		S								(HITRAN, 2004)		
							0.152	0.151	0.170	0.164	0.160	**(Forster et al., 2005)**					1,570	296		250-2000	13.2			(3)
							0.151	0.151	0.169	0.163	0.161	(Gohar et al., 2004)	A	S			1,560	296		300-1550	13.1			(1)
							0.158	0.157	0.177	0.170		**(Sharpe et al., 2004)**					1,630	296		600-3000	14.2		P	
							0.156	0.155	0.174	0.168	0.192	(Orkin et al., 2003)	I	NC			1,610	295		490-1550	13.6			(2)
							0.148	0.147	0.166	0.160	0.159	(Sihra et al., 2001)	A	O	LBL/NBM		1,530	296	933	700-1550	12.4			
							0.156	0.155	0.174	0.168	0.150	(Highwood and Shine, 2000)	A	O	NBM		1,610	253		75-1540	13.6			
											0.200	(Jain et al., 2000)	A	M	NBM							(Pinnock et al., 1995)		
											0.222	(Naik et al., 2000)	A	M										
												(Newnham et al., 1996)								600-1448	13.5			
							0.161	0.160	0.181	0.174	0.171	(Imasu et al., 1995)	I	O			1,670	296		700-1500	13.5			(2)
											0.181	(Pinnock et al., 1995)	A		NBM			296		700-1400	13.1			
												(Olliff and Fischer, 1994)						295		490-1510	13.4			
							0.150	0.149	0.169	0.163		(Clerbaux et al., 1993)					1,560	287		815-1485	12.6		H08	
												(Cappellani and Restelli, 1992)						293	1000	610-1490	13.2			
											0.198	(Fisher et al., 1990)								618-1420	12.2			(2)
							0.125	0.126	0.142	0.137		(Nemtchinov and Varanasi, 2004)					1,310	296	1013	1035-1340	11.4		H08	
							0.148	0.148	0.166	0.160		(Hurley-priv.com., 2003)					1,540	296	933	700-1500	12.4		G09	
							0.143	0.143	0.161	0.155		(M.S.F./R.A.L.)					1,490	296	1000	600-1600	12.5		G09	
											0.161	H2013				1,300								
							0.155	0.154	0.173	**0.167**		**This study (avg.)**					**1,600**							
1,1,1-Trifluoroethane	420-46-2	HFC-143a	CH_3CF_3	47.1	51.0	*WMO* (2019)	0.156	0.157	0.177	0.174		**(Le Bris and Graham, 2015)**					6,360	296		570-1500	13.8		H16	
											0.217	(H Zhang et al., 2011a)		S								(HITRAN, 2004)		
							0.155	0.155	0.175	0.173		**(Sharpe et al., 2004)**					6,300	298	1013	500-3000	13.9		H16	
							0.143	0.143	0.163	0.160	0.148	**(Sihra et al., 2001)**	A	O	LBL/NBM		5,850	296	933	700-1500	12.7			
							0.143	0.144	0.164	0.161		**(Di Lonardo and Masciarelli, 2000)**					5,880	293	800	797-1460	12.8		G09	
							0.133	0.134	0.152	0.150	0.130	(Highwood and Shine, 2000)	A	O	NBM		5,460	253		800-1470	12.0			
											0.160	(Jain et al., 2000)	A	M	NBM							(Pinnock et al., 1995)		
											0.141	(Naik et al., 2000)	A	M										
							0.153	0.153	0.174	0.172		**(Smith et al., 1998)**					6,260	297	1000	700-1500	13.6		H08	
							0.151	0.151	0.171	0.168	0.168	(Pinnock et al., 1995)	A		NBM		6,130	296	933	500-1500	12.3		G09	
												(Olliff and Fischer, 1994)						293		796-1474	12.8			
											0.166	(Fisher et al., 1990)								555-1535	12.7			(2)
											0.158	H2013				4,800								
							0.150	0.150	0.171	**0.168**		**This study (avg.)**					**6,130**							
1,1-Difluoroethane	75-37-6	HFC-152a	CH_3CHF_2	1.5	1.6	*WMO* (2019)					0.132	(H Zhang et al., 2011a)		S								(HITRAN, 2004)		
							0.116	0.115	0.129	0.105		**(Sharpe et al., 2004)**					178	298	1013	525-3000	8.0		H16	
							0.110	0.109	0.123	0.100	0.095	**(Sihra et al., 2001)**	A	O	LBL/NBM		170	296	933	700-1500	6.8			
							0.108	0.107	0.120	0.098	0.090	(Highwood and Shine, 2000)	A	O	NBM		166	253		830-1500	6.7			
											0.097	(Jain et al., 2000)	A	M	NBM							(Pinnock et al., 1995)		
											0.109	(Naik et al., 2000)	A	M										
							0.112	0.110	0.124	0.101		**(Vander Auwera, 2000)**					172	293	800	840-1490	6.9		G09	
							0.111	0.110	0.124	0.101	0.133	(Pinnock et al., 1995)	A		NBM		171	296	933	700-1400	7.4		G09	
												(Olliff and Fischer, 1994)						295		450-3500	6.5			
							0.111	0.110	0.124	0.101		**(Clerbaux et al., 1993)**					171	287		840-1490	6.9		H08	
												(Cappellani and Restelli, 1992)						293	1000	830-1485	6.9			
											0.135	(Fisher et al., 1990)								775-1420	6.1			(2)
											0.098	H2013				138								
							0.112	0.111	0.125	**0.102**		**This study (avg.)**					**172**							

Table S3 (cont.)

				Lifetime (yr)			RE (W m^{-2} ppb^{-1}) – This study				RE (W m^{-2} ppb^{-1}) – Literature					GWP 100-yr		Absorption cross-sections						
Name	CASRN	Identifier	Formula	H2013	New	Reference	H2013 inst. RE	New inst. RE	New RE – const. profile	New RE – lifetime corr.	Value	Reference	Instantaneous/Adjusted	Vertical correction	RE calculation	H2013	New – lifetime corr.	*T* (K)	*p* (hPa)	Waveno. range (cm^{-1})	Int. abs. cross-section (10^{-17} cm^2 molecule^{-1} cm^{-1})	Reference	Database	Notes
1,1,1,2,3,3,3-Heptafluoropropane	431-89-0	HFC-227ea	CF_3CHFCF_3	38.9	36.0	*WMO* (2019)	0.238	0.239	0.269	0.264	0.257	**(Gohar et al., 2004)**	A	S			3,660	296		400-1550	23.3			(1)
							0.256	0.256	0.288	0.283		**(Sharpe et al., 2004)**					3,930	298	1013	500-3000	25.3		H16	
							0.233	0.234	0.264	0.259	0.256	(Sihra et al., 2001)	A	O	LBL/NBM		3,600	296	933	700-1500	22.3			
											0.322	(Jain et al., 2000)	A	M	NBM							(Pinnock et al., 1995)		
											0.288	(Naik et al., 2000)	A	M										
											0.281	(Pinnock et al., 1995)	A		NBM			296		700-1400	23.0			
											0.258	H2013				3,350								
							0.247	0.247	0.278	**0.273**		**This study (avg.)**					**3,800**							
1,1,1,3,3,3-Hexafluoropropane	690-39-1	HFC-236fa	$CF_3CH_2CF_3$	242.0	213.0	*WMO* (2019)	0.223	0.223	0.253	0.251	0.251	**(Sihra et al., 2001)**	A	O	LBL/NBM		9,210	296	933	700-1500	22.8			
											0.264	(Jain et al., 2000)	A	M	NBM							(Pinnock et al., 1995)		
											0.233	(Naik et al., 2000)	A	M										
											0.290	(Gierczak et al., 1996)	A		LBL			296	1013	600-1350	22.6			(4)
											0.272	(Pinnock et al., 1995)	A		NBM			296		700-1400	23.3			
											0.243	H2013				8,060								
							0.223	0.223	0.253	**0.251**		**This study (avg.)**					**9,210**							
1,1,1,3,3-Pentafluoropropane	460-73-1	HFC-245fa	$CHF_2CH_2CF_3$	7.7	7.9	*WMO* (2019)	0.238	0.237	0.263	0.249	0.289	**(Orkin et al., 2003)**	I	NC			1,030	295		455-1485	19.8			(2)
							0.229	0.228	0.255	0.241	0.241	**(Sihra et al., 2001)**	A	O	LBL/NBM		994	296	933	640-1500	19.6			
											0.261	(Jain et al., 2000)	A	M	NBM									
											0.279	(Naik et al., 2000)	A	M										
											0.280	(Ko et al., 1999)	A		NBM					450-2325	20.6			
											0.243	H2013				858								
							0.234	0.233	0.259	**0.245**		**This study (avg.)**					**1,010**							
1,1,1,3,3-Pentafluorobutane	406-58-6	HFC-365mfc	$CH_3CF_2CH_2CF_3$	8.7	8.9	*WMO* (2019)	0.213	0.213	0.240	0.228	0.230	**(Inoue et al., 2008)**	I	NC	Pinnock		959	296	933	665-1500	18.8			
											0.212	(Naik et al., 2000)	A	M										
											0.209	(Barry et al., 1997)	I		NBM			298	1013	600-1500				
											0.223	H2013				804								
							0.213	0.213	0.240	**0.228**		**This study (avg.)**					**959**							
1,1,1,2,2,3,4,5,5,5-Decafluoropentane	138495-42-8	HFC-43-10mee	$CF_3CHFCHFCF_2CF_3$	16.1	17.0	*WMO* (2019)	0.329	0.330	0.369	0.358	0.359	**(Le Bris et al., 2018)**	A	H	H2013		1,680	305		550-1600	30.1			
							0.329	0.330	0.368	0.357		**(Sharpe et al., 2004)**					1,680	298	1013	500-3000	30.4		H16	
											0.420	H2013				1,650								
							0.329	0.330	0.369	**0.357**		**This study (avg.)**					**1,680**							

(1) Avg. of Reading and Oslo models; (2) Scaled to the CFC-11 RE of 0.26 Wm^{-2} ppb^{-1}; (3) Average of four models; (4) A factor 0.8 was applied to obtain cloudy-sky adjusted RE from inst. clear-sky RE

Table S4. Most Abundant Chlorocarbons and Hydrochlorocarbons

Please refer to page 2 of this document for a description of the table.

				Lifetime (yr)			RE (W m^{-2} ppb^{-1}) – This study				RE (W m^{-2} ppb^{-1}) – Literature					GWP 100-yr		Absorption cross-sections						
Name	CASRN	Identifier	Formula	H2013	New	Reference	H2013 inst. RE	New inst. RE	New RE – const. profile	New RE – lifetime corr.	Value	Reference	Instantaneous/Adjusted	Vertical correction	RE calculation	H2013	New – lifetime corr.	*T* (K)	*p* (hPa)	Waveno. range (cm^{-1})	Int. abs. cross-section (10^{-17} cm^2 molecule^{-1} cm^{-1})	Reference	Database	Notes
1,1,1-Trichloroethane	71-55-6	Methyl chloroform	CH_3CCl_3	5.0	5.0	*WMO* (2019)	0.070	0.069	0.071	0.065		**(Sharpe et al., 2004)**					172	298	1013	500-3000	5.3		H16	
							0.069	0.068	0.070	0.065	0.099	**(Orkin et al., 2003)**	I	NC			169	295		480-1490	5.1			(1)
											0.065	(Jain et al., 2000)	A	M	NBM							(Fisher et al., 1990)		
							0.068	0.067	0.069	0.064	0.059	**(Imasu et al., 1995)**	I	O			167	296		700-1500	5.0			(1)
											0.062	(Fisher et al., 1990)								669-1397	4.5			(1)
											0.069	H2013				160								
							0.069	0.068	0.070	**0.065**		**This study (avg.)**					**169**							
Tetrachloromethane	56-23-5	Carbon tetrachloride	CCl_4	26.0	32.0	*WMO* (2019)	0.177	0.174	0.189	0.178		(Harrison et al., 2017)					2,480	296	1013	700-860	6.7		H16	
							0.165	0.162	0.176	0.166	0.174	**(Wallington et al., 2016)**	A	H	H2013		2,310			730-825	6.3			
												(Sharpe et al., 2004)						298		730-825	6.4		P	
							0.165	0.161	0.176	0.165		(Nemtchinov and Varanasi, 2003)					2,300	297	1010	750-812	6.2		G09	
											0.125	(Jain et al., 2000)	A	M	NBM							(Fisher et al., 1990)		
							0.165	0.161	0.176	0.166		(Varanasi-priv.com., 2000)					2,300	297	1010	750-812	6.2		H08	
												(Chu et al., 1999)								730-825	6.7			
											0.091	(Myhre and Stordal, 1997)	A		BBM							(HITRAN, 1992)		
												(Orlando et al., 1992)						298		730-825	5.9			
											0.125	(Fisher et al., 1990)								616-934	4.5			(1)
												(Brown et al., 1987)						298		786-806	3.6			
												(Zander et al., 1987)								773-802	3.7			
												(Massie et al., 1985)						298		786-806	3.6			
												(Tanabe and Saeki, 1970)								730-825	5.3			
												(Lindsay and Schatz, 1964)								730-825	6.5			
											0.170	H2013				1,730								
							0.165	0.162	0.176	**0.166**		**This study (avg.)**					**2,310**							
Chloromethane	74-87-3	Methyl chloride	CH_3Cl	1.0	0.9	*WMO* (2019)	0.006	0.006	0.006	0.005	0.004	**(Wallington et al., 2016)**	A	H	H2013		6			660-1620	0.8			
							0.007	0.007	0.007	0.005		(Sharpe et al., 2004)					6	296		600-3000	1.3		P	
											0.005	(Grossman et al., 1997)	I	NC	k-dist					661-1646	1.4			
												(Brown et al., 1987)								697-1377	0.4			
												(Elkins et al., 1984)						296		660-1610	0.7			
												(Dickson et al., 1957)								660-1610	0.7			
												(Barrow and McKean, 1952)								660-1610	0.7			
											0.010	H2013				12								
							0.006	0.006	0.006	**0.005**		**This study (avg.)**					**6**							
Dichloromethane	75-09-2	Methylene chloride	CH_2Cl_2	0.4	0.5	*WMO* (2019)	0.046	0.046	0.048	0.029	0.028	**(Wallington et al., 2016)**	A	H	H2013		12			650-1290	2.6			
							0.048	0.047	0.050	0.030		(Sharpe et al., 2004)					12	298	1013	600-3000	2.8		H16	
												(Chu et al., 1999)								650-3075	3.0			
											0.031	H2013				9								
							0.046	0.046	0.048	**0.029**		**This study (avg.)**					**12**							
Trichloromethane	67-66-3	Chloroform	$CHCl_3$	0.4	0.5	*WMO* (2019)	0.114	0.112	0.122	0.074	0.070	**(Wallington et al., 2016)**	A	H	H2013		22			720-1245	4.4			
							0.123	0.121	0.131	0.079		(Sharpe et al., 2004)					23	298	1013	580-3000	5.0		H16	
											0.094	(Sihra et al., 2001)	A	S	LBL/NBM							(McPheat and Duxbury, 2000)		
											0.110	(Highwood and Shine, 2000)	A	O	NBM			295			5.0	(McPheat and Duxbury, 2000)		
							0.127	0.124	0.135	0.082		(McPheat and Duxbury, 2000)					24	295	800	540-1600	5.6			
												(Chu et al., 1999)								650-1255	5.1			
												(Kim and King, 1984)								650-1255	4.2			
												(Tanabe and Saeki, 1970)								650-1255	4.3			
											0.078	H2013				16								
							0.114	0.112	0.122	**0.074**		**This study (avg.)**					**22**							

(1) Scaled to the CFC-11 RE of 0.26 Wm^{-2} ppb^{-1}

Table S5. Most Abundant Bromocarbons, Hydrobromocarbons and Halons

Please refer to page 2 of this document for a description of the table.

				Lifetime (yr)			RE (W m^{-2} ppb^{-1}) – This study				RE (W m^{-2} ppb^{-1}) – Literature					GWP 100-yr		Absorption cross-sections						
Name	CASRN	Identifier	Formula	H2013	New	Reference	H2013 inst. RE	New inst. RE	New RE – const. profile	New RE – lifetime corr.	Value	Reference	Instantaneous/Adjusted	Vertical correction	RE calculation	H2013	New – lifetime corr.	*T* (K)	*p* (hPa)	Waveno. range (cm^{-1})	Int. abs. cross-section (10^{-17} cm^2 molecule^{-1} cm^{-1})	Reference	Database	Notes
Bromomethane	74-83-9	Methyl bromide	CH_3Br	0.8	0.8	*WMO* (2019)	0.006	0.006	0.006	0.004		**(Sharpe et al., 2004)**					3	296		550-3000	1.1		P	
							0.006	0.006	0.006	0.004	0.005	**(Sihra et al., 2001)**	A	S	LBL/NBM		2	296	933	450-2000	0.7			
											0.007	(Jain et al., 2000)	A	NC	NBM							(Christidis et al., 1997)		
											0.007	(Christidis et al., 1997)	A	NC	NBM			296	921	450-2000	0.8			
											0.005	(Grossman et al., 1997)	I	NC	k-dist					550-1600	1.2			
												(Graner, 1981)												
											0.005	H2013				2								
							0.006	0.006	0.006	**0.004**		**This study (avg.)**					**3**							
Bromochlorodifluoromethane	353-59-3	Halon-1211	$CBrClF_2$	16.0	16.0	*WMO* (2019)	0.298	0.293	0.327	0.307		**(Sharpe et al., 2004)**					2,070	298	1013	600-3000	13.2		H16	
							0.285	0.280	0.313	0.293	0.263	**(Sihra et al., 2001)**	A	O	LBL/NBM		1,980	296	933	450-2000	12.4			
											0.251	(Jain et al., 2000)	A	M	NBM							(Christidis et al., 1997)		
											0.334	(Christidis et al., 1997)	A	NC	NBM			296	921	450-2000	11.5			
											0.294	H2013				1,750								
							0.292	0.286	0.320	**0.300**		**This study (avg.)**					**2,030**							
Bromotrifluoromethane	75-63-8	Halon-1301	$CBrF_3$	65.0	72.0	*WMO* (2019)	0.280	0.279	0.313	0.299	0.300	**(Charmet et al., 2008)**	I	NC	Pinnock		7,600	298		461-2500	16.7			
											0.310	(Drage et al., 2006)	I	NC	Pinnock			298		720-1250	15.3			
							0.270	0.269	0.301	0.288		**(Sharpe et al., 2004)**					7,320	298	1013	510-3000	16.1		H16	
							0.281	0.279	0.314	0.300	0.296	**(Orkin et al., 2003)**	I	NC			7,620	295		720-1250	16.4			(1)
							0.291	0.289	0.324	0.310	0.289	**(Sihra et al., 2001)**	A	O	LBL/NBM		7,880	296	933	450-2000	17.0			
											0.273	(Jain et al., 2000)	A	M	NBM							(Person and Polo, 1961)		
												(Varanasi and Chudamani, 1988b)								1040-1250	16.0			
											0.333	(Ramanathan et al., 1985)								1040-1250	16.1			(1)
												(Person and Polo, 1961)								720-1250	15.9			
											0.298	H2013				6,290								
							0.281	0.279	0.313	**0.299**		**This study (avg.)**					**7,600**							
1,2-Dibromo-1,1,2,2-tetrafluoroethane	124-73-2	Halon-2402	$CBrF_2CBrF_2$	20.0	28.0	*WMO* (2019)	0.310	0.305	0.334	0.314		**(Sharpe et al., 2004)**					2,300	298	1013	550-3000	16.1		H16	
							0.305	0.301	0.329	0.309	0.329	**(Sihra et al., 2001)**	A	NC	LBL/NBM		2,270	296	933	450-2000	15.4			
											0.313	H2013				1,470								
							0.307	0.303	0.332	**0.312**		**This study (avg.)**					**2,280**							

(1) Scaled to the CFC-11 RE of 0.26 Wm^{-2} ppb^{-1}

Table S6. Most Abundant Fully Fluorinated Species

Please refer to page 2 of this document for a description of the table.

				Lifetime (yr)			RE (W m^{-2} ppb^{-1}) – This study				RE (W m^{-2} ppb^{-1}) – Literature					GWP 100-yr		Absorption cross-sections						
Name	CASRN	Identifier	Formula	H2013	New	Reference	H2013 inst. RE	New inst. RE	New RE – const. profile	New RE – lifetime corr.	Value	Reference	Instantaneous/Adjusted	Vertical correction	RE calculation	H2013	New – lifetime corr.	*T* (K)	*p* (hPa)	Waveno. range (cm^{-1})	Int. abs. cross-section (10^{-17} cm^2 $molecule^{-1}$ cm^{-1})	Reference	Database	Notes
Nitrogen trifluoride	7783-54-2		NF_3	500.0	569.0	*WMO* (2019)	0.197	0.191	0.212	0.207	0.250	**(Totterdill et al., 2016)**	A	M	LBL		18,700	296		600-1970	7.3			
							0.190	0.185	0.205	0.200	0.211	**(Robson et al., 2006)**	A				18,100	296		400-2000	7.0			
							0.195	0.189	0.210	0.206		**(Sharpe et al., 2004)**					18,600	298	1013	600-3000	7.2		H16	
												(Molina et al., 1995)								600-1970	4.3			
											0.205	H2013				16,100								
							0.194	0.188	0.209	**0.204**		**This study (avg.)**					**18,500**							
Sulfur hexafluoride	2551-62-4		SF_6	3200.0	3200.0	*WMO* (2019)					0.590	(Kovács et al., 2017)	A		LBL			296		650-2000	24.0			
											0.680	(H Zhang et al., 2011b)										(HITRAN, 2004)		
												(Vlachogiannis et al., 2005)												
							0.533	0.518	0.580	0.572		**(Sharpe et al., 2004)**					27,000	298	1013	560-3000	21.2		H16	
							0.547	0.532	0.598	0.591		**(Hurley-priv.com., 2003)**					27,900	296	933	900-1000	20.7		G09	
											0.494	(Jain et al., 2000)	A	NC	NBM									
							0.497	0.483	0.543	0.537		**(Varanasi-priv.com., 2000)**					25,300	295	1013	925-955	18.8		H08	
											0.519	(Myhre and Stordal, 1997)	A		BBM							(HITRAN, 1992)		
							0.497	0.484	0.544	0.538		(Varanasi et al., 1994)					25,400	216	120	925-955	18.9		H08	
												(Ko et al., 1993)								640-1225	20.2			
												(Chapados, 1988)									18.0			
												(McDowell et al., 1986)									17.6			
												(Dunn et al., 1982)									17.6			
												(Brodbeck et al., 1980)									18.3			
												(Kim et al., 1980)									22.6			
												(Schatz and Hornig, 1953)									17.9			
											**0.567**	**H2013**				23,500								
							0.525	0.511	0.574	0.567		This study (avg.)					**26,700**							
Sulfuryl fluoride	2699-79-8		SO_2F_2	36.0	36.0	*WMO* (2019)	0.186	0.182	0.203	0.199	0.196	**(M P S Andersen et al., 2009)**	I	NC	Pinnock		4,610	296	933	805-1760	13.0			
												(Dillon et al., 2008)								500-1542	13.6			
											0.222	(Papadimitriou et al., 2008b)		NC	LBL			296		500-1542	14.0			
							0.212	0.207	0.227	0.222		**(Sharpe et al., 2004)**					5,150	298	1013	500-3000	14.0		H16	
											0.201	H2013				4,100								
							0.199	0.195	0.215	**0.211**		**This study (avg.)**					**4,880**							
Tetrafluoromethane	75-73-0	PFC-14	CF_4	50000.0	50000.0	*WMO* (2019)					0.098	(H Zhang et al., 2011b)										(HITRAN, 2004)		
											0.080	(Bravo et al., 2010b)	I	NC	Pinnock							(Hurley et al., 2005)		
							0.085	0.087	0.097	0.096	0.102	**(Hurley et al., 2005)**	A	O	NBM		7,650	296	933	1230-1300	19.0			
							0.090	0.092	0.102	0.101		**(Sharpe et al., 2004)**					8,050	298	1013	570-3000	19.8		H16	
							0.103	0.105	0.116	0.116		(Hurley-priv.com., 2003)					9,190	296	933	1230-1300	21.9		G09	
							0.101	0.104	0.114	0.114	0.116	(Sihra et al., 2001)	A	S	LBL/NBM		9,040	296	933	500-1400	21.9			
											0.089	(Jain et al., 2000)	A	NC	NBM							(McDaniel et al., 1991)		
							0.087	0.089	0.099	0.098		**(Varanasi-priv.com., 2000)**					7,790	296	844	1250-1290	19.1		H08	
											0.084	(Myhre and Stordal, 1997)	A		BBM							(HITRAN, 1992)		
											0.110	(Roehl et al., 1995)	A		BBM			300		590-1320	18.5			(1)
												(McDaniel et al., 1991)						293		1255-1310	17.2			
												(Varanasi and Chudamani, 1988b)								1255-1310	14.0			
												(Golden et al., 1978)								1255-1310	16.9			
											0.095	H2013				6,630								
							0.088	0.090	0.099	**0.099**		**This study (avg.)**					**7,830**							

Table S6 (cont.)

Name	CASRN	Identifier	Formula	Lifetime (yr)			RE (W m^{-2} ppb^{-1}) – This study				RE (W m^{-2} ppb^{-1}) – Literature					GWP 100-yr		Absorption cross-sections						Notes
				H2013	New	Reference	H2013 inst. RE	New inst. RE	New RE – const. profile	New RE – lifetime corr.	Value	Reference	Instantaneous/Adjusted	Vertical correction	RE calculation	H2013	New – lifetime corr.	*T* (K)	*p* (hPa)	Waveno. range (cm^{-1})	Int. abs. cross-section (10^{-17} cm^2 molecule^{-1})	Reference	Database	
Hexafluoroethane	76-16-4	PFC-116	C_2F_6	10000.0	10000.0	*WMO* (2019)					0.346	(H Zhang et al., 2011b)										(HITRAN, 2004)		
							0.241	0.243	0.274	0.271	0.260	**(Bravo et al., 2010b)**	I	NC	Pinnock		13,700	297		700-1400	23.0			
							0.238	0.240	0.270	0.267		**(Sharpe et al., 2004)**					13,500	298	1013	500-3000	23.1		H16	
							0.223	0.225	0.254	0.252		**(Zou et al., 2004)**					12,700	296	844	1061-1285	21.0		H08	
							0.226	0.228	0.256	0.254	0.266	**(Sihra et al., 2001)**	A	S	LBL/NBM		12,800	296	933	450-1500	21.6			
												(Ballard et al., 2000a)						293		675-1400	22.8			
							0.235	0.237	0.266	0.264	0.260	(Highwood and Shine, 2000)	A	O	NBM		13,300	253		680-1380	22.8			
							0.224	0.225	0.254	0.252		(Highwood et al., 1999)					12,700	296	933	690-1360	21.5		G09	
											0.252	(Myhre and Stordal, 1997)	A		BBM							(HITRAN, 1992)		
											0.250	(Roehl et al., 1995)	A		BBM			300		480-1385	21.6			(1)
												(Varanasi and Chudamani, 1988b)												
												(Mills et al., 1958)												
							0.231	0.232	0.262	0.260		(M.S.F./R.A.L.)					13,100	293	800	690-1360	22.2		G09	
											0.251	H2013				11,100								
							0.232	0.234	0.263	**0.261**		**This study (avg.)**					**13,200**							
Octafluoropropane	76-19-7	PFC-218	C_3F_8	2600.0	2600.0	*WMO* (2019)	0.251	0.250	0.270	0.266	0.270	**(Bravo et al., 2010b)**	I	NC	Pinnock		9,710	297		700-1400	26.8			
							0.253	0.253	0.272	0.269		**(Sharpe et al., 2004)**					9,810	298	1013	600-3000	27.5		H16	
							0.260	0.259	0.279	0.275	0.279	**(Sihra et al., 2001)**	A	S	LBL/NBM		10,000	296	933	500-1500	27.5			
											0.260	(Roehl et al., 1995)	A		BBM			300		500-1550	22.0			(1)
											0.277	H2013				8,900								
							0.255	0.254	0.274	**0.270**		**This study (avg.)**					**9,850**							
Octafluorocyclobutane	115-25-3	PFC-C-318	cyc (-$CF_2CF_2CF_2CF_2$-)	3200.0	3200.0	*WMO* (2019)					0.310	(Vasekova et al., 2006)	I	F	Pinnock			297		550-1500	21.7			
							0.281	0.279	0.314	0.310		**(Sharpe et al., 2004)**					10,700	298	1013	550-3000	21.7		H16	
											0.314	(Sihra et al., 2001)	A	S	LBL/NBM							(Highwood and Shine, 2000)		
												(Ballard et al., 2000a)						297		550-1500	22.2			
							0.290	0.287	0.322	0.318	0.320	**(Highwood and Shine, 2000)**	A	O	NBM		10,900	253		555-1460	21.6			
							0.295	0.292	0.327	0.323		(M.S.F./R.A.L.)					11,100	297		555-1430	21.8		G09	
											0.315	H2013				9,550								
							0.286	0.283	0.318	**0.314**		**This study (avg.)**					**10,800**							
Decafluorobutane	355-25-9	PFC-31-10	n-C_4F_{10}	2600.0	2600.0	*WMO* (2019)	0.334	0.334	0.372	0.367	0.370	**(Bravo et al., 2010b)**	I	NC	Pinnock		10,600	297		450-1500	31.7			
							0.337	0.337	0.375	0.370		**(Sharpe et al., 2004)**					10,700	298	1013	500-3000	32.4		H16	
							0.335	0.335	0.373	0.368	0.370	(Sihra et al., 2001)	A	S	LBL/NBM		10,600	296	933	450-1500	31.7			
											0.330	(Roehl et al., 1995)	A		BBM			300		500-1550	27.2			(1)
											0.363	H2013				9,200								
							0.336	0.336	0.374	**0.369**		**This study (avg.)**					**10,600**							
Dodecafluoropentane	678-26-2	PFC-41-12	n-C_5F_{12}	4100.0	4100.0	*WMO* (2019)	0.373	0.373	0.415	0.410	0.400	**(Bravo et al., 2010b)**	I	NC	Pinnock		9,840	297		210-2000	36.3			
							0.370	0.370	0.410	0.405		**(Sharpe et al., 2004)**					9,720	278	1013	500-3000	37.3		H16	
											0.410	(Roehl et al., 1995)	A		BBM			300		500-1550	34.6			(1)
											0.405	H2013				8,550								
							0.371	0.371	0.412	**0.408**		**This study (avg.)**					**9,780**							
Tetradecafluorohexane	355-42-0	PFC-51-14	n-C_6F_{14}	3100.0	3100.0	*WMO* (2019)	0.407	0.407	0.455	0.449	0.430	**(Bravo et al., 2010b)**	I	NC	Pinnock		9,140	297		210-2000	38.5			
											0.490	(Roehl et al., 1995)	A		BBM			300		500-1550	39.6			(1)
											0.442	H2013				7,910								
							0.407	0.407	0.455	**0.449**		**This study (avg.)**					**9,140**							
Hexadecafluoroheptane	335-57-9	PFC-61-16	n-C_7F_{16}	3000.0	3000.0	*WMO* (2019)	0.462	0.463	0.510	0.503	0.480	**(Ivy et al., 2012)**	I	NC	Pinnock		8,920	296		500-4000	44.7			
											0.502	H2013				7,820								
							0.462	0.463	0.510	**0.503**		**This study (avg.)**					**8,920**							
Octadecafluorooctane	307-34-6	PFC-71-18	n-C_8F_{18}	3000.0	3000.0	*WMO* (2019)	0.546	0.547	0.604	0.597	0.570	**(Ivy et al., 2012)**	I	NC	Pinnock		9,370	296		500-4000	52.3			
							0.470	0.471	0.526	0.519	0.500	**(Bravo et al., 2010b)**	I	NC	Pinnock		8,150	297		700-1400	45.6			
											0.552	H2013				7,620								
							0.508	0.509	0.565	**0.558**		**This study (avg.)**					**8,760**							

(1) Applied factor 1.11 to account for stratospheric adjustment

Table S7. Other Chlorofluorocarbons

Please refer to page 2 of this document for a description of the table.

Name	CASRN	Identifier	Formula	Lifetime (yr) H2013	Lifetime (yr) New	Lifetime (yr) Reference	RE ($W m^{-2} ppb^{-1}$) – This study: H2013 inst. RE	New inst. RE	New RE – const. profile	New RE – lifetime corr.	RE ($W m^{-2} ppb^{-1}$) – Literature: Value	Reference	Instantaneous/Adjusted	Vertical correction	RE calculation	GWP 100-yr: H2013	New – lifetime corr.	Absorption cross-sections: T (K)	p (hPa)	Waveno. range (cm^{-1})	Int. abs. cross-section (10^{-17} cm^2 $molecule^{-1}$)	Reference	Database	Notes
Chlorotrifluoromethane	75-72-9	CFC-13	$CClF_3$	640.0	640.0	*WMO* (2019)	0.266	0.264	0.300	0.294		**(Sharpe et al., 2004)**					18,200	296		600-3000	17.0		P	
											0.245	(Jain et al., 2000)	A	NC	NBM							(McDaniel et al., 1991)		
											0.252	(Myhre and Stordal, 1997)	A		BBM							(HITRAN, 1992)		
							0.237	0.235	0.267	0.261		(Massie et al., 1991)					16,200	293		765-1235	14.8		H16	
							0.237	0.235	0.267	0.261		**(McDaniel et al., 1991)**					16,200	293		765-1235	14.8		H08	
												(Varanasi and Chudamani, 1988b)								1050-1291	16.3			
												(Golden et al., 1978)								755-1291	16.1			
											0.255	H2013				13,900								
							0.251	0.250	0.284	**0.278**		**This study (avg.)**					**17,200**							
1,2-Difluoro-1,1,2,2-tetrachloroethane	76-12-0	CFC-112	CCl_2FCCl_2F		63.6	*WMO* (2019)	0.278	0.272	0.299	0.285	0.280	**(Davis et al., 2016)**			H2013		4,930	296		612-1225	11.2			
											0.290	(Etminan et al., 2014)	I	H	NBM			295		600-1730				
							0.273	0.267	0.292	0.279		**(Sharpe et al., 2004)**					4,820	298	1013	550-3000	11.3		H16	
												(Olliff and Fischer, 1994)						295		450-3500	10.1			
							0.276	0.270	0.295	**0.282**		**This study (avg.)**					**4,880**							
2,2-Difluoro-1,2,2,2-tetrachloroethane	76-11-9	CFC-112a	CCl_3CClF_2		52.0	*WMO* (2019)	0.246	0.241	0.259	0.246	0.250	**(Davis et al., 2016)**			H2013		3,750	296		610-1215	11.4			
							0.245	0.240	0.258	0.245		**(Sharpe et al., 2004)**					3,740	278	1013	570-3000	11.5		H16	
												(Olliff and Fischer, 1994)						295		450-3500	9.8			
							0.245	0.241	0.258	**0.246**		**This study (avg.)**					**3,740**							
1,1,1-Trichloro-2,2,2-trifluoroethane	354-58-5	CFC-113a	CCl_3CF_3		55.0	*WMO* (2019)	0.239	0.237	0.265	0.252	0.240	**(Davis et al., 2016)**			H2013		4,340	296		525-1290	14.7			
							0.217	0.215	0.241	0.230	0.230	**(Etminan et al., 2014)**	I	H	NBM		3,950	295		600-1730	13.5		H16	
												(Olliff and Fischer, 1994)						295		450-3500	11.8			
							0.228	0.226	0.253	**0.241**		**This study (avg.)**					**4,140**							
1,1-Dichloro-1,2,2,2-tetrafluoroethane	374-07-2	CFC-114a	CCl_2FCF_3		105.0	*WMO* (2019)	0.280	0.276	0.309	0.297	0.280	**(Davis et al., 2016)**			H2013		7,850	296		543-1355	16.9		H16	
												(Olliff and Fischer, 1994)						295		450-3500	14.2			
							0.280	0.276	0.309	**0.297**		**This study (avg.)**					**7,850**							
(E)-1,2-Dichlorohexafluorocyclobutane	3832-15-3	E-R316c	trans cyc (-$CClFCF_2CF_2CClF$-)		75.0	*WMO* (2019)	0.261	0.258	0.282	**0.270**	0.270	**(Papadimitriou et al., 2013)**			LBL		**4,470**	296		500-1600	16.8			
(Z)-1,2-Dichlorohexafluorocyclobutane	3934-26-7	Z-R316c	cis cyc (-$CClFCF_2CF_2CClF$-)		114.0	*WMO* (2019)	0.289	0.285	0.311	**0.300**	0.290	**(Papadimitriou et al., 2013)**			LBL		**5,990**	296		500-1600	19.2			
1,2-dichloro-1,2-difluoroethene	598-88-9	CFC 1112	$CClF{=}CClF$		7.1 days	(Barrera et al., 2015; Herath et al., 2016)					0.013	(Herath et al., 2016)			H2013			290						
											0.107	(Barrera et al., 2015)			Pinnock			298		500-1500	5.6			
											**0.013**	**This study**					**<1**							(1)
1,1-dichloro-2,2-difluoroethene	79-35-6	CFC 1112a	$CCl_2{=}CF_2$		2.3 days	(Barrera et al., 2015; Herath et al., 2016)					0.007	(Herath et al., 2016)			H2013			290						
											0.080	(Barrera et al., 2015)			Pinnock			298		500-1500	5.5			
											**0.007**	**This study**					**<1**							(1)
1,1,2-trichloro-2-fluoroethene	359-29-5		$CCl_2{=}CClF$				0.119	0.117	**0.130**			**(Sharpe et al., 2004)**						296		520-3000	5.9		P	
Chlorotrifluoroethylene	79-38-9		$CF_2{=}CClF$				0.110	0.109	**0.114**			**(Sharpe et al., 2004)**						296		530-3000	10.7		P	

(1) RE value is from (Herath et al., 2016) with lifetime correction included

Table S8. Other Hydrochlorofluorocarbons

Please refer to page 2 of this document for a description of the table.

				Lifetime (yr)			RE (W m^{-2} ppb^{-1}) – This study				RE (W m^{-2} ppb^{-1}) – Literature					GWP 100-yr		Absorption cross-sections						
Name	CASRN	Identifier	Formula	H2013	New	Reference	H2013 inst. RE	New inst. RE	New RE – const. profile	New RE – lifetime corr.	Value	Reference	Instantaneous/Adjusted	Vertical correction	RE calculation	H2013	New – lifetime corr.	T (K)	p (hPa)	Waveno. range (cm^{-1})	Int. abs. cross-section (10^{-17} cm^2 molecule^{-1})	Reference	Database	Notes
Dichlorofluoromethane	75-43-4	HCFC-21	$CHCl_2F$	1.7	1.7	*WMO* (2019)	0.170	0.167	0.181	0.149		**(Sharpe et al., 2004)**					172	298	1013	600-3000	7.9		H16	
							0.161	0.158	0.172	0.141	0.140	**(Sihra et al., 2001)**	A	S	LBL/NBM		163	296	933	600-1500	7.4			
											0.185	(Christidis et al., 1997)	A	NC	NBM			296	921	450-2000	6.8			
							0.078	0.077	0.084	0.069		(Massie et al., 1985)					80	296		785-840	2.7		H08	
											**0.145**	**H2013**				148								
							0.165	0.162	0.176	0.145		This study (avg.)					**168**							
Chlorofluoromethane	593-70-4	HCFC-31	CH_2ClF		1.2	*WMO* (2019)					0.080	(Charmet et al., 2013)	I		Pinnock			298		700-2850	4.2			
											**0.068**	**This study**					**83**							(1)
1,1,2,2-Tetrachloro-1-fluoroethane	354-14-3	HCFC-121	$CHCl_2CCl_2F$		1.1	*WMO* (2019)	0.184	0.180	0.193	**0.146**		**(Sharpe et al., 2004)**					**61**	298	1013	560-3000	8.0		H16	
1,1,2-Trichloro-2,2-difluoroethane	354-21-2	HCFC-122	$CHCl_2CClF_2$	1.0	0.9	*WMO* (2019)	0.208	0.204	0.220	0.159	0.229	**(Orkin et al., 2003)**	I	NC			59	295		560-1360	10.4			(2)
											0.168	H2013				**59**								
							0.208	0.204	0.220	**0.159**		**This study (avg.)**					59							
1,1,2-Trichloro-1,2-difluoroethane	354-15-4	HCFC-122a	$CHClFCCl_2F$	3.4	3.1	*WMO* (2019)	0.212	0.208	0.227	0.201	0.237	**(Orkin et al., 2003)**	I	NC			257	295		590-1380	9.9			(2)
											0.209	H2013				258								
							0.212	0.208	0.227	**0.201**		**This study (avg.)**					**257**							
2,2-Dichloro-1,1,1-trifluoroethane	306-83-2	HCFC-123	$CHCl_2CF_3$	1.3	1.3	*WMO* (2019)	0.185	0.184	0.207	0.162		**(Sharpe et al., 2004)**					96	298	1013	500-3000	13.5		H16	
							0.183	0.181	0.203	0.159	0.216	**(Orkin et al., 2003)**	I	NC			95	295		480-1430	13.1			(2)
							0.177	0.175	0.197	0.154	0.141	**(Sihra et al., 2001)**	A	O	LBL/NBM		92	296	933	700-1500	11.9			
											0.143	(Jain et al., 2000)	A	M	NBM							(Pinnock et al., 1995)		
											0.168	(Naik et al., 2000)	A	M										
							0.178	0.176	0.198	0.155	0.206	(Pinnock et al., 1995)	A		NBM		92	296	933	700-1400	12.0		G09	
												(Olliff and Fischer, 1994)						295		480-1430	12.7			
							0.185	0.184	0.207	0.162		**(Clerbaux et al., 1993)**					97	287		740-1450	12.9		H08	
												(Cappellani and Restelli, 1992)						293	1000	648-1440	12.7			
											0.208	(Fisher et al., 1990)								649-1307	10.6			(2)
											0.152	H2013				79								
							0.183	0.181	0.203	**0.160**		**This study (avg.)**					**95**							
1,2-Dichloro-1,1,2-trifluoroethane	354-23-4	HCFC-123a	$CHClFCClF_2$	4.0	4.0	*WMO* (2019)	0.234	0.230	0.252	0.228		**(Sharpe et al., 2004)**					418	298	1013	520-3000	12.8		H16	
							0.230	0.227	0.248	0.225	0.255	**(Orkin et al., 2003)**	I	NC			411	295		450-1400	12.3			(2)
											0.230	H2013				370								
							0.232	0.229	0.250	**0.227**		**This study (avg.)**					**415**							
2-Chloro-1,1,1,2-tetrafluoroethane	2837-89-0	HCFC-124	$CHClFCF_3$	5.9	5.9	*WMO* (2019)	0.210	0.208	0.234	0.218		**(Sharpe et al., 2004)**					660	298	1013	500-3000	15.4		H16	
							0.188	0.187	0.211	0.196	0.193	**(Sihra et al., 2001)**	A	O	LBL/NBM		594	296	933	700-1500	13.4			
											0.195	(Jain et al., 2000)	A	M	NBM							(Pinnock et al., 1995)		
											0.199	(Naik et al., 2000)	A	M										
							0.190	0.188	0.212	0.198	0.224	(Pinnock et al., 1995)	A		NBM		598	296	933	670-1435	13.8		G09	
												(Olliff and Fischer, 1994)						295		450-3500	13.6			
							0.198	0.197	0.222	0.207		**(Clerbaux et al., 1993)**					627	287		675-1430	14.4		H08	
											0.229	(Fisher et al., 1990)								440-1420	15.0			(2)
											0.198	H2013				527								
							0.199	0.197	0.222	**0.207**		**This study (avg.)**					**627**							
1-Chloro-1,1,2,2-tetrafluoroethane	354-25-6	HCFC-124a	CHF_2CClF_2		17.0	*WMO* (2019)	0.236	0.233	0.258	**0.250**		**(Sharpe et al., 2004)**					**2,170**	298	1013	525-3000	15.1		H16	
1,2-Dichloro-1,2-difluoroethane	431-06-1	HCFC-132	$CHClFCHClF$		1.7	*WMO* (2019)	0.163	0.160	0.174	**0.143**		**(Sharpe et al., 2004)**					**128**	278	1013	550-3000	8.1		H16	
1,1-Dichloro-2,2-difluoroethane	471-43-2	HCFC-132a	$CHCl_2CHF_2$		1.1	*WMO* (2019)	0.154	0.151	0.167	**0.127**		**(Sharpe et al., 2004)**					**74**	278	1013	510-3000	8.4		H16	
1,1-Dichloro-1,2-difluoroethane	1842-05-3	HCFC-132c	CH_2FCCl_2F	4.3	4.1	*WMO* (2019)	0.172	0.169	0.186	0.169	0.192	**(Orkin et al., 2003)**	I	NC			359	295		425-1490	8.4			(2)
											0.173	H2013				338								
							0.172	0.169	0.186	**0.169**		**This study (avg.)**					**359**							
2-Chloro-1,1,1-trifluoroethane	75-88-7	HCFC-133a	CH_2ClCF_3		4.6	*WMO* (2019)	0.150	0.150	0.168	0.154	0.160	**(McGillen et al., 2015)**			H2013		418	296		780-1530	12.5			
							0.141	0.140	0.158	0.145	0.150	**(Etminan et al., 2014)**	I	H	NBM		393	295		600-1730	12.0		H16	
							0.148	0.147	0.165	0.151		**(Sharpe et al., 2004)**					411	298	1013	500-3000	13.2		H16	
							0.146	0.146	0.164	**0.150**		**This study (avg.)**					**407**							
1,2-Dichloro-1-difluoroethane	430-57-9	HCFC-141	$CH_2ClCHClF$		1.1	*WMO* (2019)	0.091	0.090	0.094	**0.072**		**(Sharpe et al., 2004)**					**49**	298	1013	550-3000	5.5		H16	

Table S8 (cont.)

				Lifetime (yr)			RE (W m^{-2} ppb^{-1}) – This study				RE (W m^{-2} ppb^{-1}) – Literature					GWP 100-yr		Absorption cross-sections						
Name	CASRN	Identifier	Formula	H2013	New	Reference	H2013 inst. RE	New inst. RE	New RE – const. profile	New RE – lifetime corr.	Value	Reference	Instantaneous/Adjusted	Vertical correction	RE calculation	H2013	New – lifetime corr.	T (K)	p (hPa)	Waveno. range (cm^{-1})	Int. abs. cross-section (10^{-17} cm^2 $molecule^{-1}$)	Reference	Database	Notes
3,3-Dichloro-1,1,1,2,2-pentafluoropropane	422-56-0	HCFC-225ca	$CHCl_2CF_2CF_3$	1.9	1.9	*WMO* (2019)	0.245	0.244	0.267	0.223	0.202	**(Sihra et al., 2001)**	A	O	LBL/NBM		146	296	933	700-1400	18.0			
											0.207	(Jain et al., 2000)	A	M	NBM							(Pinnock et al., 1995)		
											0.208	(Naik et al., 2000)	A	M										
											0.268	(Pinnock et al., 1995)	A		NBM			296		700-1400	14.6			
												(Olliff and Fischer, 1994)						295		450-3500	16.3			
							0.236	0.235	0.257	0.215		**(Clerbaux et al., 1993)**					141	287		695-1420	17.7		H08	
											0.221	H2013				127								
							0.241	0.240	0.262	**0.219**		**This study (avg.)**					**143**							
1,3-Dichloro-1,1,2,2,3-pentafluoropropane	507-55-1	HCFC-225cb	$CHClFCF_2CClF_2$	5.9	5.9	*WMO* (2019)	0.275	0.271	0.302	0.281	0.280	**(Sihra et al., 2001)**	A	O	LBL/NBM		571	296	933	700-1400	15.2			
											0.245	(Jain et al., 2000)	A	M	NBM							(Pinnock et al., 1995)		
											0.259	(Naik et al., 2000)	A	M										
							0.301	0.297	0.330	0.308	0.309	**(Imasu et al., 1995)**	I	O			626	296		700-1500	16.6			(2)
											0.347	(Pinnock et al., 1995)	A		NBM			296		700-1400	15.1			
												(Olliff and Fischer, 1994)						295		450-3500	15.6			
							0.283	0.280	0.311	0.290		**(Clerbaux et al., 1993)**					590	287		715-1375	15.6		H08	
											**0.293**	**H2013**				525								
							0.286	0.283	0.314	0.293		This study (avg.)					**596**							
(E)-1-Chloro-3,3,3-trifluoroprop-1-ene	102687-65-0	HCFO-1233zd(E)	(E)-CF_3CH=CHCl	26.0 days	42.5 days	*WMO* (2019)	0.211	0.209	0.235	0.067	0.055	**(Gierczak et al., 2014)**		H	H2013		4	296		600-1800	18.6			
												(Orkin et al., 2014a)						298		600-1800	18.1			
							0.200	0.199	0.224	0.064	0.214	**(M P S Andersen et al., 2008)**	I	NC	Pinnock		4	295	933	600-1800	17.4			
											0.044	H2013				1								
							0.206	0.204	0.229	**0.065**		**This study (avg.)**					**4**							
(Z)-1-Chloro-3,3,3-trifluoroprop-1-ene	99728-16-2	HCFO-1233zd(Z)	(Z)-CF_3CH=CHCl		13.0 days	*WMO* (2019)					0.023	(L L Andersen et al., 2015)		H	H2013			296		600-2000	14.8			
							0.191	0.190	0.213	0.025	0.020	**(Gierczak et al., 2014)**		H	H2013		<1	296		450-1800	16.3			
							0.191	0.190	0.213	**0.025**		**This study**					**<1**							
(E/Z)-1-chloro-2-fluoro-ethene	460-16-2		(E/Z)-CHCl=CHF		1.8 days	(Barrera et al., 2015)					0.040	(Barrera et al., 2015)		NC	Pinnock			298		500-1500	2.8			
											**0.001**	**This study**					**<1**							(3)

(1) RE value is from (Charmet et al., 2013) except that stratospheric temperature adjustment (10% increase of inst. RE) and lifetime correction factors have been applied; (2) Scaled to the CFC-11 RE of 0.26 W m^{-2} ppb^{-1}; (3) RE value is from (Barrera et al., 2015) except that stratospheric temperature adjustment (10% increase of inst. RE) and lifetime correction factors have been applied

Table S9. Other Hydrofluorocarbons

Please refer to page 2 of this document for a description of the table.

Name	CASRN	Identifier	Formula	Lifetime (yr) H2013	Lifetime (yr) New	Lifetime (yr) Reference	RE ($W\ m^{-2}\ ppb^{-1}$) – This study: H2013 inst. RE	New inst. RE	New RE – const. profile	New RE – lifetime corr.	RE ($W\ m^{-2}\ ppb^{-1}$) – Literature: Value	Reference	Instantaneous/Adjusted	Vertical correction	RE calculation	GWP 100-yr: H2013	GWP 100-yr: New – lifetime corr.	Absorption cross-sections: T (K)	p (hPa)	Waveno. range (cm^{-1})	Int. abs. cross-section ($10^{-17}\ cm^2\ molecule^{-1}$)	Reference	Database	Notes
Fluoromethane	593-53-3	HFC-41	CH_3F	2.8	2.8	*WMO* (2019)	0.032	0.032	0.030	0.027		**(Sharpe et al., 2004)**					154	298	1013	600-3000	2.9		H16	
							0.028	0.027	0.026	0.023	0.023	**(Sihra et al., 2001)**	A	S	LBL/NBM		130	296	933	700-1400	1.5			
											0.030	(Pinnock et al., 1995)	A		NBM			296		700-1400	1.8			
											0.023	H2013				116								
							0.030	0.029	0.028	**0.025**		**This study (avg.)**					**142**							
1,1,2,2-Tetrafluoroethane	359-35-3	HFC-134	CHF_2CHF_2	9.7	10.0	*WMO* (2019)					0.272	(H Zhang et al., 2011a)		S								(HITRAN, 2004)		
							0.182	0.181	0.203	0.194		**(Sharpe et al., 2004)**					1,330	298	1013	570-3000	11.5		H16	
							0.176	0.175	0.196	0.187		(Hurley-priv.com., 2003)					1,280	296	933	450-1420	10.5		G09	
							0.176	0.175	0.196	0.187	0.180	**(Sihra et al., 2001)**	A	O	LBL/NBM		1,280	296	933	450-1550	10.5			
											0.176	(Jain et al., 2000)	A	M	NBM							(Christidis et al., 1997)		
											0.181	(Naik et al., 2000)	A	M										
							0.188	0.187	0.210	0.200		**(Smith et al., 1998)**					1,370	297	1001	600-1700	11.4		H08	
											0.206	(Christidis et al., 1997)	A	NC	NBM			296	921	450-2000	9.7			
												(Olliff and Fischer, 1994)						295		450-3500	10.4			
							0.189	0.187	0.210	0.201		(M.S.F./R.A.L.)					1,380	297	1000	600-1700	11.4		G09	
											0.191	H2013				1,120								
							0.182	0.181	0.203	**0.194**		**This study (avg.)**					**1,330**							
1,1,2-Trifluoroethane	430-66-0	HFC-143	CH_2FCHF_2	3.5	3.6	*WMO* (2019)	0.125	0.124	0.138	0.124	0.118	**(Sihra et al., 2001)**	A	O	LBL/NBM		371	296	933	700-1550	7.1			
											0.115	(Jain et al., 2000)	A	M	NBM							(Pinnock et al., 1995)		
											0.112	(Naik et al., 2000)	A	M										
											0.145	(Pinnock et al., 1995)	A		NBM			296		700-1400	6.9			
												(Olliff and Fischer, 1994)						295		450-3500	7.6			
											0.130	(Clerbaux and Colin)						287		700-1500	6.9			
							0.134	0.133	0.146	0.131		**(M.S.F./R.A.L.)**					394	296	933	400-1500	7.6		G09	
											**0.128**	**H2013**				328								
							0.130	0.128	0.142	0.128		This study (avg.)					**382**							
1,2-Difluoroethane	624-72-6	HFC-152	CH_2FCH_2F	0.4	0.5	*WMO* (2019)	0.072	0.071	0.077	0.045		**(Wallington et al., 1994)**					23			810-1510	3.7			
											0.044	H2013				16								
							0.072	0.071	0.077	**0.045**		**This study (avg.)**					**23**							
Fluoroethane	353-36-6	HFC-161	CH_3CH_2F	66.0 days	80.0 days	*WMO* (2019)	0.038	0.038	0.038	0.016	0.024	**(Sihra et al., 2001)**	A	O	LBL/NBM		5	296	933	450-1600	2.4			
											0.022	(Jain et al., 2000)	A	M	NBM							(Christidis et al., 1997)		
											0.024	(Naik et al., 2000)	A	M										
											0.040	(Christidis et al., 1997)	A	NC	NBM			296	921	450-2000	2.2			
												(Olliff and Fischer, 1994)						295		450-3500	4.0			
											**0.016**	**H2013**				4								
							0.038	0.038	0.038	0.016		This study (avg.)					**5**							
1,1,1,2,2,3,3-Heptafluoropropane	2252-84-8	HFC-227ca	$CF_3CF_2CHF_2$	28.2	30.0	*WMO* (2019)	0.248	0.248	0.269	0.264	0.249	**(Sihra et al., 2001)**	A	S	LBL/NBM		3,140	296	933	450-1550	19.9			
											0.330	(Christidis et al., 1997)	A	NC	NBM			296	921	450-2000	20.9			
											0.267	H2013				2,640								
							0.248	0.248	0.269	**0.264**		**This study (avg.)**					**3,140**							
1,1,1,2,2,3-Hexafluoropropane	677-56-5	HFC-236cb	$CH_2FCF_2CF_3$	13.1	13.4	*WMO* (2019)	0.215	0.215	0.240	0.231	0.217	**(Sihra et al., 2001)**	A	S	LBL/NBM		1,420	296	933	450-1550	17.0			
											0.258	(Christidis et al., 1997)	A	NC	NBM			296	921	450-2000	16.5			
											0.228	H2013				1,210								
							0.215	0.215	0.240	**0.231**		**This study (avg.)**					**1,420**							
1,1,1,2,3,3-Hexafluoropropane	431-63-0	HFC-236ea	CHF_2CHFCF_3	11.0	11.4	*WMO* (2019)					0.300	(Gierczak et al., 1996)	A		LBL			296	1013	680-1320	17.2			(1)
											**0.300**	**H2013**				1,340								
											0.300	This study					**1,570**							(2)
1,1,2,2,3-Pentafluoropropane	679-86-7	HFC-245ca	$CH_2FCF_2CHF_2$	6.5	6.6	*WMO* (2019)					**0.240**	**H2013**				716								
											0.240	This study					**827**							(2)

Table S9 (cont.)

				Lifetime (yr)			RE (W m^{-2} ppb^{-1}) – This study				RE (W m^{-2} ppb^{-1}) – Literature					GWP 100-yr		Absorption cross-sections						
Name	CASRN	Identifier	Formula	H2013	New	Reference	H2013 inst. RE	New inst. RE	New RE – const. profile	New RE – lifetime corr.	Value	Reference	Instantaneous/Adjusted	Vertical correction	RE calculation	H2013	New – lifetime corr.	*T* (K)	*p* (hPa)	Waveno. range (cm^{-1})	Int. abs. cross-section (10^{-17} cm^2 molecule^{-1})	Reference	Database	Notes
1,1,1,2,2-Pentafluoropropane	1814-88-6	HFC-245cb	$CF_3CF_2CH_3$	47.1	39.9	*WMO* (2019)	0.234	0.234	0.267	0.262	0.281	**(Orkin et al., 2003)**	I	NC			5,010	295		472-1490	18.1			(3)
							0.214	0.214	0.244	0.239	0.245	**(Sihra et al., 2001)**	A	NC	LBL/NBM		4,570	296	933	450-1550	16.5			
											0.274	(Christidis et al., 1997)	A	NC	NBM			296	921	450-2000	15.8			
											0.243	H2013				4,620								
							0.224	0.224	0.255	**0.251**		**This study (avg.)**					**4,790**							
1,1,2,3,3-Pentafluoropropane	24270-66-4	HFC-245ea	$CHF_2CHFCHF_2$	3.2	3.2	*WMO* (2019)					0.180	(Rajakumar et al., 2006)	A	NC	LBL			298		500-4000				
											**0.160**	**H2013**				235								
											0.160	This study					**267**							(2)
1,1,1,2,3-Pentafluoropropane	431-31-2	HFC-245eb	$CH_2FCHFCF_3$	3.1	3.2	*WMO* (2019)					0.230	(Rajakumar et al., 2006)	A	NC	LBL			298		500-4000				
											**0.204**	**H2013**				290								
											0.204	This study					**341**							(2)
1,1,1-Trifluoropropane	421-07-8	HFC-263fb	$CH_3CH_2CF_3$	1.2	1.1	*WMO* (2019)					0.130	(Rajakumar et al., 2006)	A	NC	LBL			298		500-4000				
											**0.100**	**H2013**				76								
											0.100	This study					**78**							(2)
2,2-Difluoropropane	420-45-1	HFC-272ca	$CH_3CF_2CH_3$	2.6	9.0	*WMO* (2019)	0.075	0.075	0.084	0.080	0.082	**(Sihra et al., 2001)**	A	NC	LBL/NBM		629	296	933	700-1500	5.7			
											0.090	(Pinnock et al., 1995)	A					296		700-1400	5.6			
											0.072	H2013				144								
							0.075	0.075	0.084	**0.080**		**This study (avg.)**					**629**							
1,1,1,2,2,3,3,4,4-Nonafluorobutane	375-17-7	HFC-329p	$CHF_2CF_2CF_2CF_3$	28.4	32.0	*WMO* (2019)	0.284	0.284	0.319	0.313	0.310	**(Young et al., 2009b)**	I	NC	Pinnock		3,040	296	933	700-1435	22.2			
											0.306	H2013				2,360								
							0.284	0.284	0.319	**0.313**		**This study (avg.)**					**3,040**							
trifluoroethylene	359-11-5	HFO-1123	$CHF{=}CF_2$		1.4 days	*WMO* (2019)					0.002	(Munkhbayar Baasandorj and Burkholder, 2016)		H	H2013			296		700-1910	11.1			
											**0.002**	**This study**					**<1**							(4)
1,1-Difluoroethene	75-38-7	HFO-1132a	$CH_2{=}CF_2$	4.0 days	4.6 days	*WMO* (2019)					0.086	(M. Baasandorj et al., 2010)	I	NC	Pinnock			296		745-1820	10.1			
											**0.004**	**H2013**				**<1**								
											0.004	This study					<1							(2)
Fluoroethene	75-02-5	HFO-1141	$CH_2{=}CHF$	2.1 days	2.5 days	*WMO* (2019)					0.084	(M. Baasandorj et al., 2010)	I	NC	Pinnock			296		770-1740	4.8			
												(Stoppa et al., 2009)								770-1740	4.5			
							0.081	0.079	0.089	0.002		**(Sharpe et al., 2004)**					<1	298	1013	500-3000	5.4		H16	
											**0.002**	**H2013**				**<1**								
							0.081	0.079	0.089	0.002		This study (avg.)					<1							
(Z)-1,2,3,3,3-Pentafluoroprop-1-ene	5528-43-8	HFO-1225ye(Z)	(Z)-$CF_3CF{=}CHF$	8.5 days	10.0 days	*WMO* (2019)					0.260	(Papadimitriou et al., 2008a)	A	NC	LBL			296		706-1812	19.3			
							0.238	0.237	0.265	0.025	0.250	**(Hurley et al., 2007)**	I	NC	Pinnock		<1	296	933	710-2000	18.3			
											0.021	H2013				**<1**								
							0.238	0.237	0.265	**0.025**		**This study (avg.)**					<1							
(E)-1,2,3,3,3-Pentafluoroprop-1-ene	5595-10-8	HFO-1225ye(E)	(E)-$CF_3CF{=}CHF$	4.9 days	5.7 days	*WMO* (2019)					0.290	(Antinolo et al., 2017)	I		H2013			298		500-2000	20.5			
											0.040	(N Zhang et al., 2015)		H	Pinnock					400-2500	20.3			
							0.226	0.227	0.259	0.015	0.240	**(Hurley et al., 2007)**	I	NC	Pinnock		<1	296	933	810-2000	18.4			
											0.013	H2013				**<1**								
							0.226	0.227	0.259	**0.015**		**This study (avg.)**					<1							
(Z)-1,3,3,3-Tetrafluoroprop-1-ene	29118-25-0	HFO-1234ze(Z)	(Z)-$CF_3CH{=}CHF$	10.0 days	10.0 days	*WMO* (2019)					0.230	(Antinolo et al., 2017)	I		H2013			298		500-2000	18.0			
											0.020	(N Zhang et al., 2015)		H	Pinnock					400-2500	18.8			
							0.186	0.186	0.209	0.020	0.200	**(Nilsson et al., 2009)**	I	NC	Pinnock		<1	296	933	720-2000	16.8			
											0.019	H2013				**<1**								
							0.186	0.186	0.209	**0.020**		**This study (avg.)**					<1							

Table S9 (cont.)

				Lifetime (yr)			RE (W m^{-2} ppb^{-1}) – This study				RE (W m^{-2} ppb^{-1}) – Literature					GWP 100-yr		Absorption cross-sections						
Name	CASRN	Identifier	Formula	H2013	New	Reference	H2013 inst. RE	New inst. RE	New RE – const. profile	New RE – lifetime corr.	Value	Reference	Instantaneous/Adjusted	Vertical correction	RE calculation	H2013	New – lifetime corr.	*T* (K)	*p* (hPa)	Waveno. range (cm^{-1})	Int. abs. cross-section (10^{-17} cm^2 molecule^{-1})	Reference	Database	Notes
(E)-1,3,3,3-Tetrafluoroprop-1-ene	29188-24-9	HFO-1234ze(E)	(E)-$CF_3CH=CHF$	16.4 days	19.0 days	*WMO* (2019)	0.256	0.254	0.286	0.045	0.240	**(Orkin et al., 2010)**					1	295		500-1900	19.9			(5)
							0.251	0.249	0.281	0.045	0.270	**(Søndergaard et al., 2007)**	I	NC	Pinnock		1	296	933	680-2000	19.4			
											0.039	H2013				**1**								
							0.253	0.251	0.284	**0.045**		**This study (avg.)**					1							
2,3,3,3-Tetrafluoroprop-1-ene	754-12-1	HFO-1234yf	$CF_3CF=CH_2$	10.5 days	12.0 days	*WMO* (2019)					0.023	(Tokuhashi et al., 2018)	A	H	H2013			295		400-2500	16.8			
							0.217	0.216	0.245	0.027	0.230	**(Orkin et al., 2010)**					<1	295		500-1900	17.2			(6)
											0.240	(Papadimitriou et al., 2008a)	A	NC	LBL			296		540-1810	16.9			
							0.204	0.203	0.232	0.025	0.220	**(Nielsen et al., 2007)**	I	NC	Pinnock		<1	296	933	863-1745	16.1			
											0.023	H2013				**<1**								
							0.210	0.210	0.238	**0.026**		**This study (avg.)**					<1							
(E)-1,1,1,4,4,4-Hexafluorobut-2-ene	N/A	HFO-1336mzz(E)	(E)-$CF_3CH=CHCF_3$		0.3	*WMO* (2019)					0.150	(M. Baasandorj et al., 2018)	A	H	H2013			296		550-2000	30.2			
											0.113	(Østerstrøm et al., 2017)		H	Pinnock			296		550-2000	29.6			
											**0.132**	**This study**					**19**							(7)
(Z)-1,1,1,4,4,4-Hexafluorobut-2-ene	692-49-9	HFO-1336mzz(Z)	(Z)-$CF_3CH=CHCF_3$	22.0 days	27.0 days	*WMO* (2019)					0.069	(Østerstrøm et al., 2017)		H	Pinnock			296		550-2000	25.1			
											0.380	(M. Baasandorj et al., 2011)	I	NC	Pinnock			296		1100-1500	27.8			
											0.074	H2013				**2**								
											**0.069**	**This study**					2							(8)
3,3,3-Trifluoroprop-1-ene	677-21-4	HFO-1243zf	$CF_3CH=CH_2$	7.0 days	9.0 days	*WMO* (2019)					0.016	(Tokuhashi et al., 2018)	A	H	H2013			295		400-2500	14.7			
											0.019	(González et al., 2015)	I	H	H2013			298		650-1800	15.3			
							0.159	0.158	0.177	0.015	0.159	**(M P S Andersen et al., 2012a)**	I	NC	Pinnock		<1	298	933	650-2000	12.5			
											0.012	H2013				**<1**								
							0.159	0.158	0.177	**0.015**		**This study (avg.)**					<1							
3,3,4,4,4-Pentafluorobut-1-ene	374-27-6	HFC-1345zfc	$CF_3CF_2CH=CH_2$	7.6 days	9.0 days	*WMO* (2019)	0.176	0.175	0.188	0.016	0.176	**(M P S Andersen et al., 2012a)**	I	NC	Pinnock		<1	298	933	650-2000	13.6			
											0.014	H2013				**<1**								
							0.176	0.175	0.188	**0.016**		**This study (avg.)**					<1							
3,3,4,4,5,5,6,6,6-Nonafluorohex-1-ene	19430-93-4		n-$C_4F_9CH=CH_2$	7.6 days	9.0 days	*WMO* (2019)					0.033	(González et al., 2016)	A	H	H2013			298		650-1800	29.5			
							0.320	0.318	0.354	0.030	0.338	**(M P S Andersen et al., 2012a)**	I	NC	Pinnock		<1	298	933	650-2000	24.1			
											0.026	H2013				**<1**								
							0.320	0.318	0.354	**0.030**		**This study (avg.)**					<1							
3,3,4,4,5,5,6,6,7,7,8,8,8-Tridecafluorooct-1-ene	25291-17-2		n-$C_6F_{13}CH=CH_2$	7.6 days	9.0 days	*WMO* (2019)					0.039	(González et al., 2016)	A	H	H2013			298		650-1800	37.3			
							0.357	0.356	0.396	0.034	0.376	**(M P S Andersen et al., 2012a)**	I	NC	Pinnock		<1	298	933	650-2000	29.6			
											0.029	H2013				**<1**								
							0.357	0.356	0.396	**0.034**		**This study (avg.)**					<1							
3,3,4,4,5,5,6,6,7,7,8,8,9,9,10,10,10-Heptadecafluorodec-1-ene	21652-58-4		n-$C_8F_{17}CH=CH_2$	7.6 days	9.0 days	*WMO* (2019)	0.396	0.396	0.444	0.038	0.418	**(M P S Andersen et al., 2012a)**	I	NC	Pinnock		<1	298	933	650-2000	33.7			
											0.032	H2013				**<1**								
							0.396	0.396	0.444	**0.038**		**This study (avg.)**					<1							
1-Propene, 3,3,3-trifluoro-2-(trifluoromethyl)-	382-10-5		$(CF_3)_2C=CH_2$		10.3 days	(Papadimitriou et al., 2015)					0.041	(Tokuhashi et al., 2018)	A	H	H2013			295		400-2500	25.7			
											0.033	(Papadimitriou et al., 2015)		H	H2013			296	1013	600-1600				
							0.300	0.300	0.341	0.033		**(Sharpe et al., 2004)**					<1	296		525-3000	27.1		P	
							0.300	0.300	0.341	**0.033**		**This study**					**<1**							
1,1,2,2,3,3,4-heptafluorocyclopentane	15290-77-4		cyc (-$CF_2CF_2CF_2CHFCH_2$-)		2.8	(N Zhang et al., 2015)					0.243	(N Zhang et al., 2015)	A	H	H2013					400-2500	18.6			
											**0.243**	**This study**					**243**							(9)
1,1,2,2,3,3-hexafluorocyclopentane	123768-18-3		cyc (-$CF_2CF_2CF_2CH_2CH_2$-)		1.6	(Guo et al., 2019)					0.200	(Guo et al., 2019)	A	H	Pinnock			298		400-2500	17.3			
											**0.200**	**This study**					**126**							(10)

Table S9 (cont.)

Name	CASRN	Identifier	Formula	Lifetime (yr) H2013	Lifetime (yr) New	Lifetime (yr) Reference	RE ($W m^{-2} ppb^{-1}$) – This study: H2013 inst. RE	New inst. RE	New RE – const. profile	New RE – lifetime corr.	RE ($W m^{-2} ppb^{-1}$) – Literature: Value	Reference	Instantaneous/Adjusted	Vertical correction	RE calculation	GWP 100-yr: H2013	New – lifetime corr.	Absorption cross-sections: *T* (K)	*p* (hPa)	Waveno. range (cm^{-1})	Int. abs. cross-section (10^{-17} cm^2 $molecule^{-1}$)	Reference	Database	Notes
1,3,3,4,4,5,5-heptafluorocyclopentene	1892-03-1		cyc (-$CF_2CF_2CF_2CF$=CH-)		0.6	(Liu et al., 2016)					0.215	(Liu et al., 2016)	A	H	H2013			298		400-2500	23.7			
											**0.215**	**This study**					**47**							(11)
(4R,5R)-1,1,2,2,3,3,4,5-octafluorocyclopentane	158389-18-5		trans-cyc (-$CF_2CF_2CF_2$CHFCHF-)		3.2	(N Zhang et al., 2015)					0.259	(N Zhang et al., 2015)	A	H	H2013					400-2500	19.0			
											**0.259**	**This study**					**271**							(9)
1-Butene, 1,3,4,4,4-pentafluoro-3-(trifluoromethyl)-, (1E)-	14149-41-8	HFO-1438ezy(E)	(E)-$(CF_3)_2$CFCH=CHF		0.3	*WMO* (2019)					0.079	(Papadimitriou and Burkholder, 2016)		H	H2013			296		660-1800	28.6			
											**0.079**	**This study**					**9**							(12)
1-Pentene, 3,3,4,4,5,5,5-heptafluoro-	355-08-8	HFO-1447fz	$CF_3(CF_2)_2CH$=CH_2		9.0 days	*WMO* (2019)					0.028	(Jiménez et al., 2016)		H	H2013					500-2500				
											**0.028**	**This study**					**<1**							(13)
1,3,3,4,4-pentafluorocyclobutene	374-31-2		cyc (-CH=$CFCF_2CF_2$-)		0.7	*WMO* (2019)					0.270	(Jia et al., 2013)		NC	Pinnock			298		400-2500	21.2			
											**0.270**	**This study**					**97**							(14)
3,3,4,4-tetrafluorocyclobutene	2714-38-7		cyc (-CH=$CHCF_2CF_2$-)		84.0 days	*WMO* (2019)					0.210	(Jia et al., 2013)		NC	Pinnock			298		400-2500	16.5			
											**0.210**	**This study**					**27**							(14)
3-Fluoro-1-propene	818-92-8	Allyl fluoride	CH_2=$CHCH_2F$				0.060	0.059	**0.059**			**(Sharpe et al., 2004)**						296		500-3000	4.4		P	
1-Fluorohexane	373-14-8		n-$C_6H_{13}F$				0.042	0.041	**0.041**			**(Sharpe et al., 2004)**						296		600-3000	9.3		P	
Fluorobenzene	462-06-6		C_6H_5-F				0.060	0.060	**0.065**			**(Sharpe et al., 2004)**						296		600-3000	6.3		P	

(1) Inst. clear-sky RE, a factor 0.8 was applied to obtain cloudy-sky adjusted; (2) RE value is retained from H2013, but the GWP value is updated to reflect possible changes in lifetime and $AGWP_{CO2}$; (3) Scaled to the CFC-11 RE of 0.26 $W m^{-2} ppb^{-1}$; (4) RE value is from (Munkhbayar Baasandorj and Burkholder, 2016); (5) Value of 0.24 not given in paper, but assumed from tau=19 days and GWP(100)=7.5; (6) Value of 0.23 not given in paper, but assumed from tau=12 days and GWP(100)=4.4; (7) RE value is an average of (M. Baasandorj et al., 2018; Østerstrøm et al., 2017); (8) RE value is from (Østerstrøm et al., 2017) with lifetime correction included; (9) RE value is from (N Zhang et al., 2015); (10) RE value is from (Guo et al., 2019); (11) RE value is from (Liu et al., 2016); (12) RE value is from (Papadimitriou and Burkholder, 2016); (13) RE value is from (Jiménez et al., 2016); (14) RE value is from (Jia et al., 2013)

Table S10. Other Chlorocarbons and Hydrochlorocarbons

Please refer to page 2 of this document for a description of the table.

				Lifetime (yr)			RE (W m^{-2} ppb^{-1}) – This study				RE (W m^{-2} ppb^{-1}) – Literature					GWP 100-yr		Absorption cross-sections						
Name	CASRN	Identifier	Formula	H2013	New	Reference	H2013 inst. RE	New inst. RE	New RE – const. profile	New RE – lifetime corr.	Value	Reference	Instantaneous/Adjusted	Vertical correction	RE calculation	H2013	New – lifetime corr.	*T* (K)	*p* (hPa)	Waveno. range (cm^{-1})	Int. abs. cross-section (10^{-17} cm^2 molecule^{-1})	Reference	Database	Notes
Chloroethane	75-00-3		CH_3CH_2Cl		48.0 days	*WMO* (2019)	0.011	0.010	0.011	**0.004**		**(Sharpe et al., 2004)**					**<1**	278	1013	590-3000	2.4		H16	
1,1-Dichloroethane	75-34-3		CH_3CHCl_2				0.028	0.028	**0.028**			**(Sharpe et al., 2004)**						298	1013	560-3000	3.7		H16	
1,2-Dichloroethane	107-06-2		CH_2ClCH_2Cl	65.0 days	82.0 days	*WMO* (2019)	0.020	0.020	0.021	0.009		**(Sharpe et al., 2004)**					1	298	1013	600-3000	2.3		H16	
											0.021	(Sihra et al., 2001)	A	NC	LBL/NBM							(Vander Auwera, 2000)		
							0.020	0.020	0.020	0.008	0.020	**(Highwood and Shine, 2000)**	A	O	NBM		1	273		620-1570	1.9	(Vander Auwera, 2000)		
												(Vander Auwera, 2000)						293		600-1700	2.1			
											0.008	H2013				**1**								
							0.020	0.020	0.020	**0.009**		**This study (avg.)**					1							
1,1,2-Trichloroethane	79-00-5		$CH_2ClCHCl_2$				0.048	0.047	**0.050**			**(Sharpe et al., 2004)**						298	1013	600-3000	3.6		H16	
1,1,1,2-Tetrachloroethane	630-20-6		CH_2ClCCl_3				0.099	0.097	**0.103**			**(Sharpe et al., 2004)**						278	1013	530-3000	5.1		H16	
1,1,2,2-Tetrachloroethane	79-34-5		$CHCl_2CHCl_2$				0.091	0.090	**0.096**			**(Sharpe et al., 2004)**						278	1013	530-3000	4.5		H16	
1,1,2-Trichloroethene	79-01-6		$CHClCCl_2$		5.6 days	*WMO* (2019)	0.092	0.089	0.099	**0.006**		**(Sharpe et al., 2004)**					**<1**	298	1013	595-3000	3.8		H16	
1,1,2,2-Tetrachloroethene	127-18-4		$CCl_2{=}CCl_2$		0.3	*WMO* (2019)	0.099	0.096	0.107	**0.052**		**(Sharpe et al., 2004)**					**7**	298	1013	600-3000	3.6		H16	
2-Chloropropane	75-29-6		$CH_3CHClCH_3$		22.0 days	*WMO* (2019)	0.019	0.019	0.020	**0.004**		**(Sharpe et al., 2004)**					**<1**	296		575-3000	3.7		P	
Chloromethyl benzene	100-44-7		C_6H_5-CH_2Cl				0.023	0.023	**0.024**			**(Sharpe et al., 2004)**						296		600-3000	3.7		P	
3-Chloro-1-propene	107-5-1		$CH_2{=}CHCH_2Cl$				0.044	0.043	**0.046**			**(Sharpe et al., 2004)**						296		525-3000	3.0		P	
1-Chloro-4-methylbenzene	106-43-4		p-Cl-C_6H_4-CH_3				0.047	0.046	**0.050**			**(Sharpe et al., 2004)**						296		600-3000	4.4		P	
3,4-Dichloro-1-butene	760-23-6		$CH_2ClCHClCH{=}CH_2$				0.052	0.051	**0.055**			**(Sharpe et al., 2004)**						296		575-3000	3.6		P	
1-Chloro-3-methylbenzene	108-41-8		m-Cl-C_6H_4-CH_3				0.050	0.049	**0.054**			**(Sharpe et al., 2004)**						296		600-3000	5.3		P	
2,3-Dichloropropene	78-88-6		$CH_2ClCCl{=}CH_2$				0.048	0.047	**0.052**			**(Sharpe et al., 2004)**						296		580-3000	3.8		P	
1-Chloro-2-methylbenzene	95-49-8		o-Cl-C_6H_4-CH_3				0.037	0.036	**0.036**			**(Sharpe et al., 2004)**						296		600-3000	4.2		P	
1,2-Dichloropropene	563-54-2		$CHCl{=}CClCH_3$				0.025	0.024	**0.025**			**(Sharpe et al., 2004)**						296		590-3000	3.3		P	
1-Chloropentane	543-59-9		$CH_3(CH_2)_3CH_2Cl$				0.015	0.015	**0.016**			**(Sharpe et al., 2004)**						296		590-3000	5.9		P	
1-Chlorobutane	109-69-3		$CH_3(CH_2)_2CH_2Cl$		4.5 days	(Jara-Toro et al., 2020)					0.001	(Jara-Toro et al., 2020)	I	H	Pinnock			298		500-1500	1.8			
							0.015	0.015	**0.016**	0.001		**(Sharpe et al., 2004)**					<1	296		540-3000	4.8		P	
							0.015	0.015	0.016	**0.001**		**This study (avg.)**					**<1**							
1-Chloro-2-methylpropane	513-36-0		$(CH_3)_2CHCH_2Cl$				0.019	0.019	**0.020**			**(Sharpe et al., 2004)**						296		550-3000	4.9		P	
Chloroethene	75-01-4		$CH_2{=}CHCl$				0.040	0.039	**0.041**			**(Sharpe et al., 2004)**						298	1013	540-3000	3.3		H16	
1,2-Dichloroethene (E)	156-60-5		$C_2H_2Cl_2$				0.084	0.082	**0.091**			**(Sharpe et al., 2004)**						298	1013	540-3000	3.2		H16	
Hexachloro-1,3-butadiene	87-68-3		$CCl_2{=}CClCCl{=}CCl_2$				0.134	0.131	**0.144**			**(Sharpe et al., 2004)**						298	1013	530-3000	6.8		H16	
1,3-Dichloropropene (E)	10061-02-6		(E)-$CHCl{=}CHCH_2Cl$				0.052	0.051	**0.056**			**(Sharpe et al., 2004)**						278	1013	600-3000	4.4		H16	
1,3-Dichloropropene (Z)	10061-01-5		(Z)-$CHCl{=}CHCH_2Cl$				0.057	0.056	**0.061**			**(Sharpe et al., 2004)**						296		600-3000	4.0		P	
1,3-Dichloropropane	142-28-9		$CH_2ClCH_2CH_2Cl$				0.027	0.026	**0.029**			**(Sharpe et al., 2004)**						298	1013	570-3000	3.4		H16	
Chlorobenzene	108-90-7		C_6H_5-Cl				0.038	0.038	**0.039**			**(Sharpe et al., 2004)**						298	1013	600-3000	4.1		H16	
1,4-Dichlorobenzene	106-46-7		p-Cl-C_6H_4-Cl				0.070	0.069	**0.075**			**(Sharpe et al., 2004)**						298	1013	600-3000	5.0		H16	
1,3-Dichlorobenzene	541-73-1		m-Cl-C_6H_4-Cl				0.075	0.073	**0.080**			**(Sharpe et al., 2004)**						278	1013	600-3000	6.0		H16	
1,2-Dichlorobenzene	95-50-1		o-Cl-C_6H_4-Cl				0.046	0.045	**0.046**			**(Sharpe et al., 2004)**						298	1013	600-3000	4.4		H16	
1,2-Dichloroethylene (Z)	156-59-2		(Z)-$CHCl{=}CHCl$				0.041	0.040	**0.043**			**(Sharpe et al., 2004)**						296		530-3000	3.4		P	
Hexachloro-1,3-cyclopentadiene	77-47-4		C_5Cl_6				0.100	0.099	**0.110**			**(Sharpe et al., 2004)**						323	1013	575-3000	7.5		H16	
3-Chloro-1-propyne	624-65-7		$CH_2ClC{\equiv}CH$				0.023	0.023	**0.024**			**(Sharpe et al., 2004)**						296		550-3000	3.4		P	

Table S11. Other Bromocarbons, Hydrobromocarbons and Halons

Please refer to page 2 of this document for a description of the table.

				Lifetime (yr)			RE (W m^{-2} ppb^{-1}) – This study				RE (W m^{-2} ppb^{-1}) – Literature					GWP 100-yr		Absorption cross-sections						
Name	CASRN	Identifier	Formula	H2013	New	Reference	H2013 inst. RE	New inst. RE	New RE – const. profile	New RE – lifetime corr.	Value	Reference	Instantaneous/Adjusted	Vertical correction	RE calculation	H2013	New – lifetime corr.	*T* (K)	*p* (hPa)	Waveno. range (cm^{-1})	Int. abs. cross-section (10^{-17} cm^2 molecule^{-1})	Reference	Database	Notes
Dibromomethane	74-95-3	Methylene bromide	CH_2Br_2	0.3	0.4	*WMO* (2019)	0.015	0.015	0.017	0.010		**(Sharpe et al., 2004)**					2	298	1013	540-3000	2.2		H16	
							0.015	0.015	0.017	0.009	0.011	**(Sihra et al., 2001)**	A	S	LBL/NBM		2	296	933	450-2000	2.1			
											0.019	(Jain et al., 2000)	A	NC	NBM							(Christidis et al., 1997)		
											0.021	(Christidis et al., 1997)	A	NC	NBM			296	921	450-2000	2.0			
											0.009	H2013				1								
							0.015	0.015	0.017	**0.010**		**This study (avg.)**					**2**							
Bromodifluoromethane	1511-62-2	Halon-1201	$CHBrF_2$	5.2	4.9	*WMO* (2019)	0.149	0.148	0.163	0.150		**(Charmet et al., 2010)**					393	298		530-1460	10.2			
							0.151	0.150	0.165	0.152	0.177	**(Orkin et al., 2003)**	I	NC			398	295		530-1400	10.1			(1)
							0.153	0.152	0.167	0.154	0.146	**(Sihra et al., 2001)**	A	S	LBL/NBM		404	296	933	450-2000	10.0			
											0.174	(Jain et al., 2000)	A	NC	NBM							(Christidis et al., 1997)		
											0.179	(Christidis et al., 1997)	A	NC	NBM			296	921	450-2000	9.9			
											0.154	H2013				376								
							0.151	0.150	0.165	**0.152**		**This study (avg.)**					**398**							
Dibromodifluoromethane	75-61-6	Halon-1202	CBr_2F_2	2.9	2.5	*WMO* (2019)	0.293	0.288	0.321	0.278		**(Sharpe et al., 2004)**					232	298	1013	575-3000	12.6		H16	
							0.280	0.275	0.306	0.266	0.294	**(Orkin et al., 2003)**	I	NC			221	295		400-1600	11.8			(1)
											**0.272**	**H2013**				231								
							0.287	0.281	0.313	0.272		This study (avg.)					**226**							
2-Bromo-1,1,1-trifluoroethane	421-06-7	Halon-2301	CH_2BrCF_3	3.4	3.2	*WMO* (2019)	0.137	0.137	0.152	0.135	0.174	**(Orkin et al., 2003)**	I	NC			186	295		590-1510	12.8			(1)
											**0.135**	**H2013**				173								
							0.137	0.137	0.152	0.135		This study (avg.)					**186**							
2-Bromo-2-chloro-1,1,1-trifluoroethane	151-67-7	Halon-2311 (Halothane)	$CHBrClCF_3$	1.0	1.0	*WMO* (2019)	0.156	0.155	0.174	0.129	0.165	**(M P S Andersen et al., 2012b)**	I	NC	Pinnock		46	298		650-1500	12.2			
							0.167	0.166	0.186	0.138	0.205	**(Orkin et al., 2003)**	I	NC			49	295		480-1370	13.0			(1)
											0.132	H2013				41								
							0.162	0.161	0.180	**0.133**		**This study (avg.)**					**47**							
2-Bromo-1,1,1,2-tetrafluoroethane	124-72-1	Halon-2401	$CHBrFCF_3$	2.9	2.9	*WMO* (2019)	0.192	0.191	0.214	0.189	0.226	**(Orkin et al., 2003)**	I	NC			211	295		490-1480	14.6			(1)
											0.186	H2013				184								
							0.192	0.191	0.214	**0.189**		**This study (avg.)**					**211**							
Tribromomethane	75-25-2		$CHBr_3$		57.0 days	*WMO* (2019)	0.016	0.016	0.018	**0.006**		**(Sharpe et al., 2004)**					**<1**	298	1013	600-3000	3.5		H16	
Bromochloromethane	74-97-5	Halon-1011	CH_2BrCl		0.5	*WMO* (2019)	0.034	0.034	0.035	**0.020**		**(Sharpe et al., 2004)**					**5**	278	1013	550-3000	2.6		H16	
Bromoethane	74-96-4		CH_3CH_2Br		50.0 days	*WMO* (2019)	0.017	0.017	0.018	**0.006**		**(Sharpe et al., 2004)**					**<1**	296		530-3000	2.3		P	
1,2-Dibromoethane	106-93-4	EDB	CH_2BrCH_2Br		89.0 days	*WMO* (2019)	0.026	0.026	0.027	**0.012**		**(Sharpe et al., 2004)**					**1**	278	1013	570-3000	2.1		H16	
1-Bromopropane	106-94-5		$CH_3CH_2CH_2Br$		15.0 days	*WMO* (2019)	0.017	0.017	0.018	**0.002**		**(Sharpe et al., 2004)**					**<1**	296		500-3000	3.5		P	
2-Bromopropane	75-26-3		$CH_3CHBrCH_3$		20.0 days	*WMO* (2019)	0.024	0.024	0.026	**0.004**		**(Sharpe et al., 2004)**					**<1**	296		500-3000	3.6		P	
Bromomethyl benzene	100-39-0		C_6H_5-CH_2Br				0.031	0.031	**0.032**			**(Sharpe et al., 2004)**						296		530-3000	3.5		P	
3-Bromo-1-propene	106-95-6		CH_2=$CHCH_2Br$				0.037	0.037	**0.040**			**(Sharpe et al., 2004)**						296		510-3000	2.9		P	
Bromine Nitrate	40423-14-1		$BrONO_2$				0.094	0.093	**0.102**			**(Wagner and Birk, 2016)**						293		765-1320	6.5		H16	
Bromoethene	593-60-2		CH_2=$CHBr$				0.039	0.039	**0.041**			**(Sharpe et al., 2004)**						296		525-3000	3.0		P	

(1) Scaled to the CFC-11 RE of 0.26 W m^{-2} ppb^{-1}

Table S12. Other Fully Fluorinated Species

Please refer to page 2 of this document for a description of the table.

				Lifetime (yr)			RE (W m^{-2} ppb^{-1}) – This study				RE (W m^{-2} ppb^{-1}) – Literature					GWP 100-yr		Absorption cross-sections						
Name	CASRN	Identifier	Formula	H2013	New	Reference	H2013 inst. RE	New inst. RE	New RE – const. profile	New RE – lifetime corr.	Value	Reference	Instantaneous/Adjusted	Vertical correction	RE calculation	H2013	New – lifetime corr.	*T* (K)	*p* (hPa)	Waveno. range (cm^{-1})	Int. abs. cross-section (10^{-17} cm^2 molecule^{-1})	Reference	Database	Notes
Pentadecafluorotriethylamine	359-70-6		$N(C_2F_5)_3$		1000.0	*WMO* (2019)					0.610	(Bernard et al., 2018)	A		H2013			294		570-1500	59.9			
											**0.610**	**This study**					**10,900**							(1)
Perfluorotripropylamine	338-83-0	PTPA	$N(CF_2CF_2CF_3)_3$		1000.0	*WMO* (2019)					0.750	(Bernard et al., 2018)	A		H2013			294		570-1500	74.9			
											**0.750**	**This study**					**9,580**							(1)
Heptacosafluorotributylamine	311-89-7	PFTBA	$N(CF_2CF_2CF_2CF_3)_3$		1000.0	*WMO* (2019)					0.870	(Bernard et al., 2018)	A		H2013			294		570-1500	88.9			
							0.842	0.842	0.931	0.914	0.770	**(Godin et al., 2016)**	A		H2013		9,070	298		570-1400	78.1		H16	(2)
							0.829	0.829	0.917	0.900	0.860	**(Hong et al., 2013)**	I		Pinnock		8,930	296		600-1500	84.7			(3)
							0.835	0.836	0.924	**0.907**		**This study (avg.)**					**9,000**							
Perfluorotripentylamine	338-84-1		$N(CF_2CF_2CF_2CF_2CF_3)_3$		1000.0	*WMO* (2019)					0.950	(Bernard et al., 2018)	A		H2013			294		570-1500	98.7			
											**0.950**	**This study**					**7,700**							(1)
Heptafluoroisobutyronitrile	42532-60-5		$(CF_3)_2CFCN$		34.5	(M P S Andersen et al., 2017b; Blazquez et al., 2017)					0.279	(Blazquez et al., 2017)			H2013					650-1500	28.8			
											0.217	(M P S Andersen et al., 2017b)	A	H	Pinnock			298		650-1500	22.2			
											**0.248**	**This study**					**2,900**							(4)
(Trifluoromethyl)sulfur pentafluoride	373-80-8		SF_5CF_3	800.0	800.0	*WMO* (2019)	0.581	0.568	0.631	0.619		(Sharpe et al., 2004)					20,800	296		520-3000	29.4		P	
							0.515	0.503	0.562	0.551		(Hurley-priv.com., 2003)					18,500	296	933	670-1325	25.2		G09	
							0.564	0.551	0.614	0.602		**(Rinsland et al., 2003)**					20,200	298	1013	599-2600	28.6		H08	
							0.533	0.521	0.579	0.568	0.590	**(Nielsen et al., 2002)**	A	NC	LBL/NBM		19,000	296		400-1330	26.5			
											0.567	(Sihra et al., 2001)	A	NC	LBL/NBM			296		700-2000	25.4			
											0.570	(Sturges et al., 2000)	A	NC	LBL/NBM			296	933	670-1325	25.4			
											0.592	H2013				17,400								
							0.549	0.536	0.596	**0.585**		**This study (avg.)**					**19,600**							
Hexafluorocyclobutene	697-11-0		cyc ($-CF=CFCF_2CF_2-$)		1.0	(Jia et al., 2013)					0.300	(Jia et al., 2013)		NC	Pinnock			298		400-2500	22.7			
											**0.300**	**This study**					**132**							(5)
Pentafluoro-2-(trifluoromethyl)-1-propene	382-21-8	PFIB	$(CF_3)_2C=CF_2$				0.322	0.320	**0.336**			**(Sharpe et al., 2004)**						296		520-3000	38.4		P	
Octafluorocyclopentene	559-40-0		cyc ($-CF_2CF_2CFCF_2CF_2-$)	31.0 days	1.1	*WMO* (2019)					0.246	(N Zhang et al., 2017)	A	H	H2013			298		500-2500	25.9			
											0.320	(Bravo et al., 2010b)	I	NC	Pinnock						24.0			
											0.224	(Vasekova et al., 2006)	I	F	Pinnock			297		550-1800	20.9			
											0.076	H2013				2								
											**0.246**	**This study**					**82**							(6)
Hexafluorobenzene	392-56-3		C_6F_6				0.163	0.160	**0.153**			**(Sharpe et al., 2004)**						298	1013	580-3000	19.7		H16	
Perfluorodecalin (mixed)	306-94-5	PFC-91-18	$C_{10}F_{18}$	2000.0	2000.0	*WMO* (2019)	0.510	0.508	0.545	0.537	0.560	**(Shine et al., 2005)**	A		LBL/NBM		7,940	296		0-1500	39.1			
											0.553	H2013				7,190								
							0.510	0.508	0.545	**0.537**		**This study (avg.)**					**7,940**							
Perfluorodecalin (cis)	60433-11-6		Z-$C_{10}F_{18}$	2000.0	2000.0	*WMO* (2019)	0.552	0.548	0.589	0.580	0.620	**(Le Bris et al., 2017)**	A	H	H2013		8,570	300	933	560-1500	42.7		H16	
							0.514	0.510	0.549	0.541		**(Shine et al., 2005)**					7,980	296	933	655-1370	37.9			
											0.557	H2013				7,240								
							0.533	0.529	0.569	**0.560**		**This study (avg.)**					**8,270**							
Perfluorodecalin (trans)	60433-12-7		E-$C_{10}F_{18}$	2000.0	2000.0	*WMO* (2019)	0.505	0.503	0.550	0.543	0.570	**(Le Bris et al., 2017)**	A	H	H2013		8,010	300	933	560-1500	42.3		H16	
							0.447	0.445	0.488	0.481		**(Shine et al., 2005)**					7,100	296	933	655-1360	35.6			
											0.484	H2013				6,290								
							0.476	0.474	0.519	**0.512**		**This study (avg.)**					**7,560**							

Table S12 (cont.)

				Lifetime (yr)			RE (W m^{-2} ppb^{-1}) – This study				RE (W m^{-2} ppb^{-1}) – Literature					GWP 100-yr		Absorption cross-sections						
Name	CASRN	Identifier	Formula	H2013	New	Reference	H2013 inst. RE	New inst. RE	New RE – const. profile	New RE – lifetime corr.	Value	Reference	Instantaneous/Adjusted	Vertical correction	RE calculation	H2013	New – lifetime corr.	*T* (K)	*p* (hPa)	Waveno. range (cm^{-1})	Int. abs. cross-section (10^{-17} cm^2 molecule^{-1})	Reference	Database	Notes
Perfluoroethene	116-14-3	PFC-1114	$CF_2=CF_2$	1.1 days	1.2 days	*WMO* (2019)					0.006	(Herath et al., 2016)	A	H	H2013			290						
											0.114	(Drage et al., 2006)	I	NC	Pinnock			296		1080-1400	12.0			
							0.109	0.111	0.126	0.002	0.006	**(Acerboni et al., 2001)**	A	M	BBM		<1	298		1080-1400	12.9			
											**0.002**	**H2013**				**<1**								
							0.109	0.111	0.126	0.002		This study (avg.)					<1							
Perfluoropropene	116-15-4	PFC-1216	$CF_3CF=CF_2$	4.9 days	5.5 days	*WMO* (2019)	0.228	0.227	0.238	0.013		**(Sharpe et al., 2004)**					<1	298	1013	500-3000	24.0		H16	
							0.229	0.229	0.239	0.013	0.035	**(Acerboni et al., 2001)**	A	M	BBM		<1	298		970-1850	23.5			
											**0.013**	**H2013**				**<1**								
							0.228	0.228	0.239	0.013		This study (avg.)					<1							
Hexafluorobuta-1,3-diene	685-63-2		$CF_2=CFCF=CF_2$	1.1 days	1.1 days	*WMO* (2019)					0.007	(Herath et al., 2016)	A	H	H2013			290						
											0.200	(Bravo et al., 2010b)	I	NC	Pinnock						20.7			
							0.217	0.216	0.244	0.003	0.013	**(Acerboni et al., 2001)**	A	M	BBM		<1	298		900-1850	21.8			
											**0.003**	**H2013**				**<1**								
							0.217	0.216	0.244	0.003		This study (avg.)					<1							
Octafluoro-1-butene	357-26-6		$CF_3CF_2CF=CF_2$	6.0 days	6.0 days	*WMO* (2019)	0.271	0.271	0.307	0.019	0.290	**(Young et al., 2009a)**	I	NC	Pinnock		<1	296	933	635-1830	24.0			
											0.018	H2013				**<1**								
							0.271	0.271	0.307	**0.019**		**This study (avg.)**					<1							
Octafluoro-2-buene	360-89-4		$CF_3CF=CFCF_3$	31.0 days	31.0 days	*WMO* (2019)	0.268	0.269	0.304	0.070	0.300	**(Cometto et al., 2010)**	I	NC	Pinnock		2	298		500-1410	25.3			
											0.320	(Young et al., 2009a)	I	NC	Pinnock			296	933	650-1500	28.8			
											0.068	H2013				**2**								
							0.268	0.269	0.304	**0.070**		**This study (avg.)**					2							

(1) RE value is from (Bernard et al., 2018); (2) Assuming constant decay lifetime corrections; (3) Integrated CS wrong in (Hong et al., 2013) - see (Godin et al., 2016); (4) RE value is an average of (M P S Andersen et al., 2017b; Blazquez et al., 2017); (5) RE value is from (Jia et al., 2013); (6) RE value is from (N Zhang et al., 2017)

Table S13. Halogenated Alcohols and Ethers

Please refer to page 2 of this document for a description of the table.

				Lifetime (yr)			RE (W m^{-2} ppb^{-1}) – This study				RE (W m^{-2} ppb^{-1}) – Literature					GWP 100-yr		Absorption cross-sections						
Name	CASRN	Identifier	Formula	H2013	New	Reference	H2013 inst. RE	New inst. RE	New RE – const. profile	New RE – lifetime corr.	Value	Reference	Instantaneous/Adjusted	Vertical correction	RE calculation	H2013	New – lifetime corr.	*T* (K)	*p* (hPa)	Waveno. range (cm^{-1})	Int. abs. cross-section (10^{-17} cm^2 molecule^{-1})	Reference	Database	Notes
(Difluoromethoxy)trifluoromethane	3822-68-2	HFE-125	CHF_2OCF_3	119.0	135.0	*WMO* (2019)	0.372	0.372	0.420	0.417	0.407	**(Sihra et al., 2001)**	A	S	LBL/NBM		15,100	296	933	450-1650	31.7			
												(Orkin et al., 1999)						295		980-1510	31.2			
											0.405	(Heathfield et al., 1998)	I	NC	Pinnock			298		475-1590	32.9			
											0.443	(Christidis et al., 1997)	A	NC	NBM			296		450-2000	29.2			
											0.405	H2013				12,400								
							0.372	0.372	0.420	**0.417**		**This study (avg.)**					**15,100**							
(Difluoromethoxy)difluoromethane	1691-17-4	HFE-134	CHF_2OCHF_2	24.4	26.9	*WMO* (2019)					0.440	(M P S Andersen et al., 2010a)	I	NC	Pinnock			296	933	700-1500	25.7			
											0.400	(Myhre et al., 1999)	A	O	LBL/BBM			298		25-3250	25.0			
												(Orkin et al., 1999)						295		741-1443	25.4			
											0.430	(Heathfield et al., 1998)	I	NC	Pinnock			298		750-1480	25.6			
							0.415	0.411	0.459	0.449	0.442	**(Imasu et al., 1995)**	I	O			6,980	296		700-1500	25.5			(1)
												(Garland et al., 1993)								770-1430	25.3			
											0.445	H2013				5,560								
							0.415	0.411	0.459	**0.449**		**This study (avg.)**					**6,980**							
Trifluoro(methoxy)methane	421-14-7	HFE-143a	CH_3OCF_3	4.8	4.9	*WMO* (2019)	0.189	0.190	0.213	0.196		**(Orkin et al., 2014b)**					671	298		450-2000	21.6			
							0.175	0.176	0.197	0.182	0.172	**(Sihra et al., 2001)**	A	S	LBL/NBM		622	296	933	550-1520	19.9			
											0.190	(Christensen et al., 1999)	I	NC	Pinnock									
											0.177	H2013				523								
							0.182	0.183	0.205	**0.189**		**This study (avg.)**					**647**							
1,1,1,2-Tetrafluoro-2-(trifluoromethoxy)ethane	2356-62-9	HFE-227ea	$CF_3CHFOCF_3$	51.6	54.8	*WMO* (2019)	0.407	0.409	0.464	0.458	0.402	**(Oyaro et al., 2005)**	I	NC	Pinnock		7,910	298		485-1600	37.0			
											0.360	(Takahashi et al., 2002)	I	NC	Pinnock			296	933	645-1460	33.3			
											0.310	(Jain et al., 2001)	A	O	NBM			298		700-1500	41.0			
							0.409	0.411	0.467	0.461	0.419	**(Imasu et al., 1995)**	I	O			7,960	296		700-1500	36.9			(1)
												(Suga et al., 1994)						296		500-2000	38.4			
											0.442	H2013				6,450								
							0.408	0.410	0.466	**0.459**		**This study (avg.)**					**7,930**							
2-Chloro-1-(difluoromethoxy)-1,1,2-trifluoroethane	13838-16-9	HCFE-235ca2 (enflurane)	CHF_2OCF_2CHFCl	4.3	4.4	*WMO* (2019)	0.421	0.418	0.465	0.425	0.447	**(M P S Andersen et al., 2012b)**	I	NC	Pinnock		712	298		650-1500	26.8			
							0.389	0.386	0.431	0.394	0.420	**(Dalmasso et al., 2006)**	I	NC	Pinnock		660			800-1500	25.1			
											0.407	H2013				583								
							0.405	0.402	0.448	**0.409**		**This study (avg.)**					**686**							
2-Chloro-2-(difluoromethoxy)-1,1,1-trifluoroethane	26675-46-7	HCFE-235da2 (isoflurane)	$CHF_2OCHClCF_3$	3.5	3.5	*WMO* (2019)	0.427	0.424	0.476	0.427	0.453	**(M P S Andersen et al., 2010c)**	I	NC	Pinnock		567	298		650-1500	29.1			
							0.426	0.423	0.473	0.424	0.453	**(Ryan and Nielsen, 2010)**	I	NC	Pinnock		563			400-1500	28.5			
							0.404	0.401	0.449	0.403	0.372	(Sihra et al., 2001)	A	S	LBL/NBM		535	296	933	450-1650	27.5			
											0.476	(Christidis et al., 1997)	A	NC	NBM			296	921	450-2000	26.3			
											0.421	H2013				491								
							0.426	0.424	0.475	**0.426**		**This study (avg.)**					**565**							
2-(Difluoromethoxy)-1,1,1,2-tetrafluoroethane	57041-67-5	HFE-236ea2 (desflurane)	$CHF_2OCHFCF_3$	10.8	14.1	*WMO* (2019)	0.441	0.439	0.494	0.476	0.469	**(M P S Andersen et al., 2010c)**	I	NC	Pinnock		2,790	298		650-1500	31.3			
							0.423	0.421	0.472	0.455	0.447	**(Oyaro et al., 2005)**	I	NC	Pinnock		2,670	298		485-1530	30.3			
							0.427	0.426	0.478	0.461	0.458	**(Imasu et al., 1995)**	I	O			2,710	296		700-1500	29.9			(1)
											0.453	H2013				1,790								
							0.431	0.429	0.482	**0.464**		**This study (avg.)**					**2,720**							
1,1,1-Trifluoro-2-(trifluoromethoxy)ethane	20193-67-3	HFE-236fa	$CF_3CH_2OCF_3$	7.5	7.5	*WMO* (2019)	0.344	0.346	0.393	0.371	0.354	**(Imasu et al., 1995)**	I	O			1,160	296		700-1500	33.5			(1)
											0.357	H2013				979								
							0.344	0.346	0.393	**0.371**		**This study (avg.)**					**1,160**							
1,1,1,2,2-Pentafluoro-2-methoxy-ethane	22410-44-2	HFE-245cb2	$CF_3CF_2OCH_3$	4.9	5.0	*WMO* (2019)					0.288	(Østerstrøm et al., 2016)		H	Pinnock			296		600-2000	22.5			
							0.322	0.323	0.365	0.336	0.333	**(Imasu et al., 1995)**	I	O			784	296		700-1500	24.6			(1)
											0.326	H2013				654								
							0.322	0.323	0.365	**0.336**		**This study (avg.)**					**784**							

Table S13 (cont.)

				Lifetime (yr)			RE (W m^{-2} ppb^{-1}) – This study				RE (W m^{-2} ppb^{-1}) – Literature					GWP 100-yr		Absorption cross-sections						
Name	CASRN	Identifier	Formula	H2013	New	Reference	H2013 inst. RE	New inst. RE	New RE – const. profile	New RE – lifetime corr.	Value	Reference	Instantaneous/Adjusted	Vertical correction	RE calculation	H2013	New – lifetime corr.	*T* (K)	*p* (hPa)	Waveno. range (cm^{-1})	Int. abs. cross-section (10^{-17} cm^2 molecule^{-1})	Reference	Database	Notes
1,1-Difluoro-2-(trifluoromethoxy)ethane	84011-15-4	HFE-245fa1	$CHF_2CH_2OCF_3$	6.6	6.7	*WMO* (2019)	0.297	0.297	0.335	0.314	0.309	**(Imasu et al., 1995)**	I	O			980	296		700-1500	27.9			(1)
											0.306	H2013				828								
							0.297	0.297	0.335	**0.314**		**This study (avg.)**					**980**							
2-(Difluoromethoxy)-1,1,1-trifluoroethane	1885-48-9	HFE-245fa2	$CHF_2OCH_2CF_3$	5.5	5.5	*WMO* (2019)	0.349	0.347	0.383	0.356		**(Orkin et al., 2014b)**					912	298		450-2000	26.2			
							0.355	0.353	0.391	0.363	0.374	**(Oyaro et al., 2005)**	I	NC	Pinnock		930	298		470-1550	26.5			
							0.350	0.348	0.385	0.357	0.332	**(Sihra et al., 2001)**	A	S	LBL/NBM		916	296	933	450-1550	26.3			
											0.393	(Christidis et al., 1997)	A	NC	NBM			296	921	450-2000	25.1			
							0.354	0.352	0.391	0.362	0.374	**(Imasu et al., 1995)**	I	O			929	296		700-1500	26.3			(1)
											**0.360**	**H2013**				812								
							0.352	0.350	0.388	0.360		This study (avg.)					**922**							
2,2,3,3,3-Pentafluoropropan-1-ol	422-05-9		$CF_3CF_2CH_2OH$	0.3	0.5	*WMO* (2019)	0.262	0.262	0.289	0.171	0.260	**(Godin et al., 2017b)**	I		H2013		38	298		565-3040	20.0		H16	
							0.261	0.261	0.288	0.170	0.260	**(Antiñolo et al., 2012b)**	I	NC	Pinnock		37	298		600-4000	20.5			
							0.241	0.240	0.265	0.157	0.252	**(Sellevåg et al., 2007)**	I	NC	Pinnock		34	298		490-1500	18.0			
							0.240	0.240	0.266	0.157	0.252	**(Imasu et al., 1995)**	I	O			35	296		700-1500	17.9			(1)
											0.139	H2013				19								
							0.251	0.251	0.277	**0.164**		**This study (avg.)**					**36**							
1,1,2,2-Tetrafluoro-1-methoxyethane	425-88-7	HFE-254cb1	$CH_3OCF_2CHF_2$	2.5	2.5	*WMO* (2019)					0.300	(Heathfield et al., 1998)	I	NC	Pinnock			298		500-1600	19.6			
							0.271	0.269	0.300	0.260	0.286	**(Imasu et al., 1995)**	I	O			344	296		700-1500	19.1			(1)
											0.258	H2013				301								
							0.271	0.269	0.300	**0.260**		**This study (avg.)**					**344**							
1,1,1-Trifluoro-2-methoxyethane	460-43-5	HFE-263mf	$CF_3CH_2OCH_3$	23.0 days	28.0 days	*WMO* (2019)	0.192	0.191	0.215	0.046	0.210	**(Østerstrøm et al., 2012)**	I	NC	Pinnock		2	296	933	600-1600	15.5			
							0.194	0.193	0.217	0.046	0.190	**(Oyaro et al., 2005)**	I	NC	Pinnock		2	298		500-1525	15.3			
							0.194	0.193	0.217	0.046	0.207	**(Imasu et al., 1995)**	I	O			2	296		700-1500	15.2			(1)
											0.039	H2013				1								
							0.193	0.192	0.216	**0.046**		**This study (avg.)**					**2**							
1,1,2,2-Tetrafluoro-1-(trifluoromethoxy)ethane	690-22-2	HFE-263m1	$CF_3OCH_2CH_3$	0.4	0.4	*WMO* (2019)	0.202	0.203	0.227	0.126	0.210	**(Oyaro et al., 2005)**	I	NC	Pinnock		31	298		550-1550	22.0			
											0.127	H2013				29								
							0.202	0.203	0.227	**0.126**		**This study (avg.)**					**31**							
3,3,3-Trifluoropropan-1-ol	2240-88-2		$CF_3CH_2CH_2OH$	12.0 days	15.0 days	*WMO* (2019)	0.205	0.203	0.221	0.029	0.200	**(Jimenez et al., 2010)**	I	NC	Pinnock		<1	298		500-4000	16.6			
							0.165	0.164	0.179	0.024	0.172	**(Sellevåg et al., 2007)**	I	NC	Pinnock		<1	298		515-1550	13.8			
							0.174	0.173	0.190	0.025		**(Waterland et al., 2005)**					<1	296	933	650-1530	14.6			
											0.022	H2013				<1								
							0.181	0.180	0.196	**0.026**		**This study (avg.)**					**<1**							
1,1,1,2,2-Pentafluoro-2-(1,1,2,2-tetrafluoroethoxy)ethane	134769-21-4	HFE-329mcc2	$CHF_2CF_2OCF_2CF_3$	22.5	25.0	*WMO* (2019)	0.494	0.494	0.559	0.545	0.510	**(Imasu et al., 1995)**	I	O			3,970	296		700-1500	39.2			(1)
											0.529	H2013				3,070								
							0.494	0.494	0.559	**0.545**		**This study (avg.)**					**3,970**							
2-(Difluoromethoxy)-1,1,1,3,3,3-hexafluoropropane	26103-08-2	HFE-338mmz1	$(CF_3)_2CHOCHF_2$	21.2	22.3	*WMO* (2019)	0.413	0.412	0.464	0.452	0.429	**(Imasu et al., 1995)**	I	O			3,200	296		700-1500	35.4			(1)
											0.442	H2013				2,620								
							0.413	0.412	0.464	**0.452**		**This study (avg.)**					**3,200**							
1,1,1,2,2-Pentafluoro-2-(2,2,2-trifluoroethoxy)ethane	156053-88-2	HFE-338mcf2	$CF_3CH_2OCF_2CF_3$	7.5	7.5	*WMO* (2019)	0.424	0.425	0.481	0.454	0.439	**(Imasu et al., 1995)**	I	O			1,090	296		700-1500	34.7			(1)
											0.439	H2013				929								
							0.424	0.425	0.481	**0.454**		**This study (avg.)**					**1,090**							
1,1,1,3,3,3-Hexafluoro-2-(fluoromethoxy)propane	28523-86-6	Sevoflurane (HFE-347mmz1)	$(CF_3)_2CHOCH_2F$	2.2	1.9	*WMO* (2019)	0.339	0.339	0.367	0.307	0.351	**(M P S Andersen et al., 2010c)**	I	NC	Pinnock		204	298		650-1500	30.2			
							0.342	0.341	0.370	0.309	0.365	**(Ryan and Nielsen, 2010)**	I	NC	Pinnock		206			650-1500	30.6			
											0.320	H2013				216								
							0.341	0.340	0.369	**0.308**		**This study (avg.)**					**205**							

Table S13 (cont.)

Name	CASRN	Identifier	Formula	Lifetime (yr) H2013	Lifetime (yr) New	Lifetime (yr) Reference	RE ($W m^{-2} ppb^{-1}$) – This study: H2013 inst. RE	New inst. RE	New RE – const. profile	New RE – lifetime corr.	RE ($W m^{-2} ppb^{-1}$) – Literature: Value	Reference	Instantaneous/Adjusted	Vertical correction	RE calculation	GWP 100-yr: H2013	New – lifetime corr.	Absorption cross-sections: T (K)	p (hPa)	Waveno. range (cm^{-1})	Int. abs. cross-section (10^{-17} cm^2 $molecule^{-1}$)	Reference	Database	Notes
1,1,1,2,2,3,3-Heptafluoro-3-methoxypropane	375-03-1	HFE-347mcc3 (HFE-7000)	$CH_3OCF_2CF_2CF_3$	5.0	5.1	*WMO* (2019)	0.352	0.351	0.379	0.350	0.320	**(Bravo et al., 2010a)**	I	S	Pinnock		624	298		700-1400	28.2			
							0.321	0.321	0.347	0.320	0.340	**(Ninomiya et al., 2000)**	I	NC	Pinnock		571			700-1500	27.4			
							0.348	0.347	0.376	0.347	0.348	**(Imasu et al., 1995)**	I	O			619	296		700-1500	29.5			(1)
											0.345	H2013				530								
							0.340	0.340	0.367	**0.339**		**This study (avg.)**					**605**							
1-(2,2-Difluoroethoxy)-1,1,2,2,2-pentafluoroethane	171182-95-9	HFE-347mcf2	$CHF_2CH_2OCF_2CF_3$	6.6	6.7	*WMO* (2019)	0.408	0.408	0.460	0.431	0.424	**(Imasu et al., 1995)**	I	O			1,010	296		700-1500	30.2			(1)
											0.421	H2013				854								
							0.408	0.408	0.460	**0.431**		**This study (avg.)**					**1,010**							
1,1,2,2-Tetrafluoro-1-(2,2,2-trifluoroethoxy)ethane	406-78-0	HFE-347pcf2	$CHF_2CF_2OCH_2CF_3$	6.0	6.1	*WMO* (2019)					0.465	(Heathfield et al., 1998)	I	NC	Pinnock			298		500-1600	32.2			
											**0.482**	**H2013**				889								
											0.482	This study					**1,030**							(2)
1,1,1,2,3,3,3-Heptafluoro-2-methoxypropane	22052-84-2	HFE-347mmy1	$(CF_3)_2CFOCH_3$	3.7	3.7	*WMO* (2019)					0.294	(L L Andersen et al., 2014)			Pinnock			296		600-2000	664 0.0			
							0.322	0.322	0.353	0.318	0.322	**(Imasu et al., 1995)**	I	O			412	296		700-1500	28.6			(1)
											0.319	H2013				363								
							0.322	0.322	0.353	**0.318**		**This study (avg.)**					**412**							
1,1,1,2,3,3-Hexafluoro-3-methoxypropane	382-34-3	HFE-356mec3	$CH_3OCF_2CHFCF_3$	3.8	2.5	*WMO* (2019)					0.310	(Le Bris et al., 2020)	A	H	H2013			305		660-1650	27.7			
							0.303	0.302	0.333	0.288	0.312	**(Imasu et al., 1995)**	I	O			277	296		700-1500	25.8			(1)
											0.301	H2013				387								
							0.303	0.302	0.333	**0.288**		**This study (avg.)**					**277**							
Bis(2,2,2-trifluoroethyl)ether	333-36-8	HFE-356mff2	$CF_3CH_2OCH_2CF_3$	105.0 days	0.4	*WMO* (2019)	0.322	0.320	0.357	0.187	0.334	**(Oyaro et al., 2004)**	I	NC	Pinnock		25	298		500-1600	27.2			
							0.333	0.332	0.369	0.194	0.362	**(Sihra et al., 2001)**	A	NC	LBL/NBM		26	296	933	500-1500	28.1			
												(Orkin et al., 1999)						295		500-1600	27.7			
											0.350	(Wallington et al., 1998)	I	NC	Pinnock									
											0.172	H2013				17								
							0.327	0.326	0.363	**0.190**		**This study (avg.)**					**26**							
1-(2,2-Difluoroethoxy)-1,1,2,2-tetrafluoroethane	50807-77-7	HFE-356pcf2	$CHF_2CH_2OCF_2CHF_2$	5.7	6.0	*WMO* (2019)	0.365	0.363	0.406	0.378	0.385	**(Imasu et al., 1995)**	I	O			872	296		700-1500	24.9			(1)
											0.373	H2013				719								
							0.365	0.363	0.406	**0.378**		**This study (avg.)**					**872**							
3-(Difluoromethoxy)-1,1,2,2-tetrafluoropropane	35042-99-0	HFE-356pcf3	$CHF_2OCH_2CF_2CHF_2$	3.5	3.5	*WMO* (2019)	0.382	0.379	0.421	0.377	0.406	**(Imasu et al., 1995)**	I	O			508	296		700-1500	24.8			(1)
											**0.377**	**H2013**				446								
							0.382	0.379	0.421	0.377		This study (avg.)					**508**							
1,1,2,2,3,3-Hexafluoro-1-methoxypropane	160620-20-2	HFE-356pcc3	$CH_3OCF_2CF_2CHF_2$	3.8	2.5	*WMO* (2019)	0.324	0.321	0.349	0.303	0.335	**(Imasu et al., 1995)**	I	O			291	296		700-1500	23.4			(1)
											0.322	H2013				413								
							0.324	0.321	0.349	**0.303**		**This study (avg.)**					**291**							
1,1,1,3,3,3-Hexafluoro-2-methoxypropane	13171-18-1	HFE-356mmz1	$(CF_3)_2CHOCH_3$	97.1 days	65.0 days	*WMO* (2019)					0.105	(Østerstrøm et al., 2015)		H	Pinnock			296		650-1500	2.4			
							0.302	0.302	0.340	0.126	0.309	**(Oyaro et al., 2004)**	I	NC	Pinnock		9	298		475-1550	25.8			
							0.294	0.294	0.332	0.123	0.307	**(Imasu et al., 1995)**	I	O			8	296		700-1500	25.7			(1)
											0.151	H2013				14								
							0.298	0.298	0.336	**0.125**		**This study (avg.)**					**9**							
1,1,1,2,2-Pentafluoro-3-methoxypropane	378-16-5	HFE-365mcf3	$CF_3CF_2CH_2OCH_3$	19.3 days	25.0 days	*WMO* (2019)					0.330	(Thomsen et al., 2011)	I	NC	Pinnock			296	933	650-1500	20.7			
							0.263	0.262	0.292	0.057	0.276	**(Oyaro et al., 2004)**	I	NC	Pinnock		2	298		490-1525	19.5			
							0.265	0.264	0.296	0.058	0.281	**(Imasu et al., 1995)**	I	O			2	296		700-1500	19.6			(1)
											0.047	H2013				1								
							0.264	0.263	0.294	**0.058**		**This study (avg.)**					**2**							

Table S13 (cont.)

				Lifetime (yr)			RE (W m^{-2} ppb^{-1}) – This study				RE (W m^{-2} ppb^{-1}) – Literature					GWP 100-yr		Absorption cross-sections						
Name	CASRN	Identifier	Formula	H2013	New	Reference	H2013 inst. RE	New inst. RE	New RE – const. profile	New RE – lifetime corr.	Value	Reference	Instantaneous/Adjusted	Vertical correction	RE calculation	H2013	New – lifetime corr.	*T* (K)	*p* (hPa)	Waveno. range (cm^{-1})	Int. abs. cross-section (10^{-17} cm^2 molecule^{-1})	Reference	Database	Notes
1-Ethoxy-1,1,2,2-tetrafluoroethane	512-51-6	HFE-374pc2	$CHF_2CF_2OCH_2CH_3$	5.0	76.0 days	*WMO* (2019)					0.315	(Heathfield et al., 1998)	I	NC	Pinnock			298		500-1600	20.9			
							0.294	0.292	0.325	0.132	0.307	**(Imasu et al., 1995)**	I	O			13	296		700-1500	20.7			(1)
											0.298	H2013				627								
							0.294	0.292	0.325	**0.132**		**This study (avg.)**					**13**							
4,4,4-Trifluorobutan-1-ol	461-18-7		$CF_3(CH_2)_2CH_2OH$	4.0 days	5.4 days	*WMO* (2019)	0.110	0.110	0.116	0.006	0.110	**(Jimenez et al., 2010)**	I	NC	Pinnock		<1	298		500-4000	10.6			
											0.005	H2013				**<1**								
							0.110	0.110	0.116	**0.006**		**This study (avg.)**					<1							
1,1,1,3,3,3-Hexafluoro-2-(trifluoromethyl)-2-propanol	2378-02-01		$(CF_3)_3COH$				0.345	0.344	0.382		0.335	**(Imasu et al., 1995)**	I	O				296		700-1500	37.4			(1)
											0.380	H2013												
							0.345	0.344	**0.382**			**This study (avg.)**												
2,2,3,3,4,4,5,5-Octafluorocyclopentanol	16621-87-7		cyc (-$(CF_2)_4CH(OH)$-)	0.3	0.3	*WMO* (2019)	0.298	0.296	0.319	0.156	0.307	**(Imasu et al., 1995)**	I	O			14	296		700-1500	21.1			(1)
											0.160	H2013				13								
							0.298	0.296	0.319	**0.156**		**This study (avg.)**					**14**							
1-(Difluoromethoxy)-2-((difluoromethoxy)difluoromethoxy)-1,1,2,2-tetrafluoroethane	188690-77-9	HFE-43-10pccc124 (H-Galden 1040x, HG-11)	$CHF_2OCF_2OCF_2CF_2OCHF_2$	13.5	14.1	*WMO* (2019)	0.961	0.956	1.068	1.030	1.020	**(Wallington et al., 2009)**					3,380	296	933	700-1440	67.9			
							0.938	0.934	1.040	1.003	0.987	(Sihra et al., 2001)	A	S	LBL/NBM		3,300	296	933	450-1600	66.5			
											1.370	(Myhre et al., 1999)	A	O	LBL/BBM			295	986	963-1587	84.9	(Cavalli et al., 1998)		
							1.156	1.152	1.289	1.243		(Cavalli et al., 1998)					4,080	295	986	963-1587	84.9			
											1.109	(Christidis et al., 1997)	A	NC	LBL/NBM			296	921	450-2000	65.5			
											1.018	H2013				2,820								
							0.961	0.956	1.068	**1.030**		**This study (avg.)**					**3,380**							
1,1,1,2,2,3,3,4,4-Nonafluoro-4-methoxybutane	219484-64-7	HFE-449s1 (HFE-7100)	$C_4F_9OCH_3$	4.7	4.8	*WMO* (2019)	0.360	0.358	0.391	0.359	0.310	**(Bravo et al., 2010a)**	I	S	Pinnock		483	298		700-1400	34.4			
							0.361	0.360	0.391	0.360	0.347	**(Sihra et al., 2001)**	A	S	LBL/NBM		483	296	933	450-1550	36.0			
											0.370	(Wallington et al., 1997)	I	NC	Pinnock			295	933					
											0.364	H2013				421								
							0.360	0.359	0.391	**0.360**		**This study (avg.)**					**483**							
N/A	163702-07-6	n-HFE-7100	$CF_3CF_2CF_2CF_2OCH_3$	4.7	4.8	*WMO* (2019)	0.416	0.415	0.462	0.425	0.465	**(Sihra et al., 2001)**	A	NC	LBL/NBM		571	296	933	450-1520	33.7			
											0.420	H2013				486								
							0.416	0.415	0.462	**0.425**		**This study (avg.)**					**571**							
N/A	163702-08-7	i-HFE-7100	$(CF_3)_2CFCF_2OCH_3$	4.7	4.8	*WMO* (2019)	0.349	0.347	0.371	0.341	0.374	**(Sihra et al., 2001)**	A	NC	LBL/NBM		458	296	933	450-1500	37.6			
											0.352	H2013				407								
							0.349	0.347	0.371	**0.341**		**This study (avg.)**					**458**							
1-Ethoxy-1,1,2,2,3,3,4,4,4-nonafluorobutane	N/A	HFE-569sf2 (HFE-7200)	$C_4F_9OC_2H_5$	0.8	0.8	*WMO* (2019)	0.413	0.411	0.449	0.315	0.310	**(Bravo et al., 2010a)**	I	S	Pinnock		67	298		700-1400	38.5			
							0.378	0.376	0.409	0.287	0.303	**(Sihra et al., 2001)**	A	S	LBL/NBM		61	296	933	500-1500	37.1			
											0.390	(Christensen et al., 1998)	I	NC	Pinnock									
											0.305	H2013				57								
							0.396	0.394	0.429	**0.301**		**This study (avg.)**					**64**							
N/A	163702-06-5	i-HFE-7200	$(CF_3)_2CFCF_2OCH_2CH_3$	0.8	0.6	*WMO* (2019)	0.308	0.307	0.330	0.216	0.338	**(Sihra et al., 2001)**	A	NC	LBL/NBM		36	296	933	450-1400	32.7			
											0.238	H2013				44								
							0.308	0.307	0.330	**0.216**		**This study (avg.)**					**36**							

Table S13 (cont.)

				Lifetime (yr)			RE (W m^{-2} ppb^{-1}) – This study				RE (W m^{-2} ppb^{-1}) – Literature					GWP 100-yr		Absorption cross-sections						
Name	CASRN	Identifier	Formula	H2013	New	Reference	H2013 inst. RE	New inst. RE	New RE – const. profile	New RE – lifetime corr.	Value	Reference	Instantaneous/Adjusted	Vertical correction	RE calculation	H2013	New – lifetime corr.	*T* (K)	*p* (hPa)	Waveno. range (cm^{-1})	Int. abs. cross-section (10^{-17} cm^2 molecule^{-1})	Reference	Database	Notes
3-Methoxyperfluoro(2-methylpentane)	132182-92-4	HFE-7300	$(CF_3)_2CFCF(OC_2H_5)CF_2CF_2CF_3$		5.2	(Rodríguez et al., 2014)					0.480	(Rodríguez et al., 2014)		H	H2013			298		600-2000	46.1			
											**0.480**	**This study**					**425**							(3)
3-Ethoxyperfluoro(2-methylhexane)	297730-93-9	HFE-7500	n-$C_3F_7CF(OC_2H_5)CF(CF_3)_2$		0.3	(Rodríguez et al., 2014)					0.270	(Rodríguez et al., 2014)		H	H2013			298		600-2000	50.5			
											0.370	(Goto et al., 2002)			Pinnock					900-1900				
											**0.270**	**This study**					**14**							(3)
Bis(difluoromethoxy)difluoromethane	78522-47-1	HFE-236ca12 (HG-10)	$CHF_2OCF_2OCHF_2$	25.0	26.5	*WMO* (2019)	0.608	0.604	0.663	0.648	0.660	**(Myhre et al., 1999)**	A	O	LBL/BBM		6,370	298		25-3250	44.9			
												(Cavalli et al., 1998)						295	986	978-1584	51.9			
											0.653	H2013				5,350								
							0.608	0.604	0.663	**0.648**		**This study (avg.)**					**6,370**							
1,2-Bis(difluoromethoxy)-1,1,2,2-tetrafluoroethane	188690-78-0	HFE-338pcc13 (HG-01)	$CHF_2OCF_2CF_2OCHF_2$	12.9	13.4	*WMO* (2019)	0.811	0.807	0.904	0.870	0.870	**(Myhre et al., 1999)**	A	O	LBL/BBM		3,480	298		25-3250	54.7			
												(Cavalli et al., 1998)						295	986	930-1501	60.4			
											0.858	H2013				2,910								
							0.811	0.807	0.904	**0.870**		**This study (avg.)**					**3,480**							
1,1,1,3,3,3-Hexafluoropropan-2-ol	920-66-1	HFIP	$(CF_3)_2CHOH$	1.9	1.9	*WMO* (2019)	0.296	0.296	0.334	0.279	0.293	**(Godin et al., 2017a)**		H	H2013		221	300		530-3000	27.0		H16	
							0.284	0.284	0.321	0.268	0.294	**(Imasu et al., 1995)**	I	O			212	296		700-1500	25.4			(1)
											0.261	H2013				182								
							0.290	0.290	0.327	**0.274**		**This study (avg.)**					**216**							
1-(Difluoromethoxy)-2-(2-(difluoromethoxy)-1,1,2,2-tetrafluoroethoxy)-1,1,2,2-tetrafluoroethane	205367-61-9	HG-02	$CHF_2(OCF_2CF_2)_2OCHF_2$	26.0	26.9	*WMO* (2019)					1.070	(M P S Andersen et al., 2010b)	I	NC	Pinnock			295	933	650-1500	92.5			
											**1.150**	**H2013**				5,140								
											1.150	This study					**6,030**							(2,4)
1,1,3,3,4,4,6,6,7,7,9,9,10,10,12,12-Hexadecafluoro-2,5,8,11-tetraoxadodecane	173350-37-3	HG-03	$CHF_2(OCF_2CF_2)_3OCHF_2$	26.0	26.9	*WMO* (2019)					1.330	(M P S Andersen et al., 2010b)	I	NC	Pinnock			295	933	650-1500	85.2			
											**1.429**	**H2013**				4,800								
											1.429	This study					**5,630**							(2,4)
(2,2,2-Trifluoroethoxy)ethene	406-90-6	Fluroxene	$CF_3CH_2OCH=CH_2$	3.6 days	3.6 days	*WMO* (2019)					0.270	(Bravo et al., 2013)	I	NC	Pinnock			298			23.8			
											**0.011**	**H2013**				**<1**								
											0.011	This study (avg.)					<1							(5)
2-Ethoxy-3,3,4,4,5-pentafluorotetrahydro-2,5-bis[1,2,2,2-tetrafluoro-1-(trifluoromethyl)ethyl]-furan	920979-28-8		$C_{12}H_5F_{19}O_2$	1.0	0.8	*WMO* (2019)					0.600	(Javadi et al., 2007)	I	NC	Pinnock			294	933	700-1400	55.9			
											**0.489**	**H2013**				56								
											0.489	This study					**51**							(2)
Difluoro(methoxy)methane	359-15-9		CH_3OCHF_2	1.1	1.1	*WMO* (2019)	0.197	0.196	0.201	0.153		**(Orkin et al., 2014b)**					143	298		450-2000	14.4			
											0.174	H2013				144								
							0.197	0.196	0.201	**0.153**		**This study (avg.)**					**143**							
1,1,2,2-Tetrafluoro-1,2-dimethoxyethane	73287-23-7	HG'-01	$CH_3OCF_2CF_2OCH_3$	2.0	1.7	*WMO* (2019)	0.315	0.315	0.352	0.289	0.320	**(M P S Andersen et al., 2004)**	I	NC	Pinnock		212	296	933	200-1800	23.0			
											0.292	H2013				222								
							0.315	0.315	0.352	**0.289**		**This study (avg.)**					**212**							

Table S13 (cont.)

				Lifetime (yr)			RE (W m^{-2} ppb^{-1}) – This study				RE (W m^{-2} ppb^{-1}) – Literature					GWP 100-yr		Absorption cross-sections						
Name	CASRN	Identifier	Formula	H2013	New	Reference	H2013 inst. RE	New inst. RE	New RE – const. profile	New RE – lifetime corr.	Value	Reference	Instantaneous/Adjusted	Vertical correction	RE calculation	H2013	New – lifetime corr.	T (K)	p (hPa)	Waveno. range (cm^{-1})	Int. abs. cross-section (10^{-17} cm^2 molecule^{-1})	Reference	Database	Notes
1,1,2,2-Tetrafluoro-1-methoxy-2-(1,1,2,2-tetrafluoro-2-methoxyethoxy)ethane	485399-46-0	HG'-02	$CH_3O(CF_2CF_2O)_2CH_3$	2.0	1.7	*WMO* (2019)	0.609	0.608	0.683	0.562	0.610	**(M P S Andersen et al., 2004)**	I	NC	Pinnock		240	296	933	200-1800	45.5			
											0.564	H2013				250								
							0.609	0.608	0.683	**0.562**		**This study (avg.)**					**240**							
3,3,4,4,6,6,7,7,9,9,10,10-Dodecafluoro-2,5,8,11-tetraoxadodecane	485399-48-2	HG'-03	$CH_3O(CF_2CF_2O)_3CH_3$	2.0	1.7	*WMO* (2019)	0.826	0.825	0.927	0.762	0.830	**(M P S Andersen et al., 2004)**	I	NC	Pinnock		230	296	933	200-1800	63.4			
											0.765	H2013				239								
							0.826	0.825	0.927	**0.762**		**This study (avg.)**					**230**							
1,1,1,2,3,3-Hexafluoro-3-(trifluoromethoxy)propane	428454-68-6	HFE-329me3	$CF_3CFHCF_2OCF_3$	40.0	33.6	*WMO* (2019)	0.440	0.441	0.499	0.489	0.480	**(Wallington et al., 2004)**	I	NC	Pinnock		4,620	296	933	670-1450	42.4			
											0.476	H2013				4,550								
							0.440	0.441	0.499	**0.489**		**This study (avg.)**					**4,620**							
1-(Difluoromethoxy)-1,1,2,3,3,3-hexafluoropropane	56860-85-6	HFE-338mec3	$CF_3CFHCF_2OCF_2H$			(too large disagreement between studies)	0.460	0.458	0.510		0.485	**(Oyaro et al., 2005)**	I	NC	Pinnock			298		620-1450	34.2			
							0.476	0.474	0.528		0.510	**(Wallington et al., 2004)**	I	NC	Pinnock			296	933	680-1450	36.3			
											0.515	H2013												
							0.468	0.466	**0.519**			**This study (avg.)**												
3,3,4,4,5,5,6,6,7,7,7-Undecafluoroheptan-1-ol	185689-57-0		$CF_3(CF_2)_4CH_2CH_2OH$	20.0 days	17.0 days	*WMO* (2019)	0.338	0.337	0.371	0.054		**(Waterland et al., 2005)**					<1	296	933	670-1510	26.8			
											0.061	H2013				1								
							0.338	0.337	0.371	**0.054**		**This study (avg.)**					**<1**							
3,3,4,4,5,5,6,6,7,7,8,8,9,9,9-Pentadecafluorononan-1-ol	755-02-2		$CF_3(CF_2)_6CH_2CH_2OH$	20.0 days	17.0 days	*WMO* (2019)	0.374	0.373	0.412	0.060		**(Waterland et al., 2005)**					<1	296	933	600-1480	30.9			
											0.068	H2013				1								
							0.374	0.373	0.412	**0.060**		**This study (avg.)**					**<1**							
3,3,4,4,5,5,6,6,7,7,8,8,9,9,10,10,11,11,11-Nonadecafluoroundecan-1-ol	87017-97-8		$CF_3(CF_2)_8CH_2CH_2OH$	20.0 days	17.0 days	*WMO* (2019)	0.280	0.280	0.312	0.045		**(Waterland et al., 2005)**					<1	296	933	645-1450	24.7			
											0.051	H2013				**<1**								
							0.280	0.280	0.312	**0.045**		**This study (avg.)**					<1							
2-Chloro-1,1,2-trifluoro-1-methoxyethane	425-87-6		CH_3OCF_2CHClF	1.4	1.4	*WMO* (2019)	0.241	0.239	0.265	0.211	0.260	**(Dalmasso et al., 2006)**	I	NC	Pinnock		142	298		800-1500	17.0			
											**0.211**	**H2013**				122								
							0.241	0.239	0.265	0.211		This study (avg.)					**142**							
1-(Difluoro(trifluoromethoxy)methoxy)-1,1,2,3,3,3-hexafluoro-2-(trifluoromethoxy)propane	1309353-34-1	PFPMIE (perfluoropolymethylisopropyl)	$CF_3OCF(CF_3)CF_2OCF_2OCF_3$	800.0	800.0	*WMO* (2019)	0.601	0.600	0.653	0.640	0.650	**(Young et al., 2006)**	I	NC	Pinnock		10,900	296	933	650-1500	59.2			
											0.648	H2013				9,710								
							0.601	0.600	0.653	**0.640**		**This study (avg.)**					**10,900**							
1,1,2-Trifluoro-2-(trifluoromethoxy)ethene	1187-93-5	HFE-216	$CF_3OCF{=}CF_2$	8.4 days	1.6 days	*WMO* (2019)	0.275	0.278	0.317	0.006	0.280	**(Mashino et al., 2000)**	I	NC	Pinnock		<1	296	933	890-1865	29.8			
											0.025	H2013				**<1**								
							0.275	0.278	0.317	**0.006**		**This study (avg.)**					<1							
Perfluoroethyl formate	313064-40-3		CF_3CF_2OCHO	3.5	3.6	*WMO* (2019)					0.408	(Østerstrøm et al., 2016)		H	Pinnock			296		700-2000	33.1			
											0.442	H2013				580								
											**0.408**	**This study**					**626**							(6)
2,2,2-Trifluoroethyl formate	32042-38-9		CF_3CH_2OCHO	0.4	0.5	*WMO* (2019)					0.280	(Bravo et al., 2013)	I	NC	Pinnock			298			26.1			
											0.158	H2013				33								
											**0.192**	**This study (avg.)**					**57**							(5)

Table S13 (cont.)

Name	CASRN	Identifier	Formula	Lifetime (yr)			RE (W m^{-2} ppb^{-1}) – This study				RE (W m^{-2} ppb^{-1}) – Literature					GWP 100-yr		Absorption cross-sections						
				H2013	New	Reference	H2013 inst. RE	New inst. RE	New RE – const. profile	New RE – lifetime corr.	Value	Reference	Instantaneous/Adjusted	Vertical correction	RE calculation	H2013	New – lifetime corr.	*T* (K)	*p* (hPa)	Waveno. range (cm^{-1})	Int. abs. cross-section (10^{-17} cm^2 molecule^{-1})	Reference	Database	Notes
1,1,1,3,3,3-Hexafluoropropan-2-yl formate	856766-70-6		$(CF_3)_2CHOCHO$	3.2	3.1	*WMO* (2019)					0.255	(Østerstrøm et al., 2015)		H	Pinnock			296		1000-1800	3.2			
											0.333	H2013				334								
											**0.255**	**This study**					**282**							(7)
Vinyl 2,2,2-trifluoroacetate	433-28-3		$CF_3C(O)OCH{=}CH_2$		1.4 days	(Rodriguez et al., 2016)	0.230	0.230	0.261	0.004	0.004	**(Rodriguez et al., 2016)**	A	H	H2013		<1	298		600-2000	23.1		H16	
											0.387	H2013												
							0.230	0.230	0.261	**0.004**		**This study (avg.)**					**<1**							
Ethyl 2,2,2-trifluoroacetate	383-63-1		$CF_3C(O)OCH_2CH_3$	21.9 days	22.0 days	*WMO* (2019)	0.280	0.280	0.315	0.056		**(Sharpe et al., 2004)**					2	298	1013	500-3000	25.4		H16	
											0.053	H2013				1								
							0.280	0.280	0.315	**0.056**		**This study (avg.)**					**2**							
Allyl 2,2,2-trifluoroacetate	383-67-5		$CF_3C(O)OCH_2CH{=}CH_2$		1.2 days	(Rodriguez et al., 2016)	0.297	0.296	0.334	0.005	0.005	**(Rodriguez et al., 2016)**	A	H	H2013		<1	298		600-2000	23.7		H16	
											0.354	H2013												
							0.297	0.296	0.334	**0.005**		**This study (avg.)**					**<1**							
Methyl 2,2,2-trifluoroacetate	431-47-0		$CF_3C(O)OCH_3$	0.6	1.0	*WMO* (2019)					0.158	(Østerstrøm et al., 2015)		H	Pinnock			296		650-1900	1.7			
											0.179	H2013				52								
											**0.158**	**This study**					**86**							(7)
2,2,3,3,4,4,4-Heptafluorobutan-1-ol	375-01-9		$CF_3CF_2CF_2CH_2OH$	0.6	0.6	*WMO* (2019)	0.290	0.289	0.321	0.201	0.200	**(Bravo et al., 2010a)**	I	S	Pinnock		39	298		700-1400	21.4			
							0.286	0.285	0.315	0.197	0.295	**(Sellevåg et al., 2007)**	I	NC	Pinnock		38	298		450-1500	21.4			
											0.198	H2013				33								
							0.288	0.287	0.318	**0.199**		**This study (avg.)**					**38**							(8)
1,1,2-Trifluoro-2-(trifluoromethoxy)ethane	84011-06-3		$CHF_2CHFOCF_3$	9.8	9.0	*WMO* (2019)	0.329	0.330	0.371	0.353	0.349	**(Oyaro et al., 2005)**	I	NC	Pinnock		1,320	298		465-1600	28.2			
											0.345	H2013				1,240								
							0.329	0.330	0.371	**0.353**		**This study (avg.)**					**1,320**							
1-Ethoxy-1,1,2,3,3,3-hexafluoropropane	380-34-7		$CF_3CHFCF_2OCH_2CH_3$	0.4	0.4	*WMO* (2019)	0.318	0.317	0.347	0.193	0.331	**(Oyaro et al., 2005)**	I	NC	Pinnock		28	298		675-1450	27.4			
											0.191	H2013				23								
							0.318	0.317	0.347	**0.193**		**This study (avg.)**					**28**							
1,1,1,2,2,3,3-Heptafluoro-3-(1,2,2,2-tetrafluoroethoxy)propane	3330-15-2		$CF_3CF_2CF_2OCHFCF_3$	67.0	59.4	*WMO* (2019)	0.535	0.535	0.600	0.591	0.563	**(Oyaro et al., 2005)**	I	NC	Pinnock		7,000	298		500-1450	43.7			
											0.581	H2013				6,490								
							0.535	0.535	0.600	**0.591**		**This study (avg.)**					**7,000**							
2,2,3,3-Tetrafluoropropan-1-ol	76-37-9		$CHF_2CF_2CH_2OH$	91.2 days	93.0 days	*WMO* (2019)	0.233	0.231	0.257	0.116	0.230	**(Antiñolo et al., 2012b)**	I	NC	Pinnock		16	298		600-4000	19.2			
							0.221	0.219	0.241	0.109	0.203	**(Sellevåg et al., 2007)**	I	NC	Pinnock		15	298		505-1500	13.6			
											**0.112**	**H2013**				13								
							0.227	0.225	0.249	0.112		This study (avg.)					**15**							
2,2,3,4,4,4-Hexafluorobutan-1-ol	382-31-0		$CF_3CHFCF_2CH_2OH$	94.9 days	0.4	*WMO* (2019)	0.387	0.385	0.424	0.227	0.370	**(Sellevåg et al., 2007)**	I	NC	Pinnock		32	298		450-1500	29.2			
											0.194	H2013				17								
							0.387	0.385	0.424	**0.227**		**This study (avg.)**					**32**							
1,1,2,2-Tetrafluoro-3-methoxypropane	60598-17-6		$CHF_2CF_2CH_2OCH_3$	14.2 days	26.0 days	*WMO* (2019)	0.231	0.229	0.256	0.052	0.237	**(Oyaro et al., 2004)**	I	NC	Pinnock		2	298		500-1520	13.9			
											0.032	H2013				1								
							0.231	0.229	0.256	**0.052**		**This study (avg.)**					**2**							
1,1,1,2,2,4,5,5,5-Nonafluoro-4-(trifluoromethyl)-3-pentanone	756-13-8	perfluoro-2-methylpentan-3-one	$CF_3CF_2C(O)CF(CF_3)_2$	7.0 days	7.0 days	*WMO* (2019)						(Ren et al., 2019)						298		480-1880	40.5			
							0.370	0.371	0.407	0.028		**(D'Anna et al., 2005)**					<1	298		480-1880	40.5			
											**0.028**	**H2013**				**<1**								
							0.370	0.371	0.407	0.028		This study (avg.)					<1							

Table S13 (cont.)

Name	CASRN	Identifier	Formula	Lifetime (yr) H2013	Lifetime (yr) New	Lifetime (yr) Reference	RE ($W m^{-2} ppb^{-1}$) – This study: H2013 inst. RE	New inst. RE	New RE – const. profile	New RE – lifetime corr.	RE ($W m^{-2} ppb^{-1}$) – Literature: Value	Reference	Instantaneous/Adjusted	Vertical correction	RE calculation	GWP 100-yr: H2013	New – lifetime corr.	Absorption cross-sections: *T* (K)	*p* (hPa)	Waveno. range (cm^{-1})	Int. abs. cross-section (10^{-17} cm^2 $molecule^{-1}$)	Reference	Database	Notes
3,3,3-Trifluoropropanal	460-40-2		CF_3CH_2CHO	2.0 days	3.0 days	*WMO* (2019)	0.156	0.155	0.173	0.006		**(Antiñolo et al., 2011)**					<1	298		600-1850	16.7			
							0.129	0.129	0.143	0.005		**(Sellevag et al., 2004a)**					<1	298		400-1900	14.2			
											0.004	H2013				**<1**								
							0.142	0.142	0.158	**0.005**		**This study (avg.)**					<1							
4,4,4-Trifluorobutanal	406-87-1		$CF_3CH_2CH_2CHO$				0.149	0.148	0.163			**(Antiñolo et al., 2012a)**								1000-1850	17.0			
											**0.163**	**H2013**												
							0.149	0.148	0.163			This study (avg.)												
2-Fluoroethanol	371-62-0		CH_2FCH_2OH	20.4 days	16.0 days	*WMO* (2019)	0.089	0.088	0.087	0.012	0.016	**(Sellevag et al., 2004b)**	A	M	BBM		<1	293		100-1600	6.2			
							0.079	0.078	0.081	0.011		**(Sharpe et al., 2004)**					<1	298	1013	510-3000	6.7		H16	
											0.016	H2013				1								
							0.084	0.083	0.084	**0.012**		**This study (avg.)**					**<1**							
2,2-Difluoroethanol	359-13-7		CHF_2CH_2OH	40.0 days	61.0 days	*WMO* (2019)	0.123	0.122	0.127	0.046	0.020	**(Sellevag et al., 2004b)**	A	M	BBM		6	293		100-1600	8.0			
											0.037	H2013				3								
							0.123	0.122	0.127	**0.046**		**This study (avg.)**					**6**							
2,2,2-Trifluoroethanol	75-89-8		CF_3CH_2OH	0.3	0.5	*WMO* (2019)	0.186	0.186	0.202	0.118	0.085	**(Sellevag et al., 2004b)**	A	M	BBM		38	293		100-1600	15.9			
							0.179	0.178	0.199	0.117		**(Sharpe et al., 2004)**					37	298	1013	500-3000	15.1		H16	
							0.179	0.178	0.200	0.117	0.190	**(Imasu et al., 1995)**	I	O			37	296		700-1500	14.3			(1)
											0.101	H2013				20								
							0.182	0.181	0.200	**0.117**		**This study (avg.)**					**37**							
1,1,3,3,4,4,6,6,7,7,9,9,10,10,12,12,13,13,15,15-eicosafluoro-2,5,8,11,14-Pentaoxapentadecane	173350-38-4	HG-04	$CHF_2O(CF_2CF_2O)_4CHF_2$	26.0	26.9	*WMO* (2019)					1.360	(M P S Andersen et al., 2010b)	I	NC	Pinnock			295	933	650-1500	70.2			
											**1.461**	**H2013**				3,930								
											1.461	This study					**4,610**							(2)
Methyl-perfluoroheptene-ethers			$CH_3OC_7F_{13}$		0.3	(Jubb et al., 2014)					0.550	(Jubb et al., 2014)	A	NC	H2013			296		600-1600				(9)
											**0.270**	**This study**					**16**							(10)
1,1,1-Trifluoropropan-2-one	421-50-1		$CF_3C(O)CH_3$		5.1 days	(Diaz-de-Mera et al., 2015)					0.010	(Diaz-de-Mera et al., 2015)		H	H2013			298		600-2000	8.3			
							0.184	0.184	0.205	0.011		**(Sharpe et al., 2004)**					<1	296		530-3000	15.3		P	
							0.184	0.184	0.205	**0.011**		**This study (avg.)**					**<1**							
1,1,1-Trifluorobutan-2-one	381-88-4		$CF_3C(O)CH_2CH_3$		6.6 days	(Diaz-de-Mera et al., 2015)					0.010	(Diaz-de-Mera et al., 2015)		H	H2013			298		600-2000	12.7			
											**0.010**	**This study**					**<1**							(11)
2,2,2-Trifluoroethanal	75-90-1		CF_3CHO				0.149	0.149	**0.167**			**(Sellevag et al., 2004a)**						298		400-2500	13.3		H16	
2,2,3,3,3-Pentafluoropropanal	422-06-0		CF_3CF_2CHO				0.179	0.180	**0.202**			**(Hashikawa et al., 2004)**						296	933	600-2500	16.0		H16	
2,2,3,3,4,4,4-Heptafluorobutanal	375-02-0		$CF_3CF_2CF_2CHO$				0.225	0.225	**0.250**			**(Hashikawa et al., 2004)**						296	933	600-2500	19.8		H16	
2,2,3,3,4,4,5,5,5-Nonafluoropentanal	375-53-1		$CF_3CF_2CF_2CF_2CHO$				0.255	0.255	**0.286**			**(Hashikawa et al., 2004)**						296	933	600-2500	22.8		H16	
2-Propenoyl chloride	814-68-6	Acryloyl chloride	CH_2=CHC(O)Cl				0.136	0.133	**0.146**			**(Sharpe et al., 2004)**						296		510-3000	11.4		P	
Acetyl chloride	75-36-5		CH_3COCl				0.103	0.102	**0.108**			**(Sharpe et al., 2004)**						296		550-3000	10.7		P	
1-chloro-2-ethoxyethane	628-34-2		C_4H_9ClO				0.091	0.090	**0.099**			**(Sharpe et al., 2004)**						296		600-3000	9.9		P	
2-Chloroethanol	107-07-3		CH_2ClCH_2OH				0.054	0.053	**0.057**			**(Sharpe et al., 2004)**						296		600-3000	5.5		P	
2-(chloromethyl)oxirane	106-89-8		C_3H_5ClO				0.048	0.047	**0.052**			**(Sharpe et al., 2004)**						296		550-3000	3.0		P	
1-Chloropropan-2-one	78-95-5		$CH_3C(O)CH_2Cl$				0.035	0.035	**0.038**			**(Sharpe et al., 2004)**						298	1013	600-3000	6.0		H16	
1-chloro-2-(2-chloroethoxy)ethane	111-44-4		$CH_2ClCH_2OCH_2CH_2Cl$				0.106	0.105	**0.115**			**(Sharpe et al., 2004)**						298	1013	550-3000	9.6		H16	
2-chloroethyl vinyl ether	110-75-8		$ClCH_2CH_2OCH=CH_2$		0.1 days	(Antiñolo et al., 2017)						(Antiñolo et al., 2017)			H2013			298		500-1500	7.8			
											**<0.001**	**This study**					**<1**							(12)

Table S13 (cont.)

				Lifetime (yr)			RE (W m^{-2} ppb^{-1}) – This study				RE (W m^{-2} ppb^{-1}) – Literature					GWP 100-yr		Absorption cross-sections						
Name	CASRN	Identifier	Formula	H2013	New	Reference	H2013 inst. RE	New inst. RE	New RE – const. profile	New RE – lifetime corr.	Value	Reference	Instantaneous/Adjusted	Vertical correction	RE calculation	H2013	New – lifetime corr.	T (K)	p (hPa)	Waveno. range (cm^{-1})	Int. abs. cross-section (10^{-17} cm^2 molecule^{-1}	Reference	Database	Notes
(Chlorometoxy)ethane	3188-13-4		$CH_3CH_2OCH_2Cl$				0.096	0.095	**0.106**			**(Sharpe et al., 2004)**						296		560-3000	10.3		P	
Chloro(methoxy)methane	107-30-2		CH_3OCH_2Cl				0.083	0.082	**0.092**			**(Sharpe et al., 2004)**						296		560-3000	8.8		P	
Ethyl carbonochloridate	541-41-3		$CH_3CH_2OC(O)Cl$				0.231	0.229	**0.257**			**(Sharpe et al., 2004)**						296		530-3000	20.1		P	
1-Fluoropropan-2-one	430-51-3		$CH_3C(O)CH_2F$				0.045	0.045	**0.046**			**(Sharpe et al., 2004)**						296		520-3000	6.6		P	
1,1,1,3,3,3-hexafluoropropan-2-one	684-16-2		$CF_3C(O)CF_3$				0.257	0.256	**0.289**			**(Sharpe et al., 2004)**						296		525-3000	24.6		P	
Trifluoroacetic acid	76-05-1		$CF_3C(O)OH$				0.322	0.321	**0.359**			**(Sharpe et al., 2004)**						296		540-3000	36.2		P	
Trifluoroacetic anhydride	407-25-0		$CF_3C(O)OC(O)CF_3$				0.503	0.500	**0.507**			**(Sharpe et al., 2004)**						296		500-3000	45.3		P	
Methacryloyl chloride	920-46-7		$CH_2{=}C(CH_3)C(O)Cl$				0.109	0.107	**0.116**			**(Sharpe et al., 2004)**						296		530-3000	9.9		P	

(1) Scaled to the CFC-11 RE of 0.26 W m^{-2} ppb^{-1}; (2) RE value is retained from H2013, but the GWP value is updated to reflect possible changes in lifetime and $AGWP_{CO2}$; (3) RE value is from (Rodríguez et al., 2014) with lifetime correction included; (4) In H2013 this compound was accidently listed twice with the same CASRN but with different values for lifetime, RE and GWP(100). The discussion and results presented in section 4.1.7.52 of H2013, based on (M P S Andersen et al., 2010b), are correct; (5) RE value is from (Bravo et al., 2013) except that stratospheric temperature adjustment (10% increase of inst. RE) and lifetime correction factors have been applied; (6) RE value is from (Østerstrøm et al., 2016); (7) RE value is from (Østerstrøm et al., 2015); (8) In H2013 this compound was accidently listed twice with the same CASRN but with different values for lifetime, RE and GWP(100). The discussion and results presented in sections 4.1.7.41 and 4.1.7.44 of H2013 should have been merged to include results from both (Bravo et al., 2010a) and (Sellevåg et al., 2007); (9) 6 different isomers where all have the same RE of 0.55 W m^{-2} ppb^{-1} before lifetime correction. They include scaling factors to account for nonuniform atmospheric mixing (0.042 to 0.49). The lifetime ranges from 4.0 to 111 days depending on the isomer – the upper value is listed here; (10) RE value is from (Jubb et al., 2014) but lifetime-corrected using the value for the isomer with the longest lifetime (111 days); (11) RE value is from (Diaz-de-Mera et al., 2015); (12) Based on the GWP(20) value reported in (Antiñolo et al., 2017) (they do not report the RE) it is likely that the lifetime-corrected RE is less than 0.001 W m^{-2} ppb^{-1}

Table S14. Hydrocarbons

Please refer to page 2 of this document for a description of the table.

				Lifetime (yr)			RE (W m^{-2} ppb^{-1}) – This study				RE (W m^{-2} ppb^{-1}) – Literature					GWP 100-yr		Absorption cross-sections						
Name	CASRN	Identifier	Formula	H2013	New	Reference	H2013 inst. RE	New inst. RE	New RE – const. profile	New RE – lifetime corr.	Value	Reference	Instantaneous/Adjusted	Vertical correction	RE calculation	H2013	New – lifetime corr.	*T* (K)	*p* (hPa)	Waveno. range (cm^{-1})	Int. abs. cross-section (10^{-17} cm^2 molecule^{-1})	Reference	Database	Notes
Ethane	74-84-0		C_2H_6		58.0 days	(Hodnebrog et al., 2018)					0.001	(Hodnebrog et al., 2018)	A	H	H2013			298		600-2500	0.4	(Sharpe et al., 2004)		
							<0.001	<0.001	<0.001	<0.001		(Harrison et al., 2010)					<1	296	495	2545-3000	2.3		H16	
							0.003	0.003	0.004	0.001		**(Sharpe et al., 2004)**					<1	296		600-3000	2.6		P	
											0.003	(Sihra et al., 2001)	A	NC	NBM			296		450-2000	0.4			
											0.003	(Highwood et al., 1999)	A	NC	NBM			296		450-2000	0.4			
							0.003	0.003	0.004	**0.001**		**This study (avg.)**					**<1**							
Benzene	71-43-2		C_6H_6				0.003	0.003	**0.003**			**(Rinsland et al., 2008)**						298	1013	600-3000	2.4		H16	
Methylbenzene	108-88-3	Toulene	$C_6H_5(CH_3)$				0.015	0.015	**0.014**			**(Sharpe et al., 2004)**						298	1013	600-3000	3.1		H16	
1,2-Dimethylbenzene	95-47-6	o-Xylene	o-$C_6H_4(CH_3)_2$				0.019	0.019	**0.019**			**(Sharpe et al., 2004)**						298	1013	600-3000	4.3		H16	
1,3-Dimethylbenzene	108-38-3	m-Xylene	m-$C_6H_4(CH_3)_2$				0.024	0.024	**0.026**			**(Sharpe et al., 2004)**						298	1013	580-3000	4.4		H16	
1,4-Dimethylbenzene	106-42-3	p-Xylene	p-$C_6H_4(CH_3)_2$				0.024	0.024	**0.026**			**(Sharpe et al., 2004)**						298	1013	580-3000	4.1		H16	
Propane	74-98-6		C_3H_8		13.0 days	(Hodnebrog et al., 2018)					<0.001	(Hodnebrog et al., 2018)	A	H	H2013			298		600-2500	0.6	(Sharpe et al., 2004)		
							<0.001	<0.001	<0.001	<0.001		(Harrison and Bernath, 2010)					<1	296	1017	2540-3000	4.0		H16	
							0.003	0.003	0.003	<0.001		**(Sharpe et al., 2004)**					<1	298	1013	600-3000	4.5		H16	
											0.003	(Sihra et al., 2001)	A	NC	NBM			296		450-2000	0.5			
											0.003	(Highwood et al., 1999)	A	NC	NBM			296		450-2000	0.5			
							0.003	0.003	0.003	**<0.001**		**This study (avg.)**					**<1**							
Butane	106-97-8		n-C_4H_{10}		6.8 days	(Hodnebrog et al., 2018)					<0.001	(Hodnebrog et al., 2018)	A	H	H2013			298		600-2500	0.7	(Sharpe et al., 2004)		
							0.004	0.004	0.004	<0.001		**(Sharpe et al., 2004)**					<1	298	1013	600-3000	5.8		H16	
											0.004	(Sihra et al., 2001)	A	NC	NBM			296		450-2000	0.6			
											0.005	(Highwood et al., 1999)	A	NC	NBM			296		450-2000	0.7			
							0.004	0.004	0.004	**<0.001**		**This study (avg.)**					**<1**							
2-Methyløpropane	75-28-5	Isobutane	i- C_4H_{10}				0.003	0.003	**0.004**			**(Sharpe et al., 2004)**						278	1013	600-3000	5.9		H16	
1-Butene	106-98-9		$CH_3CH_2CH=CH_2$				0.028	0.028	**0.030**			**(Sharpe et al., 2004)**						298	1013	525-3000	4.1		H16	
2-Methyl-1-propene	115-11-7	Isobutene	$(CH_3)_2C=CH_2$				0.021	0.021	**0.023**			**(Sharpe et al., 2004)**						278	1013	600-3000	4.1		H16	
1-Nonene	124-11-8		n-$C_7H_{15}CH=CH_2$				0.028	0.027	**0.030**			**(Sharpe et al., 2004)**						298	1013	550-3000	9.5		H16	
(1*S*,5*S*)-2,6,6-trimethylbicyclo[3.1.1]hept-2-ene	7785-26-4	α-pinene	$C_{10}H_{16}$ #				0.018	0.018	**0.019**			**(Sharpe et al., 2004)**						298	1013	600-3000	8.9		H16	
(1*S*,5*S*)-6,6-dimethyl-2-methylenebicyclo[3.1.1]heptane	18172-67-3	β-pinene	$C_{10}H_{16}$ #				0.027	0.026	**0.029**			**(Sharpe et al., 2004)**						298	1013	600-3000	9.4		H16	
(4R)-1-methyl-4-prop-1-en-2-ylcyclohexene	5989-27-5	D-Limonene	C10H16#				0.035	0.035	**0.037**			**(Johnson et al., 2010)**						298	1013	520-3000	8.4		H16	
1-Methyl-4-(1-methylethenyl)-cyclohexene	138-86-3	DL-Limonene	$C_{10}H_{16}$ #				0.021	0.021	**0.023**			**(Johnson et al., 2010)**						298	1013	580-3000	8.3		H16	
3,7,7-Trimethyl-bicyclo[4.1.0]hept-2-ene	554-61-0	2-Carene	$C_{10}H_{16}$ #				0.020	0.020	**0.022**			**(Johnson et al., 2010)**						298	1013	600-3000	8.5		H16	
3,7,7-Trimethyl-bicyclo[4.1.0]hept-3-ene	13466-78-9	3-Carene	$C_{10}H_{16}$ #				0.018	0.017	**0.019**			**(Johnson et al., 2010)**						298	1013	600-3000	8.4		H16	
7-Methyl-3-methylene-1,6-octadiene	123-35-3	Myrcene	$C_{10}H_{16}$ #				0.050	0.049	**0.054**			**(Johnson et al., 2010)**						298	1013	520-3000	7.0		H16	
1-Ethyl-2-methylbenzene		o-Ethyltoluene	C_9H_{12} #				0.025	0.025	**0.026**			**(Sharpe et al., 2004)**						298	1013	520-3000	5.6		H16	
1-Ethyl-4-methylbenzene	622-96-8	p-Ethyltoluene	C_9H_{12} #				0.026	0.025	**0.027**			**(Sharpe et al., 2004)**						298	1013	600-3000	5.5		H16	
1,3-Butadiene	106-99-0		$CH_2=CHCH=CH_2$				0.047	0.046	**0.049**			**(Sharpe et al., 2004)**						298	1013	530-3000	2.8		H16	
Pentane	109-66-0		n-C_5H_{12}				0.005	0.005	**0.005**			**(Sharpe et al., 2004)**						298	1013	570-3000	7.2		H16	
2-Methylbutane	78-78-4	Isopentane	$(CH_3)_2CHCH_2CH_3$				0.005	0.005	**0.006**			**(Sharpe et al., 2004)**						298	1013	600-3000	7.0		H16	
2-Methyl-1,3-butadiene	78-79-5		$CH_2=C(CH_3)CH=CH_2$				0.042	0.041	**0.046**			**(Sharpe et al., 2004)**						278	1013	600-3000	3.7		H16	
Heptane	142-82-5		n-C_7H_{16}				0.006	0.006	**0.006**			**(Sharpe et al., 2004)**						298	1013	600-3000	9.3		H16	
Naphtalene	91-20-3		$C_{10}H_8$ #				0.057	0.056	**0.060**			**(Sharpe et al., 2004)**						323	1013	600-3000	3.0		H16	
Octane	111-65-9		n-C_8H_{18}				0.007	0.007	**0.007**			**(Sharpe et al., 2004)**						298	1013	600-3000	10.5		H16	
Ethenylbenzene	100-42-5	Styrene	$C_6H_5CH=CH_2$				0.044	0.043	**0.046**			**(Sharpe et al., 2004)**						298	1013	600-3000	3.4		H16	
Decane	124-18-5		n-$C_{10}H_{22}$				0.008	0.008	**0.009**			**(Sharpe et al., 2004)**						296		580-3000	13.2		P	
Hexadecane	544-76-3		n-$C_{16}H_{34}$				0.027	0.027	**0.029**			**(Johnson et al., 2010)**						323	1013	550-3000	27.2		H16	
Pentadecane	629-62-9		n-$C_{15}H_{32}$				0.031	0.031	**0.032**			**(Johnson et al., 2010)**						298	1013	570-3000	20.5		H16	

Table S14 (cont.)

Name	CASRN	Identifier	Formula	Lifetime (yr) H2013	Lifetime (yr) New	Lifetime (yr) Reference	RE ($W m^{-2} ppb^{-1}$) – This study: H2013 inst. RE	New inst. RE	New RE – const. profile	New RE – lifetime corr.	RE ($W m^{-2} ppb^{-1}$) – Literature: Value	Reference	Instantaneous/Adjusted	Vertical correction	RE calculation	GWP 100-yr: H2013	New – lifetime corr.	Absorption cross-sections: *T* (K)	*p* (hPa)	Waveno. range (cm^{-1})	Int. abs. cross-section (10^{-17} cm^2 $molecule^{-1}$)	Reference	Database	Notes
1-ethenyl-3-methylbenzene	100-80-1	m-vinyl toluene	$CH_3C_6H_4CH=CH_2$				0.045	0.044	**0.048**			**(Johnson et al., 2010)**						298	1013	550-3000	4.2		H16	
1,2-Propadiene	463-49-0	Allene	$CH_2=C=CH_2$				0.048	0.047	**0.052**			**(Sharpe et al., 2004)**						296		550-3000	2.9		P	
Ethyne	74-86-2-	Acetylene	C_2H_2				0.041	0.041	**0.041**			**(Sharpe et al., 2004)**						296		600-3000	3.4		P	
4-Vinylcyclohexene	100-40-3		C_8H_{12} #				0.032	0.031	**0.034**			**(Sharpe et al., 2004)**						296		590-3000	6.1		P	
4-Methyl-1-pentene	691-37-2		$(CH_3)_2CHCH_2CH=CH_2$				0.030	0.029	**0.032**			**(Sharpe et al., 2004)**						296		575-3000	6.6		P	
3-Methylpentane	96-14-0		$CH_3CH_2CH(CH_3)CH_2CH_3$				0.008	0.007	**0.008**			**(Sharpe et al., 2004)**						296		600-3000	8.1		P	
3-Methylhexane	589-34-4		$CH_3CH_2CH(CH_3)CH_2CH_2CH_3$				0.008	0.008	**0.009**			**(Sharpe et al., 2004)**						296		530-3000	9.2		P	
3-Methyl-1-butene	563-45-1		$(CH_3)_2CHCH=CH_2$				0.027	0.026	**0.029**			**(Sharpe et al., 2004)**						296		600-3000	5.3		P	
1-Ethyl-3-methylbenzene	620-14-4	m-Ethyltoluene	C_9H_{12} #				0.025	0.024	**0.026**			**(Sharpe et al., 2004)**						296		600-3000	5.9		P	
2,4,4-Trimethyl-1-pentene	107-39-1		$(CH_3)_3CCH_2C(CH_3)=CH_2$				0.027	0.027	**0.029**			**(Sharpe et al., 2004)**						296		530-3000	9.1		P	
2,3-Dimethylbutane	79-29-8	diisopropyl	$(CH_3)_2CHCH(CH_3)_2$				0.008	0.008	**0.008**			**(Sharpe et al., 2004)**						296		510-3000	8.0		P	
2,2,4-Trimethyl-2-pentene	107-40-4		$(CH_3)_3CCH=C(CH_3)_2$				0.019	0.019	**0.021**			**(Sharpe et al., 2004)**						296		540-3000	8.9		P	
2,2-Dimethylbutane	75-83-2	Neohexane	$(CH_3)_3CCH_2CH_3$				0.007	0.007	**0.007**			**(Sharpe et al., 2004)**						296		600-3000	8.2		P	
2-Methyl-1-pentene	763-29-1		$CH_3CH_2CH_2C(CH_3)=CH_2$				0.024	0.024	**0.026**			**(Sharpe et al., 2004)**						296		500-3000	6.4		P	
2-Methyl-1-butene	563-46-2		$CH_3CH_2C(CH_3)=CH_2$				0.025	0.024	**0.027**			**(Sharpe et al., 2004)**						296		600-3000	5.5		P	
2-Butene	107-01-7		$CH_3CH=CHCH_3$				0.015	0.014	**0.016**			**(Sharpe et al., 2004)**						296		550-3000	3.7		P	
1,2,3,5-Tetramethylbenzene	527-53-7		$C_{10}H_{14}$ #				0.019	0.019	**0.020**			**(Sharpe et al., 2004)**						296		600-3000	6.7		P	
1,2,3,4-Tetramethylbenzene	488-23-3		$C_{10}H_{14}$ #				0.021	0.021	**0.022**			**(Sharpe et al., 2004)**						296		600-3000	6.4		P	
1,2,3,4-Tetrahydronaphthalene	119-64-2	Tetralin	$C_{10}H_{12}$ #				0.022	0.022	**0.023**			**(Sharpe et al., 2004)**						296		550-3000	6.8		P	
1-Pentene	109-67-1		n-$C_3H_7CH=CH_2$				0.021	0.020	**0.022**			**(Sharpe et al., 2004)**						296		525-3000	3.9		P	
1-Octene	111-66-0		n-$C_6H_{13}CH=CH_2$				0.028	0.027	**0.030**			**(Sharpe et al., 2004)**						296		530-3000	8.6		P	
1-Hexene	592-41-6		n-$C_4H_9CH=CH_2$				0.029	0.028	**0.031**			**(Sharpe et al., 2004)**						296		520-3000	6.5		P	
1-Butyne	107-00-6		$CH_3CH_2C≡CH$				0.011	0.011	**0.011**			**(Sharpe et al., 2004)**						296		500-3000	3.7		P	
Hexane	110-54-3		n-C_6H_{14}				0.005	0.005	**0.005**			**(Sharpe et al., 2004)**						298	1013	600-3000	8.1		H16	
1-Heptene	25339-56-4	n-Heptene	n-C_7H_{16}				0.030	0.029	**0.031**			**(Sharpe et al., 2004)**						296		590-3000	7.7		P	
Nonane	111-84-2		n-C_9H_{20}				0.007	0.007	**0.008**			**(Sharpe et al., 2004)**						296		580-3000	12.0		P	
Tridecane	629-50-5		n-$C_{13}H_{28}$				0.012	0.012	**0.013**			**(Sharpe et al., 2004)**						296		600-3000	16.0		P	
Undecane	1120-21-4		n-$C_{11}H_{24}$				0.009	0.009	**0.009**			**(Sharpe et al., 2004)**						296		580-3000	14.4		P	
2-Pentene (Z)	627-20-3		(Z)-$CH_3CH=CHCH_2CH_3$				0.012	0.012	**0.012**			**(Sharpe et al., 2004)**						296		535-3000	4.8		P	
4-Methyl-2-pentene (Z)	691-38-3		(Z)-$(CH_3)_2CHCH=CHCH_3$				0.017	0.017	**0.017**			**(Sharpe et al., 2004)**						296		560-3000	6.2		P	
(1-Methylethyl)benzine	98-82-8	Cumene	$C_6H_5CH(CH_3)_2$				0.021	0.021	**0.021**			**(Sharpe et al., 2004)**						296		600-3000	5.8		P	
Cyclodecane	293-96-9		cyc $C_{10}H_{20}$				0.008	0.008	**0.009**			**(Sharpe et al., 2004)**						296		600-3000	12.9		P	
Cycloheptane	291-64-5		cyc C_7H_{14}				0.006	0.006	**0.006**			**(Sharpe et al., 2004)**						296		600-3000	9.6		P	
Cycloheptene	628-92-2		cyc C_7H_{12}				0.012	0.012	**0.013**			**(Sharpe et al., 2004)**						296		565-3000	7.3		P	
Cyclohexene	110-83-8		cyc C_6H_{10}				0.011	0.011	**0.012**			**(Sharpe et al., 2004)**						296		575-3000	6.1		P	
Cyclooctane	292-64-8		cyc C_8H_{16}				0.008	0.008	**0.009**			**(Sharpe et al., 2004)**						296		600-3000	10.7		P	
Cyclopentane	287-92-3		cyc C_5H_{10}				0.004	0.004	**0.004**			**(Sharpe et al., 2004)**						296		510-3000	6.1		P	
Cyclopentene	142-29-0		cyc C_5H_8				0.013	0.013	**0.013**			**(Sharpe et al., 2004)**						296		550-3000	4.9		P	
Cyclopropane	75-19-4		cyc C_3H_6				0.023	0.022	**0.023**			**(Sharpe et al., 2004)**						296		600-3000	1.3		P	
Ethyl benzene	100-41-4		$C_6H_5CH_2CH_3$				0.022	0.021	**0.022**			**(Sharpe et al., 2004)**						296		520-3000	4.7		P	
Ethene	74-85-1	Ethylene	$CH=CH_2$				0.037	0.036	**0.039**			**(Sharpe et al., 2004)**						296		600-3000	1.9		P	
Propylbenzene	103-65-1	Isocumene	$C_6H_5CH_2CH_2$ CH_3				0.017	0.017	**0.017**			**(Sharpe et al., 2004)**						296		600-3000	5.7		P	
2,2,4-trimethylpentane	540-84-1	Isooctane	$(CH_3)_2CHCH_2C(CH_3)_3$				0.009	0.009	**0.010**			**(Sharpe et al., 2004)**						296		600-3000	10.5		P	
Propyne	74-99-7	Methyl acetylene	$CH_3C≡CH$				0.008	0.008	**0.008**			**(Sharpe et al., 2004)**						296		560-3000	2.6		P	
(1-Methylpropyl)benzene	135-98-8	sec-Butylbenzene	$C_6H_5CH(CH_3)CH_2CH_3$				0.022	0.021	**0.022**			**(Sharpe et al., 2004)**						296		600-3000	7.0		P	
(1,1-dimethylethyl)benzene	98-06-6	tert-Butylbenzene	$C_6H_5C(CH_3)_3$				0.028	0.028	**0.029**			**(Sharpe et al., 2004)**						296		520-3000	7.6		P	
2-Pentene (E)	646-04-8		(E)-$(CH_3)_2CHCH=CHCH_3$				0.023	0.022	**0.024**			**(Sharpe et al., 2004)**						296		550-3000	5.4		P	

Table S15. Alcohols, ethers and other oxygenated hydrocarbons

Please refer to page 2 of this document for a description of the table.

				Lifetime (yr)			RE (W m^{-2} ppb^{-1}) – This study				RE (W m^{-2} ppb^{-1}) – Literature					GWP 100-yr		Absorption cross-sections						
Name	CASRN	Identifier	Formula	H2013	New	Reference	H2013 inst. RE	New inst. RE	New RE – const. profile	New RE – lifetime corr.	Value	Reference	Instantaneous/Adjusted	Vertical correction	RE calculation	H2013	New – lifetime corr.	*T* (K)	*p* (hPa)	Waveno. range (cm^{-1})	Int. abs. cross-section (10^{-17} cm^2 molecule^{-1})	Reference	Database	Notes
2,4,6-Trimethyl-1,3,5-Trioxane	123-63-7	Paraldehyde	$C_6H_{12}O_3$				0.239	0.237	**0.267**			**(Sharpe et al., 2004)**						296		510-3000	21.4		P	
Cyclohexanol	108-93-0		cyc-C_6H_{11}-OH				0.056	0.056	**0.060**			**(Sharpe et al., 2004)**						296		600-3000	10.8		P	
Cyclohexanone	108-94-1		cyc-C_6H_{10}=O				0.022	0.022	**0.024**			**(Sharpe et al., 2004)**						296		540-3000	9.2		P	
Acetone	67-64-1		$CH_3C(O)CH_3$				0.024	0.024	**0.027**			**(Harrison et al., 2011)**						296	1012	700-1780	5.5		H16	
Acetaldehyde	75-07-0		CH_3CHO				<0.001<br>0.016<br>0.016	<0.001<br>0.016<br>0.016	<0.001<br>0.017<br>**0.017**			(Tereszchuk and Bernath, 2011)<br>**(Sharpe et al., 2004)**<br>**This study (avg.)**						298<br>298	1013<br>1013	2400-3000<br>510-3000	2.0<br>5.9		H16<br>H16	
1-Penten-3-ol	616-25-1		CH_2=$CHCHOHCH_2CH_3$				0.072	0.071	**0.076**			**(Johnson et al., 2010)**						298	1013	550-3000	7.3		H16	
3-Methylbutanal	590-86-3	Isovaleraldehyde	$(CH_3)_2CHCH_2CHO$				0.022	0.021	**0.022**			**(Johnson et al., 2010)**						278	1013	500-3000	9.3		H16	
2-Pentanone	107-87-9	Methyl propyl ketone	$CH_3C(O)CH_2CH_2CH_3$				0.033<br>0.031<br>0.032	0.033<br>0.031<br>0.032	0.037<br>0.034<br>**0.036**			**(Johnson et al., 2010)**<br>**(Sharpe et al., 2004)**<br>**This study (avg.)**						298<br>278	1013<br>1013	520-3000<br>520-3000	8.4<br>8.2		H16<br>H16	
Pentanal	110-62-3	Valeraldehyde	n-C_4H_9CHO				0.014	0.014	**0.015**			**(Sharpe et al., 2004)**						278	1013	600-3000	8.9		H16	
2,3-Dimethylfuran	14920-89-9		$C_6H_8O^{\#}$				0.043	0.043	**0.045**			**(Johnson et al., 2010)**						278	1013	510-3000	5.7		H16	
2,5-Dimethylfuran	625-86-5		$C_6H_8O^{\#}$				0.052	0.052	**0.055**			**(Johnson et al., 2010)**						278	1013	550-3000	5.5		H16	
3,3-Dimethyl-2-pentanol	19781-24-9		$CH_3CHOHC(CH_3)_2CH_2CH_3$				0.066	0.065	**0.069**			**(Johnson et al., 2010)**						298	1013	520-3000	12.2		H16	
4-Methylpentanoic acid	646-07-1	4-Methylvaleric acid	$(CH_3)_2CHCH_2CH_2COOH$				0.119	0.119	**0.129**			**(Johnson et al., 2010)**						323	1013	500-3000	17.8		H16	
Methyl 2-methylbutanoate	868-57-5		$CH_3CH_2CH(CH_3)C(O)OCH_3$				0.115	0.114	**0.127**			**(Johnson et al., 2010)**						298	1013	520-3000	16.5		H16	
Methyl 2,2-dimethylpropanoate	598-98-1	Methyl pivalate	$(CH_3)_3CC(O)OCH_3$				0.107	0.107	**0.120**			**(Sharpe et al., 2004)**						278	1013	550-3000	16.0		H16	
4-hydroxy-4-methyl-2-pentanone	123-42-2	Diacetone alcohol	$CH_3C(O)CH_2COH(CH_3)_2$				0.083	0.083	**0.091**			**(Johnson et al., 2010)**						298	1013	520-3000	12.6		H16	
4-Penten-1-ol	821-09-0		CH_2=$CHCH_2CH_2CH_2OH$				0.071	0.069	**0.072**			**(Johnson et al., 2010)**						298	1013	520-3000	7.9		H16	
Acetic acid	64-19-7		CH_3COOH				0.101	0.101	**0.109**			**(Sharpe et al., 2004)**						298	1013	550-3000	14.1		H16	
2-Propenal	107-02-8	Acrolein	CH_2=$CHCHO$				0.048	0.047	**0.051**			**(Sharpe et al., 2004)**						278	1013	540-3000	7.0		H16	
Benzaldehyde	100-52-7		C_6H_5CHO				0.054	0.053	**0.058**			**(Sharpe et al., 2004)**						278	1013	600-3000	10.1		H16	
Benzylalcohol	100-51-6		$C_6H_5CH_2OH$				0.060	0.059	**0.059**			**(Sharpe et al., 2004)**						323	1013	550-3000	6.3		H16	
Methoxymethane	115-10-6	Dimethyl ether	CH_3OCH_3				0.056	0.055	**0.062**			**(Sharpe et al., 2004)**						278	1013	600-3000	7.9		H16	
Ethanol	64-17-5		CH_3CH_2OH				0.044	0.043	**0.044**			**(Sharpe et al., 2004)**						278	1013	600-3000	5.5		H16	
Ethyl formate	109-94-4		CH_3CH_2OCHO				0.115	0.115	**0.130**			**(Sharpe et al., 2004)**						278	1013	600-3000	14.7		H16	
1-Hydroxy-2-propanone	116-09-6	Acetol	$CH_3C(O)CH_2OH$				0.066	0.065	**0.071**			**(Johnson et al., 2010)**						323	1013	435-3000	8.6		H16	
Etheneoxyethane	109-92-2	Ethyl vinyl ether	CH_2=$CHOCH_2CH_3$				0.108	0.107	**0.118**			**(Sharpe et al., 2004)**						278	1013	570-3000	11.5		H16	
2-Butanone	78-93-3	Methylethyl ketone	$CH_3C(O)CH_2CH_3$				0.030	0.030	**0.033**			**(Sharpe et al., 2004)**						278	1013	550-3000	6.9		H16	
Formic acid dimer	14523-98-9		$(HCOOH)_2$				0.169	0.167	**0.187**			**(Sharpe et al., 2004)**						298	1013	540-3000	39.1		H16	
Furan	110-00-9		Cyc C_4H_4O				0.070	0.068	**0.071**			**(Sharpe et al., 2004)**						298	1013	550-3000	4.5		H16	
Furfuryl alcohol	98-00-0		$C_5H_6O_2^{\#}$				0.101	0.099	**0.101**			**(Sharpe et al., 2004)**						298	1013	550-3000	7.9		H16	
2,3-Butanedione	431-03-8	Biacetyl	$CH_3C(O)C(O)CH_3$				0.040	0.039	**0.044**			**(Johnson et al., 2010)**						278	1013	580-3000	6.8		H16	
2,6-Dimethoxyphenol	91-10-1		$C_8H_{10}O_3$				0.168	0.167	**0.185**			**(Johnson et al., 2010)**						323	1013	580-3000	21.2		H16	
2-Hexanol	626-93-7		n-C_4H_9-$CHOHCH_3$				0.053	0.053	**0.056**			**(Johnson et al., 2010)**						298	1013	520-3000	10.3		H16	
4-Methyl-1-pentanol	626-89-1		$(CH_3)_2CHCH_2CH_2CH_2OH$				0.052	0.051	**0.052**			**(Johnson et al., 2010)**						298	1013	520-3000	10.6		H16	
2-Methylfuran	534-22-5		$C_5H_6O^{\#}$				0.052	0.051	**0.054**			**(Johnson et al., 2010)**						298	1013	550-3000	4.9		H16	
2-Nonanone	821-55-6		n-C_7H_{15}-$C(O)CH_3$				0.037	0.037	**0.041**			**(Johnson et al., 2010)**						298	1013	550-3000	13.2		H16	
3,5,5-trimethylcyclohex-2-en-1-one	78-59-1	Isophorone	$C_9H_{14}O^{\#}$				0.031	0.031	**0.034**			**(Sharpe et al., 2004)**						298	1013	600-3000	12.7		H16	

Table S15 (cont.)

Name	CASRN	Identifier	Formula	Lifetime (yr) – H2013	Lifetime (yr) – New	Lifetime (yr) – Reference	RE (W m^{-2} ppb^{-1}) – This study – H2013 inst. RE	New inst. RE	New RE – const. profile	New RE – lifetime corr.	RE (W m^{-2} ppb^{-1}) – Literature – Value	Reference	Instantaneous/Adjusted	Vertical correction	RE calculation	GWP 100-yr – H2013	New – lifetime corr.	Absorption cross-sections – T (K)	p (hPa)	Waveno. range (cm^{-1})	Int. abs. cross-section (10^{-17} cm^2 molecule^{-1})	Reference	Database	Notes
2-Pentylfuran	3777-69-3		$C_9H_{14}O$ #				0.054	0.054	0.056			(Johnson et al., 2010)						323	1013	550-3000	9.6		H16	
							0.052	0.051	0.054			**(Sharpe et al., 2004)**						296		550-3000	9.4		P	
							0.052	0.051	**0.054**			**This study (avg.)**												
1-Methyl-4-(1-methylethenyl)-7-oxabicyclo[4.1.0]heptane	1195-92-2	Limonene oxide	$C_9H_{14}O$ #				0.054	0.054	**0.056**			**(Johnson et al., 2010)**						323	1013	550-3000	9.6		H16	
3-methylphenol	108-39-4	m-Creosol	$CH_3C_6H_4OH$				0.089	0.088	**0.097**			**(Sharpe et al., 2004)**						298	1013	570-3000	10.3		H16	
5-methyl-2-(1-methylethyl)-cyclohexanol	1490-04-6	Menthol	$C_{10}H_{20}O$ #				0.048	0.048	**0.049**			**(Johnson et al., 2010)**						323	1013	580-3000	12.7		H16	
2-methyl-3-pentanone	565-69-5		$CH_3CH_2C(O)CH(CH_3)_2$		5.5 days	(Diaz-de-Mera et al., 2015)					0.020	(Diaz-de-Mera et al., 2015)	H		H2013			298		600-2000	39.4			
											**0.020**	**This study**					**<1**							(1)
Ethyl methyl ether	540-67-0		$CH_3CH_2OCH_3$		2.0 days	(M P S Andersen et al., 2017a)					0.001	(M P S Andersen et al., 2017a)	H		Pinnock			298		1000-3100	8.0			
							0.064	0.063	0.071	0.002		**(Sharpe et al., 2004)**	H		Pinnock		<1	296		600-6500	9.2		P	
							0.064	0.063	0.071	**0.002**		**This study (avg.)**					**<1**							
Methyl butyl ether	628-28-4		n-C_4H_9-OCH_3				0.068	0.067	**0.075**			**(Sharpe et al., 2004)**						278	1013	600-3000	12.1		H16	
3-Pentanol	584-02-1		$CH_3CH_2CHOHCH_2CH_3$				0.053	0.053	**0.058**			**(Johnson et al., 2010)**						298	1013	570-3000	8.7		H16	
5-Methyl-2-hexanone	110-12-3	Methyl isoamyl ketone	$(CH_3)_2CHCH_2CH_2C(O)CH_3$				0.032	0.032	**0.036**			**(Sharpe et al., 2004)**						298	1013	550-3000	10.2		H16	
Methyl methacrylate	80-62-6		$CH_3C(=CH_2)C(O)OCH_3$				0.121	0.120	**0.134**			**(Sharpe et al., 2004)**						278	1013	550-3000	15.3		H16	
Ethyl Prop-2-enoate	140-88-5	Ethyl acrylate	$CH_2CHCOOC_2H_5$				0.153	0.152	**0.168**			**(Sharpe et al., 2004)**						278	1013	580-3000	17.8		H16	
Methyl propanoate	554-12-1		$CH_3OC(O)CH_2CH_3$				0.098	0.098	**0.110**			**(Sharpe et al., 2004)**						278	1013	530-3000	13.7		H16	
Acetic acid ethyl ether	141-78-6		$CH_3C(O)OCH_2CH_3$				0.107	0.107	**0.115**			**(Sharpe et al., 2004)**						278	1013	565-3000	16.1		H16	
2-Methyl propanoic acid	79-31-2	Isobutyric acid	$(CH_3)_2CHCOOH$				0.138	0.137	**0.146**			**(Johnson et al., 2010)**						340	1013	530-3000	15.5		H16	
Methyl salicylate	119-36-8		$C_8H_8O_3$ #				0.194	0.195	**0.216**			**(Sharpe et al., 2004)**						298	1013	550-3000	30.7		H16	
Methoxyethene	107-25-5	Methyl vinyl ether	$CH_2=CHOCH_3$				0.096	0.095	**0.105**			**(Johnson et al., 2010)**						278	1013	550-3000	9.7		H16	
Propanal	123-38-6	Propion aldehyde	CH_3CH_2CHO				0.019	0.019	**0.021**			**(Sharpe et al., 2004)**						278	1013	520-3000	6.8		H16	
3-Methoxyphenol	150-19-6		$C_7H_8O_2$#				0.165	0.164	**0.180**			**(Johnson et al., 2010)**						323	1013	550-3000	20.3		H16	
2-Methoxyphenol	90-05-1	Guaiacol	$C_7H_8O_2$ #				0.133	0.133	**0.140**			**(Johnson et al., 2010)**						298	1013	520-3000	17.2		H16	
3-methylfuran	930-27-8		C_5H_6O #				0.052	0.051	**0.054**			**(Johnson et al., 2010)**						298	1013	550-3000	4.9		H16	
5-Nonanol	623-93-8		$(CH_3CH_2CH_2)_2CHOH$				0.049	0.049	**0.051**			**(Johnson et al., 2010)**						298	1013	580-3000	13.4		H16	
2-Propenoic acid	79-10-7	Acrylic acid	$CH_2=CHCOOH$				0.151	0.149	**0.160**			**(Johnson et al., 2010)**						323	1013	525-3000	14.6		H16	
2-Oxopropanal	78-98-8	Methylglyoxal	$CH_3C(O)CHO$				0.019	0.019	**0.021**			**(Johnson et al., 2010)**						298	1013	550-3000	6.0		H16	
1,3,3-trimethyl-2-oxabicyclo[2.2.2]octane	470-82-6	Cineole	$C_{10}H_{18}O$ #				0.084	0.083	**0.089**			**(Johnson et al., 2010)**						323	1013	520-3000	14.0		H16	
Ethyl benzoate	93-89-0		$C_9H_{10}O_2$ #				0.142	0.141	**0.153**			**(Johnson et al., 2010)**						323	1013	600-3000	20.9		H16	
Furfural	98-01-1		$C_5H_4O_2$ #				0.092	0.091	**0.096**			**(Johnson et al., 2010)**						298	1013	550-3000	12.4		H16	
(2*E*)-3,7-dimethyl-2,6-octadien-1-ol	106-24-1	Geraniol	$C_{10}H_{18}O$ #				0.080	0.079	**0.084**			**(Johnson et al., 2010)**						323	1013	600-3000	12.2		H16	
2-Hydroxyacetaldehyde	141-46-8	Glycoaldehyde	C2H4O2				0.058	0.058	**0.063**			**(Johnson et al., 2010)**						298	1013	570-3000	8.6		H16	
Ethanedial	107-22-2	Glyoxal	CHOCHO				0.003	0.003	**0.003**			**(Johnson et al., 2010)**						298	1013	600-3000	4.5		H16	
Hexyl acetate	142-92-7		$(n\text{-}C_6H_{13})OC(O)CH_3$				0.116	0.116	**0.125**			**(Johnson et al., 2010)**						298	1013	580-3000	21.1		H16	
Octanoic acid	124-07-2		$(n\text{-}C_7H_{15})COOH$				0.106	0.105	**0.115**			**(Johnson et al., 2010)**						323	1013	550-3000	22.7		H16	
Isopentyl acetate	123-92-2		$(CH_3)_2CHCH_2CH_2)OC(O)CH_3$				0.120	0.120	**0.130**			**(Johnson et al., 2010)**						298	1013	550-3000	20.1		H16	
Methyl-3-oxobutanoate	105-45-3	Methyl acetoacetate					0.133	0.133	**0.146**			**(Johnson et al., 2010)**						298	1013	520-3000	21.1		H16	
Phenol	108-95-2		C_6H_5OH				0.084	0.083	**0.091**			**(Johnson et al., 2010)**						298	1013	550-3000	8.7		H16	
4_methyl-1,3-dioxolan-2-one	108-32-7	Propylene carbonate	$C_4H_6O_3$ #				0.171	0.170	**0.185**			**(Johnson et al., 2010)**						298	1013	600-3000	21.9		H16	

Table S15 (cont.)

Name	CASRN	Identifier	Formula	Lifetime (yr) H2013	Lifetime (yr) New	Lifetime (yr) Reference	RE ($W m^{-2} ppb^{-1}$) – This study: H2013 inst. RE	New inst. RE	New RE – const. profile	New RE – lifetime corr.	RE ($W m^{-2} ppb^{-1}$) – Literature: Value	Reference	Instantaneous/Adjusted	Vertical correction	RE calculation	GWP 100-yr: H2013	New – lifetime corr.	Absorption cross-sections: T (K)	p (hPa)	Waveno. range (cm^{-1})	Int. abs. cross-section (10^{-17} cm^2 $molecule^{-1}$)	Reference	Database	Notes
Pentanoic acid	109-52-4	Valeric acid	$(n\text{-}C_4H_9)COOH$				0.118	0.117	**0.126**			**(Johnson et al., 2010)**						340	1013	520-3000	16.6		H16	
2-Propenol	107-18-6	Allyl alcohol	$CH_2=CHCH_2OH$				0.075	0.074	**0.077**			**(Sharpe et al., 2004)**						296		550-3000	6.2		P	
Acetic anhydride	108-24-7	Acetyl acetate	$CH_3C(O)OC(O)CH_3$				0.032	0.031	**0.034**			**(Sharpe et al., 2004)**						296		590-3000	6.1		P	
3-Methyl-2-pentanone	565-61-7		$CH_3CH_2CH(CH_3)C(O)CH_3$				0.031	0.031	**0.035**			**(Sharpe et al., 2004)**						296		540-3000	8.6		P	
(3-hydroxy-2,2,4-trimethylpentyl) 2-methylpropanoate	77-68-9	Texanol	$C_{12}H_{24}O_3$				0.183	0.182	**0.201**			**(Sharpe et al., 2004)**						296		600-3000	26.1		P	
2-Methyl-2-propenal	78-85-3	Isobutenal	$CH_2C(CH_3)CHO$				0.035	0.035	**0.037**			**(Sharpe et al., 2004)**						296		600-3000	7.2		P	
2-Methyl-1-propanal	78-84-2	Isobutyraldehyde	$(CH_3)_2CHCHO$				0.023	0.022	**0.025**			**(Sharpe et al., 2004)**						296		525-3000	7.8		P	
2-Methoxyethanol	109-86-4		$CH_3OCH_2CH_2OH$				0.099	0.098	**0.106**			**(Sharpe et al., 2004)**						296		600-3000	11.3		P	
2-Hexanone	591-78-6		$(n\text{-}C_4H_9)C(O)CH_3$				0.030	0.030	**0.033**			**(Sharpe et al., 2004)**						296		550-3000	8.8		P	
2-Ethyl-1-hexanol	104-76-7		$C_8H_{18}O$ #				0.050	0.049	**0.050**			**(Sharpe et al., 2004)**						296		570-3000	12.5		P	
2-Ethoxyethyl acetate	111-15-9		$C_6H_{12}O_3$ #				0.188	0.187	**0.205**			**(Sharpe et al., 2004)**						296		540-3000	23.9		P	
2-Butoxyethanol	111-76-2		$(n\text{-}C_4H_9)OCH_2CH_2OH$				0.125	0.124	**0.134**			**(Sharpe et al., 2004)**						296		550-3000	16.3		P	
1,4-Dioxane	123-91-1		$C_4H_8O_2$ #				0.105	0.103	**0.115**			**(Sharpe et al., 2004)**						296		550-3000	11.6		P	
2-Methyloxirane	75-56-9	Propylene oxide	$C_3H_6O_2$ #				0.036	0.036	**0.039**			**(Sharpe et al., 2004)**						296		600-3000	3.4		P	
2-Ethyloxirane	106-88-7	1,2-Epoxybutane	C_4H_8O #				0.037	0.036	**0.040**			**(Sharpe et al., 2004)**						296		600-3000	4.5		P	
1-Propanol	71-23-8		$CH_3CH_2CH_2OH$				0.048	0.047	**0.049**			**(Sharpe et al., 2004)**						296		600-3000	7.0		P	
1-Hexanol	111-27-3		$(n\text{-}C_5H_{11})CH_2OH$				0.048	0.047	**0.048**			**(Sharpe et al., 2004)**						296		600-3000	10.8		P	
1-Hexanoic acid	142-62-1		$(n\text{-}C_5H_{11})COOH$				0.116	0.115	**0.125**			**(Sharpe et al., 2004)**						296		550-3000	17.9		P	
1-Heptanol	111-70-6		$(n\text{-}C_6H_{13})CH_2OH$				0.049	0.048	**0.049**			**(Sharpe et al., 2004)**						296		600-3000	12.2		P	
Butyl acetate	123-86-4		$(n\text{-}C_4H_9)OC(O)CH_3$				0.115	0.115	**0.124**			**(Sharpe et al., 2004)**						296		570-3000	18.7		P	
Butanal	123-72-8	Butyraldehyde	$CH_3CH_2CH_2CHO$				0.017	0.017	**0.018**			**(Sharpe et al., 2004)**						296		600-3000	8.0		P	
Butanoic acid	107-92-6	Butyric acid	$(n\text{-}C_4H_9)COOH$				0.112	0.112	**0.120**			**(Sharpe et al., 2004)**						296		550-3000	15.3		P	
Diethylketone	96-22-0		$(CH_3CH_2)_2CO$				0.037	0.037	**0.040**			**(Sharpe et al., 2004)**						296		550-3000	7.8		P	
Diisopropyl ether	108-20-3		$(CH_3)_2CHOCH(CH_3)_2$				0.090	0.090	**0.097**			**(Sharpe et al., 2004)**						296		600-3000	12.9		P	
Dimethoxymethane	109-87-5		$CH_3OCH_2OCH_3$				0.162	0.159	**0.169**			**(Sharpe et al., 2004)**						296		540-3000	14.8		P	
Dimethyl carbonate	616-38-6		$CH_3O)_2CO$				0.109	0.109	**0.120**			**(Sharpe et al., 2004)**						296		575-3000	23.8		P	
1-(3-methoxypropoxy)propan-1-ol	34590-94-8	Dipropylene glycol methyl ether	$C_7H_{16}O$#				0.200	0.198	**0.218**			**(Sharpe et al., 2004)**						296		530-3000	21.7		P	
Dipropyl ether	111-43-3		$(CH_3CH_2CH_2)_2O$				0.083	0.082	**0.091**			**(Sharpe et al., 2004)**						296		600-3000	13.6		P	
Ethyl butyrate	105-54-4		$CH_3CH_2CH_2C(O)OCH_2CH_3$				0.130	0.130	**0.143**			**(Sharpe et al., 2004)**						296		550-3000	18.3		P	
Ethylene oxide	75-21-8		cyc C_2H_4O				0.039	0.038	**0.043**			**(Sharpe et al., 2004)**						296		600-3000	2.2		P	
2-ethoxy-2-methylpropane	637-92-3	Ethyl tert-butyl ether	$(CH_3)_3COCH_2CH_3$				0.085	0.085	**0.094**			**(Sharpe et al., 2004)**						296		600-3000	12.2		P	
1,2-Dimethoxyethane	110-71-4	Dimethyl glycol	$CH_3O(CH_2)_2OCH_3$				0.124	0.123	**0.136**			**(Sharpe et al., 2004)**						296		520-3000	15.0		P	
3-methyl-1-butanol	123-51-3	Isoamyl alcohol	$(CH_3)_2CHCH_2CH_2OH$				0.049	0.048	**0.050**			**(Sharpe et al., 2004)**						296		600-3000	9.6		P	
Isopropyl acetate	108-21-4		$(CH_3)_2CHOC(O)CH_3$				0.127	0.127	**0.139**			**(Sharpe et al., 2004)**						296		570-3000	17.8		P	
Methyl acetate	79-20-9		$CH_3OC(O)CH_3$				0.084	0.084	**0.091**			**(Sharpe et al., 2004)**						296		550-3000	13.9		P	
Methyl acrylate	96-33-3		$CH_2=CHC(O)OCH_3$				0.132	0.131	**0.146**			**(Sharpe et al., 2004)**						296		580-3000	15.7		P	
Methyl benzoate	93-58-3		$C_6H_5C(O)OCH_3$				0.118	0.118	**0.129**			**(Sharpe et al., 2004)**						296		550-3000	19.4		P	
Methyl formate	107-31-3		CH_3OCHO				0.095	0.095	**0.108**			**(Sharpe et al., 2004)**						296		600-3000	12.7		P	
Methyl isobutyl ketone	108-10-1	MIBK	$(CH_3)_2CHCH_2C(O)CH_3$				0.034	0.034	**0.038**			**(Sharpe et al., 2004)**						296		550-3000	9.6		P	
Methyl isobutyrate	547-63-7		$(CH_3)_2CHC(O)OCH_3$				0.110	0.109	**0.123**			**(Sharpe et al., 2004)**						296		560-3000	15.1		P	
3-methyl-2-butanone	563-80-4	Methyl isopropyl ketone	$(CH_3)_2CHC(O)CH_3$				0.032	0.032	**0.035**			**(Sharpe et al., 2004)**						296		540-3000	7.8		P	
n-Amyl acetate	628-63-7		$(n\text{-}C_5H_{11})OC(O)CH_3$				0.105	0.106	**0.114**			**(Sharpe et al., 2004)**						296		570-3000	18.3		P	
Butanol	71-36-3		$CH_3CH_2CH_2CH_2OH$				0.049	0.048	**0.050**			**(Sharpe et al., 2004)**						296		600-3000	8.6		P	

Table S15 (cont.)

Name	CASRN	Identifier	Formula	Lifetime (yr) H2013	Lifetime (yr) New	Lifetime (yr) Reference	RE ($W m^{-2} ppb^{-1}$) – This study H2013 inst. RE	New inst. RE	New RE – const. profile	New RE – lifetime corr.	RE ($W m^{-2} ppb^{-1}$) – Literature Value	Reference	Instantaneous/Adjusted	Vertical correction	RE calculation	GWP 100-yr H2013	New – lifetime corr.	Absorption cross-sections T (K)	p (hPa)	Waveno. range (cm^{-1})	Int. abs. cross-section (10^{-17} cm^2 $molecule^{-1}$)	Reference	Database	Notes
Vinyl acetate	108-05-4		CH_2=CHOC(O)CH_3				0.202	0.202	**0.224**			**(Sharpe et al., 2004)**						296		540-3000	20.9		P	
2-phenyl-oxirane	96-09-3	Styrene oxide	C_8H_8O #				0.055	0.054	**0.058**			**(Sharpe et al., 2004)**						296		510-3000	4.3		P	
2-methyl-2-propanol	75-65-0	t-Butyl alcohol	$(CH_3)_3$COH				0.054	0.053	**0.059**			**(Sharpe et al., 2004)**						296		550-3000	7.5		P	
2-methoxy-2-methylbutane	994-05-8	tert-Amyl methyl ether	$CH_3CH_2C(CH_3)_2OCH_3$				0.071	0.070	**0.078**			**(Sharpe et al., 2004)**						296		600-3000	11.5		P	
tert-Butyl acetate	540-88-5		$(CH_3)_3$COC(O)CH_3				0.128	0.128	**0.140**			**(Sharpe et al., 2004)**						296		560-3000	19.0		P	
tert-Butyl methyl ether	1634-04-4		$(CH_3)_3COCH_3$				0.068	0.067	**0.076**			**(Sharpe et al., 2004)**						296		600-3000	10.4		P	
Propargyl alcohol	107-19-7		$HCCCH_2OH$				0.058	0.057	**0.055**			**(Sharpe et al., 2004)**						296		530-3000	6.1		P	
Propyl acetate	109-60-4		$CH_3CH_2CH_2OC(O)CH_3$				0.114	0.114	**0.124**			**(Sharpe et al., 2004)**						296		560-3000	17.6		P	
Propylene glycol	57-55-6		$CH_3CHOHCH_2OH$				0.085	0.084	**0.087**			**(Sharpe et al., 2004)**						296		590-3000	9.1		P	
2-Butanol	78-92-2	sec-Butyl alcohol	$CH_3CH_2CHOHCH_3$				0.051	0.051	**0.055**			**(Sharpe et al., 2004)**						296		560-3000	7.6		P	
Neopentyl alcohol	75-84-3		$(CH_3)_3CCH_2OH$				0.044	0.044	**0.043**			**(Sharpe et al., 2004)**						296		600-3000	9.2		P	
Octafluorotetrahydrofuran	773-14-8	Octafluorooxolane	c-C_4F_8O		3000.0	(Vollmer et al., 2019)	0.430	0.428	0.469	**0.463**	0.430	**(Vollmer et al., 2019)**		H	H2013		**14,700**	296		500-1500	32.1			
Crotonaldehyde	4170-30-3		CH_3CH=CHCHO		0.2 days	(Lindenmaier et al., 2017)					<0.001<br>**0.000**	(Lindenmaier et al., 2017)<br>**This study**			H2013		 **<1**	296		670-3170				(2)
Methyl vinyl ketone	78-94-4	MVK	$CH_3C(O)CH$=CH_2		0.4 days	(Lindenmaier et al., 2017)	 0.046<br>0.046	 0.045<br>0.045	 0.050<br>0.050	 0.000<br>**0.000**	<0.001	(Lindenmaier et al., 2017)<br>**(Sharpe et al., 2004)**<br>**This study (avg.)**			H2013		 <1<br>**<1**	296<br>296		635-3150<br>540-3000	 6.5		 P	
trans-Crotonaldehyde							0.049	0.048	**0.053**			**(Johnson et al., 2010)**						298	1013	500-3000	8.8		H16	
allyl ether	557-40-4	AE	$(CH_2$=$CHCH_2)_2O$		0.1 days	(Antiñolo et al., 2017)					 **<0.001**	(Antiñolo et al., 2017)<br>**This study**			H2013		 **<1**	298		500-1500	6.8			(3)
allyl ethyl ether	557-31-3	AEE	$CH_3CH_2OCH_2CH$=CH_2		0.2 days	(Antiñolo et al., 2017)					 **<0.001**	(Antiñolo et al., 2017)<br>**This study**			H2013		 **<1**	298		500-1500	6.1			(3)
2-Propenoic acid, hexyl ester	2499-95-8		CH_2=CHC(O)O$(CH_2)_5CH_3$									(Moreno et al., 2014)			Pinnock			298						(4)
2-Propenoic acid, 1-ethylpentyl ester	3953-31-9		CH_2=CHC(O)OCH$(CH_2CH_3)(CH_2)_3CH_3$									(Moreno et al., 2014)			Pinnock			298						(4)
(E/Z)-2-butenoic acid methylester	18707-60-3		CH_3CH=CHC(O)OCH_3									(Moreno et al., 2014)			Pinnock			298						(4)
Methyl 3,3-dimethacrylate	924-50-5		$(CH_3)_2C$=CHC(O)OCH_3									(Moreno et al., 2014)			Pinnock			298						(4)
(Z)-2-hexen-1-ol	928-94-9		$CH_3CH_2CH_2CH$=$CHCH_2OH$		0.0 days	(Peirone et al., 2014)					0.038<br>**0.038**	(Peirone et al., 2014)<br>**This study**			Pinnock		 **<1**	298		500-1500	2.6			(5)
(E)-3-hexen-1-ol	928-95-0		$CH_3CH_2CH_2CH$=$CHCH_2OH$		0.0 days	(Peirone et al., 2014)					0.036<br>**0.036**	(Peirone et al., 2014)<br>**This study**			Pinnock		 **<1**	298		500-1500	1.9			(5)

(1) RE value is from (Diaz-de-Mera et al., 2015); (2) RE value is from (Lindenmaier et al., 2017); (3) Based on the GWP(20) value reported in (Antiñolo et al., 2017) (they do not report the RE) it is likely that the lifetime-corrected RE is less than 0.001 W m^{-2} ppb^{-1}; (4) This study (Moreno et al., 2014) is mostly describing the results without giving the numbers, very low GWPs (<1E-2); (5) RE value is from (Peirone et al., 2014)

Table S16. Iodocarbons and hydroiodocarbons

Please refer to page 2 of this document for a description of the table.

				Lifetime (yr)			RE (W m^{-2} ppb^{-1}) – This study				RE (W m^{-2} ppb^{-1}) – Literature					GWP 100-yr		Absorption cross-sections						
Name	CASRN	Identifier	Formula	H2013	New	Reference	H2013 inst. RE	New inst. RE	New RE – const. profile	New RE – lifetime corr.	Value	Reference	Instantaneous/Adjusted	Vertical correction	RE calculation	H2013	New – lifetime corr.	*T* (K)	*p* (hPa)	Waveno. range (cm^{-1})	Int. abs. cross-section (10^{-17} cm^2 molecule^{-1})	Reference	Database	Notes
Diiodomethane	75-11-6		CH_2I_2				0.036	0.036	**0.038**			**(Sharpe et al., 2004)**						298	1013	530-3000	2.0		H16	
Iodoethane	75-03-6		CH_3CH_2I				0.018	0.018	**0.021**			**(Sharpe et al., 2004)**						298	1013	600-3000	2.4		H16	
Iodomethane	74-88-4		CH_3I				0.008	0.008	**0.009**			**(Sharpe et al., 2004)**						298	1013	500-3000	1.0		H16	
3-Iodo-1-propene	556-56-9	Allyl iodide	$CH_2{=}CHCH_2I$				0.039	0.038	**0.042**			**(Sharpe et al., 2004)**						296		510-3000	2.8		P	
2-Iodopropane	75-30-9	Isopropyl iodide	$(CH_3)_2CHI$				0.027	0.027	**0.030**			**(Sharpe et al., 2004)**						296		600-3000	4.0		P	
2-Iodo-2-methylpropane	558-17-8		$(CH_3)_3CI$				0.029	0.028	**0.032**			**(Sharpe et al., 2004)**						296		550-3000	5.2		P	

Table S17. Nitriles, amines and other nitrogenated hydrocarbons

Please refer to page 2 of this document for a description of the table.

				Lifetime (yr)			RE (W m^{-2} ppb^{-1}) – This study				RE (W m^{-2} ppb^{-1}) – Literature					GWP 100-yr		Absorption cross-sections						
Name	CASRN	Identifier	Formula	H2013	New	Reference	H2013 inst. RE	New inst. RE	New RE – const. profile	New RE – lifetime corr.	Value	Reference	Instantaneous/Adjusted	Vertical correction	RE calculation	H2013	New – lifetime corr.	*T* (K)	*p* (hPa)	Waveno. range (cm^{-1})	Int. abs. cross-section (10^{-17} cm^2 molecule^{-1} cm^{-1})	Reference	Database	Notes
Acetonitrile	75-05-8	Methyl Cyanide	CH_3CN				0.002	0.002	**0.002**			**(Harrison and Bernath, 2012)**						297	1013	880-1700	0.5		H16	
Peroxyacetyl nitrate	2278-22-0		$CH_3C(O)OONO_2$				0.002<br>0.160<br>0.161<br>0.160	0.002<br>0.158<br>0.159<br>0.158	0.002<br>0.173<br>0.174<br>**0.173**			(Allen et al., 2005)<br>**(Allen, priv. comm. 2005)**<br>**(Harrison, priv. comm.)**<br>**This study (avg.)**						250<br>273<br>295		1590-2200<br>560-1400<br>560-1400	6.4<br>9.8<br>9.8		H16<br>H16<br>H16	
Diethylamine	109-89-7		$(CH_3CH_2)_2NH$				0.035	0.035	**0.038**			**(Sharpe et al., 2004)**						278	1013	590-3000	10.1		H16	
N-Butylamine	109-73-9		$(n\text{-}C_4H_9)NH_2$				0.064	0.063	**0.068**			**(Sharpe et al., 2004)**						278	1013	600-3000	8.8		H16	
Diisopropylamine	108-18-9		$((CH_3)_2CH)_2NH$				0.037	0.037	**0.041**			**(Sharpe et al., 2004)**						278	1013	550-3000	11.4		H16	
Triethylamine	121-44-8		$(CH_3CH_2)_3N$				0.044	0.044	**0.048**			**(Sharpe et al., 2004)**						278	1013	575-3000	12.6		H16	
Dimethylamine	124-40-3		$(CH_3)_2NH$				0.035	0.035	**0.036**			**(Sharpe et al., 2004)**						278	1013	550-3000	7.8		H16	
Ethylamine	75-04-7		$CH_3CH_2NH_2$				0.063	0.062	**0.067**			**(Sharpe et al., 2004)**						278	1013	600-3000	6.3		H16	
Ethylenediamine	107-15-3		$NH_2CH_2CH_2NH_2$				0.112	0.110	**0.119**			**(Sharpe et al., 2004)**						298	1013	600-3000	9.0		H16	
Hydrazine	302-01-2		NH_2NH_2				0.071	0.069	**0.076**			**(Sharpe et al., 2004)**						298	1013	600-3000	3.5		H16	
Isocyanic acid	75-13-8		HNCO				0.076	0.075	**0.078**			**(Sharpe et al., 2004)**						298	1013	500-3000	13.5		H16	
Isopropylamine	75-31-0		$(CH_3)_2CHNH_2$				0.065	0.064	**0.070**			**(Sharpe et al., 2004)**						278	1013	600-3000	7.4		H16	
Trimethylamine	75-50-3		$(CH_3)_3N$				0.032	0.032	**0.033**			**(Sharpe et al., 2004)**						278	1013	600-3000	10.2		H16	
N,N-Diethylaniline	91-66-7		$C_6H_5N(CH_2CH_3)_2$				0.071	0.071	**0.076**			**(Sharpe et al., 2004)**						298	1013	550-3000	16.5		H16	
Nitrous acid	7782-77-6		HONO				0.138	0.135	**0.142**			**(Sharpe et al., 2004)**						298	1013	550-3000	10.8		H16	
2-Methylbenzenamine	95-53-4	o-Toluidine	C_7H_9N #				0.053	0.053	**0.055**			**(Sharpe et al., 2004)**						298	1013	550-3000	8.9		H16	
Piperidine	110-89-4		cyc $C_5H_{11}N$				0.043	0.043	**0.044**			**(Sharpe et al., 2004)**						298	1013	510-3000	10.6		H16	
2-Methylaziridine	75-55-8	Propylenimine	C_3H_7N #				0.048	0.047	**0.052**			**(Sharpe et al., 2004)**						298	1013	600-3000	4.0		H16	
Pentan-2-amine	41444-43-3	2-Pentamine	$C_5H_{13}N$				0.062	0.061	**0.067**			**(Sharpe et al., 2004)**						298	1013	540-3000	9.2		H16	
2-Vinylpyridine	100-69-6		C_7H_7N #				0.051	0.050	**0.054**			**(Johnson et al., 2010)**						298	1013	540-3000	4.5		H16	
Prop-2-enenitrile	107-13-1	Acrylonitrile	$CH_2{=}CHCN$				0.025	0.024	**0.027**			**(Sharpe et al., 2004)**						298	1013	550-3000	1.4		H16	
N,N-diethyl formamide	617-84-5		$(CH_3CH_2)_2NCHO$				0.060	0.060	**0.066**			**(Johnson et al., 2010)**						298	1013	520-3000	18.5		H16	
Benzonitrile	100-47-0	Phenyl cyanide	C_6H_5CN				0.028	0.027	**0.028**			**(Sharpe et al., 2004)**						296		520-3000	2.4		P	
Phenylamine	62-53-3	Aniline	$C_6H_5NH_2$				0.051	0.050	**0.053**			**(Sharpe et al., 2004)**						296		570-3000	8.7		P	
Pentyl nitrate	1002-16-0	amyl nitrate	$(n\text{-}C_5H_{11})ONO_2$				0.182	0.178	**0.195**			**(Sharpe et al., 2004)**						296		540-3000	22.8		P	
3-isothiocyanato-1-propene	57-06-7	Allyl isothiocyante	$CH_2{=}CHCH_2NCS$				0.057	0.056	**0.062**			**(Sharpe et al., 2004)**						296		580-3000	23.0		P	
2-Hydroxy-2-methylpropanenitrile	75-86-5	Acetone cyanohydrin	$(CH_3)_2C(OH)CN$				0.062	0.061	**0.069**			**(Sharpe et al., 2004)**						296		600-3000	5.7		P	
4-Methylpyridine	108-89-4	4-Picoline	C_6H_7N #				0.023	0.023	**0.025**			**(Sharpe et al., 2004)**						296		600-3000	3.4		P	
3-Methylpyridine	108-99-6	3-Picoline	C_6H_7N #				0.021	0.020	**0.021**			**(Sharpe et al., 2004)**						296		600-3000	3.1		P	
2-Methylpyridine	109-06-8	2-Picoline	C_6H_7N #				0.023	0.023	**0.024**			**(Sharpe et al., 2004)**						296		530-3000	4.0		P	
2-Nitropropane	79-46-9		$(CH_3)_2CHNO_2$				0.023	0.023	**0.025**			**(Sharpe et al., 2004)**						296		575-3000	8.0		P	
2,4-Diisocyanate-1-methylbenzene	584-84-9	2,4-Toulene diisocyanate	$C_9H_6N_2O_2$ #				0.104	0.102	**0.109**			**(Sharpe et al., 2004)**						296		540-3000	66.9		P	
1-Nitropropane	108-03-2		$(n\text{-}C_3H_7)NO_2$				0.020	0.021	**0.023**			**(Sharpe et al., 2004)**						296		600-3000	8.3		P	
1,1-Dimethylhydrazine	57-14-7		$(CH_3)_2NNH_2$				0.057	0.056	**0.061**			**(Sharpe et al., 2004)**						296		600-3000	7.5		P	
Cadaverine	462-94-2		$NH_2(CH_2)_5NH_2$				0.140	0.137	**0.147**			**(Sharpe et al., 2004)**						296		550-3000	14.5		P	
Chloropicrin	76-06-2		CCl_3NO_2				0.141	0.137	**0.152**			**(Sharpe et al., 2004)**						296		600-3000	10.7		P	
Cyanogenchloride			CNCl				0.004	0.004	**0.005**			**(Sharpe et al., 2004)**						296		600-3000	0.5		P	
Cyanogen	460-195		NCCN				0.001	0.001	**0.001**			**(Sharpe et al., 2004)**						296		575-3000	0.2		P	
Propanenitrile	107-12-0	Ethyl cyanide	CH_3CH_2CN				0.003	0.003	**0.004**			**(Sharpe et al., 2004)**						296		600-3000	1.2		P	
3-Butenenitrile	109-75-1	Allyl cyanide	$CH_2{=}CHCH_2CN$		0.7 days	(Antinolo et al., 2019)					<0.001<br>**<0.001**	(Antinolo et al., 2019)<br>**This study**	A	H	H2013		**<1**	298		500-2500	1.6			(1)
Ethyl nitrite	109-95-5		CH_3CH_2ONO				0.178	0.174	**0.186**			**(Sharpe et al., 2004)**						296		525-3000	13.1		P	
2-Methyl-2-propenenitrile	126-98-7	Methylacrylonitrile	$CH_2{=}C(CH_3)CN$				0.022	0.021	**0.023**			**(Sharpe et al., 2004)**						296		520-3000	2.0		P	
Methylamine	74-89-5		CH_3NH_2				0.056	0.055	**0.059**			**(Sharpe et al., 2004)**						296		530-3000	5.0		P	
Methylhydrazine	60-34-4		CH_3NHNH_2				0.065	0.063	**0.069**			**(Sharpe et al., 2004)**						296		600-3000	4.8		P	
Morpholine	110-91-8		C_4H_9NO #				0.101	0.100	**0.108**			**(Sharpe et al., 2004)**						296		550-3000	11.5		P	
n-Butyl isocyanate	111-36-4		$(n\text{-}C_4H_9)NCO$				0.026	0.026	**0.027**			**(Sharpe et al., 2004)**						296		530-3000	22.9		P	
Nitric acid	7697-37-2		$HONO_2$				0.110	0.109	**0.120**			**(Sharpe et al., 2004)**						296		720-3000	14.7		P	
Nitric oxide	10102-43-9		NO				<0.001	<0.001	**<0.001**			**(Sharpe et al., 2004)**						296		600-3000	0.5		P	
Nitrogen dioxide	10102-44-0		NO_2				0.004	0.004	**0.005**			**(Sharpe et al., 2004)**						296		600-3000	6.3		P	
Nitromethane	75-52-5		CH_3NO_2				0.014	0.014	**0.015**			**(Sharpe et al., 2004)**						296		580-3000	7.5		P	
Nitrobenzene	98-95-3		$C_6H_5NO_2$				0.042	0.042	**0.045**			**(Sharpe et al., 2004)**						298	1013	550-3000	9.9		H16	
Nitrosyl chloride	2696-92-6		ClNO				0.028	0.027	**0.027**			**(Sharpe et al., 2004)**						296		545-3000	10.8		P	
Methyl nitrite	624-91-9		CH_3ONO				0.135	0.133	**0.140**			**(Sharpe et al., 2004)**						298	1013	520-3000	10.4		H16	
Pyridine	110-86-1		cyc C_5H_5N				0.013	0.013	**0.013**			**(Sharpe et al., 2004)**						296		565-3000	2.6		P	
Trifluoronitrosomethane	334-99-6		CF_3NO				0.119	0.119	**0.134**			**(Sharpe et al., 2004)**						296		520-3000	14.3		P	
2-Methyl-2-butanamine	594-39-8	tert-Amylamine	$CH_3CH_2C(CH_3)_2NH_2$				0.069	0.068	**0.074**			**(Sharpe et al., 2004)**						296		500-3000	9.2		P	
Quinoline	91-22-5		C_9H_7N #				0.059	0.058	**0.062**			**(Sharpe et al., 2004)**						296		570-3000	4.2		P	
Dimethylcarbamoyl chloride	79-44-7		$(CH_3)_2NC(O)Cl$				0.123	0.122	**0.134**			**(Sharpe et al., 2004)**						296		540-3000	21.2		P	

(1) RE value is from (Antinolo et al., 2019)

Table S18. Sulfur containing compounds

Please refer to page 2 of this document for a description of the table.

Name	CASRN	Identifier	Formula	Lifetime (yr)			RE (W m^{-2} ppb^{-1}) – This study				RE (W m^{-2} ppb^{-1}) – Literature					GWP 100-yr		Absorption cross-sections						
				H2013	New	Reference	H2013 inst. RE	New inst. RE	New RE – const. profile	New RE – lifetime corr.	Value	Reference	Instantaneous/Adjusted	Vertical correction	RE calculation	H2013	New – lifetime corr.	*T* (K)	*p* (hPa)	Waveno. range (cm^{-1})	Int. abs. cross-section (10^{-17} cm^{2} molecule^{-1})	Reference	Database	Notes
Carbon disulfide	75-15-0		S=C=S				0.004	0.004	**0.004**			**(Sharpe et al., 2004)**						298	1013	600-3000	10.9		H16	
Dimethyl disulfide	624-92-0		CH_3SSCH_3				0.007	0.007	**0.008**			**(Sharpe et al., 2004)**						298	1013	600-3000	1.8		H16	
Dimethyl sulfate	77-78-1		$CH_3O(SO_2)OCH_3$				0.352	0.346	**0.357**			**(Sharpe et al., 2004)**						298	1013	525-3000	23.1		H16	
Dimethyl sulfide	75-18-3		CH_3SCH_3				0.005	0.005	**0.005**			**(Sharpe et al., 2004)**						298	1013	600-3000	2.4		H16	
Dimethyl sulfoxide	67-68-5		$(CH_3)_2S{=}O$				0.059	0.058	**0.065**			**(Sharpe et al., 2004)**						298	1013	575-3000	4.4		H16	
Methyl isothiocyanate	556-61-6		CH_3NCS				0.026	0.026	**0.028**			**(Sharpe et al., 2004)**						298	1013	600-3000	20.6		H16	
Methanethiol	74-93-1	Methyl mercaptan	CH_3SH				0.005	0.005	**0.005**			**(Sharpe et al., 2004)**						298	1013	600-3000	1.0		H16	
Benzenethiol	108-98-5	Thiophenol	C_6H_5SH				0.025	0.025	**0.026**			**(Sharpe et al., 2004)**						296		550-3000	3.2		P	
2-Methyl-1-propanethiol	513-44-0		$(CH_3)_2CHCH_2SH$				0.011	0.011	**0.012**			**(Sharpe et al., 2004)**						296		550-3000	4.8		P	
Carbonyl Sulfide	463-58-1	OCS	COS				0.015	0.015	**0.016**			**(Sharpe et al., 2004)**						296		510-3000	12.3		P	
Diethyl sulfate	64-67-5		$CH_3CH_2O(SO_2)OCH_2CH_3$				0.442	0.432	**0.460**			**(Sharpe et al., 2004)**						296		520-3000	26.7		P	
Diethyl sulfide	352-93-2		$(CH_3CH_2)_2S$				0.012	0.012	**0.013**			**(Sharpe et al., 2004)**						296		600-3000	4.9		P	
Thiirane	420-12-2	Ethylene sulfide	cyc C_2H_4S				0.010	0.010	**0.009**			**(Sharpe et al., 2004)**						296		560-3000	1.1		P	
Ethyl mercaptan	75-08-1		CH_3CH_2SH				0.008	0.008	**0.008**			**(Sharpe et al., 2004)**						296		585-3000	2.3		P	
Sulfur dioxide	7446-09-5		SO_2				0.016	0.016	**0.018**			**(Sharpe et al., 2004)**						296		500-3000	3.5		P	
Sulfur trioxide	7446-11-9		SO_3				0.030	0.030	**0.030**			**(Sharpe et al., 2004)**						296		440-3000	5.8		P	
Thiophene	110-02-1		cyc C_4H_4S				0.030	0.029	**0.030**			**(Sharpe et al., 2004)**						296		580-3000	3.1		P	
Thiophosgene	463-71-8		$Cl_2C{=}S$				0.169	0.166	**0.184**			**(Sharpe et al., 2004)**						296		600-3000	7.5		P	
Tetrahydrothiophene	110-01-0		cyc C_4H_8S				0.005	0.005	**0.006**			**(Sharpe et al., 2004)**						296		600-3000	3.0		P	
Methanesulfonyl chloride	124-63-0		$CH_3(SO_2)Cl$				0.121	0.120	**0.127**			**(Sharpe et al., 2004)**						296		520-3000	9.6		P	
Perchloromethyl mercaptan	594-42-3		CCl_3SCl				0.117	0.115	**0.123**			**(Sharpe et al., 2004)**						296		520-3000	4.5		P	

Table S19. Silicon containing compounds

Please refer to page 2 of this document for a description of the table.

Name	CASRN	Identifier	Formula	Lifetime (yr) H2013	Lifetime (yr) New	Lifetime (yr) Reference	RE ($W m^{-2} ppb^{-1}$) – This study: H2013 inst. RE	New inst. RE	New RE – const. profile	New RE – lifetime corr.	RE ($W m^{-2} ppb^{-1}$) – Literature: Value	Reference	Instantaneous/Adjusted	Vertical correction	RE calculation	GWP 100-yr: H2013	New – lifetime corr.	Absorption cross-sections: *T* (K)	*p* (hPa)	Waveno. range (cm^{-1})	Int. abs. cross-section (10^{-17} cm^2 $molecule^{-1}$ cm^{-1})	Reference	Database	Notes
hexamethyldisiloxane	107-46-0		$C_6H_{12}OSi_2$ #		9.0 days	(Bernard et al., 2017)					0.047<br>**0.047**	(Bernard et al., 2017)<br>**This study**		H	H2013		**<1**	294		650-3000	29.9			(1)
octamethyltrisiloxane	107-51-7		$C_8H_{24}O_2Si_3$ #		7.0 days	(Bernard et al., 2017)					0.060<br>**0.060**	(Bernard et al., 2017)<br>**This study**		H	H2013		**<1**	294		650-3000	48.0			(1)
decamethyltetrasiloxane	141-62-8		$C_{10}H_{30}O_3Si_4$ #		5.0 days	(Bernard et al., 2017)					0.060<br>**0.060**	(Bernard et al., 2017)<br>**This study**		H	H2013		**<1**	294		650-3000	64.5			(1)
dodecamethylpentasiloxane	141-63-9		$C_{12}H_{36}O_4Si_5$ #		4.0 days	(Bernard et al., 2017)					0.064<br>**0.064**	(Bernard et al., 2017)<br>**This study**		H	H2013		**<1**	294		650-3000	82.4			(1)
hexamethylcyclotrisiloxane	541-05-9		$C_6H_{18}O_3Si_3$#		14.0 days	(Bernard et al., 2017)					0.100<br>**0.100**	(Bernard et al., 2017)<br>**This study**		H	H2013		**<1**	294		650-3000	47.8			(1)
octamethylcyclotetrasiloxane	556-67-2		$C_8H_{24}O_4Si_4$ #		10.0 days	(Bernard et al., 2017)					0.120<br>**0.120**	(Bernard et al., 2017)<br>**This study**		H	H2013		**<1**	294		650-3000	65.5			(1)
decamethylcyclopentasiloxane	541-02-6		$C_{10}H_{30}O_5Si_5$ #		6.0 days	(Bernard et al., 2017)					0.098<br>**0.098**	(Bernard et al., 2017)<br>**This study**		H	H2013		**<1**	294		650-3000	84.8			(1)
dodecamethylcyclohexasiloxane	540-97-6		$C_{12}H_{36}O_6Si_6$ #		4.0 days	(Bernard et al., 2017)					0.086<br>**0.086**	(Bernard et al., 2017)<br>**This study**		H	H2013		**<1**	294		650-3000	105.5			(1)
Dichlorosilane	4109-96-0		SiH_2Cl_2				0.205	0.200	**0.214**			**(Sharpe et al., 2004)**						296		510-3000	10.8		P	
Methyltrichlorosilane	75-79-6		CH_3SiCl_3				0.179	0.176	**0.180**			**(Sharpe et al., 2004)**						296		520-3000	8.6		P	
Silane	7803-62-5		SiH_4				0.179	0.174	**0.194**			**(Sharpe et al., 2004)**						296		600-3000	11.4		P	

(1) RE value is from (Bernard et al., 2017)

Table S20. Other compounds

Please refer to page 2 of this document for a description of the table.

Name	CASRN	Identifier	Formula	Lifetime (yr) H2013	Lifetime (yr) New	Lifetime (yr) Reference	RE (W m^{-2} ppb^{-1}) – This study H2013 inst. RE	New inst. RE	New RE – const. profile	New RE – lifetime corr.	RE (W m^{-2} ppb^{-1}) – Literature Value	Reference	Instantaneous/Adjusted	Vertical correction	RE calculation	GWP 100-yr H2013	New – lifetime corr.	Absorption cross-sections T (K)	p (hPa)	Waveno. range (cm^{-1})	Int. abs. cross-section (10^{-17} cm^2 molecule^{-1})	Reference	Database	Notes
Chlorine Nitrate	14545-72-3		$ClONO_2$				0.079	0.078	**0.086**			**(Wagner and Birk, 2003)**						297		750-1320	5.8		H16	
Dinitrogen pentoxide	10102-03-1		O_2NONO_2				0.180	0.179	**0.185**			**(Wagner and Birk, 2003)**						294		540-1380	13.4		H16	
Peroxynitric acid	26404-66-0		$HOONO_2$				0.047	0.046	**0.051**			**(May and Friedl, 1993)**						220		780-830	1.7		H16	
Chlorine peroxide	12292-23-8		$ClOOCl$				0.007	0.007	**0.007**			**(Wetzel et al., 2010)**						250		500-835	0.7		H16	
Ammonia	7664-41-7		NH_3				0.057	0.056	**0.061**			**(Sharpe et al., 2004)**						296		540-3000	2.9		P	
Arsine	7784-42-1		AsH_3				0.021	0.020	**0.022**			**(Sharpe et al., 2004)**						296		600-3000	4.8		P	
Trichloroborone	10294-34-5		$BrCl_3$				0.296	0.288	**0.321**			**(Sharpe et al., 2004)**						296		600-3000	11.6		P	
Trifluoroborone	7637-07-2		BrF_3				0.018	0.019	**0.020**			**(Sharpe et al., 2004)**						296		600-3000	16.6		P	
Diborane	19287-45-7		$cyc\ (-BH_2-H-BH_2-H-)$				0.031	0.030	**0.034**			**(Sharpe et al., 2004)**						296		600-3000	13.2		P	
Dichloromethylphosphine	676-83-5		CH_3PCl_2				0.016	0.016	**0.018**			**(Sharpe et al., 2004)**						296		520-3000	1.4		P	
Carbon monoxide	630-08-0		CO				0.001	0.001	**0.001**			**(Sharpe et al., 2004)**						296		600-3000	1.0		P	
Germane	7782-65-2		GeH_4				0.143	0.140	**0.155**			**(Sharpe et al., 2004)**						296		600-3000	10.4		P	
Hydrogen cyanide	74-90-8	Prussic acid	HCN				0.012	0.012	**0.012**			**(Sharpe et al., 2004)**						296		550-3000	1.4		P	
Hydrogen peroxide	7722-84-1		$HOOH$				0.014	0.014	**0.015**			**(Sharpe et al., 2004)**						296		510-3000	2.0		P	
Hydrogen sulfide	7783-06-4		H_2S				<0.001	<0.001	**<0.001**			**(Sharpe et al., 2004)**						296		600-3000	0.0		P	
Iron pentacarbonyl	13463-40-6		$Fe(CO)_5$				0.089	0.092	**0.099**			**(Sharpe et al., 2004)**						296		530-3000	71.2		P	
Nickel carbonyl	13463-39-3		$Ni(CO)_4$				0.032	0.032	**0.037**			**(Sharpe et al., 2004)**						296		600-3000	37.3		P	
Phosphoric trichloride	10025-87-3	Phosphorous oxychloride	$P(O)Cl_3$				0.113	0.112	**0.104**			**(Sharpe et al., 2004)**						296		540-3000	9.1		P	
Titanium tetrachloride	7550-45-0		$TiCl_4$				0.018	0.017	**0.016**			**(Sharpe et al., 2004)**						296		500-3000	0.9		P	
Tungsten hexafluoride	7783-82-6		WF_6				0.093	0.092	**0.086**			**(Sharpe et al., 2004)**						296		600-3000	14.0		P	
Carbonyl Fluoride	353-50-4		CF_2O				0.109	0.109	**0.123**			**(Sharpe et al., 2004)**						296		550-3000	15.5		P	

Table S21. Structures of selected compounds

Identifier / Name	CASRN	Structure
trans-1,2-Dichlorohexafluorocyclobutane / E-R316c	3832-15-3	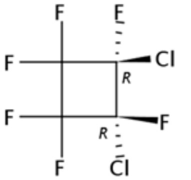
cis-1,2-Dichlorohexafluorocyclobutane / Z-R316c	3934-26-7	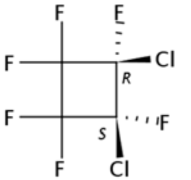
2-Ethoxy-3,3,4,4,5-pentafluorotetrahydro-2,5-bis[1,2,2,2-tetrafluoro-1-(trifluoromethyl)ethyl]-furan	920979-28-8	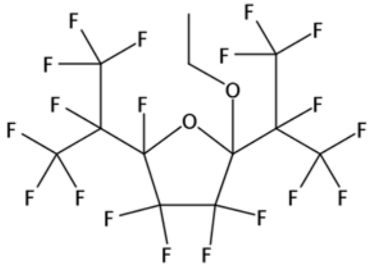
Perfluorodecalin (cis/trans), PFC-91-18	306-94-5	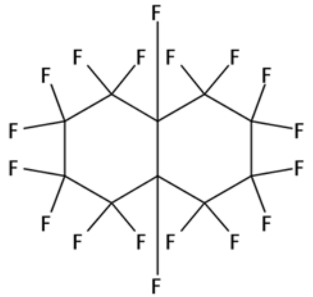
Perfluorodecalin (cis)	60433-11-6	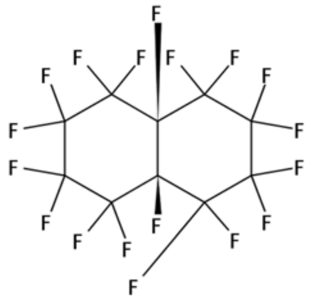

Perfluorodecalin (trans)	60433-12-7	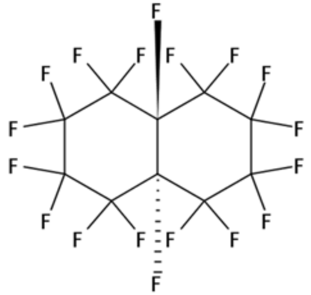
2-(Chloromethyl)oxirane	106-89-8	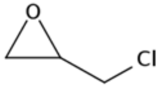
(1*S*,5*S*)-2,6,6-trimethylbicyclo[3.1.1]hept-2-ene	7785-26-4	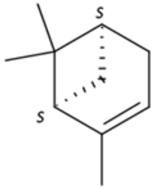
(1*S*,5*S*)-6,6-dimethyl-2-methylenebicyclo[3.1.1]heptane	18172-67-3	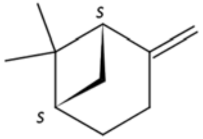
1-Methyl-4-(1-methylethenyl)-cyclohexene	138-86-3	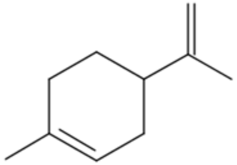
3,7,7-Trimethyl-bicyclo[4.1.0]hept-2-ene	554-61-0	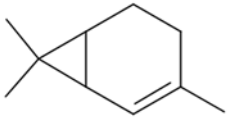
3,7,7-Trimethyl-bicyclo[4.1.0]hept-3-ene	13466-78-9	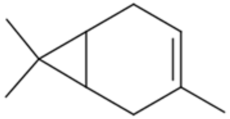
7-Methyl-3-methylene-1,6-octadiene	123-35-3	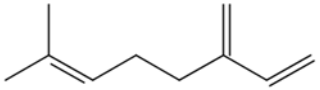

1-Ethyl-2-methylbenzene	611-14-3	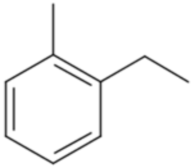
1-Ethyl-4-methylbenzene	622-96-8	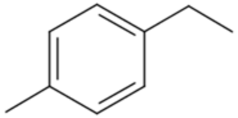
4-Vinylcyclohexene	100-40-3	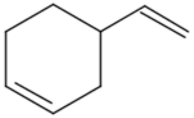
1-Ethyl-3-methylvenzene	620-14-4	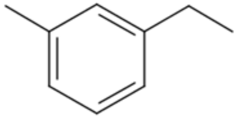
1,2,3,5-Tetramethylbenzene	527-53-7	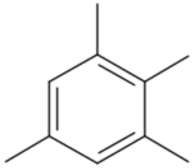
1,2,3,4-Tetramethylbenzene	488-23-3	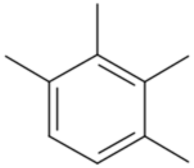
1,2,3,4-Tetrahydronaphthalene	119-64-2	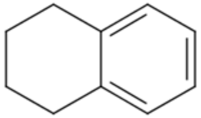
2,3-Dimethylfuran	14920-89-9	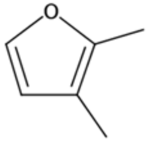
2,5-Dimethylfuran	625-86-5	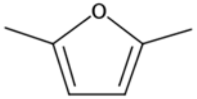
Furfuryl alcohol	98-00-0	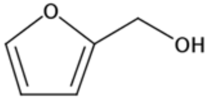

2-Methylfuran	534-22-5	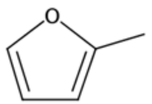
Isophorone	78-59-1	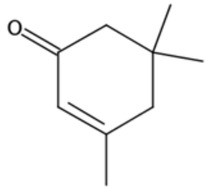
2-Pentylfuran	3777-69-3	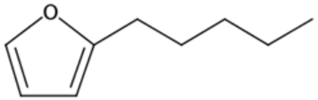
1-Methyl-4-(1-methylethenyl)-7-oxabicyclo[4.1.0]heptane	1195-92-2	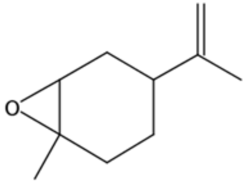
5-methyl-2-(1-methylethyl)-cyclohexanol	1490-04-6	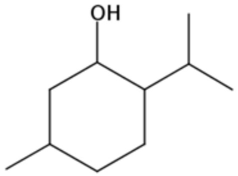
Methyl salicylate	119-36-8	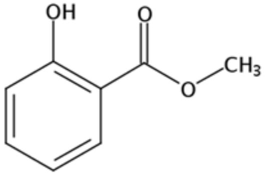
Guaiacol	90-05-1	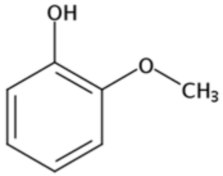
3-methylfuran	930-27-8	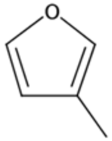

1,3,3-trimethyl-2-oxabicyclo[2.2.2]octane	470-82-6	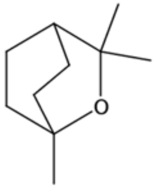
Ethyl benzoate	93-89-0	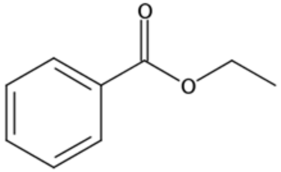
Furfural	98-01-1	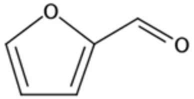
(2*E*)-3,7-dimethyl-2,6-octadien-1-ol	106-24-1	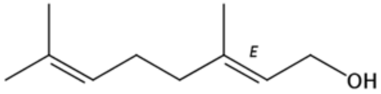
4_methyl-1,3-dioxolan-2-one	108-32-7	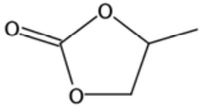
2-Ethyl-1-hexanol	104-76-7	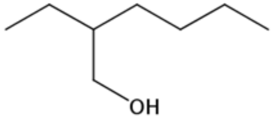
2-Ethoxyethyl acetate	111-15-9	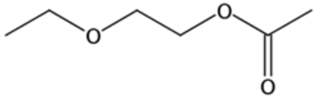
1,4-Dioxane	123-91-1	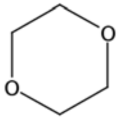
2-Methyloxirane	75-56-9	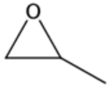
2-ethyloxirane	106-88-7	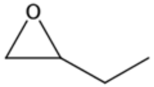

2-phenyl-oxirane	96-09-3	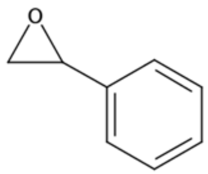
2-Methylbenzenamine	95-53-4	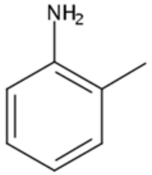
2-Methylaziridine	75-55-8	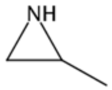
2-Vinylpyridine	100-69-6	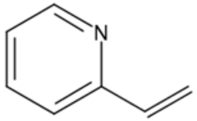
4-Methylpyridine	108-89-4	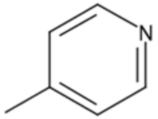
3-Methylpyridine	108-99-6	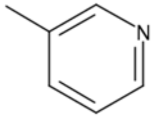
2-Methylpyridine	109-06-8	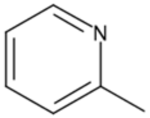
2,4-Diisocyanate-1-methylbenzene	584-84-9	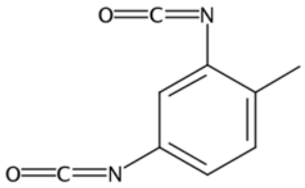
Morpholine	110-91-8	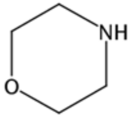

Quinoline	91-22-5	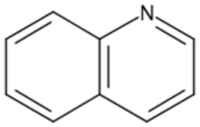
hexamethyldisiloxane	107-46-0	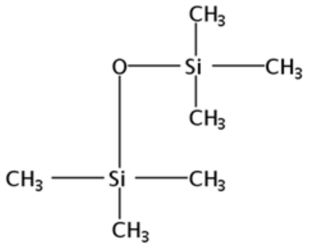
octamethyltrisiloxane	107-51-7	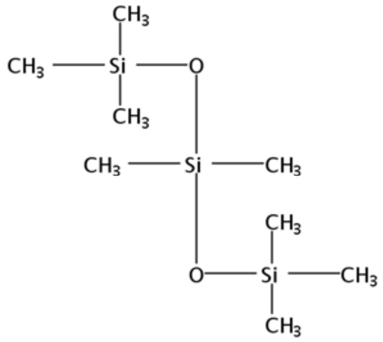
decamethyltetrasiloxane	141-62-8	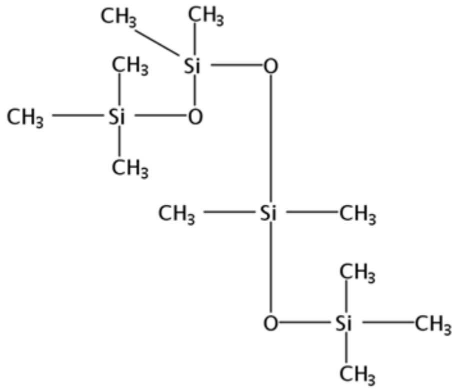

dodecamethylpentasiloxane	141-63-9	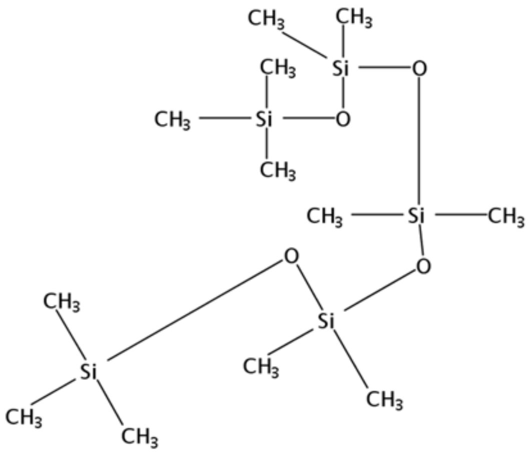
hexamethylcyclotrisiloxane	541-05-9	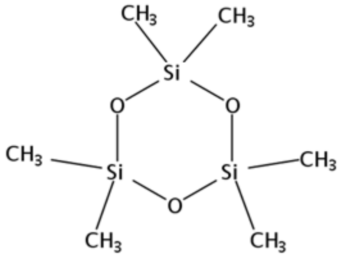
octamethylcyclotetrasiloxane	556-67-2	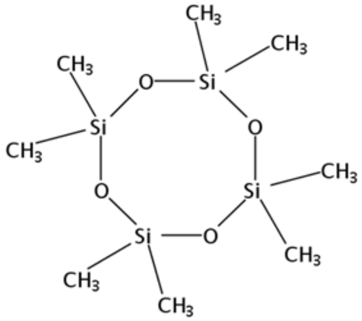

decamethylcyclopentasiloxane	541-02-6	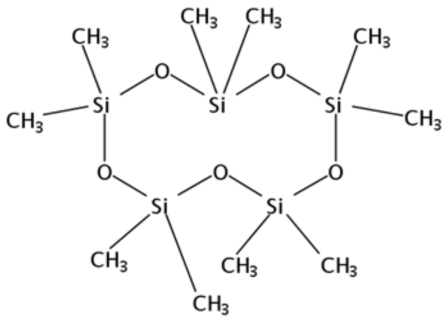
dodecamethylcyclohexasiloxane	540-97-6	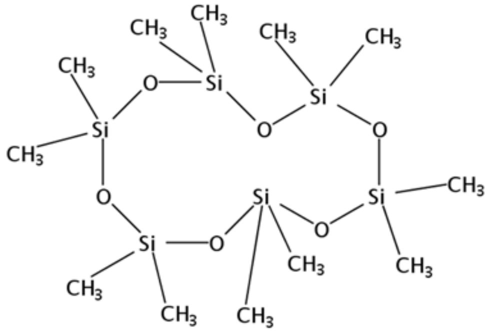

References

Acerboni, G., J. A. Beukes, N. R. Jensen, J. Hjorth, G. Myhre, C. J. Nielsen, and J. K. Sundet (2001), Atmospheric degradation and global warming potentials of three perfluoroalkenes, *Atmos. Environ.*, *35*(24), 4113-4123, doi: 10.1016/s1352-2310(01)00209-6.

Allen, G., J. J. Remedios, D. A. Newnham, K. M. Smith, and P. S. Monks (2005), Improved mid-infrared cross-sections for peroxyacetyl nitrate (PAN) vapour, *Atmos. Chem. Phys.*, *5*, 47-56, doi: 10.5194/acp-5-47-2005.

Anastasi, C., A. E. Heathfield, G. P. Knight, and F. Nicolaisen (1994), Integrated absorption-coefficients of $CHClF_2$ (HCFC-22) and CH_3Br in the atmospheric infrared window region, *Spectrochimica Acta Part a-Molecular and Biomolecular Spectroscopy*, *50*(10), 1791-1798, doi: 10.1016/0584-8539(94)00132-4.

Andersen, L. L., F. F. Østerstrøm, O. J. Nielsen, M. P. S. Andersen, and T. J. Wallington (2014), Atmospheric chemistry of (CF3)2CFOCH3, *Chem. Phys. Lett.*, *607*(Supplement C), 5-9, doi: https://doi.org/10.1016/j.cplett.2014.05.036.

Andersen, L. L., F. F. Østerstrøm, M. P. Sulbaek Andersen, O. J. Nielsen, and T. J. Wallington (2015), Atmospheric chemistry of cis-CF3CHCHCl (HCFO-1233zd(Z)): Kinetics of the gas-phase reactions with Cl atoms, OH radicals, and O3, *Chem. Phys. Lett.*, *639*(Supplement C), 289-293, doi: https://doi.org/10.1016/j.cplett.2015.09.008.

Andersen, M. P. S., S. B. Svendsen, F. F. Osterstrom, and O. J. Nielsen (2017a), Atmospheric Chemistry of CH3CH2OCH3: Kinetics and Mechanism of Reactions with Cl Atoms and OH Radicals, *Int. J. Chem. Kinet.*, *49*(1), 10-20, doi: 10.1002/kin.21051.

Andersen, M. P. S., D. R. Blake, F. S. Rowland, M. D. Hurley, and T. J. Wallington (2009), Atmospheric Chemistry of Sulfuryl Fluoride: Reaction with OH Radicals, CI Atoms and O_3, Atmospheric Lifetime, IR Spectrum, and Global Warming Potential, *Environ. Sci. Technol.*, *43*(4), 1067-1070, doi: 10.1021/es802439f.

Andersen, M. P. S., M. D. Hurley, V. F. Andersen, O. J. Nielsen, and T. J. Wallington (2010a), CHF_2OCHF_2 (HFE-134): IR Spectrum and Kinetics and Products of the Chlorine-Atom-Initiated Oxidation, *J. Phys. Chem. A*, *114*(14), 4963-4967, doi: 10.1021/jp101507f.

Andersen, M. P. S., V. F. Andersen, O. J. Nielsen, S. P. Sander, and T. J. Wallington (2010b), Atmospheric Chemistry of $HCF_2O(CF_2CF_2O)_xCF_2H$ (x=2-4): Kinetics and Mechanisms of the Chlorine-Atom-Initiated Oxidation, *Chemphyschem*, *11*(18), 4035-4041, doi: 10.1002/cphc.201000438.

Andersen, M. P. S., R. L. Waterland, S. P. Sander, O. J. Nielsen, and T. J. Wallington (2012a), Atmospheric chemistry of CxF2x+1CH=CH2 (x=1, 2, 4, 6 and 8): Radiative efficiencies and global warming potentials, *J. Photochem. Photobiol. A-Chem.*, *233*, 50-52, doi: 10.1016/j.jphotochem.2012.02.020.

Andersen, M. P. S., O. J. Nielsen, T. J. Wallington, B. Karpichev, and S. P. Sander (2012b), Assessing the Impact on Global Climate from General Anesthetic Gases, *Anesthesia and Analgesia*, *114*(5), 1081-1085, doi: 10.1213/ANE.0b013e31824d6150.

Andersen, M. P. S., M. Kyte, S. T. Andersen, C. J. Nielsen, and O. J. Nielsen (2017b), Atmospheric Chemistry of $(CF_3)_2CFCN$: A Replacement Compound for the Most Potent Industrial Greenhouse Gas, SF6, *Environ. Sci. Technol.*, *51*(3), 1321-1329, doi: 10.1021/acs.est.6b03758.

Andersen, M. P. S., E. J. K. Nilsson, O. J. Nielsen, M. S. Johnson, M. D. Hurley, and T. J. Wallington (2008), Atmospheric chemistry of trans-$CF_3CHCHCl$: Kinetics of the gas-phase reactions with Cl atoms, OH radicals, and O_3, *Journal of Photochemistry and Photobiology A: Chemistry*, *199*(1), 92-97, doi: 10.1016/j.jphotochem.2008.05.013.

Andersen, M. P. S., S. P. Sander, O. J. Nielsen, D. S. Wagner, T. J. Sanford, and T. J. Wallington (2010c), Inhalation anaesthetics and climate change, *British Journal of Anaesthesia*, *105*(6), 760-766, doi: 10.1093/bja/aeq259.

Andersen, M. P. S., M. D. Hurley, T. J. Wallington, F. Blandini, N. R. Jensen, V. Librando, and J. Hjorth (2004), Atmospheric chemistry of $CH_3O(CF_2CF_2O)_nCH_3$ (n=1-3): Kinetics and mechanism of oxidation initiated by Cl atoms and OH radicals, IR spectra, and global warmin potentials, *J. Phys. Chem. A*, *108*(11), 1964-1972, doi: 10.1021/jp036615a.

Antinolo, M., I. Bravo, E. Jimenez, B. Ballesteros, and J. Albaladejo (2017), Atmospheric Chemistry of E- and Z-CF_3CH=CHF (HFO-1234ze): OH Reaction Kinetics as a Function of Temperature and UV and IR Absorption Cross Sections, *J. Phys. Chem. A*, *121*(43), 8322-8331, doi: 10.1021/acs.jpca.7b06174.

Antinolo, M., R. del Olmo, I. Bravo, J. Albaladejo, and E. Jimenez (2019), Tropospheric fate of allyl cyanide (CH_2=$CHCH_2CN$): Kinetics, reaction products and secondary organic aerosol formation, *Atmos. Environ.*, *219*, 12, doi: 10.1016/j.atmosenv.2019.117041.

Antiñolo, M., E. Jimenez, and J. Albaladejo (2011), UV absorption cross sections between 230 and 350 nm and pressure dependence of the photolysis quantum yield at 308 nm of CF_3CH_2CHO, *Phys. Chem. Chem. Phys.*, *13*(35), 15936-15946, doi: 10.1039/c1cp21368g.

Antiñolo, M., E. Jimenez, and J. Albaladejo (2012a), Photochemistry of $CF_3(CH_2)_2CHO$ in air: UV absorption cross sections between 230 and 340 nm and photolysis quantum yields at 308 nm, *J. Photochem. Photobiol. A-Chem.*, *231*(1), 33-40, doi: 10.1016/j.jphotochem.2011.12.023.

Antiñolo, M., S. González, B. Ballesteros, J. Albaladejo, and E. Jiménez (2012b), Laboratory Studies of $CHF_2CF_2CH_2OH$ and $CF_3CF_2CH_2OH$: UV and IR Absorption Cross Sections and OH Rate Coefficients between 263 and 358 K, *The Journal of Physical Chemistry A*, doi: 10.1021/jp2111633.

Antiñolo, M., A. J. Ocaña, J. P. Aranguren, S. I. Lane, J. Albaladejo, and E. Jiménez (2017), Atmospheric degradation of 2-chloroethyl vinyl ether, allyl ether and allyl ethyl ether: Kinetics with OH radicals and UV photochemistry, *Chemosphere*, *181*(Supplement C), 232-240, doi: https://doi.org/10.1016/j.chemosphere.2017.04.053.

Ballard, J., R. J. Knight, and D. A. Newnham (2000a), Infrared absorption cross-sections and integrated absorption intensities of perfluoroethane and cis-perfluorocyclobutane, *J. Quant. Spectrosc. Radiat. Transf.*, *66*(2), 199-212, doi: 10.1016/s0022-4073(99)00217-4.

Ballard, J., R. J. Knight, D. A. Newnham, J. Vander Auwera, M. Herman, G. Di Lonardo, G. Masciarelli, F. M. Nicolaisen, J. A. Beukes, L. K. Christensen, R. McPheat, G. Duxbury, R. Freckleton, and K. P. Shine (2000b), An intercomparison of laboratory measurements of absorption cross-sections and integrated absorption intensities for HCFC-22, *J. Quant. Spectrosc. Radiat. Transf.*, *66*(2), 109-128, doi: 10.1016/s0022-4073(99)00211-3.

Barrera, J. A., P. R. Dalmasso, J. P. Aranguren Abrate, R. A. Taccone, and S. I. Lane (2015), Kinetic study of the OH and Cl-initiated oxidation, lifetimes and atmospheric acceptability indices of three halogenated ethenes, *RSC Advances*, *5*(90), 73501-73509, doi: 10.1039/C5RA13589C.

Barrow, G. M., and D. C. McKean (1952), The intensities of absorption bands in the methyl halides, *Proceedings of the Royal Society of London Series a-Mathematical and Physical Sciences*, *213*(1112), 27-41, doi: 10.1098/rspa.1952.0108.

Barry, J., G. Locke, D. Scollard, H. Sidebottom, J. Treacy, C. Clerbaux, R. Colin, and J. Franklin (1997), 1,1,1,3,3,-pentafluorobutane (HFC-365mfc): Atmospheric degradation and contribution to radiative forcing, *Int. J. Chem. Kinet.*, *29*(8), 607-617, doi: 10.1002/(sici)1097-4601(1997)29:8<607::aid-kin6>3.0.co;2-y.

Bernard, F., D. K. Papanastasiou, V. C. Papadimitriou, and J. B. Burkholder (2017), Infrared absorption spectra of linear (L-2-L-5) and cyclic (D-3-D-6) permethylsiloxanes, *J. Quant. Spectrosc. Radiat. Transf.*, *202*, 247-254, doi: 10.1016/j.jqsrt.2017.08.006.

Bernard, F., D. K. Papanastasiou, V. C. Papadimitriou, and J. B. Burkholder (2018), Infrared absorption spectra of $N(C_xF_{2x}+1)_3$ x=2-5 perfluoroamines, *J. Quant. Spectrosc. Radiat. Transf.*, *211*, 166-171, doi: 10.1016/j.jqsrt.2018.02.039.

Blazquez, S., M. Antinolo, O. J. Nielsen, J. Albaladejo, and E. Jimenez (2017), Reaction kinetics of $(CF_3)_2CFCN$ with OH radicals as a function of temperature (278-358 K): A good replacement for greenhouse SF_6?, *Chem. Phys. Lett.*, *687*, 297-302, doi: 10.1016/j.cplett.2017.09.039.

Bravo, I., Y. Diaz-de-Mera, A. Aranda, K. Smith, K. P. Shine, and G. Marston (2010a), Atmospheric chemistry of $C_4F_9OC_2H_5$ (HFE-7200), $C_4F_9OCH_3$ (HFE-7100), $C_3F_7OCH_3$ (HFE-7000) and $C_3F_7CH_2OH$: temperature dependence of the kinetics of their reactions with OH radicals, atmospheric lifetimes and global warming potentials, *Phys. Chem. Chem. Phys.*, *12*(19), 5115-5125, doi: 10.1039/b923092k.

Bravo, I., A. Rodríguez, D. Rodríguez, Y. Diaz-de-Mera, A. Notario, and A. Aranda (2013), Atmospheric Chemistry and Environmental Assessment of Inhalational Fluroxene, *ChemPhysChem*, *14*(16), 3834-3842, doi: 10.1002/cphc.201300559.

Bravo, I., A. Aranda, M. D. Hurley, G. Marston, D. R. Nutt, K. P. Shine, K. Smith, and T. J. Wallington (2010b), Infrared absorption spectra, radiative efficiencies, and global warming potentials of perfluorocarbons: Comparison between experiment and theory, *J. Geophys. Res.-Atmos.*, *115*(D24317), doi: 10.1029/2010jd014771.

Brodbeck, C., I. Rossi, H. Strapelias, and J. P. Bouanich (1980), Infrared spectral absorption intensities in the v_3 and v_4 regions of SF_6, *Chemical Physics*, *54*(1), 1-7, doi: 10.1016/0301-0104(80)80029-2.

Brown, L. R., C. B. Farmer, C. P. Rinsland, and R. A. Toth (1987), Molecular line parameters for the atmospheric trace molecule spectroscopy experiment, *Applied Optics*, *26*(23), 5154-5182.

Baasandorj, M., and J. B. Burkholder (2016), Rate Coefficient for the Gas-Phase OH + CHF=CF2 Reaction between 212 and 375 K, *Int. J. Chem. Kinet.*, *48*(11), 714-723, doi: 10.1002/kin.21027.

Baasandorj, M., A. R. Ravishankara, and J. B. Burkholder (2011), Atmospheric Chemistry of (Z)-$CF_3CH=CHCF_3$: OH Radical Reaction Rate Coefficient and Global Warming Potential, *J. Phys. Chem. A*, *115*(38), 10539-10549, doi: 10.1021/jp206195g.

Baasandorj, M., P. Marshall, R. L. Waterland, A. R. Ravishankara, and J. B. Burkholder (2018), Rate Coefficient Measurements and Theoretical Analysis of the OH + (E)-CF3CH=CHCF3 Reaction, *J. Phys. Chem. A*, *122*(19), 4635-4646, doi: 10.1021/acs.jpca.8b02771.

Baasandorj, M., G. Knight, V. C. Papadimitriou, R. K. Talukdar, A. R. Ravishankara, and J. B. Burkholder (2010), Rate Coefficients for the Gas-Phase Reaction of the Hydroxyl Radical with CH_2=CHF and CH_2= CF_2, *J. Phys. Chem. A*, *114*(13), 4619-4633, doi: 10.1021/jp100527z.

Cappellani, F., and G. Restelli (1992), Infrared band strengths and their temperature-dependence of the hydrohalocarbons HFC-134a, HFC-152a, HCFC-22, HCFC-123 and HCFC-142b, *Spectrochimica Acta Part a-Molecular and Biomolecular Spectroscopy*, *48*(8), 1127-1131, doi: 10.1016/0584-8539(92)80122-d.

Cavalli, F., M. Glasius, J. Hjorth, B. Rindone, and N. R. Jensen (1998), Atmospheric lifetimes, infrared spectra and degradation products of a series of hydrofluoroethers, *Atmos. Environ.*, *32*(21), 3767-3773, doi: 10.1016/s1352-2310(98)00106-x.

Chapados, C. (1988), Infrared-absorption of SF_6 from 32-cm^{-1} to 3000-cm^{-1} in the gaseous and liquid states, *J. Mol. Spectrosc.*, *132*(2), 323-351, doi: 10.1016/0022-2852(88)90329-3.

Charmet, A. P., N. Tasinato, P. Stoppa, A. Baldacci, and S. Giorgianni (2008), Jet-cooled diode laser spectrum and FTIR integrated band intensities of CF_3Br: rovibrational analysis of $2\nu_5$ and $\nu_2+\nu_3$ bands near 9 μm and cross-section measurements in the 450-2500 cm^{-1} region, *Molecular Physics*, *106*(9-10), 1171-1179, doi: 10.1080/00268970802026709.

Charmet, A. P., P. Stoppa, N. Tasinato, A. Baldan, S. Giorgianni, and A. Gambi (2010), Spectroscopic study of $CHBrF_2$ up to 9500 cm^{-1}: Vibrational analysis, integrated band intensities, and ab initio calculations, *J. Chem. Phys.*, *133*(4), doi: 10.1063/1.3460922.

Charmet, A. P., P. Stoppa, N. Tasinato, S. Giorgianni, V. Barone, M. Biczysko, J. Bloino, C. Cappelli, I. Carnimeo, and C. Puzzarini (2013), An integrated experimental and quantum-chemical investigation on the vibrational spectra of chlorofluoromethane, *J. Chem. Phys.*, *139*(16), 15, doi: 10.1063/1.4825380.

Christensen, L. K., T. J. Wallington, A. Guschin, and M. D. Hurley (1999), Atmospheric degradation mechanism of CF_3OCH_3, *J. Phys. Chem. A*, *103*(21), 4202-4208, doi: 10.1021/jp984455a.

Christensen, L. K., J. Sehested, O. J. Nielsen, M. Bilde, T. J. Wallington, A. Guschin, L. T. Molina, and M. J. Molina (1998), Atmospheric chemistry of HFE-7200 ($C_4F_9OC_2H_5$): Reaction with OH radicals and fate of $C_4F_9OCH_2CH_2O$(•) and C_4F_9OCHO(•)CH_3 radicals, *J. Phys. Chem. A*, *102*(25), 4839-4845, doi: 10.1021/jp981128u.

Christidis, N., M. D. Hurley, S. Pinnock, K. P. Shine, and T. J. Wallington (1997), Radiative forcing of climate change by CFC-11 and possible CFC-replacements, *J. Geophys. Res.-Atmos.*, *102*(D16), 19597-19609, doi: 10.1029/97jd01137.

Chu, P. M., F. R. Guenther, G. C. Rhoderick, and W. J. Lafferty (1999), The NIST quantitative infrared database, *J. Res. Natl. Inst. Stand. Technol.*, *104*(1), 59-81, doi: 10.6028/jres.104.004.

Clerbaux, C., and R. Colin (1994), Determination of the infrared cross-sections and global warming potentials of 1,1,2-trifluoroethane (HFC-143), *Geophys. Res. Lett.*, *21*(22), 2377-2380, doi: 10.1029/94gl02365.

Clerbaux, C., R. Colin, P. C. Simon, and C. Granier (1993), Infrared cross-sections and global warming potentials of 10 alternative hydrohalocarbons, *J. Geophys. Res.-Atmos.*, *98*(D6), 10491-10497, doi: 10.1029/93jd00390.

Cometto, P. M., R. A. Taccone, J. D. Nieto, P. R. Dalmasso, and S. I. Lane (2010), Kinetic Study of OH Radical Reactions with $CF_3CCl=CCl_2$, $CF_3CCl=CClCF_3$ and $CF_3CF=CFCF_3$, *Chemphyschem*, *11*(18), 4053-4059, doi: 10.1002/cphc.201000430.

D'Anna, B., S. R. Sellevag, K. Wirtz, and C. J. Nielsen (2005), Photolysis study of perfluoro-2-methyl-3-pentanone under natural sunlight conditions, *Environ. Sci. Technol.*, *39*(22), 8708-8711, doi: 10.1021/es048088u.

Dalmasso, P. R., R. A. Taccone, J. D. Nieto, M. A. Teruel, and S. I. Lane (2006), CH_3OCF_2CHFCl and CHF_2OCF_2CHFCl: Reaction with Cl atoms, atmospheric lifetimes, ozone depletion and global warming potentials, *Atmos. Environ.*, *40*(38), 7298-7307, doi: 10.1016/j.atmosenv.2006.06.031.

Davis, M. E., F. Bernard, M. R. McGillen, E. L. Fleming, and J. B. Burkholder (2016), UV and infrared absorption spectra, atmospheric lifetimes, and ozone depletion and global warming potentials for CCl2FCCl2F (CFC-112), CCl3CClF2 (CFC-112a), CCl3CF3 (CFC-113a), and CCl2FCF3 (CFC-114a), *Atmos. Chem. Phys.*, *16*(12), 8043-8052, doi: 10.5194/acp-16-8043-2016.

Di Lonardo, G., and G. Masciarelli (2000), Infrared absorption cross-sections and integrated absorption intensities of HFC-125 and HFC-143a, *J. Quant. Spectrosc. Radiat. Transf.*, *66*(2), 129-142.

Diaz-de-Mera, Y., A. Aranda, A. Notario, A. Rodriguez, D. Rodriguez, and I. Bravo (2015), Photolysis study of fluorinated ketones under natural sunlight conditions, *Phys. Chem. Chem. Phys.*, *17*(35), 22991-22998, doi: 10.1039/C5CP03527A.

Dickson, A. D., I. M. Mills, and B. Crawford (1957), Vibrational Intensities .8. CH3 and CD3 Chloride, Bromide, and Iodide, *J. Chem. Phys.*, *27*(2), 445-455, doi: 10.1063/1.1743744.

Dillon, T. J., A. Horowitz, and J. N. Crowley (2008), The atmospheric chemistry of sulphuryl fluoride, SO_2F_2, *Atmos. Chem. Phys.*, *8*(6), 1547-1557.

Drage, E. A., D. Jaksch, K. M. Smith, R. A. McPheat, E. Vasekova, and N. J. Mason (2006), FTIR spectroscopy and estimation of the global warming potential of CF_3Br and C_2F_4, *J. Quant. Spectrosc. Radiat. Transf.*, *98*(1), 44-56, doi: 10.1016/j.jqsrt.2005.05.071.

Dunn, D. S., K. Scanlon, and J. Overend (1982), The absolute intensities of the binary combination bands in the infrared-spectrum of SF_6, *Spectrochimica Acta Part a-Molecular and Biomolecular Spectroscopy*, *38*(8), 841-847, doi: 10.1016/0584-8539(82)80103-7.

Elkins, J. W., R. H. Kagann, and R. L. Sams (1984), Infrared band strengths for methyl-chloride in the regions of atmospheric interest, *J. Mol. Spectrosc.*, *105*(2), 480-490, doi: 10.1016/0022-2852(84)90235-2.

Etminan, M., E. Highwood, J. Laube, R. McPheat, G. Marston, K. Shine, and K. Smith (2014), Infrared Absorption Spectra, Radiative Efficiencies, and Global Warming Potentials of Newly-Detected Halogenated Compounds: CFC-113a, CFC-112 and HCFC-133a, *Atmosphere*, *5*(3), 473.

Fisher, D. A., C. H. Hales, W. C. Wang, M. K. W. Ko, and N. D. Sze (1990), Model-calculations of the relative effects of CFCs and their replacements on global warming, *Nature*, *344*(6266), 513-516, doi: 10.1038/344513a0.

Forster, P. M., J. B. Burkholder, C. Clerbaux, P. F. Coheur, M. Dutta, L. K. Gohar, M. D. Hurley, G. Myhre, R. W. Portmann, K. P. Shine, T. J. Wallington, and D. Wuebbles (2005), Resolution of the uncertainties in the radiative forcing of HFC-134a, *J. Quant. Spectrosc. Radiat. Transf.*, *93*(4), 447-460, doi: 10.1016/j.jqsrt.2004.08.038.

Freckleton, R. S., E. J. Highwood, K. P. Shine, O. Wild, K. S. Law, and M. G. Sanderson (1998), Greenhouse gas radiative forcing: Effects of averaging and inhomogeneities in trace gas distribution, *Quarterly Journal of the Royal Meteorological Society*, *124*(550), 2099-2127, doi: 10.1256/smsqj.55013.

Garland, N. L., L. J. Medhurst, and H. H. Nelson (1993), Potential chlorofluorocarbon replacements - OH reaction-rate constants between 250 and 315-K and infrared-absorption spectra, *J. Geophys. Res.-Atmos.*, *98*(D12), 23107-23111, doi: 10.1029/93jd02550.

Gierczak, T., M. Baasandorj, and J. B. Burkholder (2014), OH + (E)- and (Z)-1-Chloro-3,3,3-trifluoropropene-1 (CF3CH═CHCl) Reaction Rate Coefficients: Stereoisomer-Dependent Reactivity, *The Journal of Physical Chemistry A*, *118*(46), 11015-11025, doi: 10.1021/jp509127h.

Gierczak, T., R. K. Talukdar, J. B. Burkholder, R. W. Portmann, J. S. Daniel, S. Solomon, and A. R. Ravishankara (1996), Atmospheric fate and greenhouse warming potentials of HFC 236fa and HFC 236ea, *J. Geophys. Res.-Atmos.*, *101*(D8), 12905-12911, doi: 10.1029/96jd00059.

Godin, P. J., K. Le Bris, and K. Strong (2017a), Conformational analysis and global warming potentials of 1,1,1,3,3,3-hexafluoro-2-propanol from absorption spectroscopy, *J. Quant. Spectrosc. Radiat. Transf.*, *203*, 522-529, doi: 10.1016/j.jqsrt.2017.04.031.

Godin, P. J., A. Cabaj, L.-H. Xu, K. Le Bris, and K. Strong (2017b), A study of the temperature dependence of the infrared absorption cross-sections of 2,2,3,3,3-pentafluoropropanol in the range of 298–362K, *Journal of Quantitative Spectroscopy and Radiative Transfer*, *186*(Supplement C), 150-157, doi: https://doi.org/10.1016/j.jqsrt.2016.05.031.

Godin, P. J., A. Cabaj, S. Conway, A. C. Hong, K. Le Bris, S. A. Mabury, and K. Strong (2016), Temperature-dependent absorption cross-sections of perfluorotributylamine, *J. Mol. Spectrosc.*, *323*(Supplement C), 53-58, doi: https://doi.org/10.1016/j.jms.2015.11.004.

Gohar, L. K., G. Myhre, and K. P. Shine (2004), Updated radiative forcing estimates of four halocarbons, *J. Geophys. Res.-Atmos.*, *109*(D01107), doi: 10.1029/2003jd004320.

Golden, W. G., D. A. Horner, and J. Overend (1978), Intensities of binary overtone and combination bands in IR-spectrum of $CClF_3$, *J. Chem. Phys.*, *68*(3), 964-969, doi: 10.1063/1.435835.

González, S., E. Jiménez, and J. Albaladejo (2016), Assessment of the atmospheric loss processes initiated by OH radicals and sunlight, and the radiative efficiency for a series of hydrofluoroolefins, CF3(CF2)x=1,3,5CHCH2, *Chemosphere*, *151*(Supplement C), 45-54, doi: https://doi.org/10.1016/j.chemosphere.2016.02.025.

González, S., E. Jiménez, B. Ballesteros, E. Martínez, and J. Albaladejo (2015), Hydroxyl radical reaction rate coefficients as a function of temperature and IR absorption cross sections for CF3CH=CH2 (HFO-1243zf), potential replacement of CF3CH2F (HFC-134a), *Environmental Science and Pollution Research*, *22*(7), 4793-4805, doi: 10.1007/s11356-014-3426-2.

Good, D. A., J. S. Francisco, A. K. Jain, and D. J. Wuebbles (1998), Lifetimes and global warming potentials for dimethyl ether and for fluorinated ethers: CH_3OCF_3 (E143a), CHF_2OCHF_2 (E134), CHF_2OCF_3 (E125), *J. Geophys. Res.-Atmos.*, *103*(D21), 28181-28186, doi: 10.1029/98jd01880.

Goto, M., Y. Inoue, M. Kawasaki, A. G. Guschin, L. T. Molina, M. J. Molina, T. J. Wallington, and M. D. Hurley (2002), Atmospheric chemistry of HFE-7500 n-C3F7CF(OC2H5)CF(CF3)(2) : Reaction with OH radicals and Cl atoms and atmospheric fate of n-C3F7CF(OCHO center dot)CF(CF3)(2) and n-C3F7CF(OCH2CH2O center dot)CF(CF3)(2) radicals, *Environ. Sci. Technol.*, *36*(11), 2395-2402, doi: 10.1021/es0113798.

Graner, G. (1981), The methyl-bromide molecule - a critical consideration of perturbations in spectra, *J. Mol. Spectrosc.*, *90*(2), 394-438, doi: 10.1016/0022-2852(81)90136-3.

Grossman, A. S., K. E. Grant, W. E. Blass, and D. J. Wuebbles (1997), Radiative forcing calculations for CH_3Cl and CH_3Br, *J. Geophys. Res.-Atmos.*, *102*(D12), 13651-13656, doi: 10.1029/97jd00611.

Guo, Q., N. Zhang, T. Uchimaru, L. Chen, H. Quan, and J. Mizukado (2019), Atmospheric chemistry for gas-phase reactions of cyc-CF2CF2CF2CHXCHX- (X = H or F) with OH radicals in the temperature range of 253-328 K, *Atmos. Environ.*, *215*, 8, doi: 10.1016/j.atmosenv.2019.116895.

Hansen, J., M. Sato, and R. Ruedy (1997), Radiative forcing and climate response, *J. Geophys. Res.-Atmos.*, *102*(D6), 6831-6864.

Harrison, J. J. (2013), Infrared absorption cross sections for trifluoromethane, *J. Quant. Spectrosc. Radiat. Transf.*, *130*, 359-364, doi: 10.1016/j.jqsrt.2013.05.026.

Harrison, J. J. (2015a), New and improved infrared absorption cross sections for dichlorodifluoromethane (CFC-12), *Atmospheric Measurement Techniques*, *8*(8), 3197-3207, doi: 10.5194/amt-8-3197-2015.

Harrison, J. J. (2015b), Infrared absorption cross sections for 1,1,1,2-tetrafluoroethane, *J. Quant. Spectrosc. Radiat. Transf.*, *151*, 210-216, doi: 10.1016/j.jqsrt.2014.09.023.

Harrison, J. J. (2016), New and improved infrared absorption cross sections for chlorodifluoromethane (HCFC-22), *Atmospheric Measurement Techniques*, *9*(6), 2593-2601, doi: 10.5194/amt-9-2593-2016.

Harrison, J. J. (2019), Infrared absorption cross sections for air-broadened 1,1-dichloro-1-fluoroethane (HCFC-141b), *Journal of Quantitative Spectroscopy and Radiative Transfer*, *238*, 106489, doi: https://doi.org/10.1016/j.jqsrt.2019.04.041.

Harrison, J. J., and P. F. Bernath (2010), Infrared absorption cross sections for propane (C3H8) in the 3 mu m region, *J. Quant. Spectrosc. Radiat. Transf.*, *111*(9), 1282-1288, doi: 10.1016/j.jqsrt.2009.11.027.

Harrison, J. J., and P. F. Bernath (2012), Mid- and long-wave infrared absorption cross sections for acetonitrile, *J. Quant. Spectrosc. Radiat. Transf.*, *113*(3), 221-225, doi: 10.1016/j.jqsrt.2011.11.003.

Harrison, J. J., N. D. C. Allen, and P. F. Bernath (2010), Infrared absorption cross sections for ethane (C2H6) in the 3 mu m region, *J. Quant. Spectrosc. Radiat. Transf.*, *111*(3), 357-363, doi: 10.1016/j.jqsrt.2009.09.010.

Harrison, J. J., N. D. C. Allen, and P. F. Bernath (2011), Infrared absorption cross sections for acetone (propanone) in the 3 mu m region, *J. Quant. Spectrosc. Radiat. Transf.*, *112*(1), 53-58, doi: 10.1016/j.jqsrt.2010.08.011.

Harrison, J. J., C. D. Boone, and P. F. Bernath (2017), New and improved infra-red absorption cross sections and ACE-FTS retrievals of carbon tetrachloride (CCl4), *Journal of Quantitative Spectroscopy and Radiative Transfer*, *186*(Supplement C), 139-149, doi: https://doi.org/10.1016/j.jqsrt.2016.04.025.

Hashikawa, Y., M. Kawasaki, R. L. Waterland, M. D. Hurley, J. C. Ball, T. J. Wallington, M. P. S. Andersen, and O. J. Nielsen (2004), Gas phase UV and IR absorption spectra of CxF2x+1CHO (x=1-4), *J. Fluor. Chem.*, *125*(12), 1925-1932, doi: 10.1016/j.jfluchem.2004.07.006.

Heathfield, A. E., C. Anastasi, A. McCulloch, and F. M. Nicolaisen (1998), Integrated infrared absorption coefficients of several partially fluorinated ether compounds: CF_3OCF_2H, CF_2HOCF_2H, $CH_3OCF_2CF_2H$, CH_3OCF_2CFClH, $CH_3CH_2OCF_2CF_2H$, $CF_3CH_2OCF_2CF_2H$ and CH_2=$CHCH_2OCF_2CF_2H$, *Atmos. Environ.*, *32*(16), 2825-2833, doi: 10.1016/s1352-2310(97)00462-7.

Herath, T. N., E. C. Clinch, I. Orozco, E. L. Raign, and P. Marshall (2016), Relative Rate and Product Studies of the Reactions of Atomic Chlorine with Tetrafluoroethylene, 1,2-Dichloro-1,2-difluoroethylene, 1,1-Dichloro-2,2-difluoroethylene, and Hexafluoro-1,3-butadiene in the Presence of Oxygen, *The Journal of Physical Chemistry A*, *120*(37), 7311-7319, doi: 10.1021/acs.jpca.6b05305.

Highwood, E. J., and K. P. Shine (2000), Radiative forcing and global warming potentials of 11 halogenated compounds, *J. Quant. Spectrosc. Radiat. Transf.*, *66*(2), 169-183, doi: 10.1016/s0022-4073(99)00215-0.

Highwood, E. J., K. P. Shine, M. D. Hurley, and T. J. Wallington (1999), Estimation of direct radiative forcing due to non-methane hydrocarbons, *Atmos. Environ.*, *33*(5), 759-767, doi: 10.1016/s1352-2310(98)00220-9.

Hodnebrog, Ø., S. B. Dalsoren, and G. Myhre (2018), Lifetimes, direct and indirect radiative forcing, and global warming potentials of ethane (C2H6), propane (C3H8), and butane (C4H10), *Atmospheric Science Letters*, *19*(2), 7, doi: 10.1002/asl.804.

Hodnebrog, Ø., M. Etminan, J. S. Fuglestvedt, G. Marston, G. Myhre, C. J. Nielsen, K. P. Shine, and T. J. Wallington (2013), Global Warming Potentials and Radiative Efficiencies of Halocarbons and Related Compounds: A Comprehensive Review, *Reviews of Geophysics*, *51*(2), 300-378, doi: 10.1002/rog.20013.

Hong, A. C., C. J. Young, M. D. Hurley, T. J. Wallington, and S. A. Mabury (2013), Perfluorotributylamine: A novel long-lived greenhouse gas, *Geophys. Res. Lett.*, *40*(22), 6010-6015, doi: 10.1002/2013GL058010.

Hurley, M. D., J. C. Ball, and T. J. Wallington (2007), Atmospheric chemistry of the *Z* and *E* isomers of $CF_3CF{=}CHF$; Kinetics, mechanisms, and products of gas-phase reactions with Cl atoms, OH radicals, and O_3, *J. Phys. Chem. A*, *111*(39), 9789-9795, doi: 10.1021/jp0753530.

Hurley, M. D., T. Wallington, G. Buchanan, L. Gohar, G. Marston, and K. Shine (2005), IR spectrum and radiative forcing of CF_4 revisited, *J. Geophys. Res.-Atmos.*, *110*(D2), doi: 10.1029/2004jd005201.

Imasu, R., A. Suga, and T. Matsuno (1995), Radiative effects and halocarbon global warming potentials of replacement compounds for chlorofluorocarbons, *Journal of the Meteorological Society of Japan*, *73*(6), 1123-1136.

Inoue, Y., M. Kawasaki, T. J. Wallington, and M. D. Hurley (2008), Atmospheric chemistry of $CF_3CH_2CF_2CH_3$ (HFC-365mfc): Kinetics and mechanism of chlorine atom initiated oxidation, infrared spectrum, and global warming potential, *Chem. Phys. Lett.*, *462*(4-6), 164-168, doi: 10.1016/j.cplett.2008.07.054.

Ivy, D. J., M. Rigby, M. Baasandorj, J. B. Burkholder, and R. G. Prinn (2012), Global emission estimates and radiative impact of C_4F_{10}, C_5F_{12}, C_6F_{14}, C_7F_{16} and C_8F_{18}, *Atmos. Chem. Phys.*, *12*, 7635-7645, doi: 10.5194/acp-12-7635-2012.

Jain, A. K., B. P. Briegleb, K. Minschwaner, and D. J. Wuebbles (2000), Radiative forcings and global warming potentials of 39 greenhouse gases, *J. Geophys. Res.-Atmos.*, *105*(D16), 20773-20790, doi: 10.1029/2000jd900241.

Jain, A. K., Z. J. Li, V. Naik, D. J. Wuebbles, D. A. Good, J. C. Hansen, and J. S. Francisco (2001), Evaluation of the atmospheric lifetime and radiative forcing on climate for 1,2,2,2-Tetrafluoroethyl Trifluoromethyl Ether ($CF_3OCHFCF_3$), *J. Geophys. Res.-Atmos.*, *106*(D12), 12615-12618, doi: 10.1029/2001jd900013.

Jara-Toro, R. A., J. A. Barrera, J. P. Aranguren-Abrate, R. A. Taccone, and G. A. Pino (2020), Rate Coefficient and Mechanism of the OH-Initiated Degradation of 1-Chlorobutane: Atmospheric Implications, *J. Phys. Chem. A*, *124*(1), 229-239, doi: 10.1021/acs.jpca.9b10426.

Javadi, M. S., O. J. Nielsen, T. J. Wallington, M. D. Hurley, and J. G. Owens (2007), Atmospheric chemistry of 2-ethoxy-3,3,4,4,5-pentafluorotetra-hydro-2,5-bis 1,2,2,2-tetrafluoro-1- (trifluoromethyl)ethyl -furan: Kinetics, mechanisms, and products of Cl atom and OH radical initiated oxidation, *Environ. Sci. Technol.*, *41*(21), 7389-7395, doi: 10.1021/es071175c.

Jia, X., L. Chen, J. Mizukado, S. Kutsuna, and K. Tokuhashi (2013), Rate constants for the gas-phase reactions of cyclo-CXCXCF2CF2– (X=H, F) with OH radicals at a temperature range of 253–328K, *Chem. Phys. Lett.*, *572*(Supplement C), 21-25, doi: https://doi.org/10.1016/j.cplett.2013.04.020.

Jimenez, E., M. Antinolo, B. Ballesteros, E. Martinez, and J. Albaladejo (2010), Atmospheric Lifetimes and Global Warming Potentials of $CF_3CH_2CH_2OH$ and $CF_3(CH_2)_2CH_2OH$, *Chemphyschem*, *11*(18), 4079-4087, doi: 10.1002/cphc.201000365.

Jiménez, E., S. González, M. Cazaunau, H. Chen, B. Ballesteros, V. Daële, J. Albaladejo, and A. Mellouki (2016), Atmospheric Degradation Initiated by OH Radicals of the Potential Foam Expansion Agent, CF3(CF2)2CH═CH2 (HFC-1447fz): Kinetics and Formation of Gaseous Products and Secondary Organic Aerosols, *Environ. Sci. Technol.*, *50*(3), 1234-1242, doi: 10.1021/acs.est.5b04379.

Johnson, T. J., L. T. M. Profeta, R. L. Sams, D. W. T. Griffith, and R. L. Yokelson (2010), An infrared spectral database for detection of gases emitted by biomass burning, *Vib. Spectrosc.*, *53*(1), 97-102, doi: 10.1016/j.vibspec.2010.02.010.

Jubb, A. M., T. Gierczak, M. Baasandorj, R. L. Waterland, and J. B. Burkholder (2014), Methyl-Perfluoroheptene-Ethers (CH3OC7F13): Measured OH Radical Reaction Rate Coefficients for Several Isomers and Enantiomers and Their Atmospheric Lifetimes and Global Warming Potentials, *Environ. Sci. Technol.*, *48*(9), 4954-4962, doi: 10.1021/es500888v.

Kagann, R. H., J. W. Elkins, and R. L. Sams (1983), Absolute band strengths of halocarbons F-11 and F-12 in the 8-mu-mu to 16-mu-m region, *Journal of Geophysical Research-Oceans and Atmospheres*, *88*(NC2), 1427-1432, doi: 10.1029/JC088iC02p01427.

Kim, K., and W. T. King (1984), Infrared intensities in chloroform, *J. Chem. Phys.*, *80*(3), 978-982, doi: 10.1063/1.446792.

Kim, K., R. S. McDowell, and W. T. King (1980), Integrated infrared intensities and transition moments in SF_6, *J. Chem. Phys.*, *73*(1), 36-41, doi: 10.1063/1.439883.

Ko, M., R. L. Shia, N. D. Sze, H. Magid, and R. G. Bray (1999), Atmospheric lifetime and global warming potential of HFC-245fa, *J. Geophys. Res.-Atmos.*, *104*(D7), 8173-8181, doi: 10.1029/1998jd100097.

Ko, M., N. D. Sze, W. C. Wang, G. Shia, A. Goldman, F. J. Murcray, D. G. Murcray, and C. P. Rinsland (1993), Atmospheric sulfur-hexafluoride - sources, sinks and greenhouse warming, *J. Geophys. Res.-Atmos.*, *98*(D6), 10499-10507, doi: 10.1029/93jd00228.

Kovács, T., W. Feng, A. Totterdill, J. M. C. Plane, S. Dhomse, J. C. Gómez-Martín, G. P. Stiller, F. J. Haenel, C. Smith, P. M. Forster, R. R. García, D. R. Marsh, and M. P. Chipperfield (2017), Determination of the atmospheric lifetime and global warming potential of sulfur hexafluoride using a three-dimensional model, *Atmos. Chem. Phys.*, *17*(2), 883-898, doi: 10.5194/acp-17-883-2017.

Le Bris, K., and K. Strong (2010), Temperature-dependent absorption cross-sections of HCFC-142b, *J. Quant. Spectrosc. Radiat. Transf.*, *111*(3), 364-371, doi: 10.1016/j.jqsrt.2009.10.005.

Le Bris, K., and L. Graham (2015), Quantitative comparisons of absorption cross-section spectra and integrated intensities of HFC-143a, *Journal of Quantitative Spectroscopy and Radiative Transfer*, *151*(Supplement C), 13-17, doi: https://doi.org/10.1016/j.jqsrt.2014.09.005.

Le Bris, K., R. Pandharpurkar, and K. Strong (2011), Mid-infrared absorption cross-sections and temperature dependence of CFC-113, *J. Quant. Spectrosc. Radiat. Transf.*, *112*(8), 1280-1285, doi: 10.1016/j.jqsrt.2011.01.023.

Le Bris, K., J. McDowell, and K. Strong (2012), Measurements of the infrared absorption cross-sections of HCFC-141b (CH3CFCl2), *J. Quant. Spectrosc. Radiat. Transf.*, *113*(15), 1913-1919, doi: 10.1016/j.jqsrt.2012.05.004.

Le Bris, K., J. DeZeeuw, P. J. Godin, and K. Strong (2017), Cis- and trans-perfluorodecalin: Infrared spectra, radiative efficiency and global warming potential, *J. Quant. Spectrosc. Radiat. Transf.*, *203*, 538-541, doi: 10.1016/j.jqsrt.2017.01.011.

Le Bris, K., J. DeZeeuw, P. J. Godin, and K. Strong (2018), Infrared absorption cross-sections, radiative efficiency and global warming potential of HFC-43-10mee, *J. Mol. Spectrosc.*, *348*, 64-67, doi: 10.1016/j.jms.2017.06.004.

Le Bris, K., J. DeZeeuw, P. J. Godin, and K. Strong (2020), Radiative efficiency and global warming potential of the hydrofluoroether HFE-356mec3 (CH3OCF2CHFCF3) from experimental and theoretical infrared absorption cross-sections, *J. Mol. Spectrosc.*, *367*, 5, doi: 10.1016/j.jms.2019.111241.

Li, Z. H., and P. Varanasi (1994), Measurement of the absorption cross-sections of CFC-11 at conditions representing various model atmospheres, *J. Quant. Spectrosc. Radiat. Transf.*, *52*(2), 137-144, doi: 10.1016/0022-4073(94)90002-7.

Lindenmaier, R., S. D. Williams, R. L. Sams, and T. J. Johnson (2017), Quantitative Infrared Absorption Spectra and Vibrational Assignments of Crotonaldehyde and Methyl Vinyl Ketone Using Gas-Phase Mid-Infrared, Far-Infrared, and Liquid Raman Spectra: s-cis vs s-trans Composition Confirmed via Temperature Studies and ab Initio Methods, *The Journal of Physical Chemistry A*, *121*(6), 1195-1212, doi: 10.1021/acs.jpca.6b10872.

Lindsay, L. P., and P. N. Schatz (1964), Absolute infrared intensities in CHBr3(l), CHBr3(g) and CCl4(g), *Spectrochimica Acta*, *20*(9), 1421-1429, doi: 10.1016/0371-1951(64)80123-5.

Liu, D., S. Qin, W. Li, D. Zhang, and Z. Guo (2016), Atmospheric Chemistry of 1H-Heptafluorocyclopentene (cyc-CF2CF2CF2CF═CH−): Rate Constant, Products, and Mechanism of Gas-Phase Reactions with OH Radicals, IR Absorption Spectrum, Photochemical Ozone Creation Potential, and Global Warming Potential, *The Journal of Physical Chemistry A*, *120*(48), 9557-9563, doi: 10.1021/acs.jpca.6b10348.

M.S.F./R.A.L. Molecular Spectroscopy Facility / Rutherford Appleton Laboratory, http://www.msf.rl.ac.uk.

Mashino, M., M. Kawasaki, T. J. Wallington, and M. D. Hurley (2000), Atmospheric degradation of $CF_3OCF{=}CF_2$: Kinetics and mechanism of its reaction with OH radicals and Cl atoms, *J. Phys. Chem. A*, *104*(13), 2925-2930, doi: 10.1021/jp9942264.

Massie, S. T., A. Goldman, D. G. Murcray, and J. C. Gille (1985), Approximate absorption cross-sections of F12, F11, C10NO$_2$, N_2O_5, HNO_3, CCl_4, CF_4, F21, F113, F114, and HNO_4, *Applied Optics*, *24*(21), 3426-3427.

Massie, S. T., A. Goldman, A. H. McDaniel, C. A. Cantrell, J. A. Davidson, R. E. Shetter, and J. G. Calvert (1991), Temperature dependent infrared cross sections for CFC-11, CFC-12, CFC-13, CFC-14, CFC-22, CFC-113, CFC-114, and CFC-115*Rep.*, 67 pp, NCAR Tech. Note NCAR/TN-358+STR, Nat. Cent. for Atmos. Res., Boulder, Colorado.

May, R. D., and R. R. Friedl (1993), Integrated band intensities of HO2NO2 at 220-K, *J. Quant. Spectrosc. Radiat. Transf.*, *50*(3), 257-266, doi: 10.1016/0022-4073(93)90076-t.

McDaniel, A. H., C. A. Cantrell, J. A. Davidson, R. E. Shetter, and J. G. Calvert (1991), The temperature-dependent, infrared-absorption cross-sections for the chlorofluorocarbons - CFC-11, CFC-12, CFC-13, CFC-14, CFC-22, CFC-113, CFC-114, and CFC-115, *Journal of Atmospheric Chemistry*, *12*(3), 211-227, doi: 10.1007/bf00048074.

McDowell, R. S., B. J. Krohn, H. Flicker, and M. C. Vasquez (1986), Vibrational levels and anharmonicity in SF_6 .1. Vibrational band analysis, *Spectrochimica Acta Part a-Molecular and Biomolecular Spectroscopy*, *42*(2-3), 351-369, doi: 10.1016/0584-8539(86)80199-4.

McGillen, M. R., F. Bernard, E. L. Fleming, and J. B. Burkholder (2015), HCFC-133a (CF3CH2Cl): OH rate coefficient, UV and infrared absorption spectra, and atmospheric implications, *Geophys. Res. Lett.*, *42*(14), 6098-6105, doi: 10.1002/2015GL064939.

McPheat, R., and G. Duxbury (2000), Infrared absorption cross-sections and integrated absorption intensities of chloroform and fluoroform vapour, *J. Quant. Spectrosc. Radiat. Transf.*, *66*(2), 153-167, doi: 10.1016/s0022-4073(99)00214-9.

Mills, I. M., W. B. Person, J. R. Scherer, and B. Crawford (1958), Vibrational intensities .9. C_2F_6 - extension and revision, *J. Chem. Phys.*, *28*(5), 851-853, doi: 10.1063/1.1744282.

Minschwaner, K., R. W. Carver, B. P. Briegleb, and A. E. Roche (1998), Infrared radiative forcing and atmospheric lifetimes of trace species based on observations from UARS, *J. Geophys. Res.-Atmos.*, *103*(D18), 23243-23253, doi: 10.1029/98jd02116.

Molina, L. T., P. J. Woolridge, and M. J. Molina (1995), Atmospheric reactions and ultraviolet and infrared absorptivities of nitrogen trifluoride, *Geophys. Res. Lett.*, *22*(14), 1873-1876, doi: 10.1029/95gl01669.

Morcillo, J., L. J. Zamorano, and J. M. V. Heredia (1966), Infra-red intensities in CH_2F_2, CH_2Cl_2 and CF_2Cl_2, *Spectrochimica Acta*, *22*(12), 1969-1980, doi: 10.1016/0371-1951(66)80048-6.

Moreno, A., M. P. Gallego-Iniesta, R. Taccone, M. P. Martín, B. Cabañas, and M. S. Salgado (2014), FTIR gas-phase kinetic study on the reactions of some acrylate esters with OH radicals and Cl atoms, *Environmental Science and Pollution Research*, *21*(19), 11541-11551, doi: 10.1007/s11356-014-3112-4.

Myhre, G., and F. Stordal (1997), Role of spatial and temporal variations in the computation of radiative forcing and GWP, *J. Geophys. Res.-Atmos.*, *102*(D10), 11181-11200, doi: 10.1029/97jd00148.

Myhre, G., E. J. Highwood, K. P. Shine, and F. Stordal (1998), New estimates of radiative forcing due to well mixed greenhouse gases, *Geophys. Res. Lett.*, *25*(14), 2715-2718, doi: 10.1029/98gl01908.

Myhre, G., C. J. Nielsen, D. L. Powell, and F. Stordal (1999), Infrared absorption cross section, radiative forcing, and GWP of four hydrofluoro(poly)ethers, *Atmos. Environ.*, *33*(27), 4447-4458, doi: 10.1016/s1352-2310(99)00208-3.

Myhre, G., F. Stordal, I. Gausemel, C. J. Nielsen, and E. Mahieu (2006), Line-by-line calculations of thermal infrared radiation representative for global condition: CFC-12 as an example, *J. Quant. Spectrosc. Radiat. Transf.*, *97*(3), 317-331, doi: 10.1016/j.jqsrt.2005.04.015.

Naik, V., A. K. Jain, K. O. Patten, and D. J. Wuebbles (2000), Consistent sets of atmospheric lifetimes and radiative forcings on climate for CFC replacements: HCFCs and HFCs, *J. Geophys. Res.-Atmos.*, *105*(D5), 6903-6914, doi: 10.1029/1999jd901128.

Nanes, R., P. M. Silvaggio, and R. W. Boese (1980), Temperature-dependence of intensities of the 8-12 μm bands of $CFCl_3$, *J. Quant. Spectrosc. Radiat. Transf.*, *23*(2), 211-220, doi: 10.1016/0022-4073(80)90008-4.

Nemtchinov, V., and P. Varanasi (2003), Thermal infrared absorption cross-sections of CCl_4 needed for atmospheric remote sensing, *J. Quant. Spectrosc. Radiat. Transf.*, *82*(1-4), 473-481, doi: 10.1016/s0022-4073(03)00171-7.

Nemtchinov, V., and P. Varanasi (2004), Absorption cross-sections of HFC-134a in the spectral region between 7 and 12 μm, *J. Quant. Spectrosc. Radiat. Transf.*, *83*(3-4), 285-294, doi: 10.1016/s0022-4073(02)00356-4.

Newnham, D., J. Ballard, and M. Page (1996), Infrared band strengths of HFC-134a vapour, *J. Quant. Spectrosc. Radiat. Transf.*, *55*(3), 373-381, doi: 10.1016/0022-4073(95)00164-6.

Nielsen, O. J., F. M. Nicolaisen, C. Bacher, M. D. Hurley, T. J. Wallington, and K. P. Shine (2002), Infrared spectrum and global warming potential of SF_5CF_3, *Atmos. Environ.*, *36*(7), 1237-1240, doi: 10.1016/s1352-2310(01)00551-9.

Nielsen, O. J., M. S. Javadi, M. P. S. Andersen, M. D. Hurley, T. J. Wallington, and R. Singh (2007), Atmospheric chemistry of $CF_3CF=CH_2$: Kinetics and mechanisms of gas-phase reactions with Cl atoms, OH radicals, and O_3, *Chem. Phys. Lett.*, *439*(1-3), 18-22, doi: 10.1016/j.cplett.2007.03.053.

Nilsson, E. J. K., O. J. Nielsen, M. S. Johnson, M. D. Hurley, and T. J. Wallington (2009), Atmospheric chemistry of cis-CF_3CHCHF: Kinetics of reactions with OH radicals and O_3 and products of OH radical initiated oxidation, *Chem. Phys. Lett.*, *473*(4-6), 233-237, doi: 10.1016/j.cplett.2009.03.076.

Ninomiya, Y., M. Kawasaki, A. Guschin, L. T. Molina, M. J. Molina, and T. J. Wallington (2000), Atmospheric chemistry of *n*-$C_3F_7OCH_3$: Reaction with OH radicals and Cl atoms and atmospheric fate of *n*-$C_3F_7OCH_2O$(•) radicals, *Environ. Sci. Technol.*, *34*(14), 2973-2978, doi: 10.1021/es991449z.

Olliff, M. P., and G. Fischer (1994), Integrated absorption intensities of haloethanes and halopropanes, *Spectrochimica Acta Part a-Molecular and Biomolecular Spectroscopy*, *50*(13), 2223-2237, doi: 10.1016/0584-8539(93)e0027-t.

Orkin, V. L., L. E. Martynova, and A. N. Ilichev (2010), High-Accuracy Measurements of OH Reaction Rate Constants and IR Absorption Spectra: CH_2=CF-CF_3 and trans-CHF=CH-CF_3, *J. Phys. Chem. A*, *114*(19), 5967-5979, doi: 10.1021/jp9092817.

Orkin, V. L., L. E. Martynova, and M. J. Kurylo (2014a), Photochemical Properties of trans-1-Chloro-3,3,3-trifluoropropene (trans-CHCl══CHCF3): OH Reaction Rate Constant, UV and IR Absorption Spectra, Global Warming Potential, and Ozone Depletion Potential, *The Journal of Physical Chemistry A*, *118*(28), 5263-5271, doi: 10.1021/jp5018949.

Orkin, V. L., V. G. Khamaganov, and A. G. Guschin (2014b), Photochemical Properties of Hydrofluoroethers CH_3OCHF_2, CH_3OCF_3, and $CHF_2OCH_2CF_3$: Reactivity toward OH, IR Absorption Cross Sections, Atmospheric Lifetimes, and Global Warming Potentials, *The Journal of Physical Chemistry A*, *118*(45), 10770-10777, doi: 10.1021/jp506377w.

Orkin, V. L., E. Villenave, R. E. Huie, and M. J. Kurylo (1999), Atmospheric lifetimes and global warming potentials of hydrofluoroethers: Reactivity toward OH, UV spectra, and IR absorption cross sections, *J. Phys. Chem. A*, *103*(48), 9770-9779, doi: 10.1021/jp991741t.

Orkin, V. L., A. G. Guschin, I. K. Larin, R. E. Huie, and M. J. Kurylo (2003), Measurements of the infrared absorption cross-sections of haloalkanes and their use in a simplified calculational approach for estimating direct global warming potentials, *J. Photochem. Photobiol. A-Chem.*, *157*(2-3), 211-222, doi: 10.1016/s1010-6030(03)00057-1.

Orlando, J. J., G. S. Tyndall, A. Huang, and J. G. Calvert (1992), Temperature-dependence of the infrared-absorption cross-sections of carbon-tetrachloride, *Geophys. Res. Lett.*, *19*(10), 1005-1008, doi: 10.1029/91gl01036.

Oyaro, N., S. R. Sellevag, and C. J. Nielsen (2004), Study of the OH and Cl-initiated oxidation, IR absorption cross-section, radiative forcing, and global warming potential of four C_4-hydrofluoroethers, *Environ. Sci. Technol.*, *38*(21), 5567-5576, doi: 10.1021/es0497330.

Oyaro, N., S. R. Sellevag, and C. J. Nielsen (2005), Atmospheric chemistry of hydrofluoroethers: Reaction of a series of hydrofluoro ethers with OH radicals and Cl atoms, atmospheric lifetimes, and global warming potentials, *J. Phys. Chem. A*, *109*(2), 337-346, doi: 10.1021/jp047860c.

Papadimitriou, V. C., and J. B. Burkholder (2016), OH Radical Reaction Rate Coefficients, Infrared Spectrum, and Global Warming Potential of (CF3)2CFCH══CHF (HFO-1438ezy(E)), *The Journal of Physical Chemistry A*, *120*(33), 6618-6628, doi: 10.1021/acs.jpca.6b06096.

Papadimitriou, V. C., R. K. Talukdar, R. W. Portmann, A. R. Ravishankara, and J. B. Burkholder (2008a), $CF_3CF=CH_2$ and (*Z*)-$CF_3CF=CHF$: temperature dependent OH rate coefficients and global warming potentials, *Phys. Chem. Chem. Phys.*, *10*(6), 808-820, doi: 10.1039/b714382f.

Papadimitriou, V. C., R. W. Portmann, D. W. Fahey, J. Muhle, R. F. Weiss, and J. B. Burkholder (2008b), Experimental and Theoretical Study of the Atmospheric Chemistry and Global Warming Potential of SO_2F_2, *J. Phys. Chem. A*, *112*(49), 12657-12666, doi: 10.1021/jp806368u.

Papadimitriou, V. C., C. S. Spitieri, P. Papagiannakopoulos, M. Cazaunau, M. Lendar, V. Daele, and A. Mellouki (2015), Atmospheric chemistry of (CF3)2C[double bond, length as m-dash]CH2: OH radicals, Cl atoms and O3 rate coefficients, oxidation end-products and IR spectra, *Phys. Chem. Chem. Phys.*, *17*(38), 25607-25620, doi: 10.1039/C5CP03840E.

Papadimitriou, V. C., M. R. McGillen, S. C. Smith, A. M. Jubb, R. W. Portmann, B. D. Hall, E. L. Fleming, C. H. Jackman, and J. B. Burkholder (2013), 1,2-Dichlorohexafluoro-cyclobutane (1,2-c-$C_4F_6Cl_2$, R-316c) a Potent Ozone Depleting Substance and Greenhouse Gas: Atmospheric Loss Processes, Lifetimes, and Ozone Depletion and Global Warming Potentials for the (E) and (Z) Stereoisomers, *The Journal of Physical Chemistry A*, *117*(43), 11049-11065, doi: 10.1021/jp407823k.

Peirone, S. A., J. A. Barrera, R. A. Taccone, P. M. Cometto, and S. I. Lane (2014), Relative rate coefficient measurements of OH radical reactions with (Z)-2-hexen-1-ol and (E)-3-hexen-1-ol under simulated atmospheric conditions, *Atmos. Environ.*, *85*(Supplement C), 92-98, doi: https://doi.org/10.1016/j.atmosenv.2013.11.076.

Person, W. B., and S. R. Polo (1961), Infrared intensities of the fundamental frequencies of CF_3Br, *Spectrochimica Acta*, *17*(1), 101-111, doi: 10.1016/0371-1951(61)80016-7.

Pinnock, S., M. D. Hurley, K. P. Shine, T. J. Wallington, and T. J. Smyth (1995), Radiative forcing of climate by hydrochlorofluorocarbons and hydrofluorocarbons, *J. Geophys. Res.-Atmos.*, *100*(D11), 23227-23238, doi: 10.1029/95jd02323.

Rajakumar, B., R. W. Portmann, J. B. Burkholder, and A. R. Ravishankara (2006), Rate coefficients for the reactions of OH with $CF_3CH_2CH_3$ (HFC-263fb), CF_3CHFCH_2F (HFC-245eb), and $CHF_2CHFCHF_2$ (HFC-245ea) between 238 and 375 K, *J. Phys. Chem. A*, *110*(21), 6724-6731, doi: 10.1021/jp056248y.

Ramanathan, V., R. J. Cicerone, H. B. Singh, and J. T. Kiehl (1985), Trace gas trends and their potential role in climate change, *J. Geophys. Res.-Atmos.*, *90*(ND3), 5547-5566, doi: 10.1029/JD090iD03p05547.

Ren, Y. G., F. Bernard, V. Daele, and A. Mellould (2019), Atmospheric Fate and Impact of Perfluorinated Butanone and Pentanone, *Environ. Sci. Technol.*, *53*(15), 8862-8871, doi: 10.1021/acs.est.9b02974.

Rinsland, C. P., S. W. Sharpe, and R. L. Sams (2003), Temperature-dependent absorption cross-sections in the thermal infrared bands of SF_5CF_3, *J. Quant. Spectrosc. Radiat. Transf.*, *82*(1-4), 483-490, doi: 10.1016/s0022-4073(03)00172-9.

Rinsland, C. P., V. M. Devi, T. A. Blake, R. L. Sams, S. Sharpe, and L. Chiou (2008), Quantitative measurement of integrated band intensities of benzene vapor in the mid-infrared at 278, 298, and 323 K, *J. Quant. Spectrosc. Radiat. Transf.*, *109*(15), 2511-2522, doi: 10.1016/j.jqsrt.2008.04.007.

Robson, J. I., L. K. Gohar, M. D. Hurley, K. P. Shine, and T. J. Wallington (2006), Revised IR spectrum, radiative efficiency and global warming potential of nitrogen trifluoride, *Geophys. Res. Lett.*, *33*(10), doi: 10.1029/2006gl026210.

Rodriguez, A., I. Bravo, D. Rodriguez, M. Tajuelo, Y. Diaz-de-Mera, and A. Aranda (2016), The environmental impact of unsaturated fluoroesters: atmospheric chemistry towards OH radicals and Cl atoms, radiative behavior and cumulative ozone creation, *RSC Advances*, *6*(26), 21833-21843, doi: 10.1039/C6RA00630B.

Rodríguez, A., D. Rodríguez, A. Moraleda, I. Bravo, E. Moreno, and A. Notario (2014), Atmospheric chemistry of HFE-7300 and HFE-7500: Temperature dependent kinetics, atmospheric lifetimes, infrared spectra and global warming potentials, *Atmos. Environ.*, *96*(Supplement C), 145-153, doi: https://doi.org/10.1016/j.atmosenv.2014.07.033.

Roehl, C. M., D. Boglu, C. Bruhl, and G. K. Moortgat (1995), Infrared band intensities and global warming potentials of CF_4, C_2F_6, C_3F_8, C_4F_{10}, C_5F_{12}, and C_6F_{14}, *Geophys. Res. Lett.*, *22*(7), 815-818, doi: 10.1029/95gl00488.

Rogers, J. D., and R. D. Stephens (1988), Absolute infrared intensities for F-113 and F-114 and an assessment of their greenhouse warming potential relative to other chlorofluorocarbons, *J. Geophys. Res.-Atmos.*, *93*(D3), 2423-2428, doi: 10.1029/JD093iD03p02423.

Ryan, S. M., and C. J. Nielsen (2010), Global Warming Potential of Inhaled Anesthetics: Application to Clinical Use, *Anesthesia and Analgesia*, *111*(1), 92-98, doi: 10.1213/ANE.0b013e3181e058d7.

Schatz, P. N., and D. F. Hornig (1953), Bond moments and derivatives in CF_4, SIF_4, and SF_6 from infrared intensities, *J. Chem. Phys.*, *21*(9), 1516-1530, doi: 10.1063/1.1699291.

Sellevag, S. R., T. Kelly, H. Sidebottom, and C. J. Nielsen (2004a), A study of the IR and UV-Vis absorption cross-sections, photolysis and OH-initiated oxidation of CF_3CHO and CF_3CH_2CHO, *Phys. Chem. Chem. Phys.*, *6*(6), 1243-1252, doi: 10.1039/b315941h.

Sellevag, S. R., C. J. Nielsen, O. A. Sovde, G. Myhre, J. K. Sundet, F. Stordal, and I. S. A. Isaksen (2004b), Atmospheric gas-phase degradation and global warming potentials of 2-fluoro ethanol, 2,2-difluoroethanol, and 2,2,2-trifluoroethanol, *Atmos. Environ.*, *38*(39), 6725-6735, doi: 10.1016/j.atmosenv.2004.09.023.

Sellevåg, S. R., B. D'Anna, and C. J. Nielsen (2007), Infrared Absorption Cross-Sections and Estimated Global Warming Potentials of $CF_3CH_2CH_2OH$, $CHF_2CF_2CH_2OH$, $CF_3CF_2CH_2OH$, $CF_3CHFCF_2CH_2OH$, and $CF_3CF_2CF_2CH_2OH$, *Asian Chemistry Letters*(11), 33-40.

Sharpe, S. W., T. J. Johnson, R. L. Sams, P. M. Chu, G. C. Rhoderick, and P. A. Johnson (2004), Gas-phase databases for quantitative infrared spectroscopy, *Applied Spectroscopy*, *58*(12), 1452-1461, doi: 10.1366/0003702042641281.

Shine, K. P., and G. Myhre (2020), The Spectral Nature of Stratospheric Temperature Adjustment and its Application to Halocarbon Radiative Forcing, *Journal of Advances in Modeling Earth Systems*, *12*(3), e2019MS001951, doi: 10.1029/2019MS001951.

Shine, K. P., L. K. Gohar, M. D. Hurley, G. Marston, D. Martin, P. G. Simmonds, T. J. Wallington, and M. Watkins (2005), Perfluorodecalin: global warming potential and first detection in the atmosphere, *Atmos. Environ.*, *39*(9), 1759-1763, doi: 10.1016/j.atmosenv.2005.01.001.

Sihra, K., M. D. Hurley, K. P. Shine, and T. J. Wallington (2001), Updated radiative forcing estimates of 65 halocarbons and nonmethane hydrocarbons, *J. Geophys. Res.-Atmos.*, *106*(D17), 20493-20505, doi: 10.1029/2000jd900716.

Smith, K., D. Newnham, M. Page, J. Ballard, and G. Duxbury (1996), Infrared band strengths and absorption cross-sections of HFC-32 vapour, *J. Quant. Spectrosc. Radiat. Transf.*, *56*(1), 73-82, doi: 10.1016/0022-4073(96)00019-2.

Smith, K., D. Newnham, M. Page, J. Ballard, and G. Duxbury (1998), Infrared absorption cross-sections and integrated absorption intensities of HFC-134 and HFC-143a vapour, *J. Quant. Spectrosc. Radiat. Transf.*, *59*(3-5), 437-451, doi: 10.1016/s0022-4073(97)00114-3.

Stoppa, P., A. P. Charmet, N. Tasinato, S. Giorgianni, and A. Gambi (2009), Infrared Spectra, Integrated Band Intensities, and Anharmonic Force Field of H_2C=CHF, *J. Phys. Chem. A*, *113*(8), 1497-1504, doi: 10.1021/jp808556e.

Sturges, W. T., T. J. Wallington, M. D. Hurley, K. P. Shine, K. Sihra, A. Engel, D. E. Oram, S. A. Penkett, R. Mulvaney, and C. A. M. Brenninkmeijer (2000), A potent greenhouse gas identified in the atmosphere: SF_5CF_3, *Science*, *289*(5479), 611-613, doi: 10.1126/science.289.5479.611.

Suga, A., Y. Mochizuki, N. Nagasaki, Y. Gotoh, H. Ito, S. Yamashita, T. Uchimaru, M. Sugie, A. Sekiya, S. Kondo, and M. Aoyagi (1994), Estimation of total infrared intensities of fluorinated ethyl methyl ethers, *Chemistry Letters*(12), 2365-2368, doi: 10.1246/cl.1994.2365.

Søndergaard, R., O. J. Nielsen, M. D. Hurley, T. J. Wallington, and R. Singh (2007), Atmospheric chemistry of trans-CF_3CH = CHF: Kinetics of the gas-phase reactions with Cl atoms, OH radicals, and O_3, *Chem. Phys. Lett.*, *443*(4-6), 199-204, doi: 10.1016/j.cplett.2007.06.084.

Takahashi, K., Y. Matsumi, T. J. Wallington, and M. D. Hurley (2002), Atmospheric chemistry of $CF_3CFHOCF_3$: Reaction with OH radicals, atmospheric lifetime, and global warming potential, *J. Geophys. Res.-Atmos.*, *107*(D21), doi: 10.1029/2002jd002125.

Tanabe, K., and S. Saeki (1970), Calculation of infrared band intensities of various chlorinated methanes, *Spectrochimica Acta Part a-Molecular Spectroscopy*, *A 26*(7), 1469-&, doi: 10.1016/0584-8539(70)80208-2.

Tereszchuk, K. A., and P. F. Bernath (2011), Infrared absorption cross-sections for acetaldehyde (CH3CHO) in the 3 mu m region, *J. Quant. Spectrosc. Radiat. Transf.*, *112*(6), 990-993, doi: 10.1016/j.jqsrt.2010.12.003.

Thomsen, D. L., V. F. Andersen, O. J. Nielsen, and T. J. Wallington (2011), Atmospheric chemistry of $C_2F_5CH_2OCH_3$ (HFE-365mcf), *Phys. Chem. Chem. Phys.*, *13*(7), 2758-2764.

Tokuhashi, K., K. Takizawa, and S. Kondo (2018), Rate constants for the reactions of OH radicals with CF3CX=CY2 (X = H, F, CF3, Y = H, F, Cl), *Environmental Science and Pollution Research*, *25*(15), 15204-15215, doi: 10.1007/s11356-018-1700-4.

Totterdill, A., T. Kovacs, W. H. Feng, S. Dhomse, C. J. Smith, J. C. Gomez-Martin, M. P. Chipperfield, P. M. Forster, and J. M. C. Plane (2016), Atmospheric lifetimes, infrared absorption spectra, radiative forcings and global warming potentials of NF_3 and CF_3CF_2Cl (CFC-115), *Atmos. Chem. Phys.*, *16*(17), 11451-11463, doi: 10.5194/acp-16-11451-2016.

Vander Auwera, J. (2000), Infrared absorption cross-sections for two substituted ethanes: 1,1-difluoroethane (HFC-152a) and 1,2-dichloroethane, *J. Quant. Spectrosc. Radiat. Transf.*, *66*(2), 143-151.

Vanthanh, N., I. Rossi, A. Jeanlouis, and H. Rippel (1986), Infrared band shapes and band strengths of CF_2Cl_2 from 800 to 1200 cm^{-1} at 296 and 200 K, *J. Geophys. Res.-Atmos.*, *91*(D3), 4056-4062.

Varanasi, P., and F. K. Ko (1977), Intensity measurements in freon bands of atmospheric interest, *J. Quant. Spectrosc. Radiat. Transf.*, *17*(3), 385-388, doi: 10.1016/0022-4073(77)90116-9.

Varanasi, P., and S. Chudamani (1988a), Remeasurement of the absolute intensities of CFC-11 ($CFCl_3$) and CFC-12 (CF_2Cl_2), *J. Quant. Spectrosc. Radiat. Transf.*, *39*(3), 193-195, doi: 10.1016/s0022-4073(88)90026-x.

Varanasi, P., and S. Chudamani (1988b), Infrared intensities of some chlorofluorocarbons capable of perturbing the global climate, *J. Geophys. Res.-Atmos.*, *93*(D2), 1666-1668, doi: 10.1029/JD093iD02p01666.

Varanasi, P., and V. Nemtchinov (1994), Thermal infrared-absorption coefficients of CFC-12 at atmospheric conditions, *J. Quant. Spectrosc. Radiat. Transf.*, *51*(5), 679-687, doi: 10.1016/0022-4073(94)90124-4.

Varanasi, P., Z. Li, V. Nemtchinov, and A. Cherukuri (1994), Spectral absorption-coefficient data on HCFC-22 and SF_6 for remote-sensing applications, *J. Quant. Spectrosc. Radiat. Transf.*, *52*(3-4), 323-332, doi: 10.1016/0022-4073(94)90162-7.

Vasekova, E., E. A. Drage, K. M. Smith, and N. J. Mason (2006), FTIR spectroscopy and radiative forcing of octafluorocyclobutane and octofluorocyclopentene, *J. Quant. Spectrosc. Radiat. Transf.*, *102*(3), 418-424, doi: 10.1016/j.jqsrt.2006.02.023.

Vlachogiannis, D., A. Sfetsos, A. K. Stubos, R.-E. P. Sotiropoulou, E. Tagaris, C. Pilinis, W. Zhong, J. D. Haigh, D. O. Eriksen, S. K. Hartvig, C. Chatzichristos, J. Muller, R. Kleven, and I. Nielssen (2005), Assessment of the impact of SF_6 and PFC reservoir tracers on global warming, the AEOLOS study, *Environmental Sciences*, *2*(2-3), 263-272, doi: 10.1080/15693430500396170.

Vollmer, M. K., F. Bernard, B. Mitrevski, L. P. Steele, C. M. Trudinger, S. Reimann, R. L. Langenfelds, P. B. Krummel, P. J. Fraser, D. M. Etheridge, M. A. J. Curran, and J. B. Burkholder (2019), Abundances, emissions, and loss processes of the long-lived and potent greenhouse gas octafluorooxolane (octafluorotetrahydrofuran, c-C4F8O) in the atmosphere, *Atmos. Chem. Phys.*, *19*(6), 3481-3492, doi: 10.5194/acp-19-3481-2019.

Wagner, G., and A. Birk (2003), New infrared spectroscopic database for chlorine nitrate, *J. Quant. Spectrosc. Radiat. Transf.*, *82*(1-4), 443-460, doi: 10.1016/s0022-4073(03)00169-9.

Wagner, G., and M. Birk (2016), New infrared spectroscopic database for bromine nitrate, *J. Mol. Spectrosc.*, *326*, 95-105, doi: 10.1016/j.jms.2016.03.007.

Wallington, T. J., M. D. Hurley, and O. J. Nielsen (2009), The radiative efficiency of $HCF_2OCF_2OCF_2CF_2OCF_2H$ (H-Galden 1040x) revisited, *Atmos. Environ.*, *43*(27), 4247-4249, doi: 10.1016/j.atmosenv.2009.05.046.

Wallington, T. J., M. D. Hurley, O. J. Nielsen, and M. P. S. Andersen (2004), Atmospheric chemistry of $CF_3CFHCF_2OCF_3$ and $CF_3CFHCF_2OCF_2H$: Reaction with Cl atoms and OH radicals, degradation mechanism, and global warming potentials, *J. Phys. Chem. A*, *108*(51), 11333-11338, doi: 10.1021/jp046454q.

Wallington, T. J., M. D. Hurley, J. C. Ball, T. Ellermann, O. J. Nielsen, and J. Sehested (1994), Atmospheric chemistry of HFC-152 - UV absorption-spectrum of CH_2FCFHO_2 radicals, kinetics of the reaction CH_2FCFHO_2+NO-> $CH_2FCHFO+NO_2$, and fate of the alkoxy radical CH_2FCFHO, *Journal of Physical Chemistry*, *98*(21), 5435-5440, doi: 10.1021/j100072a008.

Wallington, T. J., A. Guschin, T. N. N. Stein, J. Platz, J. Sehested, L. K. Christensen, and O. J. Nielsen (1998), Atmospheric chemistry of $CF_3CH_2OCH_2CF_3$: UV spectra and kinetic data for $CF_3CH(\bullet)OCH_2CF_3$ and $CF_3CH(OO\bullet)OCH_2CF_3$ radicals and atmospheric fate of $CF_3CH(O\bullet)OCH_2CF_3$ radicals, *J. Phys. Chem. A*, *102*(7), 1152-1161, doi: 10.1021/jp972933w.

Wallington, T. J., B. P. Pivesso, A. M. Lira, J. E. Anderson, C. J. Nielsen, N. H. Andersen, and Ø. Hodnebrog (2016), CH_3Cl, CH_2Cl_2, $CHCl_3$, and CCl_4: Infrared spectra, radiative efficiencies, and global warming potentials, *Journal of Quantitative Spectroscopy and Radiative Transfer*, *174*(Supplement C), 56-64, doi: https://doi.org/10.1016/j.jqsrt.2016.01.029.

Wallington, T. J., W. F. Schneider, J. Sehested, M. Bilde, J. Platz, O. J. Nielsen, L. K. Christensen, M. J. Molina, L. T. Molina, and P. W. Wooldridge (1997), Atmospheric chemistry of HFE-7100 ($C_4F_9OCH_3$): Reaction with OH radicals, UV spectra and kinetic data for $C_4F_9OCH_2\bullet$ and $C_4F_9CH_2O_2\bullet$ radicals, and the atmospheric fate of $C_4F_9OCH_2O$ center dot radicals, *J. Phys. Chem. A*, *101*(44), 8264-8274, doi: 10.1021/jp971353w.

Waterland, R. L., M. D. Hurley, J. A. Misner, T. J. Wallington, S. M. L. Melo, K. Strong, R. Dumoulin, L. Castera, N. L. Stock, and S. A. Mabury (2005), Gas phase UV and IR absorption spectra of $CF_3CH_2CH_2OH$ and $F(CF_2CF_2)_xCH_2CH_2OH$ (x=2, 3, 4), *J. Fluor. Chem.*, *126*(9-10), 1288-1296, doi: 10.1016/j.jfluchem.2005.06.010.

Wetzel, G., H. Oelhaf, O. Kirner, R. Ruhnke, F. Friedl-Vallon, A. Kleinert, G. Maucher, H. Fischer, M. Birk, G. Wagner, and A. Engel (2010), First remote sensing measurements of ClOOCl along with ClO and ClONO2 in activated and deactivated Arctic vortex conditions using new ClOOCl IR absorption cross sections, *Atmos. Chem. Phys.*, *10*(3), 931-945, doi: 10.5194/acp-10-931-2010.

WMO (2019), Scientific Assessment of Ozone Depletion: 2018, Global Ozone Research and Monitoring Project — Report No. 58, 588 pp., World Meteorological Organization, Geneva, Switzerland*Rep.*

Young, C. J., M. D. Hurley, T. J. Wallington, and S. A. Mabury (2006), Atmospheric lifetime and global warming potential of a perfluoropolyether, *Environ. Sci. Technol.*, *40*(7), 2242-2246, doi: 10.1021/es052077z.

Young, C. J., M. D. Hurley, T. J. Wallington, and S. A. Mabury (2009a), Atmospheric chemistry of perfluorobutenes ($CF_3CFCFCF_3$ and $CF_3CF_2CFCF_2$): Kinetics and mechanisms of reactions with OH radicals and chlorine atoms, IR spectra, global warming potentials, and oxidation to perfluorocarboxylic acids, *Atmos. Environ.*, *43*(24), 3717-3724, doi: 10.1016/j.atmosenv.2009.04.025.

Young, C. J., M. D. Hurley, T. J. Wallington, and S. A. Mabury (2009b), Atmospheric chemistry of CF_3CF_2H and $CF_3CF_2CF_2CF_2H$: Kinetics and products of gas-phase reactions with Cl atoms and OH radicals, infrared spectra, and formation of perfluorocarboxylic acids, *Chem. Phys. Lett.*, *473*(4-6), 251-256, doi: 10.1016/j.cplett.2009.04.001.

Zander, R., C. P. Rinsland, C. B. Farmer, and R. H. Norton (1987), Infrared spectroscopic measurements of halogenated source gases in the stratosphere with the ATMOS instrument, *J. Geophys. Res.-Atmos.*, *92*(D8), 9836-9850, doi: 10.1029/JD092iD08p09836.

Zhang, H., J. X. Wu, and P. Luc (2011a), A study of the radiative forcing and global warming potentials of hydrofluorocarbons, *J. Quant. Spectrosc. Radiat. Transf.*, *112*(2), 220-229, doi: 10.1016/j.jqsrt.2010.05.012.

Zhang, H., J. X. Wu, and Z. P. Shen (2011b), Radiative forcing and global warming potential of perfluorocarbons and sulfur hexafluoride, *Science China-Earth Sciences*, *54*(5), 764-772, doi: 10.1007/s11430-010-4155-0.

Zhang, N., T. Uchimaru, Q. Guo, F. Qing, L. Chen, and J. Mizukado (2017), Atmospheric chemistry of perfluorocyclopentene (cyc-$CF_2CF_2CF_2CF{=}CF-$): Kinetics, products and mechanism of gas-phase reactions with OH radicals, and atmospheric implications, *Atmos. Environ.*, *160*(Supplement C), 46-54, doi: https://doi.org/10.1016/j.atmosenv.2017.04.012.

Zhang, N., L. Chen, T. Uchimaru, F. Qing, J. Mizukado, H. Quan, and H. Suda (2015), Kinetics of gas-phase reactions of cyc-CF2CF2CF2CHFCH2 and trans-cyc-CF2CF2CF2CHFCHF with OH radicals between 253 and 328K, *Chem. Phys. Lett.*, *639*(Supplement C), 199-204, doi: https://doi.org/10.1016/j.cplett.2015.09.020.

Zou, Q., C. Sun, V. Nemtchinov, and P. Varanasi (2004), Thermal infrared cross-sections of C_2F_6 at atmospheric temperatures, *J. Quant. Spectrosc. Radiat. Transf.*, *83*(2), 215-221, doi: 10.1016/s0022-4073(02)00353-9.

Østerstrøm, F. F., O. J. Nielsen, and T. J. Wallington (2016), Atmospheric chemistry of CF3CF2OCH3, *Chem. Phys. Lett.*, *653*(Supplement C), 149-154, doi: https://doi.org/10.1016/j.cplett.2016.04.086.

Østerstrøm, F. F., O. J. Nielsen, M. P. S. Andersen, and T. J. Wallington (2012), Atmospheric chemistry of $CF_3CH_2OCH_3$: Reaction with chlorine atoms and OH radicals, kinetics, degradation mechanism and global warming potential, *Chem. Phys. Lett.*, *524*, 32-37, doi: 10.1016/j.cplett.2011.12.047.

Østerstrøm, F. F., T. J. Wallington, M. P. Sulbaek Andersen, and O. J. Nielsen (2015), Atmospheric Chemistry of (CF3)2CHOCH3, (CF3)2CHOCHO, and CF3C(O)OCH3, *The Journal of Physical Chemistry A*, *119*(42), 10540-10552, doi: 10.1021/acs.jpca.5b08204.

Østerstrøm, F. F., S. T. Andersen, T. I. Solling, O. J. Nielsen, and M. P. Sulbaek Andersen (2017), Atmospheric chemistry of Z- and E-CF3CH[double bond, length as m-dash]CHCF3, *Phys. Chem. Chem. Phys.*, *19*(1), 735-750, doi: 10.1039/C6CP07234H.
